# Expression quantitative trait loci for PI3K/AKT pathway

**DOI:** 10.1097/MD.0000000000005817

**Published:** 2017-01-10

**Authors:** Dongchan Ryu, Chaeyoung Lee

**Affiliations:** Department of Bioinformatics and Life Science, Soongsil University, Dongjak-gu, Seoul, Korea.

**Keywords:** collagen, expression quantitative trait locus, genome-wide association study, PI3K/AKT pathway, single nucleotide variant

## Abstract

A genome-wide association study (GWAS) was conducted to identify expression quantitative trait loci (eQTLs) for the genes involved in phosphatidylinositol-3-kinase/v-akt murine thymoma viral oncogene homolog (PI3K/AKT) pathway.

Data on mRNA expression of 341 genes in lymphoblastoid cell lines of 373 Europeans recruited by the 1000 Genomes Project using Illumina HiSeq2000 were utilized. We used their genotypes at 5,941,815 nucleotide variants obtained by Genome Analyzer II and SOLiD.

The association analysis revealed 4166 nucleotide variants associated with expression of 85 genes (*P* < 5 × 10^−8^). A total of 73 eQTLs were identified as association signals for the expression of multiple genes. They included 9 eQTLs for both of the genes encoding *collagen type I alpha 1* (*COL1A1*) and *integrin alpha 11* (*ITGA11*), which synthesize a major complex of plasma membrane. They also included eQTLs for type IV collagen molecules; 13 eQTLs for both *collagen type IV alpha 1* (*COL4A1*) and *collagen type IV alpha 2* (*COL4A2*) and 18 eQTLs for both *collagen type IV alpha 5* (*COL4A5*) and *collagen type IV alpha 6* (*COL4A6*). Some genes expressed by the eQTLs might induce expression of the genes encoding type IV collagen. One eQTL (rs16871986) was located in the promoter of *palladin* (*PALLD*) gene which might synthesize collagen by activating fibroblasts through the PI3K/AKT pathway. Another eQTL (rs34845474) was located in an enhancer of *cadherin related family member 3* (*CDHR3*) gene which can mediate cell adhesion.

This study showed a profile of eQTLs for the genes involved in the PI3K/AKT pathway using a healthy population, revealing 73 eQTLs associated with expression of multiple genes. They might be candidates of common variants in predicting genetic susceptibility to cancer and in targeting cancer therapy. Further studies are required to examine their underlying mechanisms for regulating expression of the genes.

## Abstract 2

Supplemental Digital Content is available in the text

## 1 Introduction

Phosphatidylinositol-3-kinase/v-akt murine thymoma viral oncogene homolog (PI3K/AKT) pathway plays an essential role in cellular functions including cell growth, cell survival, cell proliferation, and glucose metabolism.^[1–3]^ Many components of the pathway which consists of multiple kinase cascades are implicated in a variety of types of human cancers and have greatly been concerned as attractive pharmacological targets.^[4–6]^ Everolimus and temsirolimus as inhibitors of *mammalian target of rapamycin* (*mTOR*) have been approved for cancer treatment, and some inhibitors of its components such as *PI3K*, *AKT*, and/or *mTOR* (eg, buparlisib as a pan-*PI3K* inhibitor and alpelisib as a *PI3Kα*-selective inhibitor) are currently in clinical development.^[7,8]^ Many studies on the PI3K/AKT pathway have focused on targeting cancer therapy, and its genetic studies were mostly to examine mutations and its contribution to tumorigenesis and tumor maintenance by hyper-activating the PI3K/AKT pathway.^[9]^ Somatic mutations of *phosphatidylinositol-3-kinase, catalytic, polypeptide* (*PIK3CA*) have been frequently observed within its kinase domain (H1047R in exon 20) and helical domain (E542K and E545K in exon 9) in a variety of cancers.^[10]^ Germline mutations in exons of *phosphatase and tensin homolog* (*PTEN*) are well known for causes of hamartoma tumor syndromes.^[1,11,12]^

Moreover, common genetic variants in *PTEN*, *mTOR*, *AKT1*, and *AKT2* were found to be associated with clinical outcomes such as response to therapy, survival, and recurrence in esophageal cancer patients treated with chemoradiotherapy (*P* < 0.05).^[13]^ However, common genetic variants for regulating the pathway have been rarely investigated in healthy populations. Associations of common genetic variants with phosphorylation of *AKT1* or *RPS6KB1* were identified in lymphoblastoid cell lines of Centre d’Etude du Polymorphisme Humain (6.35 × 10^−5^ < *P* < 4.20 × 10^−3^).^[14]^ In this study, we conducted a genome-wide association study to examine genetic associations of common variants with mRNA expression of the genes involved in the PI3K/AKT pathway and to see whether the variants simultaneously influence multiple genes in the pathway.

## 2 Materials and methods

### 2.1 Genotype and gene expression data

**Figure 1:**
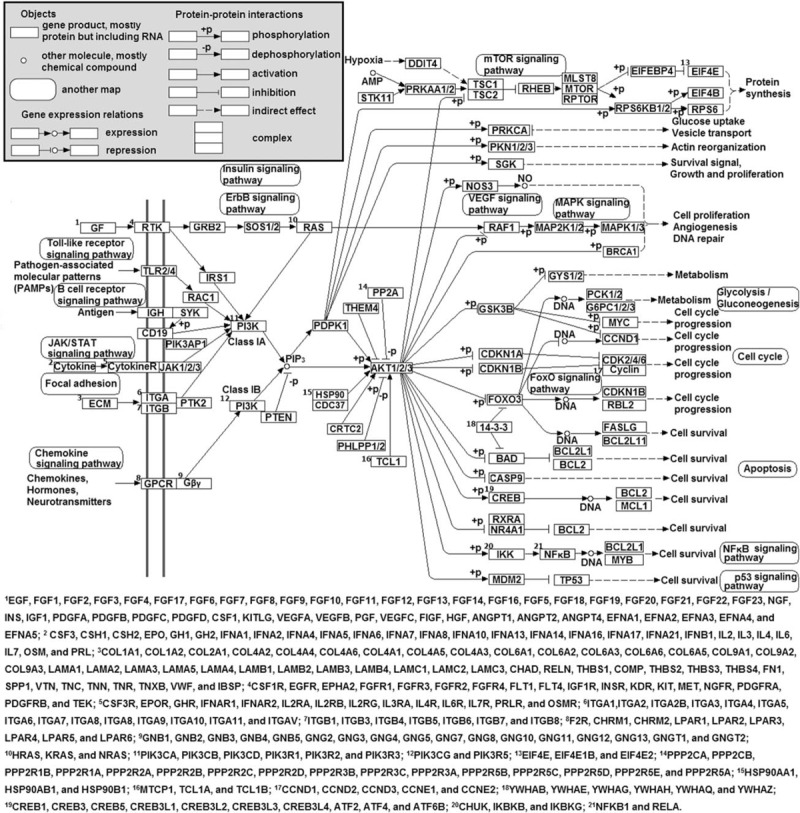
Diagram of genes in the PI3K/AKT signaling pathway. The genes were obtained from the KEGG pathway database and used in this study. KEGG = kyoto encyclopedia of genes and genomes, PI3K/AKT = phosphatidylinositol-3-kinase/v-akt murine thymoma viral oncogene homolog.

All the genes mapped in the PI3K/AKT pathway of the Kyoto Encyclopedia of Genes and Genomes pathway database (http://www.genome.jp/dbget-bin/www_bget?pathway+hsa04151) were selected (Fig. 1). This study used their mRNA expression data on lymphoblastoid cell lines of 373 individuals from European populations of Centre d’Etude du Polymorphisme Humain, Finns, British, and Toscani recruited by the 1000 Genomes Project.^[15]^ The expression levels were assessed through next generation sequencing using the Illumina HiSeq2000 platform with paired-end 75-bp mRNA-seq to reduce technical variation between laboratories than individuals (Mann–Whitney *U P* < 2.2 × 10^−6^).^[16]^ The RNAseq data with low mapping quality (MAPQ < 150) were excluded in the current study. Then, mRNA reads were mapped with an average of 48.9 million reads per sample using the GEM mapper.^[17]^ The expression of mRNA was quantified as the sum of all transcript reads per kilobase per million mapped reads per gene.^[15]^ All the expression data were normalized by probabilistic estimation of expression residuals to remove technical variation.^[18]^ Their genotypic data were obtained from the 1000 Genomes Project (phase 1) using Genome Analyser II and SOLiD.^[19]^ They included genotypes called using Beagle^[20]^ and imputed using and MaCH/Thunder.^[21]^ Variants with high confidence calls (phred-scaled quality score = 100) and high imputation quality (MaCH RSQ < 0.3) were used in this study. Depth of coverage was unavailable and thus could not be considered in filtering variants. Nucleotide variants with genotypic call rate <95%, with minor allele frequency <0.05, or in Hardy–Weinberg disequilibrium (*P* < 0.001) were removed, and a total of 5,941,815 nucleotide variants were used in the analysis. Ethical approval was not necessary because we dealt with publically available data.

### 2.2 Statistical analyses

The genome-wide association analysis for expression of 341 genes involved in the PI3K/AKT pathway was conducted using a mixed model.^[22]^ The analytical model employed a genetic relationship matrix for random polygenic effects to avoid population stratification as follows: 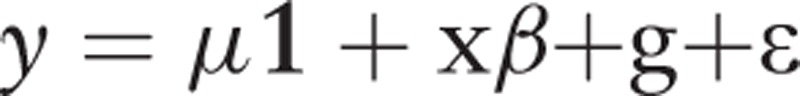


where **y** is the vector of gene expressions, μ the overall mean, **1** the vector of 1's, β the fixed effect for the minor allele of the candidate variant, and **x** is the vector with elements of 0, 1, and 2 for the homozygote of the minor allele, heterozygote, and homozygote of the major allele, **g** is the vector of random polygenic effects  
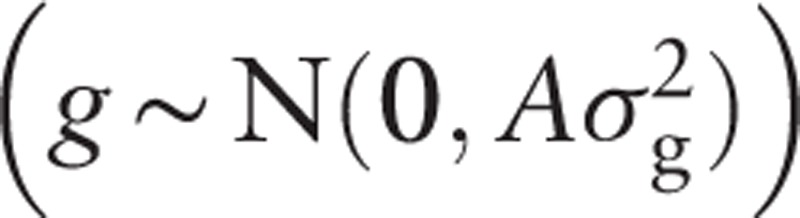
 where **A** is the genomic relationship matrix with elements of pairwise relationship coefficients and  
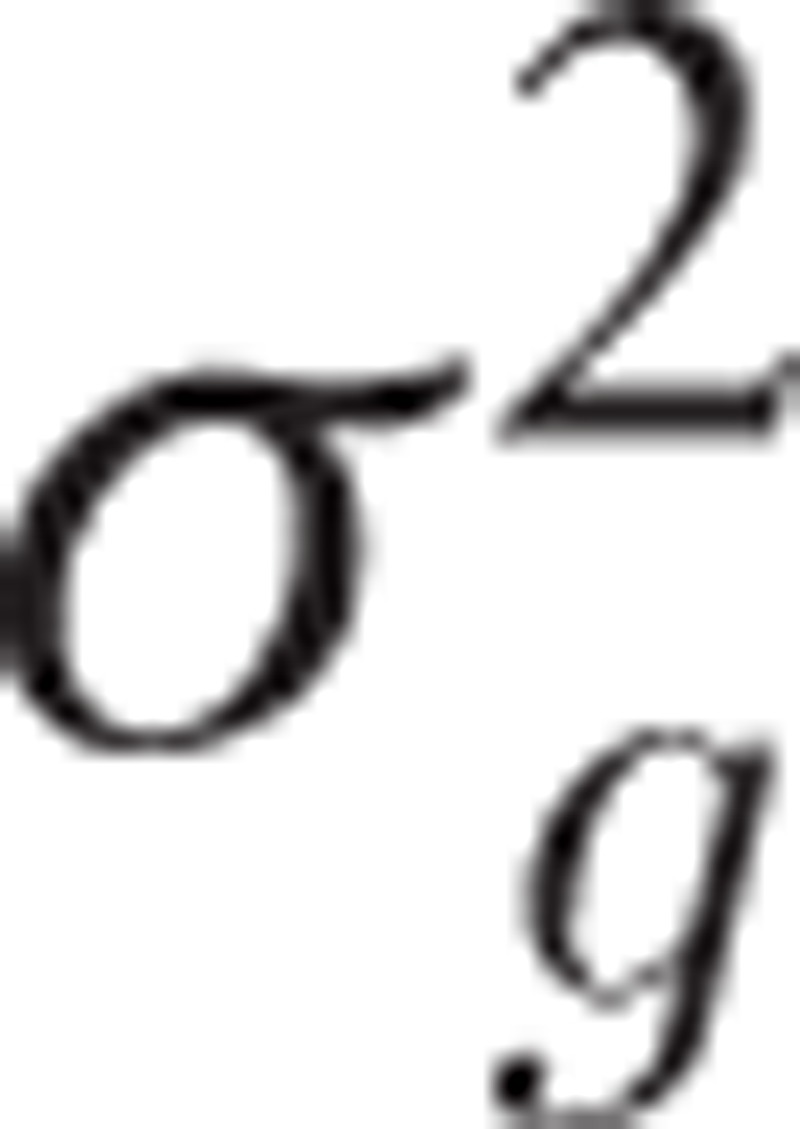
 is the polygenic variance component, **ε** is the vector of residual effects  
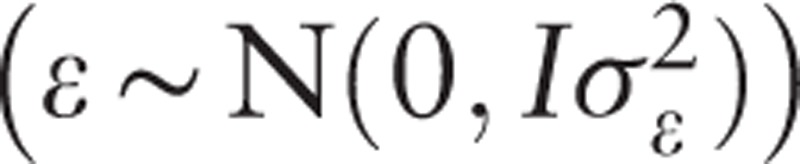
 where  
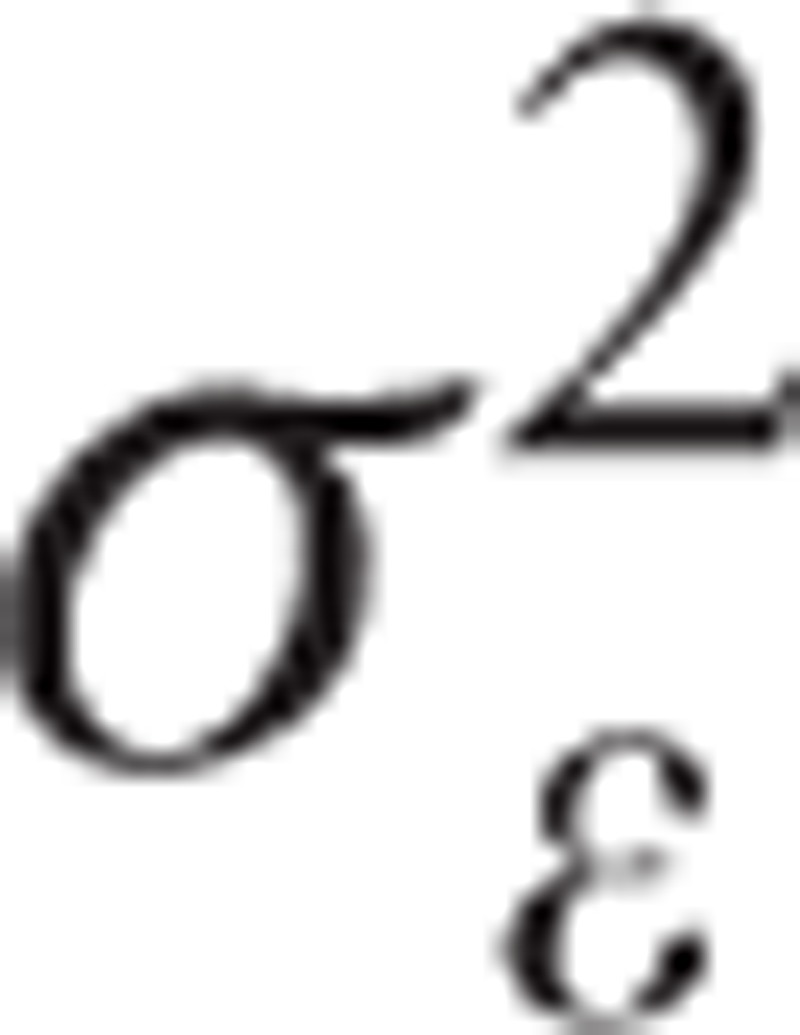
 is the residual variance component, and **I** is the identity matrix. The polygenic and residual variance components were estimated using restricted maximum likelihood. The fixed effect was then solved with the estimated variance components under the mixed model equations. The association analysis was conducted using Genome-wide Complex Trait Analysis version 1.26.^[23]^ Multiple testing was applied with a significance threshold of *P* < 5 × 10^−8^.^[24]^ Linkage disequilibrium blocks were mapped to exclude dependent associations produced by linkage. The blocks were determined based on the algorithm of Gabriel et al^[25]^ with 95% confidence interval of D′ value using Haploview.^[26]^ Potential deleteriousness of identified variants were examined using the combined annotation dependent depletion.^[27]^ Potential regulatory variants were also determined based on RegulomeDB.^[28]^ We further examined whether the variants associated with gene expression showed functional evidence for an enhancer or a promoter using data resulted from a chromatin interaction analysis with paired-end-tag sequencing (ChIA-PET) study.^[29]^ The ChIA-PET study was conducted with RNA polymerase II using MCF7 cells (human breast cancer cell line) and K562 cells (human erythroleukemic cell line). Correlation between gene expressions was assessed using SPSS 23.0 software for Windows (SPSS Inc., Chicago, IL). Epistasis was analyzed to examine interaction between expression quantitative loci (eQTLs) simultaneously identified for specific genes using linear regression with the function of “epistasis” implemented in PLINK version 1.9.^[30]^ Significance threshold was adjusted for the multiple testing by the Bonferroni correction. We conducted a functional enrichment analysis with the genes containing the eQTLs using ToppGene.^[31]^

## 3 Results

The genome-wide association analysis revealed 4166 single nucleotide polymorphisms (SNPs) associated with expression of 85 genes involved in the PI3K/AKT pathway (*P* < 5 × 10^−8^, Supplementary Table S1). Among them, 244 SNPs were associated with expression of multiple genes. The SNPs were contained in 73 linkage disequilibrium blocks (Supplementary Table S2; Supplementary Figs. S1 and S2). This indicates that 73 association signals were identified in the current study. The signals included 3 cis-eQTLs and 70 trans-eQTLs. There were one signals identified for association with 4 genes, 3 signals for association with 3 genes, and 69 signals for association with 2 genes. Most of the gene groups had multiple signals associated simultaneously with their expression. In particular, 13 association signals were associated with expression of both *collagen type IV alpha 1* (*COL4A1*) and *collagen type IV alpha 2* (*COL4A2*; Table 1), and 18 signals were associated with expression of both *collagen type IV alpha 5* (*COL4A5*) and *collagen type IV alpha 6* (*COL4A6*; Table 2).

**Table 1:**
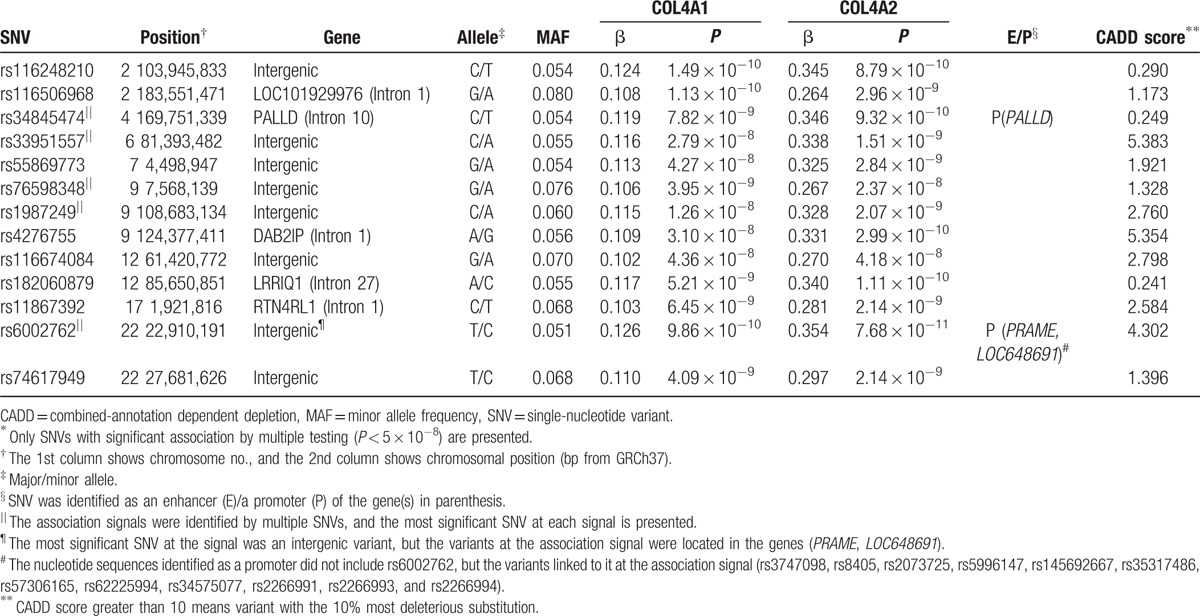
Nucleotide variants associated with gene expression of *COL4A1* and *COL4A2* in lymphoblastoid cell lines of Europeans by a genome-wide association study^∗^.

**Table 2:**
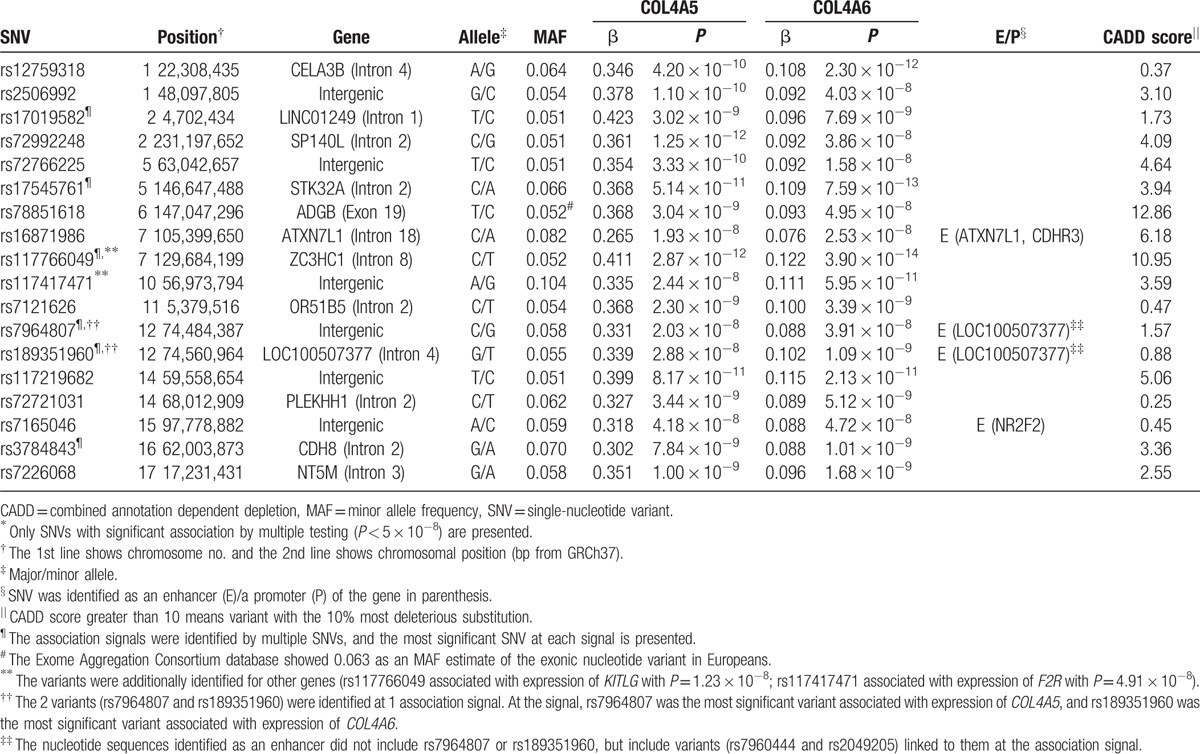
Nucleotide variants associated with gene expression of *COL4A5* and *COL4A6* in lymphoblastoid cell lines of Europeans by a genome-wide association study^∗^.

The combined annotation dependent depletion scores of the identified variants showed that rs78851618 and rs117766049 might have the 10% most deleterious substitution. The functional annotation analysis with RegulomeDB revealed variants likely to have regulatory functions (Supplementary Tables S3 and S4). Especially, both rs7165046 and rs145692667 had annotation for transcription factor binding, any motif, DNase footprint and DNase peak. These nucleotide variants were identified as an enhancer or a promoter identified by ChIA-PET (Tables 1 and 2).

**Figure 2:**
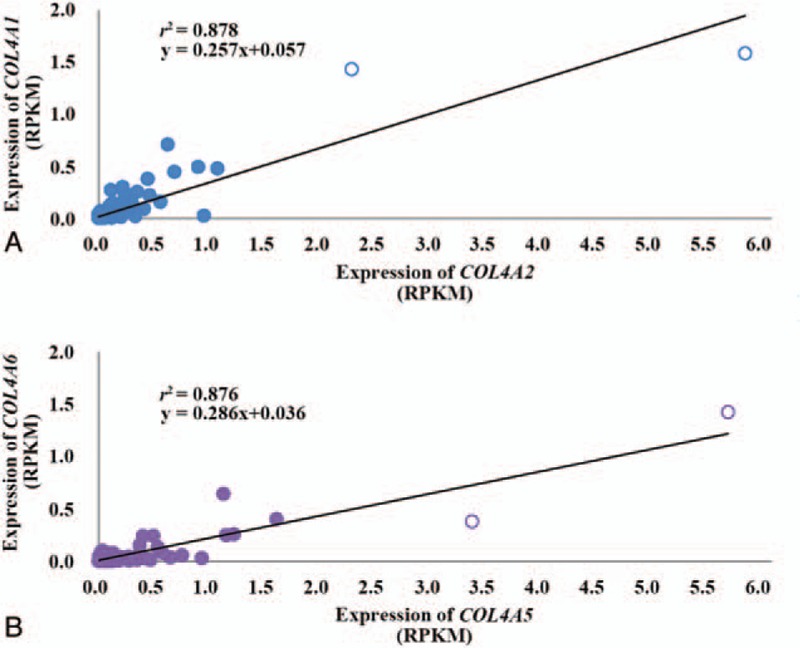
Correlation of expression between *COL4A1* and *COL4A2* (A), between *COL4A5* and *COL4A6* (B) in European lymphoblastoid cell lines. Unfilled circles indicate individuals with expression greater than 2 standard deviations of reads per kilobase per million mapped reads (RPKM).

Expression of *COL4A1* was positively correlated with that of *COL4A2* in lymphoblastoid cell lines (*r*^2^ = 0.878; Fig. 2A). There were 2 individuals with a large expression (>2 SD) of *COL4A2*. One of them had all homozygotes of minor alleles at single-nucleotide variants identified in Table 1. Similarly, expression of *COL4A5* was also positively correlated with that of *COL4A2* (*r*^2^ = 0.876; Fig. 2B). Two individuals were observed with a large expression (>2 SD) of *COL4A5*, and one of them had all homozygotes of minor alleles at single-nucleotide variants identified in Table 2. After removing individuals who had a large expression (>2 SD), positive correlations were still obtained (*P* < 0.05; *r*^2^ = 0.771 between *COL4A1* and *COL4A2*, *r*^2^ = 0.674 between *COL4A5* and *COL4A6*).

Further analysis showed interactions between the eQTLs significant for the genes encoding type IV collagen in Supplementary Tables S5 and S6. All the pairwise interactions were significant (*P* < 2.16 × 10^−4^).

**Figure 3:**
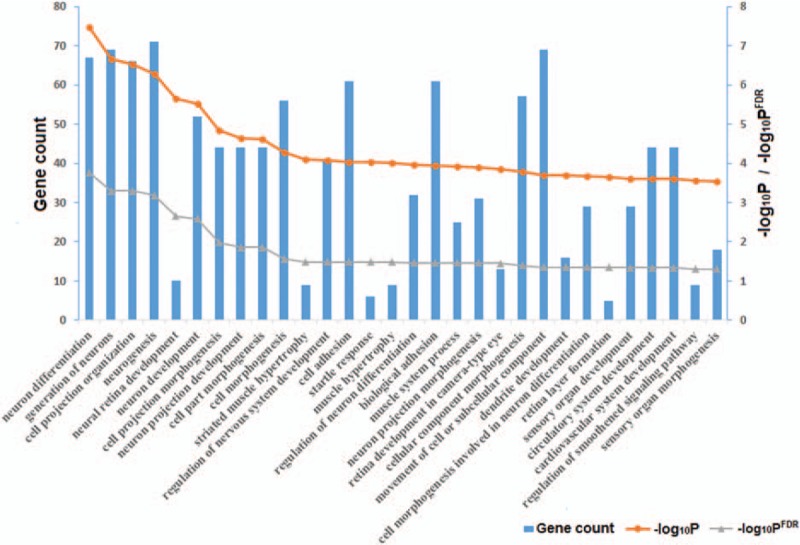
Functional enrichment (*P*^FDR^ < 0.05) of the genes containing eQTL for genes involved in the PI3K/AKT signaling pathway. eQTL = expression quantitative trait locus, PI3K/AKT = phosphatidylinositol-3-kinase/v-akt murine thymoma viral oncogene homolog.

Functional enrichment analysis showed that the genes containing the eQTLs were significantly enriched in many biological processes, especially in cellular functions such as cell differentiation, cell development, cell morphogenesis, and cell adhesion (FDR < 0.05; Fig. 3).

## 4 Discussion

The current study revealed 73 eQTLs associated with 2 or more genes involved in the PI3K/AKT pathway (*P* < 5 × 10^−8^). The gene groups were mostly associated with multiple eQTLs. The genes were simultaneously upregulated by specific allele of each eQTL. These results suggested that the genes in each group might be needed for incorporative functions. For example, 9 signals were identified for association with mRNA expression of both *collagen type I alpha 1* (*COL1A1*) and *integrin alpha 11* (*ITGA11*). Dimer of *ITGA11*/*integrin beta 1* (*ITGB1*) is well known as a receptor for *COL1A1*, and the complex of *ITGA11*/*ITGB1*/*COL1A1* is a major part of plasma membrane.^[32]^ In addition, 2 gene pairs are encoding type IV collagen molecules. The genes of *COL4A1* and *COL4A2* were associated with 13 eQTLs, and the genes of *COL4A5* and *COL4A6* were associated with 18 eQTLs. The collagen molecules are most abundant in basement membranes and are involved in ensuring structural integrity in tissues and in modulating cell differentiation, cell growth, and cell adhesion.^[33,34]^ The genes of *COL4A1* and *COL4A2* have a unique head-to-head orientation, and the genes of *COL4A5* and *COL4A6* also have it. They can initiate transcription from opposite DNA strands by sharing a bidirectional promoter.^[35,36]^ The *COL4A1* gene was 235 bp apart from *COL4A2* gene, and the *COL4A5* gene was 315 bp apart from *COL4A6* gene. The eQTLs located between the gene pairs are likely to be critical promoter regions. This was supported further by positive correlation of expression between the genes. On the other hand, functional roles of the eQTLs apart from the genes are hardly guessed. Some signals turned out to be an enhancer or a promoter of other genes by ChIA-PET. The rs16871986 associated with the genes of *COL4A5* and *COL4A6* was located within an enhancer of *cadherin related family member 3* (*CDHR3*) gene. The *CDHR3* mediates homophilic cell adhesion through its binding to calcium ions.^[37,38]^ This leads to a possibility of direct or indirect induction of *COL4A5* and *COL4A6* by *CDHR3*. Similarly, the rs34845474 associated with the genes of *COL4A1* and *COL4A2* was located within the promoter of *palladin* (*PALLD*). The *PALLD* regulates actin cytoskeletal organization and cell adhesion formation.^[39]^ Furthermore, it was identified as a key node in *focal adhesion kinase* (*FAK*) and *PI3K* of the PI3K/AKT pathway.^[40]^ Collagens are synthesized for a variety of human tissues by fibroblast which can be activated by *FAK* and *PI3K*.^[40]^ This suggests the *PALLD* might ultimately induce *COL4A1* and *COL4A2*.

The 13 eQTLs identified for both *COL4A1* and *COL4A2* were supported by positive correlation between the number of their minor alleles and *COL4A1*/*COL4A2* expression. Similarly, the 18 eQTLs identified for both *COL4A5* and *COL4A6* were also supported by positive correlation between the number of their minor alleles and their expression. In particular, all the minor alleles at the 13 eQTLs were observed only in 1 individual who had the largest expression of *COL4A2*, and all the minor alleles at the 18 eQTLs were observed only in 1 individual who had the largest expression of *COL4A5*.

This study also revealed strong epistases between the eQTLs for the genes encoding type IV collagen. For example, individuals with “CC” genotype at rs4276755 showed increased expression of *COL4A2* by nucleotide substitution of rs55869773 from “GG” to “GA/AA” whereas individuals with “CT/TT” genotype at rs4276755 showed decreased the expression (*P* = 1.86 × 10^−237^; Supplementary Fig. S3). We attempted to examine several epistasis among 3 eQTLs and found them significant (data not shown), which implied other epistasis among more than 2 eQTLs. This suggests a variety of interactions among eQTLs in regulating the gene expression.

We suggested that multiple genes involved in the PI3K/AKT pathway were simultaneously expressed with associations of multiple eQTLs. Further studies are required to understand relationship among the genes simultaneously expressed in the current study and their specific roles and mechanisms in the PI3K/AKT pathway.

## Acknowledgments

The authors thank the Basic Science Research Program through the National Research Foundation of Korea, Ministry of Education, Science, and Technology (Grant No. NRF-2012M3A9D1054705) for the support.

## Supplementary Material

Supplemental Digital Content
