# Supplementary material for: Expression quantitative trait loci for PI3K/AKT pathway
Source: Medicine (Baltimore). 2017 Jan 10;96(1):e5817. doi: 10.1097/MD.0000000000005817 (PMC5228698; doi:10.1097/MD.0000000000005817)
Supplement: Supplemental Digital Content [file medi-96-e5817-s001.pdf]

Figure S1 Linkage disequilibrium (LD) blocks with nucleotide variants in proximity of association signals for gene expression of *COL4A1* and *COL4A2*. Pair-wise linkage disequilibrium estimate (r^2) is presented in brightness. Red box indicates nucleotide variant with the most significant association within each LD block. Cytogenic location (A) 4q32.3, (B) 6q13.1, (C) 9p24.1, (D) 9q31.2 and (E) 22q11.22.

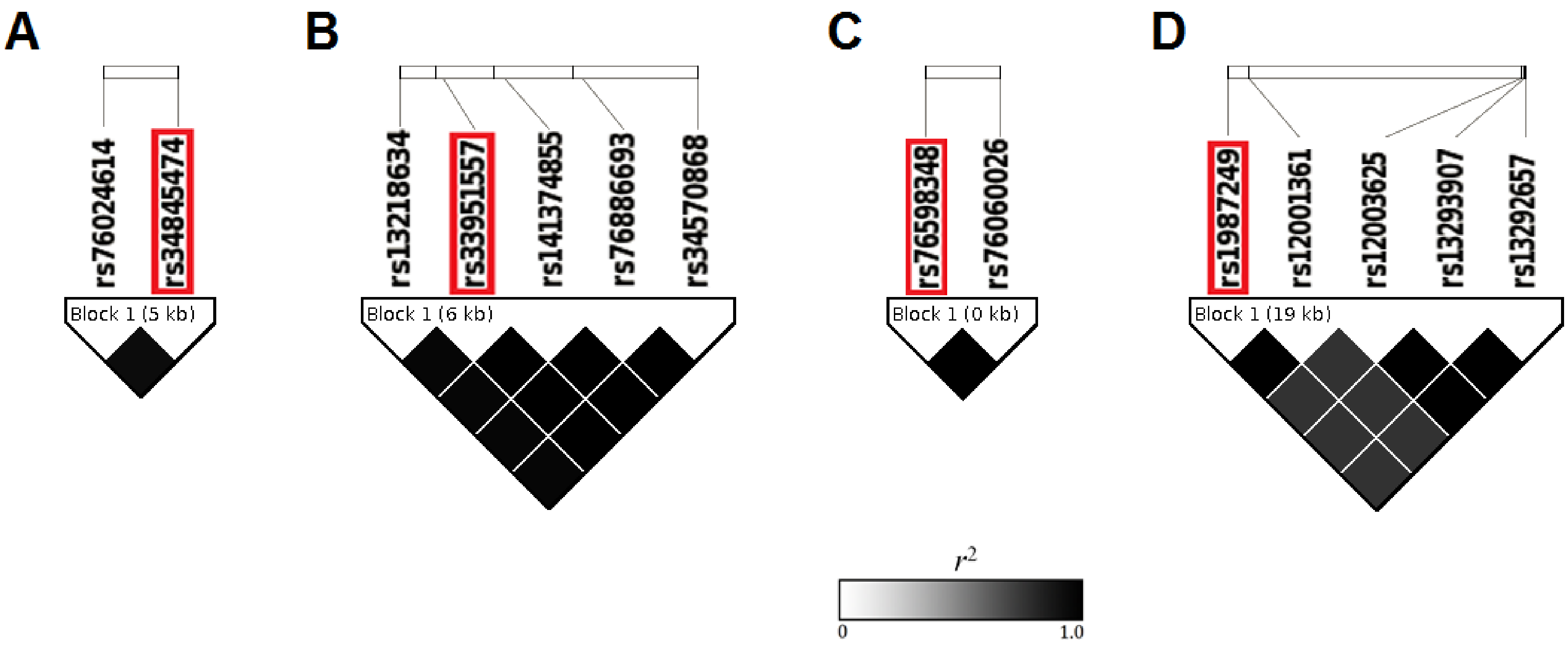

E

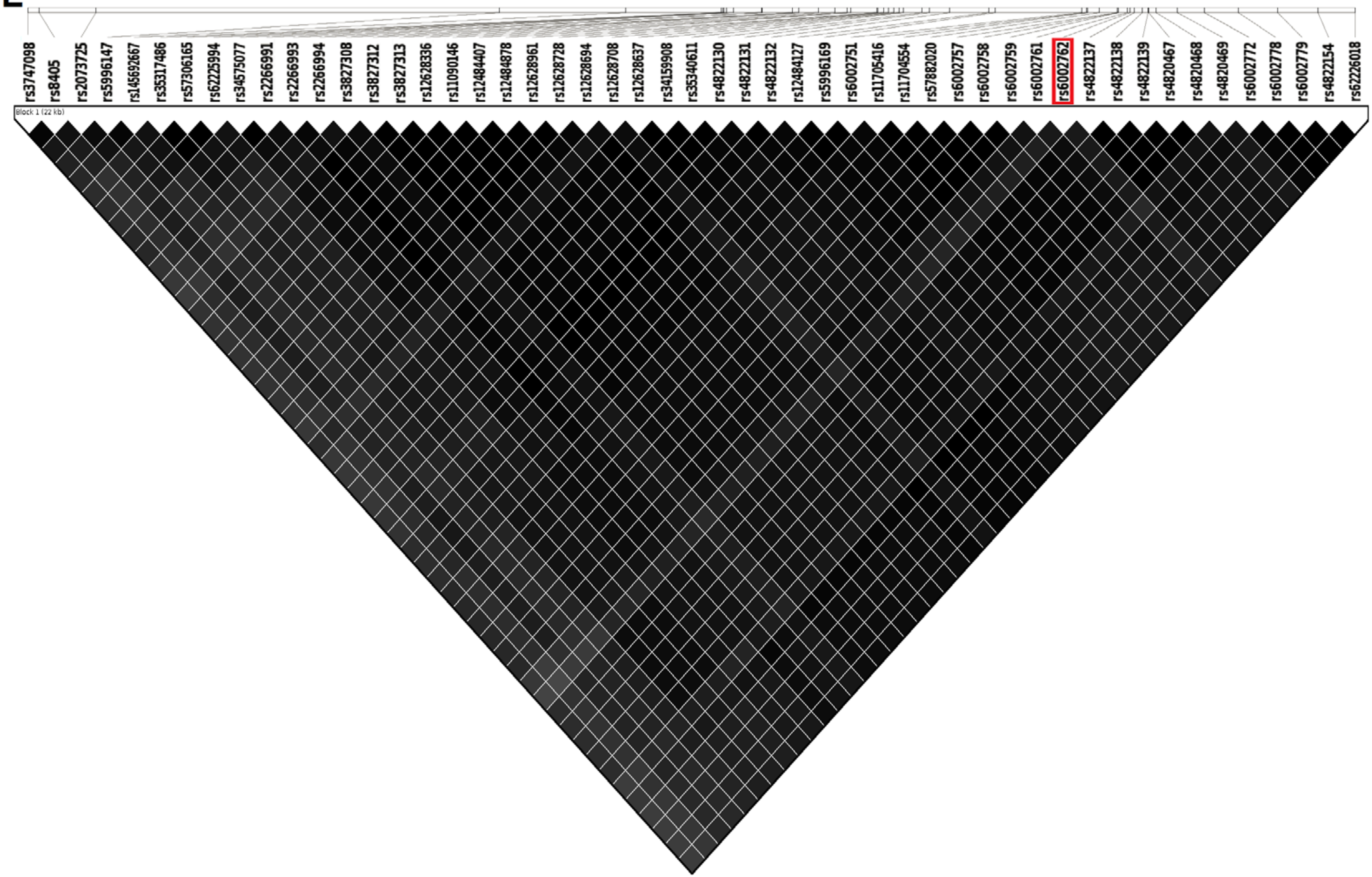
rs3747098
rs8405
rs2073725
rs5996147
rs145692667
rs35317486
rs57306165
rs62225994
rs34575077
rs2266991
rs2266993
rs2266994
rs3827308
rs3827312
rs3827313
rs12628336
rs11090146
rs12484407
rs12484878
rs12628961
rs12628728
rs12628694
rs12628708
rs12628637
rs34159908
rs35340611
rs4822130
rs4822131
rs4822132
rs12484127
rs5996169
rs6002751
rs11705416
rs11704554
rs57882020
rs6002757
rs6002758
rs6002759
rs6002761
rs6002762
rs4822137
rs4822138
rs4822139
rs4820467
rs4820468
rs4820469
rs6002772
rs6002778
rs6002779
rs4822154
rs62226018
Block 1 (22 kb)

Figure S2 Linkage disequilibrium (LD) blocks with nucleotide variants in proximity of association signals for gene expression of *COL4A5* and *COL4A6*. Pair-wise linkage disequilibrium estimate (r^2) is presented in brightness. Red box indicates nucleotide variant with the most significant association within each LD block. The LD block in (E) includes two variants in boxes: rs7964807 was the most significant variant associated with expression of *COL4A5* and rs189351960 was the most significant variant associated with expression of *COL4A6*. Cytogenic location (A) 2p25.2, (B) 7q32.2, (C) 5q32, (D) 16q21, and (E) 12q21.1.

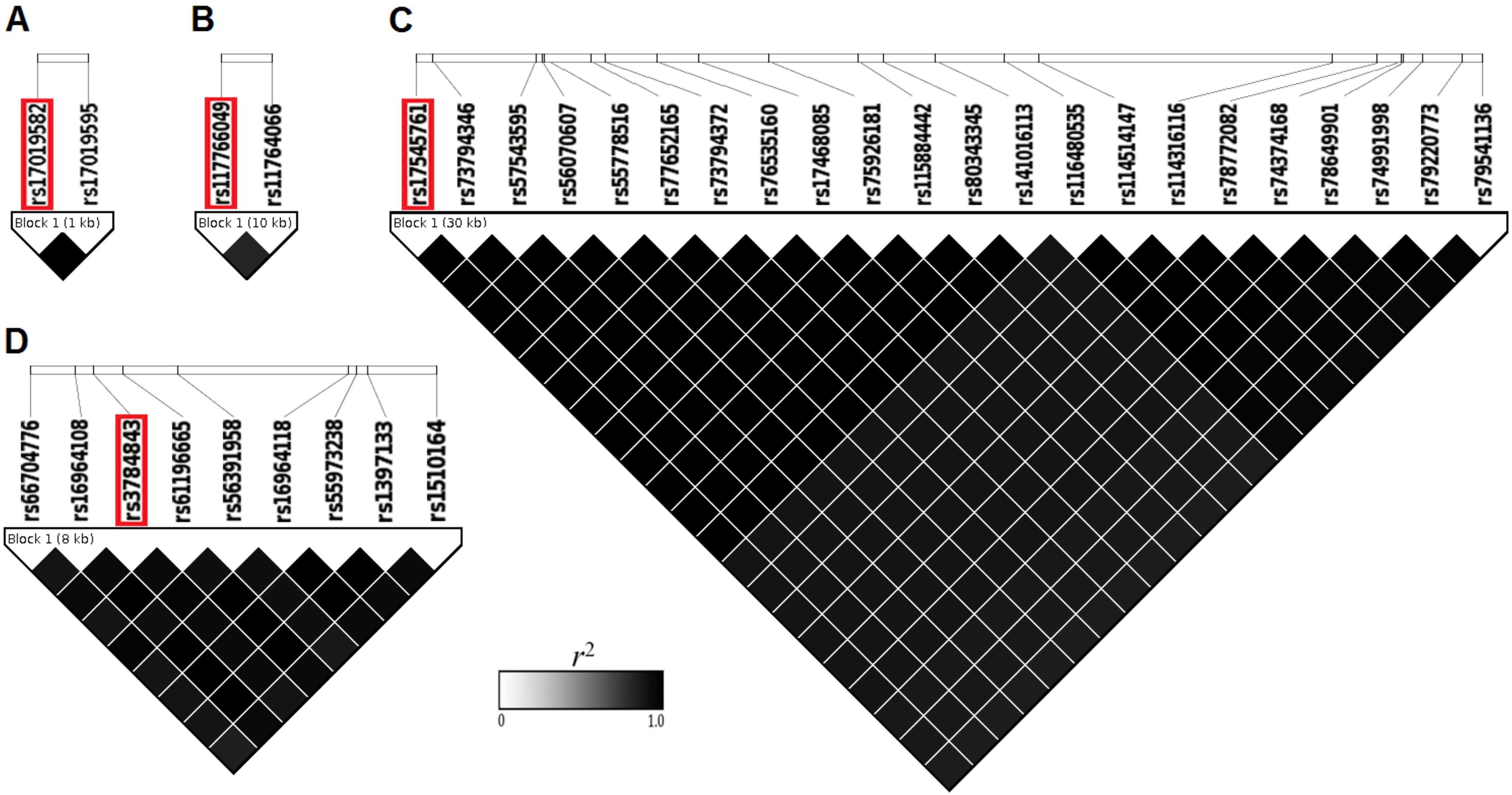

E

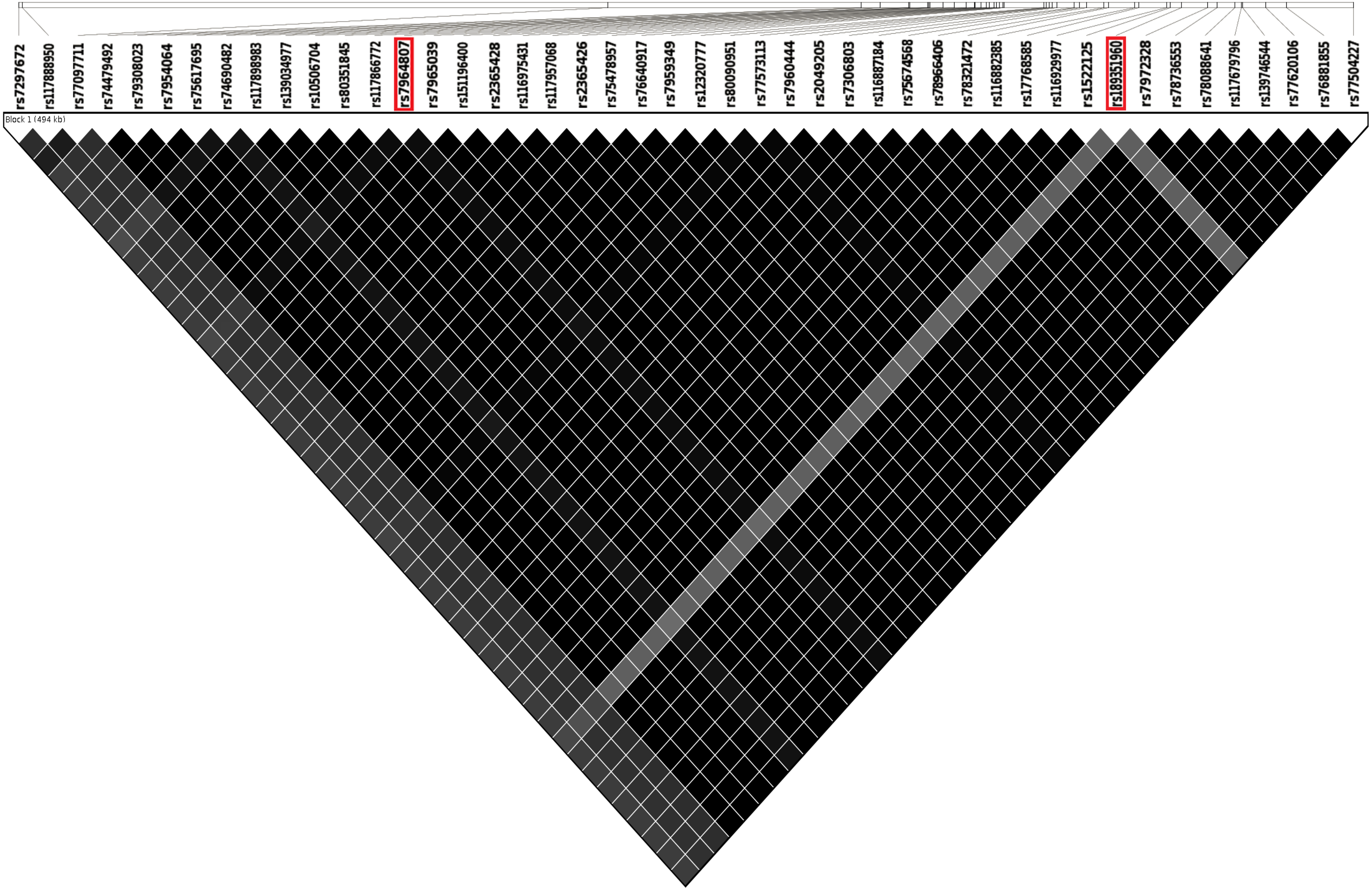
rs7297672
rs117888950
rs77097711
rs74479492
rs79308023
rs79540064
rs75617695
rs74690482
rs117898983
rs139034977
rs10506704
rs80351845
rs117866772
rs7964807
rs7965039
rs151196400
rs2365428
rs116975431
rs117957068
rs2365426
rs75478957
rs76640917
rs7959349
rs12320777
rs80090951
rs77573113
rs7960444
rs2049205
rs7306803
rs116887184
rs75674568
rs78966406
rs78321472
rs116882385
rs17768585
rs116929977
rs1522125
rs189351960
rs7972328
rs78736553
rs78088641
rs117679796
rs139746544
rs77620106
rs76881855
rs77504227
Block 1 (494 kb)

Figure S3 Epistasis between nucleotide variants associated with expression of *COL4A2* (A, B, C, and D), and *COL4A5* (E). Epistatic effects with $P < 1\times10^{-200}$ are presented. Value in each cell denotes mean estimate of RPKM for individuals with specific combined genotypes. A darker cell indicates a larger mean estimate.

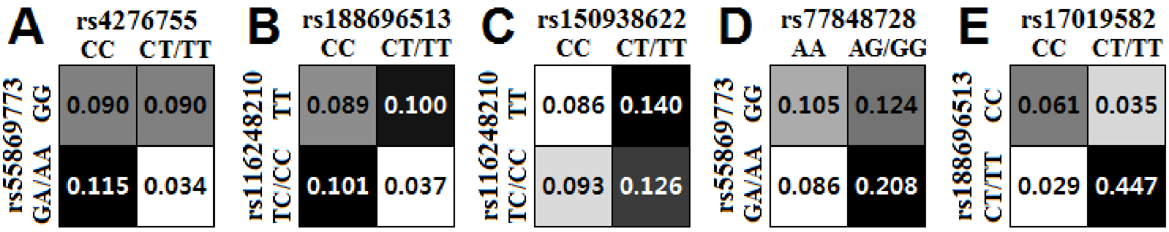

Table S1 Nucleotide variants associated with expression of the genes involved in the PI3K/AKT pathway by a genome-wide association study ($P<5\times10^{-8}$).

Expressed Gene	*SNV*					
	ID	*Chr*	*Position* [a]	*Gene*	β	P
ATF4	rs61519868	5	59,855,364	Intergenic	21.48	4.85×10^{-8}
	rs150915887	8	94,915,864	Intergenic	29.54	4.12×10^{-8}
	rs12873072	13	102,309,754	*ITGBL1*	29.49	2.34×10^{-8}
	rs139331134	14	20,442,676	Intergenic	29.83	2.86×10^{-9}
	rs10873097	14	20,446,556	Intergenic	29.20	3.59×10^{-9}
	rs113845735	14	20,454,230	Intergenic	29.83	2.86×10^{-9}
	rs112843116	14	20,455,388	Intergenic	26.99	3.87×10^{-8}
	rs117990005	14	20,455,906	Intergenic	29.83	2.86×10^{-9}
	rs72667632	14	20,461,839	Intergenic	29.83	2.86×10^{-9}
	rs72667634	14	20,464,780	Intergenic	29.83	2.86×10^{-9}
	rs72667636	14	20,467,477	Intergenic	29.83	2.86×10^{-9}
	rs72667637	14	20,468,104	Intergenic	29.83	2.86×10^{-9}
	rs72667638	14	20,468,749	Intergenic	29.83	2.86×10^{-9}
	rs72667639	14	20,469,029	Intergenic	29.83	2.86×10^{-9}
	rs72667641	14	20,469,971	Intergenic	29.83	2.86×10^{-9}
	rs72667643	14	20,471,786	Intergenic	29.83	2.86×10^{-9}
CCND1	rs187381186	7	2,662,189	Intergenic	9.76	1.41×10^{-8}
	rs11228599	11	69,026,286	Intergenic	7.40	3.98×10^{-8}
	rs7940107	11	69,027,770	Intergenic	7.59	2.78×10^{-8}
	rs7122516	11	69,029,009	Intergenic	7.59	2.78×10^{-8}
	rs7122786	11	69,029,210	Intergenic	7.59	2.78×10^{-8}
	rs11228600	11	69,029,424	Intergenic	7.59	2.78×10^{-8}
	rs11228602	11	69,029,827	Intergenic	7.59	2.78×10^{-8}
	rs72930263	11	69,030,206	Intergenic	7.59	2.78×10^{-8}
	rs72930267	11	69,030,990	Intergenic	7.59	2.78×10^{-8}
	rs74367143	11	69,031,273	Intergenic	7.59	2.78×10^{-8}
	rs12418263	11	69,031,698	Intergenic	7.59	2.78×10^{-8}
	rs10896454	11	69,033,016	Intergenic	7.59	2.78×10^{-8}
	rs10896455	11	69,035,337	Intergenic	7.59	2.78×10^{-8}
	rs10896456	11	69,035,585	Intergenic	7.59	2.78×10^{-8}
CDK6	rs28592627	15	31,327,398	*TRPM1*	1.69	1.08×10^{-8}
	rs75969010	15	31,329,753	*TRPM1*	2.30	2.26×10^{-9}
	rs28449259	15	31,331,353	*TRPM1*	1.62	3.28×10^{-8}
	rs28535333	15	31,334,684	*TRPM1*	1.64	2.29×10^{-8}
CDKN1A	rs75927735	15	86,169,103	*AKAP13*	23.45	3.59×10^{-8}
CHAD	rs62225420	22	34,167,980	*LARGE*	0.03	2.65×10^{-8}
	rs2267283	22	34,171,554	*LARGE*	0.03	2.65×10^{-8}
	rs2267284	22	34,172,783	*LARGE*	0.03	2.65×10^{-8}
	rs62225421	22	34,174,674	*LARGE*	0.03	2.65×10^{-8}
	rs62225423	22	34,174,927	*LARGE*	0.03	2.65×10^{-8}
	rs62225435	22	34,176,266	*LARGE*	0.03	2.65×10^{-8}
	rs74608905	22	34,176,823	*LARGE*	0.03	2.65×10^{-8}
	rs111819723	22	34,177,405	*LARGE*	0.03	2.65×10^{-8}
	rs117847225	22	34,178,895	*LARGE*	0.03	2.65×10^{-8}

	rs62225437	22	34,180,711	*LARGE*	0.03	2.65×10^{-8}
	rs62225438	22	34,180,801	*LARGE*	0.03	2.65×10^{-8}
	rs11912964	22	34,182,108	*LARGE*	0.03	2.65×10^{-8}
	rs78249327	22	34,182,849	*LARGE*	0.03	3.04×10^{-8}
	rs17735056	22	34,183,257	*LARGE*	0.03	2.65×10^{-8}
	rs62225440	22	34,184,095	*LARGE*	0.03	2.65×10^{-8}
	rs62225441	22	34,185,870	*LARGE*	0.03	2.65×10^{-8}
	rs62225442	22	34,186,187	*LARGE*	0.03	2.65×10^{-8}
	rs62225443	22	34,186,316	*LARGE*	0.03	2.65×10^{-8}
	rs11912570	22	34,189,746	*LARGE*	0.03	2.65×10^{-8}
	rs62225445	22	34,190,124	*LARGE*	0.03	2.65×10^{-8}
	rs62225447	22	34,190,793	*LARGE*	0.03	2.65×10^{-8}
	rs62225448	22	34,190,850	*LARGE*	0.03	2.39×10^{-8}
	rs113825065	22	34,191,496	*LARGE*	0.03	1.34×10^{-8}
	rs113950244	22	34,191,634	*LARGE*	0.03	2.65×10^{-8}
	rs2267287	22	34,192,621	*LARGE*	0.03	2.65×10^{-8}
	rs2267289	22	34,194,000	*LARGE*	0.03	2.65×10^{-8}
COL1A1	rs79442714	1	113,833,382	Intergenic	0.85	5.99×10^{-10}
	rs12756965	1	113,839,149	Intergenic	0.85	5.99×10^{-10}
	rs12045733	1	113,848,704	Intergenic	0.85	5.99×10^{-10}
	rs12038527	1	113,848,729	Intergenic	0.85	5.99×10^{-10}
	rs4839306	1	113,849,470	Intergenic	0.85	5.99×10^{-10}
	rs12048530	1	113,851,139	Intergenic	0.85	5.99×10^{-10}
	rs12046318	1	113,854,250	Intergenic	0.85	5.99×10^{-10}
	rs35059446	1	113,858,904	Intergenic	0.85	5.99×10^{-10}
	rs2226155	1	113,865,778	Intergenic	0.70	2.10×10^{-8}
	rs1361765	1	113,866,202	Intergenic	0.70	2.10×10^{-8}
	rs12024820	1	113,866,431	Intergenic	0.70	2.10×10^{-8}
	rs6674942	1	113,866,749	Intergenic	0.70	2.10×10^{-8}
	rs12025961	1	113,867,828	Intergenic	0.70	2.10×10^{-8}
	rs12047722	1	113,868,398	Intergenic	0.70	2.10×10^{-8}
	rs4838983	1	113,870,537	Intergenic	0.70	2.10×10^{-8}
	rs4839309	1	113,871,003	Intergenic	0.70	2.10×10^{-8}
	rs72779188	2	8,393,977	*LINC00299*	0.76	1.80×10^{-8}
	rs10490219	2	8,400,895	*LINC00299*	0.76	1.99×10^{-8}
	rs72779193	2	8,403,566	*LINC00299*	0.79	1.10×10^{-8}
	rs72779198	2	8,405,284	*LINC00299*	0.81	6.53×10^{-9}
	rs115336235	3	90,358,752	Intergenic	0.69	3.92×10^{-8}
	rs150483042	3	90,431,083	Intergenic	0.72	1.14×10^{-8}
	rs184655552	3	90,432,562	Intergenic	0.70	1.98×10^{-8}
	rs144677859	3	90,435,679	Intergenic	0.72	1.14×10^{-8}
	rs141940565	3	90,435,940	Intergenic	0.72	1.14×10^{-8}
	rs13315212	3	115,527,956	*LSAMP*	0.78	4.40×10^{-9}
	rs13315216	3	115,527,978	*LSAMP*	0.78	4.40×10^{-9}
	rs13325588	3	115,528,064	*LSAMP*	0.78	4.40×10^{-9}
	rs13128743	4	7,301,089	*SORCS2*	0.73	4.44×10^{-8}
	rs55932807	4	90,753,339	*SNCA*	0.77	2.89×10^{-9}
	rs192402944	4	129,628,719	Intergenic	0.71	2.08×10^{-8}
	rs184741912	4	129,628,720	Intergenic	0.71	2.08×10^{-8}

rs189724000	4	129,628,727	Intergenic	0.73	1.25×10^{-8}
rs13147782	4	131,034,260	Intergenic	0.93	1.33×10^{-10}
rs116135844	4	168,179,343	Intergenic	0.86	1.16×10^{-9}
rs11967504	6	52,521,611	Intergenic	0.77	1.72×10^{-9}
rs75882651	6	148,792,046	*SASH1*	0.72	3.10×10^{-8}
rs140793115	6	160,606,525	Intergenic	0.57	4.24×10^{-8}
rs73024503	6	162,326,020	*PARK2*	0.67	2.33×10^{-8}
rs116871569	7	101,731,567	*CUX1*	0.78	3.92×10^{-9}
rs17223858	8	74,698,803	*UBE2W*	0.71	1.25×10^{-8}
rs112981873	8	74,798,939	Intergenic	0.74	5.07×10^{-9}
rs7019175	9	18,522,594	*ADAMTSL1*	0.84	1.82×10^{-10}
rs62566425	9	67,310,474	Intergenic	0.75	8.78×10^{-9}
rs55861025	10	7,473,910	Intergenic	0.96	2.00×10^{-11}
rs12249425	10	131,930,990	Intergenic	0.73	3.89×10^{-8}
rs112346479	10	131,931,709	Intergenic	0.73	3.89×10^{-8}
rs11017097	10	131,933,983	Intergenic	0.73	3.89×10^{-8}
rs12259362	10	131,935,267	*GLRX3*	0.76	2.14×10^{-8}
rs12259552	10	131,935,484	*GLRX3*	0.75	1.70×10^{-8}
rs16910119	10	131,941,109	*GLRX3*	0.75	1.70×10^{-8}
rs59112182	10	131,941,320	*GLRX3*	0.75	1.70×10^{-8}
rs12241046	10	131,941,976	*GLRX3*	0.75	1.70×10^{-8}
rs73389584	10	131,944,335	*GLRX3*	0.75	1.70×10^{-8}
rs16910127	10	131,945,050	*GLRX3*	0.75	1.70×10^{-8}
rs12247627	10	131,945,715	*GLRX3*	0.75	1.70×10^{-8}
rs73389601	10	131,954,511	*GLRX3*	0.75	1.70×10^{-8}
rs12245251	10	131,958,985	*GLRX3*	0.75	1.70×10^{-8}
rs112831038	10	131,961,468	*GLRX3*	0.75	1.70×10^{-8}
rs113773648	10	131,961,510	*GLRX3*	0.76	2.14×10^{-8}
rs16910163	10	131,967,640	*GLRX3*	0.75	1.70×10^{-8}
rs73391530	10	131,968,213	*GLRX3*	0.75	1.70×10^{-8}
rs73391534	10	131,968,889	*GLRX3*	0.75	1.70×10^{-8}
rs16910167	10	131,970,613	*GLRX3*	0.75	1.70×10^{-8}
rs28548207	10	131,973,052	*GLRX3*	0.75	1.70×10^{-8}
rs12262460	10	131,973,632	*GLRX3*	0.75	1.70×10^{-8}
rs111905354	10	131,974,461	*GLRX3*	0.75	1.70×10^{-8}
rs77706663	10	131,985,265	Intergenic	0.75	1.70×10^{-8}
rs11017122	10	131,985,618	Intergenic	0.75	1.70×10^{-8}
rs11017125	10	131,988,165	Intergenic	0.76	2.14×10^{-8}
rs190065357	10	134,951,797	Intergenic	0.78	2.89×10^{-8}
rs77531197	11	22,373,423	*SLC17A6*	0.82	6.35×10^{-9}
rs67897587	12	13,293,153	Intergenic	0.94	1.09×10^{-11}
rs73054493	12	18,897,257	Intergenic	0.78	5.19×10^{-9}
rs73054495	12	18,897,688	Intergenic	0.78	5.19×10^{-9}
rs73054500	12	18,897,924	Intergenic	0.78	5.19×10^{-9}
rs11830000	12	18,898,022	Intergenic	0.77	4.81×10^{-9}
rs73056209	12	18,898,288	Intergenic	0.78	5.19×10^{-9}
rs17488409	12	18,899,969	Intergenic	0.76	9.28×10^{-9}
rs73056216	12	18,902,792	Intergenic	0.72	3.08×10^{-8}
rs140550132	12	124,415,437	*DNAH10*	0.74	3.00×10^{-8}

	rs181843847	12	133,107,327	*FBRSL1*	0.69	7.53×10^{-9}
	rs182087623	13	43,443,521	Intergenic	0.75	4.74×10^{-9}
	rs142713725	13	88,877,310	Intergenic	0.77	5.24×10^{-9}
	rs72658018	13	113,230,049	*TUBGCP3*	0.84	1.50×10^{-9}
	rs77851865	14	37,011,524	Intergenic	0.69	1.99×10^{-8}
	rs114444884	14	37,013,268	Intergenic	0.69	1.99×10^{-8}
	rs141785160	14	37,013,866	Intergenic	0.69	1.99×10^{-8}
	rs147896263	14	37,014,219	Intergenic	0.69	1.99×10^{-8}
	rs12433344	14	37,015,480	Intergenic	0.69	1.99×10^{-8}
	rs8012101	14	37,023,683	Intergenic	0.73	8.09×10^{-9}
	rs75202955	14	37,029,268	Intergenic	0.73	8.09×10^{-9}
	rs12433736	14	47,505,657	*MDGA2*	0.59	4.84×10^{-8}
	rs7156878	14	76,140,170	*TTLL5*	0.70	2.10×10^{-8}
	rs77439688	14	76,151,092	*TTLL5*	0.70	2.10×10^{-8}
	rs45614641	14	90,744,345	Intergenic	0.81	1.21×10^{-8}
	rs78466601	14	90,753,082	*NRDE2*	0.80	1.22×10^{-8}
	rs75782643	14	90,785,989	*NRDE2*	0.82	7.74×10^{-9}
	rs79684894	14	90,791,704	*NRDE2*	0.82	7.74×10^{-9}
	rs111350821	14	90,805,500	Intergenic	0.79	1.11×10^{-8}
	rs34561641	16	48,756,957	Intergenic	0.88	1.71×10^{-10}
	rs181029676	17	64,558,223	*PRKCA*	0.79	1.32×10^{-8}
	rs192073405	17	69,639,501	Intergenic	0.82	2.21×10^{-9}
	rs140544461	19	1,747,397	Intergenic	0.78	1.01×10^{-8}
	rs146734736	19	17,281,448	*MYO9B*	0.73	1.37×10^{-8}
	rs77904645	19	17,282,804	*MYO9B*	0.73	1.37×10^{-8}
	rs80243139	19	17,314,600	*MYO9B*	0.69	3.55×10^{-8}
	rs117115747	19	17,320,272	*MYO9B*	0.73	1.37×10^{-8}
	rs113461568	19	17,326,124	Intergenic	0.69	2.53×10^{-8}
	rs12971477	19	47,047,113	*PPP5D1*	0.65	1.09×10^{-8}
	rs77494829	20	6,778,465	Intergenic	0.71	3.05×10^{-8}
	rs76537270	20	6,782,025	Intergenic	0.76	6.21×10^{-9}
	rs117388418	20	61,279,131	*SLCO4A1*	0.70	2.94×10^{-8}
	rs34419428	20	61,288,015	*SLCO4A1*	0.83	8.95×10^{-10}
	rs6006621	22	44,419,407	*PARVB*	0.77	4.86×10^{-8}
COL1A2	rs3763469	7	94,021,475	Intergenic	0.03	2.73×10^{-17}
	rs17166249	7	94,036,547	*COL1A2*	0.02	2.48×10^{-8}
	rs2521206	7	94,039,187	*COL1A2*	0.02	4.64×10^{-12}
	rs2621208	7	94,039,410	*COL1A2*	0.02	4.64×10^{-12}
	rs421587	7	94,043,274	*COL1A2*	0.02	2.50×10^{-9}
	rs2250365	7	94,044,803	*COL1A2*	0.02	2.50×10^{-9}
	rs2621212	7	94,045,166	*COL1A2*	0.02	1.72×10^{-9}
	rs2621213	7	94,046,468	*COL1A2*	0.02	2.38×10^{-9}
	rs2023729	7	94,048,165	*COL1A2*	0.02	5.91×10^{-9}
	rs77964799	7	94,048,229	*COL1A2*	0.02	5.91×10^{-9}
	rs62464629	7	94,052,603	*COL1A2*	0.02	6.89×10^{-10}
	rs41317734	7	94,054,194	*COL1A2*	0.02	6.89×10^{-10}
	rs62464631	7	94,055,885	*COL1A2*	0.02	2.14×10^{-10}
	rs7804898	7	94,056,099	*COL1A2*	0.02	6.89×10^{-10}
	rs7805430	7	94,056,216	*COL1A2*	0.02	6.89×10^{-10}

	rs1800248	7	94,056,349	*COL1A2*	0.02	6.89×10^{-10}
	rs59226272	7	94,058,198	*COL1A2*	0.02	1.80×10^{-8}
	rs11179009	12	72,356,955	*TPH2*	0.02	8.78×10^{-9}
	rs11179011	12	72,357,672	*TPH2*	0.02	8.99×10^{-9}
COL4A1	rs149085004	1	1,963,089	Intergenic	0.11	6.71×10^{-9}
	rs79466073	1	50,569,011	*ELAVL4*	0.11	1.51×10^{-8}
	rs116248210	2	103,945,833	Intergenic	0.12	8.79×10^{-10}
	rs116506968	2	183,551,471	Intergenic	0.11	1.13×10^{-10}
	rs191368451	3	165,841,347	Intergenic	0.12	3.25×10^{-8}
	rs76024614	4	169,745,500	*PALLD*	0.11	1.72×10^{-8}
	rs34845474	4	169,751,339	*PALLD*	0.12	7.82×10^{-9}
	rs13218634	6	81,392,645	Intergenic	0.11	3.88×10^{-8}
	rs33951557	6	81,393,482	Intergenic	0.12	2.79×10^{-8}
	rs141374855	6	81,394,818	Intergenic	0.12	2.79×10^{-8}
	rs76886693	6	81,396,607	Intergenic	0.12	2.79×10^{-8}
	rs34570868	6	81,399,464	Intergenic	0.12	2.79×10^{-8}
	rs55869773	7	4,498,947	Intergenic	0.11	4.27×10^{-8}
	rs78256678	7	140,156,723	*MKRN1*	0.08	2.96×10^{-8}
	rs76598348	9	7,568,139	Intergenic	0.11	3.95×10^{-9}
	rs76060026	9	7,568,568	Intergenic	0.11	3.95×10^{-9}
	rs1987249	9	108,683,134	Intergenic	0.11	1.26×10^{-8}
	rs12001361	9	108,684,545	Intergenic	0.11	1.26×10^{-8}
	rs4276755	9	124,377,411	*DAB2IP*	0.11	3.10×10^{-8}
	rs3898867	12	9,461,001	*LOC642846*	0.08	4.15×10^{-8}
	rs116674084	12	61,420,772	Intergenic	0.10	4.36×10^{-8}
	rs182060879	12	85,650,851	Intergenic	0.12	5.21×10^{-9}
	rs77320233	14	41,176,356	Intergenic	0.10	2.51×10^{-8}
	rs58877020	14	95,162,140	Intergenic	0.10	1.89×10^{-8}
	rs112258457	14	95,162,842	Intergenic	0.10	2.83×10^{-8}
	rs112946166	14	95,165,113	Intergenic	0.10	2.83×10^{-8}
	rs4396507	15	32,938,761	*SCG5*	0.12	2.75×10^{-8}
	rs11867392	17	1,921,816	*RTN4RL1*	0.10	6.45×10^{-9}
	rs11868558	17	6,550,543	*MED31*	0.14	8.34×10^{-11}
	rs77341824	18	6,474,441	Intergenic	0.09	4.45×10^{-8}
	rs3747098	22	22,890,066	Intergenic	0.11	9.37×10^{-9}
	rs8405	22	22,890,270	*PRAME*	0.10	3.27×10^{-8}
	rs2073725	22	22,891,308	*PRAME*	0.11	2.01×10^{-8}
	rs5996147	22	22,898,675	*PRAME*	0.11	1.52×10^{-8}
	rs145692667	22	22,900,984	*PRAME*	0.11	8.09×10^{-9}
	rs35317486	22	22,902,728	*LOC648691*	0.12	3.05×10^{-9}
	rs57306165	22	22,902,759	*LOC648691*	0.12	3.05×10^{-9}
	rs62225994	22	22,902,783	*LOC648691*	0.12	3.05×10^{-9}
	rs34575077	22	22,902,829	*LOC648691*	0.12	4.02×10^{-9}
	rs2266991	22	22,902,952	*LOC648691*	0.12	4.02×10^{-9}
	rs2266993	22	22,903,465	*LOC648691*	0.11	1.11×10^{-8}
	rs2266994	22	22,903,480	*LOC648691*	0.11	1.88×10^{-8}
	rs3827308	22	22,904,030	*LOC648691*	0.11	1.39×10^{-8}
	rs3827312	22	22,904,143	*LOC648691*	0.11	1.11×10^{-8}
	rs3827313	22	22,904,150	*LOC648691*	0.11	1.11×10^{-8}

	rs12628336	22	22,904,506	*LOC648691*	0.11	1.11×10^{-8}
	rs11090146	22	22,904,802	*LOC648691*	0.11	1.11×10^{-8}
	rs12484407	22	22,905,030	*LOC648691*	0.11	1.11×10^{-8}
	rs12484878	22	22,905,095	*LOC648691*	0.11	1.11×10^{-8}
	rs12628961	22	22,905,574	*LOC648691*	0.11	1.11×10^{-8}
	rs12628728	22	22,905,592	*LOC648691*	0.11	1.11×10^{-8}
	rs12628694	22	22,905,693	*LOC648691*	0.11	7.13×10^{-9}
	rs12628708	22	22,905,786	*LOC648691*	0.11	1.19×10^{-8}
	rs12628637	22	22,905,883	*LOC648691*	0.11	7.13×10^{-9}
	rs34159908	22	22,905,979	*LOC648691*	0.11	7.13×10^{-9}
	rs35340611	22	22,906,060	*LOC648691*	0.11	7.13×10^{-9}
	rs4822130	22	22,906,391	*LOC648691*	0.11	7.13×10^{-9}
	rs4822131	22	22,906,529	*LOC648691*	0.11	1.11×10^{-8}
	rs4822132	22	22,906,901	*LOC648691*	0.11	1.11×10^{-8}
	rs12484127	22	22,907,618	*LOC648691*	0.11	1.12×10^{-8}
	rs5996169	22	22,907,730	*LOC648691*	0.11	1.12×10^{-8}
	rs6002751	22	22,909,312	Intergenic	0.11	1.12×10^{-8}
	rs11705416	22	22,909,404	Intergenic	0.12	2.75×10^{-9}
	rs11704554	22	22,909,424	Intergenic	0.12	2.75×10^{-9}
	rs57882020	22	22,909,636	Intergenic	0.12	2.75×10^{-9}
	rs6002757	22	22,909,963	Intergenic	0.12	2.75×10^{-9}
	rs6002758	22	22,909,981	Intergenic	0.12	2.75×10^{-9}
	rs6002759	22	22,909,983	Intergenic	0.12	2.75×10^{-9}
	rs6002761	22	22,910,145	Intergenic	0.12	4.02×10^{-9}
	rs6002762	22	22,910,191	Intergenic	0.13	9.86×10^{-10}
	rs4822137	22	22,910,273	Intergenic	0.12	1.68×10^{-9}
	rs4822138	22	22,910,416	Intergenic	0.12	1.68×10^{-9}
	rs4822139	22	22,910,523	Intergenic	0.12	1.68×10^{-9}
	rs4820467	22	22,910,531	Intergenic	0.12	1.68×10^{-9}
	rs4820468	22	22,910,674	Intergenic	0.12	1.68×10^{-9}
	rs4820469	22	22,911,060	Intergenic	0.11	1.84×10^{-8}
	rs6002772	22	22,911,549	Intergenic	0.11	7.13×10^{-9}
	rs6002778	22	22,912,168	Intergenic	0.11	1.10×10^{-8}
	rs6002779	22	22,912,890	Intergenic	0.11	1.10×10^{-8}
	rs4822154	22	22,913,629	Intergenic	0.11	1.10×10^{-8}
	rs62226018	22	22,914,300	Intergenic	0.11	1.10×10^{-8}
	rs74617949	22	27,681,626	Intergenic	0.11	4.09×10^{-9}
COL4A2	rs3007418	1	6,526,633	*PLEKHG5*	0.32	1.29×10^{-8}
	rs74595111	1	9,117,948	*SLC2A5*	0.28	1.65×10^{-8}
	rs3908530	1	82,943,550	Intergenic	0.31	2.61×10^{-8}
	rs12563144	1	82,944,058	Intergenic	0.31	2.61×10^{-8}
	rs12022387	1	82,946,962	Intergenic	0.31	2.61×10^{-8}
	rs12023491	1	82,948,454	Intergenic	0.31	2.61×10^{-8}
	rs75406969	1	82,953,386	Intergenic	0.31	2.61×10^{-8}
	rs12076363	1	82,957,788	Intergenic	0.31	2.61×10^{-8}
	rs11163485	1	82,958,934	Intergenic	0.30	3.48×10^{-8}
	rs11163486	1	82,958,955	Intergenic	0.31	2.61×10^{-8}
	rs17108426	1	82,960,175	Intergenic	0.31	2.61×10^{-8}
	rs71652641	1	83,942,161	Intergenic	0.31	7.85×10^{-9}

rs148799277	1	83,943,833	Intergenic	0.32	$3.87{\times}10^{-9}$
rs114094232	1	83,947,011	Intergenic	0.32	$3.87{\times}10^{-9}$
rs144281778	1	83,949,995	Intergenic	0.32	$3.87{\times}10^{-9}$
rs183214628	1	83,950,958	Intergenic	0.32	$3.87{\times}10^{-9}$
rs146985494	1	83,952,773	Intergenic	0.32	$3.87{\times}10^{-9}$
rs17129518	1	83,957,067	Intergenic	0.32	$3.87{\times}10^{-9}$
rs17129962	1	83,962,407	Intergenic	0.32	$3.87{\times}10^{-9}$
rs9427710	1	200,656,391	*LOC101929224*	0.31	$9.89{\times}10^{-9}$
rs72810483	2	47,464,847	*LOC101927043*	0.29	$3.07{\times}10^{-8}$
rs116248210	2	103,945,833	Intergenic	0.34	$1.49{\times}10^{-10}$
rs140099187	2	115,060,095	Intergenic	0.31	$1.16{\times}10^{-8}$
rs12467531	2	115,150,766	Intergenic	0.33	$4.96{\times}10^{-9}$
rs113126958	2	134,065,882	*MIR7853; NCKAP5*	0.26	$3.64{\times}10^{-8}$
rs116506968	2	183,551,471	Intergenic	0.26	$2.96{\times}10^{-9}$
rs3732667	3	13,612,020	*FBLN2*	0.28	$2.36{\times}10^{-8}$
rs62311710	4	83,992,416	*COPS4*	0.31	$3.47{\times}10^{-8}$
rs143921144	4	164,979,336	*MARCH1*	0.33	$2.75{\times}10^{-9}$
rs139975382	4	164,979,337	*MARCH1*	0.33	$2.75{\times}10^{-9}$
rs112705949	4	165,101,542	*MARCH1*	0.29	$4.21{\times}10^{-8}$
rs76024614	4	169,745,500	*PALLD*	0.32	$2.56{\times}10^{-9}$
rs34845474	4	169,751,339	*PALLD*	0.34	$9.32{\times}10^{-10}$
rs190329732	5	178,839,347	Intergenic	0.30	$4.14{\times}10^{-9}$
rs13216553	6	49,863,679	Intergenic	0.24	$1.55{\times}10^{-8}$
rs34634780	6	49,864,066	Intergenic	0.24	$1.55{\times}10^{-8}$
rs35960031	6	49,866,346	Intergenic	0.24	$2.06{\times}10^{-8}$
rs13198541	6	49,866,519	Intergenic	0.25	$1.14{\times}10^{-8}$
rs13220185	6	49,871,623	Intergenic	0.24	$1.88{\times}10^{-8}$
rs71546680	6	49,871,833	Intergenic	0.24	$1.88{\times}10^{-8}$
rs13193597	6	49,872,143	Intergenic	0.24	$1.88{\times}10^{-8}$
rs13209755	6	49,872,219	Intergenic	0.24	$1.88{\times}10^{-8}$
rs35833551	6	73,989,046	*C6orf147; KHDC1*	0.32	$2.95{\times}10^{-8}$
rs34353921	6	73,994,478	*C6orf147; KHDC1*	0.33	$1.34{\times}10^{-8}$
rs13218634	6	81,392,645	Intergenic	0.33	$2.77{\times}10^{-9}$
rs33951557	6	81,393,482	Intergenic	0.34	$1.51{\times}10^{-9}$
rs141374855	6	81,394,818	Intergenic	0.34	$1.51{\times}10^{-9}$
rs76886693	6	81,396,607	Intergenic	0.34	$1.51{\times}10^{-9}$
rs34570868	6	81,399,464	Intergenic	0.34	$1.51{\times}10^{-9}$
rs55869773	7	4,498,947	Intergenic	0.32	$2.84{\times}10^{-9}$
rs11773071	7	46,988,659	Intergenic	0.31	$1.81{\times}10^{-8}$
rs75097405	7	47,016,706	Intergenic	0.30	$1.77{\times}10^{-8}$
rs143149138	7	75,703,521	Intergenic	0.29	$3.09{\times}10^{-8}$
rs78438375	7	114,409,885	Intergenic	0.32	$8.02{\times}10^{-9}$
rs112971957	8	20,123,575	Intergenic	0.31	$2.47{\times}10^{-8}$
rs67109150	8	20,128,497	Intergenic	0.32	$2.04{\times}10^{-8}$
rs73607971	8	20,129,346	Intergenic	0.32	$2.04{\times}10^{-8}$
rs67777401	8	20,129,780	Intergenic	0.32	$2.04{\times}10^{-8}$
rs56268521	8	20,130,102	Intergenic	0.32	$2.09{\times}10^{-8}$
rs4922145	8	20,130,379	Intergenic	0.33	$1.43{\times}10^{-8}$
rs4922146	8	20,130,816	Intergenic	0.33	$1.43{\times}10^{-8}$

rs66782430	8	20,131,952	Intergenic	0.33	1.43×10^{-8}
rs185794438	8	40,231,575	Intergenic	0.31	3.91×10^{-9}
rs17726591	8	97,650,119	Intergenic	0.32	1.40×10^{-8}
rs34547819	8	115,982,486	Intergenic	0.35	7.48×10^{-10}
rs4909903	8	136,241,420	Intergenic	0.29	4.90×10^{-8}
rs76598348	9	7,568,139	Intergenic	0.27	2.37×10^{-8}
rs76060026	9	7,568,568	Intergenic	0.27	2.37×10^{-8}
rs151151023	9	32,101,765	Intergenic	0.28	4.01×10^{-8}
rs139805874	9	32,132,547	Intergenic	0.29	2.74×10^{-8}
rs10816327	9	108,544,458	Intergenic	0.27	4.38×10^{-8}
rs10123013	9	108,547,625	Intergenic	0.32	3.86×10^{-9}
rs7869341	9	108,620,328	Intergenic	0.33	8.95×10^{-10}
rs10978312	9	108,623,184	Intergenic	0.33	8.95×10^{-10}
rs10816350	9	108,624,160	Intergenic	0.33	8.95×10^{-10}
rs1806664	9	108,626,502	Intergenic	0.37	1.81×10^{-10}
rs9299115	9	108,627,769	Intergenic	0.27	1.88×10^{-8}
rs4742963	9	108,631,025	Intergenic	0.26	4.86×10^{-8}
rs10978329	9	108,642,814	Intergenic	0.26	4.86×10^{-8}
rs35457712	9	108,644,271	Intergenic	0.26	4.86×10^{-8}
rs13297669	9	108,647,398	Intergenic	0.26	4.86×10^{-8}
rs13301197	9	108,647,882	Intergenic	0.26	4.86×10^{-8}
rs1987249	9	108,683,134	Intergenic	0.32	2.07×10^{-9}
rs12001361	9	108,684,545	Intergenic	0.32	2.07×10^{-9}
rs12003625	9	108,702,802	Intergenic	0.28	3.80×10^{-8}
rs13293907	9	108,702,986	Intergenic	0.28	3.80×10^{-8}
rs13292657	9	108,703,075	Intergenic	0.28	3.80×10^{-8}
rs34778810	9	108,830,793	Intergenic	0.34	5.41×10^{-9}
rs13299446	9	108,838,212	Intergenic	0.30	3.45×10^{-8}
rs62575314	9	108,839,612	Intergenic	0.30	3.45×10^{-8}
rs13289129	9	108,842,947	Intergenic	0.30	3.45×10^{-8}
rs115877227	9	108,849,934	Intergenic	0.30	3.45×10^{-8}
rs62575316	9	108,850,692	Intergenic	0.30	3.45×10^{-8}
rs62575317	9	108,851,032	Intergenic	0.30	3.45×10^{-8}
rs10978410	9	108,851,348	Intergenic	0.30	3.45×10^{-8}
rs10978412	9	108,851,598	Intergenic	0.30	3.45×10^{-8}
rs1154711	9	108,852,297	Intergenic	0.30	3.45×10^{-8}
rs34057980	9	108,852,359	Intergenic	0.35	1.57×10^{-9}
rs12350203	9	108,855,329	Intergenic	0.30	3.45×10^{-8}
rs13298859	9	108,856,907	Intergenic	0.30	3.45×10^{-8}
rs35649358	9	108,857,737	Intergenic	0.30	3.45×10^{-8}
rs13301778	9	108,859,326	Intergenic	0.30	3.45×10^{-8}
rs13285819	9	108,860,073	Intergenic	0.30	3.45×10^{-8}
rs13285861	9	108,860,215	Intergenic	0.30	3.45×10^{-8}
rs36037477	9	108,877,583	Intergenic	0.32	8.69×10^{-9}
rs4276755	9	124,377,411	*DAB2IP*	0.33	2.99×10^{-10}
rs72770225	9	132,454,730	*PRRX2*	0.32	2.38×10^{-8}
rs1472187	10	105,433,192	*SH3PXD2A*	0.33	1.97×10^{-9}
rs61752934	11	44,956,459	*TP53I11*	0.36	1.89×10^{-10}
rs9666102	11	58,363,293	*ZFP91; ZFP91-CNTF*	0.34	8.38×10^{-10}

rs78149287	12	26,596,890	*ITPR2*	0.33	3.72×10^{-9}
rs116674084	12	61,420,772	Intergenic	0.27	4.18×10^{-8}
rs182060879	12	85,650,851	Intergenic	0.34	1.11×10^{-10}
rs4767652	12	118,467,133	*RFC5*	0.30	4.47×10^{-8}
rs9581495	13	26,527,621	*ATP8A2*	0.29	3.85×10^{-8}
rs61951493	13	64,861,473	Intergenic	0.31	4.82×10^{-8}
rs17260562	14	90,407,322	*EFCAB11*	0.31	9.29×10^{-9}
rs146704566	14	90,410,653	*EFCAB11*	0.31	9.29×10^{-9}
rs12232164	14	90,417,508	*EFCAB11*	0.31	9.29×10^{-9}
rs4143999	14	90,421,493	Intergenic	0.31	2.78×10^{-9}
rs3759710	14	90,421,558	Intergenic	0.31	2.78×10^{-9}
rs2181599	14	90,422,211	Intergenic	0.31	9.29×10^{-9}
rs28736874	14	90,422,382	*TDP1*	0.32	1.62×10^{-9}
rs34348197	14	90,423,131	*TDP1*	0.31	9.29×10^{-9}
rs10498624	14	90,465,624	*TDP1*	0.31	9.29×10^{-9}
rs75312599	14	90,482,549	*TDP1*	0.31	8.39×10^{-9}
rs11159945	14	90,496,548	*TDP1*	0.29	2.33×10^{-8}
rs36045057	14	90,508,101	*TDP1*	0.29	2.33×10^{-8}
rs115371217	16	83,186,860	*CDH13*	0.30	4.52×10^{-8}
rs78423309	16	83,193,188	*CDH13*	0.30	1.11×10^{-8}
rs145590108	16	89,641,624	Intergenic	0.31	7.77×10^{-9}
rs11867392	17	1,921,816	*RTN4RL1*	0.28	2.14×10^{-9}
rs75551527	17	12,142,282	Intergenic	0.31	1.41×10^{-8}
rs6501759	17	66,805,247	Intergenic	0.30	2.78×10^{-10}
rs34552950	17	79,775,191	Intergenic	0.32	1.47×10^{-9}
rs117726027	18	27,204,592	Intergenic	0.34	2.56×10^{-9}
rs2825712	21	21,023,654	Intergenic	0.25	2.59×10^{-8}
rs3747098	22	22,890,066	Intergenic	0.30	1.34×10^{-9}
rs8405	22	22,890,270	*PRAME*	0.29	5.06×10^{-9}
rs2073725	22	22,891,308	*PRAME*	0.30	3.13×10^{-9}
rs5996147	22	22,898,675	*PRAME*	0.30	2.36×10^{-9}
rs145692667	22	22,900,984	*PRAME*	0.31	1.35×10^{-9}
rs35317486	22	22,902,728	*LOC648691*	0.32	4.05×10^{-10}
rs57306165	22	22,902,759	*LOC648691*	0.32	4.05×10^{-10}
rs62225994	22	22,902,783	*LOC648691*	0.32	4.05×10^{-10}
rs34575077	22	22,902,829	*LOC648691*	0.33	3.62×10^{-10}
rs2266991	22	22,902,952	*LOC648691*	0.33	3.62×10^{-10}
rs2266993	22	22,903,465	*LOC648691*	0.31	1.45×10^{-9}
rs2266994	22	22,903,480	*LOC648691*	0.30	2.61×10^{-9}
rs3827308	22	22,904,030	*LOC648691*	0.30	2.36×10^{-9}
rs3827312	22	22,904,143	*LOC648691*	0.31	1.45×10^{-9}
rs3827313	22	22,904,150	*LOC648691*	0.31	1.45×10^{-9}
rs12628336	22	22,904,506	*LOC648691*	0.31	1.45×10^{-9}
rs11090146	22	22,904,802	*LOC648691*	0.31	1.45×10^{-9}
rs12484407	22	22,905,030	*LOC648691*	0.31	1.45×10^{-9}
rs12484878	22	22,905,095	*LOC648691*	0.31	1.45×10^{-9}
rs12628961	22	22,905,574	*LOC648691*	0.31	1.45×10^{-9}
rs12628728	22	22,905,592	*LOC648691*	0.31	1.45×10^{-9}
rs12628694	22	22,905,693	*LOC648691*	0.31	9.63×10^{-10}

	rs12628708	22	22,905,786	*LOC648691*	0.30	1.69×10^{-9}
	rs12628637	22	22,905,883	*LOC648691*	0.31	9.63×10^{-10}
	rs34159908	22	22,905,979	*LOC648691*	0.31	9.63×10^{-10}
	rs35340611	22	22,906,060	*LOC648691*	0.31	9.63×10^{-10}
	rs4822130	22	22,906,391	*LOC648691*	0.31	9.63×10^{-10}
	rs4822131	22	22,906,529	*LOC648691*	0.31	1.45×10^{-9}
	rs4822132	22	22,906,901	*LOC648691*	0.31	1.45×10^{-9}
	rs12484127	22	22,907,618	*LOC648691*	0.30	2.10×10^{-9}
	rs5996169	22	22,907,730	*LOC648691*	0.30	2.10×10^{-9}
	rs6002751	22	22,909,312	Intergenic	0.30	2.10×10^{-9}
	rs11705416	22	22,909,404	Intergenic	0.32	4.80×10^{-10}
	rs11704554	22	22,909,424	Intergenic	0.32	4.80×10^{-10}
	rs57882020	22	22,909,636	Intergenic	0.32	4.80×10^{-10}
	rs6002757	22	22,909,963	Intergenic	0.32	4.80×10^{-10}
	rs6002758	22	22,909,981	Intergenic	0.32	4.80×10^{-10}
	rs6002759	22	22,909,983	Intergenic	0.32	4.80×10^{-10}
	rs6002761	22	22,910,145	Intergenic	0.33	4.74×10^{-10}
	rs6002762	22	22,910,191	Intergenic	0.35	7.68×10^{-11}
	rs4822137	22	22,910,273	Intergenic	0.33	3.08×10^{-10}
	rs4822138	22	22,910,416	Intergenic	0.33	3.08×10^{-10}
	rs4822139	22	22,910,523	Intergenic	0.33	3.08×10^{-10}
	rs4820467	22	22,910,531	Intergenic	0.33	3.08×10^{-10}
	rs4820468	22	22,910,674	Intergenic	0.33	3.08×10^{-10}
	rs4820469	22	22,911,060	Intergenic	0.29	4.29×10^{-9}
	rs6002772	22	22,911,549	Intergenic	0.31	9.63×10^{-10}
	rs6002778	22	22,912,168	Intergenic	0.30	1.84×10^{-9}
	rs6002779	22	22,912,890	Intergenic	0.30	1.84×10^{-9}
	rs4822154	22	22,913,629	Intergenic	0.30	1.84×10^{-9}
	rs62226018	22	22,914,300	Intergenic	0.30	1.84×10^{-9}
	rs74617949	22	27,681,626	Intergenic	0.30	2.14×10^{-9}
COL4A5	rs12759318	1	22,308,435	*CELA3B*	0.35	4.20×10^{-10}
	rs2506992	1	48,097,805	Intergenic	0.39	1.10×10^{-10}
	rs145840510	1	147,832,715	*NBPF8*	0.36	2.38×10^{-10}
	rs78710246	1	170,641,733	*PRRX1*	0.31	2.83×10^{-10}
	rs141448914	1	170,749,621	Intergenic	0.28	2.27×10^{-9}
	rs12563492	1	242,909,082	Intergenic	0.32	4.03×10^{-8}
	rs17019582	2	4,702,434	*LINC01249*	0.43	1.25×10^{-12}
	rs17019595	2	4,704,141	Intergenic	0.43	1.25×10^{-12}
	rs114677142	2	131,059,340	Intergenic	0.35	1.74×10^{-10}
	rs7566184	2	158,806,438	Intergenic	0.30	1.60×10^{-9}
	rs10168705	2	158,806,808	Intergenic	0.30	6.66×10^{-9}
	rs10203893	2	158,806,911	Intergenic	0.29	1.08×10^{-8}
	rs13405535	2	158,808,807	Intergenic	0.29	1.08×10^{-8}
	rs13432405	2	158,809,474	Intergenic	0.29	1.08×10^{-8}
	rs13397930	2	158,810,210	Intergenic	0.29	1.08×10^{-8}
	rs10167048	2	158,811,030	Intergenic	0.29	1.08×10^{-8}
	rs10175615	2	158,811,223	Intergenic	0.29	1.08×10^{-8}
	rs11900941	2	158,812,042	Intergenic	0.29	1.08×10^{-8}
	rs10171199	2	158,812,888	Intergenic	0.29	1.08×10^{-8}

rs79918420	2	158,814,171	Intergenic	0.29	1.08×10^{-8}
rs13408052	2	158,814,842	Intergenic	0.30	6.66×10^{-9}
rs13408263	2	158,815,012	Intergenic	0.29	1.08×10^{-8}
rs13386758	2	158,815,586	Intergenic	0.30	6.66×10^{-9}
rs13387442	2	158,816,186	Intergenic	0.29	1.08×10^{-8}
rs78122893	2	158,816,395	Intergenic	0.30	6.66×10^{-9}
rs13415315	2	158,816,891	Intergenic	0.29	1.08×10^{-8}
rs13403742	2	158,816,902	Intergenic	0.29	1.08×10^{-8}
rs13404058	2	158,817,214	Intergenic	0.29	1.41×10^{-8}
rs11902033	2	158,820,256	Intergenic	0.30	8.63×10^{-9}
rs2355159	2	158,820,860	Intergenic	0.29	1.41×10^{-8}
rs2108812	2	158,823,815	Intergenic	0.30	8.63×10^{-9}
rs116562129	2	158,828,149	Intergenic	0.36	2.08×10^{-10}
rs78308467	2	158,828,240	Intergenic	0.30	8.63×10^{-9}
rs10170886	2	158,829,363	Intergenic	0.29	1.41×10^{-8}
rs11904437	2	158,830,525	Intergenic	0.29	1.71×10^{-8}
rs72992248	2	231,197,652	*SP140L*	0.35	3.02×10^{-9}
rs115882550	3	83,458,648	Intergenic	0.31	2.38×10^{-10}
rs6873250	5	3,876,466	Intergenic	0.33	9.00×10^{-10}
rs72729464	5	3,882,170	Intergenic	0.33	9.00×10^{-10}
rs12520673	5	3,885,882	Intergenic	0.31	3.01×10^{-9}
rs6860576	5	3,887,013	Intergenic	0.32	1.61×10^{-9}
rs7721528	5	3,888,766	Intergenic	0.32	1.61×10^{-9}
rs59862860	5	3,892,971	Intergenic	0.32	1.94×10^{-9}
rs67486607	5	3,895,075	Intergenic	0.32	1.94×10^{-9}
rs56194178	5	63,020,210	Intergenic	0.32	9.55×10^{-9}
rs72766206	5	63,022,860	Intergenic	0.33	3.09×10^{-9}
rs72766215	5	63,034,388	Intergenic	0.33	3.09×10^{-9}
rs72766223	5	63,040,234	Intergenic	0.33	3.09×10^{-9}
rs72766225	5	63,042,657	Intergenic	0.37	3.33×10^{-10}
rs17545761	5	146,647,488	*STK32A*	0.36	5.14×10^{-11}
rs73794346	5	146,647,973	*STK32A*	0.36	5.14×10^{-11}
rs57543595	5	146,650,914	*STK32A*	0.36	5.14×10^{-11}
rs56070607	5	146,651,084	*STK32A*	0.36	5.14×10^{-11}
rs55778516	5	146,651,130	*STK32A*	0.36	5.14×10^{-11}
rs77652165	5	146,652,471	*STK32A*	0.36	5.14×10^{-11}
rs73794372	5	146,652,849	*STK32A*	0.36	5.14×10^{-11}
rs76535160	5	146,654,318	*STK32A*	0.36	5.14×10^{-11}
rs17468085	5	146,655,511	*STK32A*	0.36	5.14×10^{-11}
rs75926181	5	146,657,499	*STK32A*	0.36	5.14×10^{-11}
rs115884442	5	146,660,024	*STK32A*	0.36	5.14×10^{-11}
rs80343345	5	146,660,758	*STK32A*	0.36	5.14×10^{-11}
rs141016113	5	146,662,215	*STK32A*	0.36	5.14×10^{-11}
rs116480535	5	146,664,189	*STK32A*	0.33	3.88×10^{-10}
rs114514147	5	146,665,169	*STK32A*	0.33	3.88×10^{-10}
rs114316116	5	146,673,485	*STK32A*	0.33	3.88×10^{-10}
rs78772082	5	146,674,762	*STK32A*	0.33	3.88×10^{-10}
rs74374168	5	146,675,454	*STK32A*	0.33	3.88×10^{-10}
rs78649901	5	146,675,510	*STK32A*	0.33	3.88×10^{-10}

rs74991998	5	146,676,067	*STK32A*	0.32	6.26×10^{-10}
rs79220773	5	146,677,174	*STK32A*	0.32	6.26×10^{-10}
rs79541136	5	146,677,740	*STK32A*	0.32	1.02×10^{-9}
rs12663463	6	41,446,439	Intergenic	0.32	4.45×10^{-10}
rs56228155	6	41,450,083	Intergenic	0.34	8.32×10^{-11}
rs56094721	6	41,450,339	Intergenic	0.34	8.32×10^{-11}
rs11760167	6	106,656,099	*ATG5*	0.37	2.10×10^{-9}
rs146369928	6	115,472,507	Intergenic	0.32	6.75×10^{-9}
rs34486266	6	115,487,074	Intergenic	0.31	2.01×10^{-8}
rs78851618	6	147,047,296	*ADGB*	0.36	3.04×10^{-9}
rs76974166	6	147,048,955	*ADGB*	0.34	1.10×10^{-8}
rs79238855	6	147,052,125	*ADGB*	0.34	1.10×10^{-8}
rs76988737	6	147,055,623	*ADGB*	0.34	1.10×10^{-8}
rs79047306	6	147,056,760	*ADGB*	0.34	1.10×10^{-8}
rs79017204	6	147,059,238	*ADGB*	0.34	1.10×10^{-8}
rs80014390	6	147,059,490	*ADGB*	0.34	1.10×10^{-8}
rs80345405	6	147,069,749	*ADGB*	0.34	1.10×10^{-8}
rs117110483	6	147,072,612	*ADGB*	0.34	1.10×10^{-8}
rs78554154	6	147,076,662	*ADGB*	0.34	1.10×10^{-8}
rs17535206	6	147,091,074	*ADGB*	0.33	2.22×10^{-8}
rs259418	6	147,094,641	*ADGB*	0.33	2.22×10^{-8}
rs171826	6	147,096,447	*ADGB*	0.33	2.22×10^{-8}
rs16871986	7	105,399,650	*ATXN7L1*	0.28	1.93×10^{-8}
rs117766049	7	129,684,199	*ZC3HC1*	0.40	2.87×10^{-12}
rs11764066	7	129,694,738	Intergenic	0.37	4.45×10^{-11}
rs143068807	8	57,176,364	Intergenic	0.27	2.68×10^{-8}
rs112931164	9	140,294,496	*EXD3*	0.34	1.48×10^{-8}
rs10124498	9	140,295,278	*EXD3*	0.35	3.57×10^{-10}
rs12257253	10	2,284,381	Intergenic	0.34	1.37×10^{-8}
rs34700213	10	15,299,262	*FAM171A1*	0.29	2.50×10^{-9}
rs117417471	10	56,973,794	Intergenic	0.34	2.44×10^{-8}
rs7121626	11	5,379,516	*OR51B5*	0.36	2.30×10^{-9}
rs7939481	11	5,446,156	*OR51B5*	0.34	1.85×10^{-8}
rs61894126	11	5,449,449	*OR51B5*	0.36	7.93×10^{-9}
rs61894127	11	5,450,493	*OR51B5*	0.33	3.17×10^{-8}
rs16930808	11	5,451,094	*OR51B5*	0.37	3.95×10^{-9}
rs75819971	11	123,347,708	Intergenic	0.25	1.69×10^{-8}
rs117888950	12	74,131,740	Intergenic	0.29	4.23×10^{-8}
rs74479492	12	74,442,420	Intergenic	0.32	3.98×10^{-8}
rs79308023	12	74,449,371	Intergenic	0.32	3.98×10^{-8}
rs7954064	12	74,460,019	Intergenic	0.32	3.98×10^{-8}
rs75617695	12	74,460,439	Intergenic	0.32	3.98×10^{-8}
rs117898983	12	74,467,396	Intergenic	0.32	3.98×10^{-8}
rs139034977	12	74,467,905	Intergenic	0.32	3.98×10^{-8}
rs10506704	12	74,472,786	Intergenic	0.32	3.98×10^{-8}
rs80351845	12	74,476,822	Intergenic	0.32	3.98×10^{-8}
rs117866772	12	74,481,378	Intergenic	0.32	3.98×10^{-8}
rs7964807	12	74,484,387	Intergenic	0.32	2.03×10^{-8}
rs7965039	12	74,484,536	Intergenic	0.32	3.98×10^{-8}

rs151196400	12	74,486,750	Intergenic	0.32	3.98×10^{-8}
rs2365428	12	74,488,719	Intergenic	0.32	3.98×10^{-8}
rs116975431	12	74,489,764	Intergenic	0.32	3.98×10^{-8}
rs117957068	12	74,491,627	Intergenic	0.32	3.98×10^{-8}
rs2365426	12	74,492,568	Intergenic	0.32	3.98×10^{-8}
rs75478957	12	74,493,555	Intergenic	0.32	3.98×10^{-8}
rs76640917	12	74,495,006	Intergenic	0.32	3.98×10^{-8}
rs7959349	12	74,495,388	Intergenic	0.32	3.98×10^{-8}
rs12320777	12	74,510,145	Intergenic	0.32	3.98×10^{-8}
rs80090951	12	74,510,976	Intergenic	0.32	3.98×10^{-8}
rs7960444	12	74,513,151	Intergenic	0.32	3.98×10^{-8}
rs2049205	12	74,514,939	Intergenic	0.32	3.98×10^{-8}
rs7306803	12	74,521,219	Intergenic	0.32	3.98×10^{-8}
rs116887184	12	74,523,219	Intergenic	0.32	3.98×10^{-8}
rs75674568	12	74,525,779	Intergenic	0.32	3.98×10^{-8}
rs78966406	12	74,532,289	*LOC100507377*	0.32	3.98×10^{-8}
rs78321472	12	74,533,995	*LOC100507377*	0.32	3.98×10^{-8}
rs116882385	12	74,543,795	*LOC100507377*	0.32	3.98×10^{-8}
rs17768585	12	74,545,304	*LOC100507377*	0.32	3.98×10^{-8}
rs116929977	12	74,555,636	*LOC100507377*	0.32	3.98×10^{-8}
rs1522125	12	74,556,765	*LOC100507377*	0.32	3.98×10^{-8}
rs189351960	12	74,560,964	*LOC100507377*	0.33	2.88×10^{-8}
rs7972328	12	74,570,627	*LOC100507377*	0.32	3.98×10^{-8}
rs78736553	12	74,574,220	*LOC100507377*	0.32	3.98×10^{-8}
rs78088641	12	74,580,926	*LOC100507377*	0.32	3.98×10^{-8}
rs117679796	12	74,583,323	*LOC100507377*	0.32	3.98×10^{-8}
rs139746544	12	74,583,646	*LOC100507377*	0.32	3.98×10^{-8}
rs77620106	12	74,592,298	*LOC100507377*	0.32	3.98×10^{-8}
rs76881855	12	74,600,042	*LOC100507377*	0.32	3.98×10^{-8}
rs77504227	12	74,624,665	*LOC100507377*	0.32	3.98×10^{-8}
rs17006602	12	80,690,425	*OTOGL*	0.32	8.35×10^{-9}
rs17248984	12	80,716,485	*OTOGL*	0.32	1.27×10^{-8}
rs17249026	12	80,717,094	*OTOGL*	0.32	1.27×10^{-8}
rs76018282	12	80,722,241	*OTOGL*	0.32	1.27×10^{-8}
rs4381423	12	80,741,184	*OTOGL*	0.30	3.78×10^{-8}
rs1037340	12	80,743,318	*OTOGL*	0.32	1.05×10^{-8}
rs1037338	12	80,743,626	*OTOGL*	0.32	1.05×10^{-8}
rs10161042	12	80,745,683	*OTOGL*	0.32	1.27×10^{-8}
rs75058023	12	80,748,602	*OTOGL*	0.32	1.93×10^{-8}
rs2122700	12	80,751,042	*OTOGL*	0.31	1.61×10^{-8}
rs2034528	12	80,752,660	*OTOGL*	0.32	1.27×10^{-8}
rs79907299	12	80,752,859	*OTOGL*	0.32	1.27×10^{-8}
rs79171001	12	80,753,741	*OTOGL*	0.32	1.27×10^{-8}
rs7969728	12	80,753,804	*OTOGL*	0.32	1.05×10^{-8}
rs10778729	12	80,754,747	*OTOGL*	0.32	1.05×10^{-8}
rs2400696	12	80,756,770	*OTOGL*	0.32	1.05×10^{-8}
rs76833906	12	80,757,262	*OTOGL*	0.32	1.27×10^{-8}
rs2400698	12	80,757,497	*OTOGL*	0.31	2.20×10^{-8}
rs921417	12	80,759,351	*OTOGL*	0.32	1.27×10^{-8}

rs921418	12	80,759,399	*OTOGL*	0.32	1.05×10^{-8}
rs4842348	12	80,759,781	*OTOGL*	0.32	1.27×10^{-8}
rs2717482	12	80,761,465	*OTOGL*	0.32	1.27×10^{-8}
rs1037336	12	80,765,741	*OTOGL*	0.32	1.05×10^{-8}
rs7968158	12	80,768,109	*OTOGL*	0.32	1.05×10^{-8}
rs10746158	12	80,768,374	*OTOGL*	0.32	1.05×10^{-8}
rs2598531	12	80,775,056	Intergenic	0.32	1.05×10^{-8}
rs7959610	12	80,778,807	Intergenic	0.32	1.27×10^{-8}
rs2598537	12	80,781,699	Intergenic	0.32	1.27×10^{-8}
rs2598529	12	80,788,779	Intergenic	0.32	1.27×10^{-8}
rs2465814	12	80,789,445	Intergenic	0.32	1.27×10^{-8}
rs7969982	12	80,793,045	Intergenic	0.32	1.27×10^{-8}
rs2598523	12	80,798,318	Intergenic	0.32	1.27×10^{-8}
rs2598527	12	80,800,421	Intergenic	0.32	1.27×10^{-8}
rs2443190	12	80,802,383	Intergenic	0.32	1.27×10^{-8}
rs2717471	12	80,807,880	Intergenic	0.32	1.27×10^{-8}
rs4399374	12	80,814,828	Intergenic	0.32	1.27×10^{-8}
rs4842284	12	80,823,060	Intergenic	0.31	2.50×10^{-8}
rs11614522	12	80,833,089	Intergenic	0.30	3.61×10^{-8}
rs7972765	12	80,833,840	Intergenic	0.30	3.61×10^{-8}
rs7959257	12	80,833,974	Intergenic	0.30	3.61×10^{-8}
rs11611529	12	80,835,014	Intergenic	0.30	3.61×10^{-8}
rs11609716	12	80,835,125	Intergenic	0.30	3.61×10^{-8}
rs73343885	12	80,835,509	Intergenic	0.30	3.61×10^{-8}
rs11838287	12	80,835,853	Intergenic	0.30	3.61×10^{-8}
rs58304957	12	80,836,548	Intergenic	0.30	3.61×10^{-8}
rs73343892	12	80,836,774	Intergenic	0.30	3.61×10^{-8}
rs56711652	12	80,838,790	*PTPRQ*	0.30	3.61×10^{-8}
rs60520650	12	80,838,885	*PTPRQ*	0.30	3.61×10^{-8}
rs78809205	12	80,840,221	*PTPRQ*	0.30	3.56×10^{-8}
rs184037792	12	80,846,305	*PTPRQ*	0.30	3.61×10^{-8}
rs140264421	12	80,847,016	*PTPRQ*	0.30	3.61×10^{-8}
rs7294621	12	80,851,812	*PTPRQ*	0.30	3.61×10^{-8}
rs59973438	12	80,852,925	*PTPRQ*	0.30	3.61×10^{-8}
rs11608378	12	80,854,501	*PTPRQ*	0.30	3.61×10^{-8}
rs60342585	12	80,855,825	*PTPRQ*	0.30	3.61×10^{-8}
rs140528976	12	80,857,137	*PTPRQ*	0.30	3.61×10^{-8}
rs10744193	12	125,570,197	*AACS*	0.38	1.05×10^{-9}
rs56838200	13	106,123,349	*DAOA; DAOA-AS1*	0.32	1.27×10^{-8}
rs12434703	14	24,461,668	*DHRS4L2*	0.33	5.85×10^{-9}
rs117548728	14	28,325,001	Intergenic	0.28	4.75×10^{-8}
rs117219682	14	59,558,654	Intergenic	0.40	8.17×10^{-11}
rs72721031	14	68,012,909	*PLEKHH1*	0.32	3.44×10^{-9}
rs7165046	15	97,778,882	Intergenic	0.32	4.18×10^{-8}
rs34381428	16	16,042,558	Intergenic	0.24	1.10×10^{-8}
rs762778	16	16,047,448	*ABCC1*	0.23	4.45×10^{-8}
rs762775	16	16,048,157	*ABCC1*	0.22	4.91×10^{-8}
rs71378214	16	16,048,653	*ABCC1*	0.23	3.30×10^{-8}
rs66704776	16	62,002,463	*CDH8*	0.29	1.76×10^{-8}

	rs16964108	16	62,003,448	*CDH8*	0.29	3.21×10^{-8}
	rs3784843	16	62,003,873	*CDH8*	0.30	7.84×10^{-9}
	rs61196665	16	62,004,512	*CDH8*	0.29	3.21×10^{-8}
	rs56391958	16	62,005,709	*CDH8*	0.29	1.14×10^{-8}
	rs16964118	16	62,009,483	*CDH8*	0.29	3.21×10^{-8}
	rs55973238	16	62,009,665	*CDH8*	0.29	3.21×10^{-8}
	rs1397133	16	62,009,911	*CDH8*	0.29	3.21×10^{-8}
	rs1510164	16	62,011,417	*CDH8*	0.30	1.46×10^{-8}
	rs286672	16	82,884,821	*CDH13*	0.35	1.78×10^{-8}
	rs7226068	17	17,231,431	*NT5M*	0.35	1.00×10^{-9}
	rs115384385	18	49,502,045	Intergenic	0.30	6.90×10^{-9}
	rs79355234	18	49,502,964	Intergenic	0.31	3.04×10^{-9}
	rs9946784	18	49,503,711	Intergenic	0.30	6.90×10^{-9}
	rs9952887	18	49,505,224	Intergenic	0.34	5.45×10^{-10}
	rs73453344	18	49,511,348	Intergenic	0.34	3.93×10^{-10}
	rs73453345	18	49,514,729	Intergenic	0.30	9.67×10^{-9}
	rs115725720	19	35,240,069	Intergenic	0.34	2.07×10^{-8}
	rs11696375	20	16,963,721	Intergenic	0.26	4.59×10^{-8}
	rs191717682	21	46,563,559	*ADARB1*	0.30	5.51×10^{-9}
	rs183572250	21	46,563,560	*ADARB1*	0.30	5.51×10^{-9}
	rs13048685	21	46,573,848	*ADARB1*	0.28	1.55×10^{-8}
COL4A6	rs12759318	1	22,308,435	*CELA3B*	0.11	2.30×10^{-12}
	rs2506992	1	48,097,805	Intergenic	0.09	4.03×10^{-8}
	rs12737330	1	81,723,771	Intergenic	0.09	2.94×10^{-8}
	rs12066036	1	102,295,275	*OLFM3*	0.10	1.47×10^{-9}
	rs114616075	1	195,519,020	Intergenic	0.09	3.99×10^{-8}
	rs6681782	1	199,623,380	Intergenic	0.09	1.42×10^{-8}
	rs17019582	2	4,702,434	*LINC01249*	0.09	3.86×10^{-8}
	rs17019595	2	4,704,141	Intergenic	0.09	3.86×10^{-8}
	rs72992248	2	231,197,652	*SP140L*	0.10	7.69×10^{-9}
	rs78261017	4	17,822,768	*NCAPG*	0.10	7.81×10^{-9}
	rs79611936	4	17,890,778	*LCORL*	0.09	4.97×10^{-8}
	rs79021772	4	17,891,057	*LCORL*	0.09	4.97×10^{-8}
	rs77891402	4	18,035,604	Intergenic	0.09	4.48×10^{-8}
	rs116011024	4	37,145,905	Intergenic	0.08	3.77×10^{-8}
	rs115221292	4	95,833,364	*BMPR1B*	0.10	3.56×10^{-8}
	rs188631814	4	137,400,677	Intergenic	0.10	7.85×10^{-9}
	rs77668970	4	161,592,521	Intergenic	0.08	6.65×10^{-9}
	rs141082323	5	715,278	Intergenic	0.09	1.91×10^{-9}
	rs139468541	5	740,120	Intergenic	0.08	4.07×10^{-8}
	rs74454732	5	8,529,419	Intergenic	0.10	6.95×10^{-9}
	rs72766225	5	63,042,657	Intergenic	0.09	1.58×10^{-8}
	rs2115766	5	97,051,163	Intergenic	0.08	3.79×10^{-8}
	rs13171515	5	146,638,927	*STK32A*	0.06	2.16×10^{-8}
	rs13176837	5	146,639,205	*STK32A*	0.06	2.16×10^{-8}
	rs13163858	5	146,640,039	*STK32A*	0.06	2.16×10^{-8}
	rs35901580	5	146,642,649	*STK32A*	0.06	2.16×10^{-8}
	rs35046779	5	146,645,224	*STK32A*	0.06	2.16×10^{-8}
	rs71580434	5	146,646,156	*STK32A*	0.06	2.16×10^{-8}

rs34183645	5	146,646,627	*STK32A*	0.06	2.16×10^{-8}
rs17545761	5	146,647,488	*STK32A*	0.11	7.59×10^{-13}
rs73794346	5	146,647,973	*STK32A*	0.11	7.59×10^{-13}
rs57543595	5	146,650,914	*STK32A*	0.11	7.59×10^{-13}
rs56070607	5	146,651,084	*STK32A*	0.11	7.59×10^{-13}
rs55778516	5	146,651,130	*STK32A*	0.11	7.59×10^{-13}
rs77652165	5	146,652,471	*STK32A*	0.11	7.59×10^{-13}
rs73794372	5	146,652,849	*STK32A*	0.11	7.59×10^{-13}
rs76535160	5	146,654,318	*STK32A*	0.11	7.59×10^{-13}
rs17468085	5	146,655,511	*STK32A*	0.11	7.59×10^{-13}
rs75926181	5	146,657,499	*STK32A*	0.11	7.59×10^{-13}
rs115884442	5	146,660,024	*STK32A*	0.11	7.59×10^{-13}
rs80343345	5	146,660,758	*STK32A*	0.11	7.59×10^{-13}
rs141016113	5	146,662,215	*STK32A*	0.11	7.59×10^{-13}
rs116480535	5	146,664,189	*STK32A*	0.10	1.03×10^{-11}
rs114514147	5	146,665,169	*STK32A*	0.10	1.03×10^{-11}
rs114316116	5	146,673,485	*STK32A*	0.10	1.03×10^{-11}
rs78772082	5	146,674,762	*STK32A*	0.10	1.03×10^{-11}
rs74374168	5	146,675,454	*STK32A*	0.10	1.03×10^{-11}
rs78649901	5	146,675,510	*STK32A*	0.10	1.03×10^{-11}
rs74991998	5	146,676,067	*STK32A*	0.10	2.15×10^{-11}
rs79220773	5	146,677,174	*STK32A*	0.10	2.15×10^{-11}
rs79541136	5	146,677,740	*STK32A*	0.10	2.43×10^{-11}
rs146728068	6	16,453,666	*ATXN1*	0.09	1.97×10^{-8}
rs78851618	6	147,047,296	*ADGB*	0.09	4.95×10^{-8}
rs73035146	6	170,447,262	Intergenic	0.10	1.01×10^{-10}
rs71518355	7	973,917	*ADAP1*	0.09	1.29×10^{-8}
rs117418571	7	5,486,051	Intergenic	0.09	1.62×10^{-8}
rs73049985	7	7,285,708	*C1GALT1*	0.09	3.96×10^{-8}
rs16871986	7	105,399,650	*ATXN7L1*	0.08	2.53×10^{-8}
rs149596765	7	129,641,095	Intergenic	0.07	3.63×10^{-8}
rs117766049	7	129,684,199	*ZC3HC1*	0.12	3.90×10^{-14}
rs11763967	7	129,694,473	Intergenic	0.07	2.18×10^{-8}
rs11764066	7	129,694,738	Intergenic	0.11	1.02×10^{-12}
rs2597395	8	20,562,950	Intergenic	0.09	1.75×10^{-8}
rs2616246	8	20,577,316	Intergenic	0.09	2.13×10^{-8}
rs4244464	8	20,645,930	Intergenic	0.10	1.60×10^{-9}
rs6474703	9	12,336,801	Intergenic	0.11	3.40×10^{-11}
rs6474704	9	12,336,809	Intergenic	0.10	1.54×10^{-10}
rs34975489	9	85,869,511	*FRMD3*	0.07	1.19×10^{-8}
rs117417471	10	56,973,794	Intergenic	0.11	5.95×10^{-11}
rs76709638	10	129,985,694	Intergenic	0.09	4.10×10^{-8}
rs7121626	11	5,379,516	*OR51B5*	0.10	3.39×10^{-9}
rs80020515	11	29,423,557	Intergenic	0.08	1.06×10^{-8}
rs79753183	11	29,423,561	Intergenic	0.08	1.06×10^{-8}
rs182129188	11	48,539,676	Intergenic	0.09	1.83×10^{-8}
rs10890946	11	108,978,494	Intergenic	0.09	4.14×10^{-8}
rs117888950	12	74,131,740	Intergenic	0.08	3.77×10^{-8}
rs74479492	12	74,442,420	Intergenic	0.09	2.39×10^{-8}

rs79308023	12	74,449,371	Intergenic	0.09	2.39×10^{-8}
rs7954064	12	74,460,019	Intergenic	0.09	2.39×10^{-8}
rs75617695	12	74,460,439	Intergenic	0.09	2.39×10^{-8}
rs117898983	12	74,467,396	Intergenic	0.09	2.39×10^{-8}
rs139034977	12	74,467,905	Intergenic	0.09	2.39×10^{-8}
rs10506704	12	74,472,786	Intergenic	0.09	2.39×10^{-8}
rs80351845	12	74,476,822	Intergenic	0.09	2.39×10^{-8}
rs117866772	12	74,481,378	Intergenic	0.09	2.39×10^{-8}
rs7964807	12	74,484,387	Intergenic	0.09	3.91×10^{-8}
rs7965039	12	74,484,536	Intergenic	0.09	2.39×10^{-8}
rs151196400	12	74,486,750	Intergenic	0.09	2.39×10^{-8}
rs2365428	12	74,488,719	Intergenic	0.09	2.39×10^{-8}
rs116975431	12	74,489,764	Intergenic	0.09	2.39×10^{-8}
rs117957068	12	74,491,627	Intergenic	0.09	2.39×10^{-8}
rs2365426	12	74,492,568	Intergenic	0.09	2.39×10^{-8}
rs75478957	12	74,493,555	Intergenic	0.09	2.39×10^{-8}
rs76640917	12	74,495,006	Intergenic	0.09	2.39×10^{-8}
rs7959349	12	74,495,388	Intergenic	0.09	2.39×10^{-8}
rs12320777	12	74,510,145	Intergenic	0.09	2.39×10^{-8}
rs80090951	12	74,510,976	Intergenic	0.09	2.39×10^{-8}
rs77573113	12	74,512,140	Intergenic	0.09	3.86×10^{-8}
rs7960444	12	74,513,151	Intergenic	0.09	2.39×10^{-8}
rs2049205	12	74,514,939	Intergenic	0.09	2.39×10^{-8}
rs7306803	12	74,521,219	Intergenic	0.09	2.39×10^{-8}
rs116887184	12	74,523,219	Intergenic	0.09	2.39×10^{-8}
rs75674568	12	74,525,779	Intergenic	0.09	2.39×10^{-8}
rs78966406	12	74,532,289	*LOC100507377*	0.09	2.39×10^{-8}
rs78321472	12	74,533,995	*LOC100507377*	0.09	2.39×10^{-8}
rs116882385	12	74,543,795	*LOC100507377*	0.09	2.39×10^{-8}
rs17768585	12	74,545,304	*LOC100507377*	0.09	2.39×10^{-8}
rs116929977	12	74,555,636	*LOC100507377*	0.09	2.39×10^{-8}
rs1522125	12	74,556,765	*LOC100507377*	0.09	2.39×10^{-8}
rs189351960	12	74,560,964	*LOC100507377*	0.10	1.09×10^{-9}
rs7972328	12	74,570,627	*LOC100507377*	0.09	2.39×10^{-8}
rs78736553	12	74,574,220	*LOC100507377*	0.09	2.39×10^{-8}
rs78088641	12	74,580,926	*LOC100507377*	0.09	2.39×10^{-8}
rs117679796	12	74,583,323	*LOC100507377*	0.09	2.39×10^{-8}
rs139746544	12	74,583,646	*LOC100507377*	0.09	2.39×10^{-8}
rs77620106	12	74,592,298	*LOC100507377*	0.09	2.39×10^{-8}
rs76881855	12	74,600,042	*LOC100507377*	0.09	2.39×10^{-8}
rs77504227	12	74,624,665	*LOC100507377*	0.09	2.39×10^{-8}
rs148043013	12	132,169,820	Intergenic	0.07	4.56×10^{-8}
rs2296990	13	99,502,260	*DOCK9*	0.09	2.06×10^{-8}
rs12428668	13	99,536,571	*DOCK9*	0.08	4.89×10^{-8}
rs79164790	13	99,547,180	*DOCK9*	0.08	4.89×10^{-8}
rs75707960	13	99,557,845	*DOCK9*	0.08	4.31×10^{-8}
rs117219682	14	59,558,654	Intergenic	0.11	2.13×10^{-11}
rs72721031	14	68,012,909	*PLEKHH1*	0.09	5.12×10^{-9}
rs190120154	14	103,575,566	*EXOC3L4*	0.09	3.78×10^{-8}

	rs10520087	15	38,072,896	Intergenic	0.08	2.34×10^{-9}
	rs57561745	15	38,075,566	Intergenic	0.08	1.74×10^{-8}
	rs7165046	15	97,778,882	Intergenic	0.09	4.72×10^{-8}
	rs1858868	16	23,885,179	*PRKCB*	0.09	3.68×10^{-8}
	rs8044732	16	23,885,608	*PRKCB*	0.09	3.68×10^{-8}
	rs4435258	16	23,885,959	*PRKCB*	0.09	3.68×10^{-8}
	rs66704776	16	62,002,463	*CDH8*	0.08	3.17×10^{-9}
	rs16964108	16	62,003,448	*CDH8*	0.09	3.46×10^{-9}
	rs3784843	16	62,003,873	*CDH8*	0.09	1.01×10^{-9}
	rs61196665	16	62,004,512	*CDH8*	0.09	3.46×10^{-9}
	rs56391958	16	62,005,709	*CDH8*	0.09	1.78×10^{-9}
	rs16964118	16	62,009,483	*CDH8*	0.09	3.46×10^{-9}
	rs55973238	16	62,009,665	*CDH8*	0.09	3.46×10^{-9}
	rs1397133	16	62,009,911	*CDH8*	0.09	3.46×10^{-9}
	rs1510164	16	62,011,417	*CDH8*	0.09	4.29×10^{-9}
	rs7226068	17	17,231,431	*NT5M*	0.10	1.68×10^{-9}
	rs145621502	17	22,243,341	Intergenic	0.08	4.17×10^{-8}
	rs151244246	20	37,107,486	*RALGAPB*	0.09	2.42×10^{-9}
	rs139172260	20	51,958,877	*TSHZ2*	0.09	2.15×10^{-8}
	rs61422883	20	56,368,107	Intergenic	0.07	2.01×10^{-8}
	rs77249457	20	56,368,913	Intergenic	0.07	3.13×10^{-8}
	rs74717384	20	56,373,076	Intergenic	0.07	3.03×10^{-8}
	rs113367448	20	56,386,246	Intergenic	0.08	1.38×10^{-8}
	rs113841886	20	56,387,916	Intergenic	0.08	8.97×10^{-9}
	rs73154681	22	31,236,543	*OSBP2*	0.08	3.26×10^{-8}
COL6A2	rs114422885	3	30,278,181	Intergenic	0.09	1.84×10^{-8}
	rs112001313	4	72,501,807	Intergenic	0.08	2.46×10^{-8}
	rs72688432	4	99,379,586	Intergenic	0.07	6.74×10^{-9}
	rs57849067	9	77,836,358	Intergenic	0.07	8.11×10^{-9}
	rs75498878	9	77,838,686	Intergenic	0.07	1.57×10^{-8}
	rs150146176	9	77,840,842	Intergenic	0.07	1.57×10^{-8}
	rs77618173	9	77,845,719	Intergenic	0.07	1.57×10^{-8}
COL9A1	rs6424206	1	233,637,491	Intergenic	0.01	3.42×10^{-8}
	rs12756713	1	233,638,169	Intergenic	0.01	8.61×10^{-9}
	rs12743334	1	233,638,192	Intergenic	0.01	8.61×10^{-9}
	rs6662352	1	233,642,172	Intergenic	0.01	9.33×10^{-9}
	rs10910200	1	233,643,856	Intergenic	0.01	9.33×10^{-9}
	rs11689566	2	118,966,616	Intergenic	0.02	4.14×10^{-8}
COMP	rs148591107	3	167,850,620	Intergenic	0.04	4.10×10^{-8}
	rs143172940	3	167,851,255	Intergenic	0.04	4.10×10^{-8}
CREB3L1	rs10921465	1	193,891,763	Intergenic	1.17	1.37×10^{-8}
	rs77272296	1	193,896,315	Intergenic	1.17	1.37×10^{-8}
	rs115496130	7	8,134,870	Intergenic	0.94	2.93×10^{-8}
	rs73333264	8	127,486,784	Intergenic	1.13	3.79×10^{-8}
CREB3L4	rs9950012	18	73,332,973	Intergenic	1.38	8.22×10^{-9}
	rs9964145	18	73,333,179	Intergenic	1.38	8.22×10^{-9}
	rs1954959	18	73,333,770	Intergenic	1.28	3.74×10^{-8}
CREB5	rs118180039	8	37,839,194	Intergenic	2.20	1.85×10^{-8}
	rs11634798	15	69,684,389	*PAQR5*	2.93	3.95×10^{-8}

	rs1394414	15	69,697,499	*PAQR5*	2.93	3.95×10^{-8}
	rs3825858	15	69,728,949	*KIF23*	2.93	3.95×10^{-8}
	rs937724	15	69,732,736	*KIF23*	2.93	3.95×10^{-8}
	rs3784363	15	69,739,847	*KIF23*	2.93	3.95×10^{-8}
	rs13613	15	69,740,350	*KIF23*	2.93	3.95×10^{-8}
	rs8027310	15	69,742,798	Intergenic	2.93	3.95×10^{-8}
CSF1R	rs17655490	5	149,340,009	Intergenic	0.13	1.90×10^{-9}
	rs17655514	5	149,346,044	*SLC26A2*	0.12	3.87×10^{-8}
	rs56369041	5	149,347,295	*SLC26A2*	0.12	7.56×10^{-9}
	rs11739934	5	149,349,161	*SLC26A2*	0.12	9.86×10^{-9}
	rs1560680	5	149,353,117	*SLC26A2*	0.13	1.39×10^{-8}
	rs2879955	5	149,353,508	*SLC26A2*	0.12	3.47×10^{-8}
	rs6896747	5	149,353,564	*SLC26A2*	0.12	9.30×10^{-9}
	rs3756307	5	149,356,486	*SLC26A2*	0.12	3.47×10^{-8}
	rs3812007	5	149,356,963	*SLC26A2*	0.12	2.27×10^{-8}
	rs17110742	5	149,357,060	*SLC26A2*	0.12	2.27×10^{-8}
	rs6860282	5	149,359,625	*SLC26A2*	0.12	9.86×10^{-9}
	rs3776070	5	149,361,221	*SLC26A2*	0.12	2.58×10^{-9}
	rs17711997	5	149,366,714	*SLC26A2*	0.13	1.87×10^{-9}
	rs10476926	5	149,366,929	*SLC26A2*	0.12	2.07×10^{-8}
	rs1816406	5	149,371,478	Intergenic	0.13	2.08×10^{-9}
	rs11748318	5	149,372,149	Intergenic	0.13	2.08×10^{-9}
	rs973077	5	149,373,140	*TIGD6*	0.13	2.08×10^{-9}
	rs73798339	5	149,373,740	*TIGD6*	0.13	2.08×10^{-9}
	rs145060437	5	149,377,533	*TIGD6*	0.15	4.73×10^{-10}
	rs11745580	5	149,378,425	*TIGD6*	0.14	8.31×10^{-10}
	rs6579765	5	149,379,897	*TIGD6*	0.15	1.30×10^{-10}
	rs4705403	5	149,380,493	*HMGXB3; TIGD6*	0.15	5.06×10^{-10}
	rs4705405	5	149,383,776	*HMGXB3*	0.15	7.76×10^{-11}
	rs17110778	5	149,383,871	*HMGXB3*	0.15	7.76×10^{-11}
	rs17110782	5	149,383,976	*HMGXB3*	0.15	7.76×10^{-11}
	rs11738182	5	149,384,675	*HMGXB3*	0.15	5.06×10^{-10}
	rs11167490	5	149,384,895	*HMGXB3*	0.15	5.06×10^{-10}
	rs11738277	5	149,385,031	*HMGXB3*	0.15	5.06×10^{-10}
	rs17110786	5	149,385,394	*HMGXB3*	0.15	5.06×10^{-10}
	rs17110790	5	149,385,425	*HMGXB3*	0.15	5.06×10^{-10}
	rs11742537	5	149,385,872	*HMGXB3*	0.15	5.06×10^{-10}
	rs6872735	5	149,386,314	*HMGXB3*	0.15	5.06×10^{-10}
	rs6888795	5	149,387,657	*HMGXB3*	0.16	1.16×10^{-10}
	rs1469689	5	149,388,340	*HMGXB3*	0.15	5.06×10^{-10}
	rs4705406	5	149,388,864	*HMGXB3*	0.15	5.06×10^{-10}
	rs11167491	5	149,389,573	*HMGXB3*	0.15	5.06×10^{-10}
	rs2241698	5	149,390,148	*HMGXB3*	0.15	5.06×10^{-10}
	rs4597953	5	149,394,704	*HMGXB3*	0.15	5.01×10^{-10}
	rs7700386	5	149,396,515	*HMGXB3*	0.16	1.10×10^{-11}
	rs7720460	5	149,396,534	*HMGXB3*	0.16	1.10×10^{-11}
	rs7700709	5	149,396,646	*HMGXB3*	0.16	1.10×10^{-11}
	rs11740243	5	149,397,104	*HMGXB3*	0.16	1.10×10^{-11}
	rs11741233	5	149,398,555	*HMGXB3*	0.15	5.06×10^{-10}

	rs4705105	5	149,399,330	*HMGXB3*	0.15	5.06×10^{-10}
	rs10515635	5	149,402,113	*HMGXB3*	0.15	5.06×10^{-10}
	rs57091804	5	149,402,905	*HMGXB3*	0.15	5.06×10^{-10}
	rs10068365	5	149,403,100	*HMGXB3*	0.15	5.06×10^{-10}
	rs58265464	5	149,408,929	*HMGXB3*	0.15	1.46×10^{-10}
	rs1469690	5	149,409,773	*HMGXB3*	0.15	1.46×10^{-10}
	rs2278393	5	149,410,153	*HMGXB3*	0.14	1.37×10^{-9}
	rs2278394	5	149,410,519	*HMGXB3*	0.16	6.85×10^{-12}
	rs6890492	5	149,412,778	*HMGXB3*	0.16	1.10×10^{-11}
	rs11167493	5	149,413,441	*HMGXB3*	0.16	9.79×10^{-12}
	rs714099	5	149,413,659	*HMGXB3*	0.16	7.18×10^{-12}
	rs12523182	5	149,414,017	*HMGXB3*	0.16	9.79×10^{-12}
	rs55756417	5	149,414,652	*HMGXB3*	0.17	3.38×10^{-12}
	rs17110845	5	149,415,095	*HMGXB3*	0.17	3.38×10^{-12}
	rs12519384	5	149,415,558	*HMGXB3*	0.17	3.38×10^{-12}
	rs6887999	5	149,417,138	*HMGXB3*	0.16	1.29×10^{-10}
	rs6888440	5	149,417,552	*HMGXB3*	0.16	1.29×10^{-10}
	rs10062594	5	149,418,126	*HMGXB3*	0.16	1.29×10^{-10}
	rs10440733	5	149,419,188	*HMGXB3*	0.15	1.82×10^{-10}
	rs6860276	5	149,419,792	*HMGXB3*	0.15	1.82×10^{-10}
	rs4705410	5	149,419,991	*HMGXB3*	0.15	1.82×10^{-10}
	rs2304070	5	149,420,698	*HMGXB3*	0.17	3.38×10^{-12}
	rs2276982	5	149,421,502	*HMGXB3*	0.16	9.79×10^{-12}
	rs58861317	5	149,425,788	*HMGXB3*	0.16	1.29×10^{-10}
	rs6894582	5	149,425,903	*HMGXB3*	0.16	1.29×10^{-10}
	rs145571340	5	149,426,307	*HMGXB3*	0.15	2.26×10^{-9}
	rs7715409	5	149,427,543	*HMGXB3*	0.16	1.94×10^{-11}
	rs216129	5	149,430,657	*HMGXB3*	0.09	3.82×10^{-8}
	rs216130	5	149,430,915	*HMGXB3*	0.09	8.53×10^{-9}
	rs4705411	5	149,431,025	*HMGXB3*	0.13	2.51×10^{-11}
	rs1062069	5	149,432,613	*HMGXB3*	0.15	6.61×10^{-11}
	rs3828609	5	149,432,863	*CSF1R*	0.16	5.62×10^{-13}
	rs6885913	5	149,436,598	*CSF1R*	0.18	8.18×10^{-13}
	rs4705412	5	149,438,377	*CSF1R*	0.16	1.12×10^{-11}
	rs3733672	5	149,439,494	*CSF1R*	0.15	7.67×10^{-11}
	rs12516676	5	149,440,045	*CSF1R*	0.18	8.18×10^{-13}
	rs12518674	5	149,442,231	*CSF1R*	0.15	3.35×10^{-10}
	rs12518632	5	149,442,232	*CSF1R*	0.15	2.35×10^{-10}
	rs2282806	5	149,442,474	*CSF1R*	0.15	6.59×10^{-11}
	rs2282805	5	149,442,548	*CSF1R*	0.13	6.33×10^{-10}
	rs11738211	5	149,442,779	*CSF1R*	0.18	8.18×10^{-13}
	rs10476929	5	149,443,670	*CSF1R*	0.15	1.47×10^{-10}
	rs2282804	5	149,445,345	*CSF1R*	0.16	6.49×10^{-12}
	rs216146	5	149,445,921	*CSF1R*	0.10	5.26×10^{-10}
	rs7719910	5	149,445,987	*CSF1R*	0.18	4.21×10^{-14}
	rs216150	5	149,447,628	*CSF1R*	0.10	2.07×10^{-9}
	rs3776074	5	149,451,326	*CSF1R*	0.12	2.83×10^{-9}
	rs13188584	5	149,459,574	*CSF1R*	0.15	8.27×10^{-10}
EFNA1	rs61853583	10	56,231,150	*PCDH15*	0.36	5.39×10^{-9}

	rs61324884	10	56,234,123	*PCDH15*	0.36	5.39×10^{-9}
	rs11004335	10	56,245,720	*PCDH15*	0.36	5.37×10^{-9}
	rs11004337	10	56,249,445	*PCDH15*	0.36	5.37×10^{-9}
	rs11045738	12	21,239,970	*SLCO1B7*	0.29	4.41×10^{-9}
	rs7330507	13	60,125,652	Intergenic	0.48	1.70×10^{-8}
	rs77863572	13	60,127,130	Intergenic	0.46	3.40×10^{-8}
	rs80346741	14	101,762,339	Intergenic	0.33	2.95×10^{-8}
EFNA3	rs587668	4	20,190,972	Intergenic	0.46	3.92×10^{-8}
	rs677198	4	20,195,830	Intergenic	0.49	5.70×10^{-9}
	rs613798	4	20,198,845	Intergenic	0.46	3.92×10^{-8}
	rs2575080	4	20,200,817	Intergenic	0.46	3.92×10^{-8}
	rs593770	4	20,201,089	Intergenic	0.46	3.92×10^{-8}
	rs3113804	4	20,234,411	Intergenic	0.44	4.21×10^{-8}
	rs665559	4	20,235,325	Intergenic	0.44	4.21×10^{-8}
	rs574666	4	20,236,850	Intergenic	0.44	4.21×10^{-8}
	rs526398	4	20,238,979	Intergenic	0.43	2.24×10^{-8}
	rs618286	4	20,238,988	Intergenic	0.43	2.24×10^{-8}
	rs117289532	19	48,077,320	Intergenic	0.88	2.37×10^{-8}
EGF	rs76256974	8	116,582,284	*TRPS1*	0.03	3.35×10^{-8}
	rs3135758	10	123,277,869	*FGFR2*	0.03	4.19×10^{-9}
	rs61934888	12	132,122,806	Intergenic	0.03	2.39×10^{-8}
EGFR	rs76088979	10	78,003,251	*C10orf11*	0.20	3.71×10^{-8}
	rs16933093	10	78,005,660	*C10orf11*	0.20	3.71×10^{-8}
	rs77953595	10	78,006,551	*C10orf11*	0.20	3.71×10^{-8}
EIF4B	rs41307147	6	74,527,043	*CD109*	18.30	1.10×10^{-8}
	rs192149131	10	7,299,082	*SFMBT2*	16.22	3.54×10^{-8}
	rs181906364	10	7,299,083	*SFMBT2*	16.22	3.54×10^{-8}
	rs185686171	10	7,299,085	*SFMBT2*	16.22	3.54×10^{-8}
	rs144071120	19	54,585,335	Intergenic	18.90	4.16×10^{-8}
	rs145149488	19	54,591,697	Intergenic	21.10	5.74×10^{-10}
	rs78522590	19	54,592,755	Intergenic	21.74	3.71×10^{-9}
	rs79060636	19	54,592,896	Intergenic	20.61	9.95×10^{-9}
	rs189820066	19	54,593,226	Intergenic	20.61	9.95×10^{-9}
	rs148439599	19	54,593,558	Intergenic	20.04	8.54×10^{-9}
	rs118149072	19	54,593,579	Intergenic	20.61	9.95×10^{-9}
	rs117045873	19	54,593,681	Intergenic	20.74	1.17×10^{-8}
	rs118026095	19	54,593,725	Intergenic	20.74	1.17×10^{-8}
	rs142554342	19	54,593,797	Intergenic	20.61	9.95×10^{-9}
	rs75603280	19	54,594,671	Intergenic	20.61	9.95×10^{-9}
	rs117195689	19	54,594,897	Intergenic	20.61	9.95×10^{-9}
	rs150414044	19	54,594,976	Intergenic	20.61	9.95×10^{-9}
	rs138139207	19	54,595,065	Intergenic	21.14	9.13×10^{-9}
	rs116939840	19	54,595,337	Intergenic	20.61	9.95×10^{-9}
	rs117291682	19	54,595,351	Intergenic	20.61	9.95×10^{-9}
	rs117637006	19	54,595,526	Intergenic	20.61	9.95×10^{-9}
	rs75625913	19	54,596,164	Intergenic	20.61	9.95×10^{-9}
	rs117982154	19	54,596,270	Intergenic	20.61	9.95×10^{-9}
	rs191155753	19	54,596,882	Intergenic	20.61	9.95×10^{-9}
	rs78928237	19	54,597,720	Intergenic	20.61	9.95×10^{-9}

	rs118027525	19	54,597,983	*OSCAR*	20.61	9.95×10^{-9}
	rs140133395	19	54,598,048	*OSCAR*	20.61	9.95×10^{-9}
EIF4E	rs17112028	10	98,430,561	*PIK3AP1*	5.75	4.37×10^{-8}
	rs17112030	10	98,430,592	*PIK3AP1*	5.75	4.37×10^{-8}
	rs74153705	10	98,431,319	*PIK3AP1*	5.75	4.37×10^{-8}
	rs7910144	10	98,432,671	*PIK3AP1*	5.75	4.37×10^{-8}
	rs7895244	10	98,432,703	*PIK3AP1*	5.75	4.37×10^{-8}
EPOR	rs71498475	9	85,401,997	Intergenic	-0.30	1.08×10^{-8}
	rs34460378	9	85,403,095	Intergenic	-0.30	1.08×10^{-8}
F2R	rs117417471	10	56,973,794	Intergenic	1.28	4.91×10^{-8}
	rs75335471	14	70,248,423	*SLC10A1*	1.25	7.02×10^{-9}
	rs75927735	15	86,169,103	*AKAP13*	1.29	7.40×10^{-11}
	rs76204898	15	86,294,126	Intergenic	1.19	1.98×10^{-10}
	rs8041288	15	86,311,593	*KLHL25*	1.21	5.57×10^{-10}
	rs35582838	15	86,312,294	*KLHL25*	1.21	5.57×10^{-10}
	rs77768079	15	86,314,419	*KLHL25*	1.21	5.57×10^{-10}
	rs12324250	15	86,319,712	*KLHL25*	1.08	4.12×10^{-8}
	rs76587220	15	86,322,550	*KLHL25*	1.21	5.57×10^{-10}
	rs80251970	15	86,325,940	*KLHL25*	1.21	5.57×10^{-10}
	rs113962703	15	86,330,332	*KLHL25*	1.03	1.41×10^{-8}
	rs16944491	15	86,330,886	*KLHL25*	1.21	5.57×10^{-10}
	rs16944522	15	86,332,128	*KLHL25*	1.21	5.57×10^{-10}
	rs75890212	15	86,337,055	*KLHL25*	1.21	7.53×10^{-10}
	rs79719545	15	86,337,648	*KLHL25*	1.21	7.53×10^{-10}
	rs111578069	15	86,338,013	*KLHL25*	1.21	1.25×10^{-9}
	rs75595815	15	86,338,955	Intergenic	1.21	7.53×10^{-10}
FGF2	rs17200502	3	16,891,069	Intergenic	0.24	3.87×10^{-8}
	rs13103403	4	123,612,272	Intergenic	-0.19	2.63×10^{-10}
	rs13101575	4	123,617,561	Intergenic	-0.19	1.80×10^{-10}
	rs376131	4	123,629,225	Intergenic	-0.19	1.62×10^{-10}
	rs13129352	4	123,633,736	Intergenic	-0.21	7.11×10^{-12}
	rs13110096	4	123,633,932	Intergenic	-0.21	7.11×10^{-12}
	rs368797	4	123,635,211	Intergenic	-0.16	8.36×10^{-10}
	rs381910	4	123,635,717	Intergenic	-0.16	9.75×10^{-10}
	rs391783	4	123,637,725	Intergenic	-0.21	1.41×10^{-11}
	rs309343	4	123,643,385	Intergenic	-0.16	8.36×10^{-10}
	rs309344	4	123,644,812	Intergenic	-0.21	7.11×10^{-12}
	rs13120869	4	123,646,152	Intergenic	-0.21	7.11×10^{-12}
	rs309348	4	123,646,391	Intergenic	-0.16	8.36×10^{-10}
	rs6845853	4	123,648,601	Intergenic	-0.16	4.62×10^{-9}
	rs309355	4	123,652,029	*CETN4P*	-0.21	1.11×10^{-12}
	rs13138426	4	123,652,580	*CETN4P*	-0.21	7.11×10^{-12}
	rs309357	4	123,653,576	*CETN4P*	-0.16	8.36×10^{-10}
	rs309358	4	123,653,926	*BBS12*	-0.21	7.11×10^{-12}
	rs309359	4	123,654,522	*BBS12*	-0.16	1.65×10^{-9}
	rs1512975	4	123,657,342	*BBS12*	-0.21	7.11×10^{-12}
	rs309361	4	123,657,978	*BBS12*	-0.21	6.73×10^{-12}
	rs309362	4	123,658,154	*BBS12*	-0.21	7.11×10^{-12}
	rs309363	4	123,658,169	*BBS12*	-0.21	5.47×10^{-12}

rs309364	4	123,658,859	*BBS12*	-0.21	5.47×10^{-12}
rs309365	4	123,659,343	*BBS12*	-0.21	7.11×10^{-12}
rs309366	4	123,661,408	*BBS12*	-0.21	5.47×10^{-12}
rs309367	4	123,662,211	*BBS12*	-0.21	5.47×10^{-12}
rs444145	4	123,662,583	*BBS12*	-0.21	4.36×10^{-12}
rs439110	4	123,662,586	*BBS12*	-0.21	6.41×10^{-12}
rs309368	4	123,662,997	*BBS12*	-0.21	7.11×10^{-12}
rs309370	4	123,664,204	*BBS12*	-0.16	1.24×10^{-9}
rs13135766	4	123,664,427	*BBS12*	-0.21	4.33×10^{-12}
rs13135778	4	123,664,446	*BBS12*	-0.21	7.11×10^{-12}
rs13135445	4	123,664,457	*BBS12*	-0.22	5.16×10^{-14}
rs13102440	4	123,664,919	*BBS12*	-0.21	7.11×10^{-12}
rs309386	4	123,665,991	*BBS12*	-0.16	9.55×10^{-10}
rs7690409	4	123,666,757	Intergenic	-0.16	1.41×10^{-9}
rs309385	4	123,666,760	Intergenic	-0.16	1.41×10^{-9}
rs12644866	4	123,667,604	Intergenic	-0.16	9.55×10^{-10}
rs309384	4	123,668,300	Intergenic	-0.16	9.55×10^{-10}
rs309382	4	123,669,772	Intergenic	-0.16	9.55×10^{-10}
rs13117125	4	123,670,824	Intergenic	-0.16	9.55×10^{-10}
rs13122636	4	123,671,045	Intergenic	-0.16	9.55×10^{-10}
rs13146929	4	123,671,151	Intergenic	-0.21	7.11×10^{-12}
rs309379	4	123,671,456	Intergenic	-0.16	9.55×10^{-10}
rs6811183	4	123,671,551	Intergenic	-0.16	4.12×10^{-10}
rs9990804	4	123,671,984	Intergenic	-0.16	9.55×10^{-10}
rs13103818	4	123,672,516	Intergenic	-0.16	4.12×10^{-10}
rs10016979	4	123,673,192	Intergenic	-0.16	9.55×10^{-10}
rs7690133	4	123,673,809	Intergenic	-0.16	1.28×10^{-9}
rs7673455	4	123,674,034	Intergenic	-0.16	9.55×10^{-10}
rs7695379	4	123,674,099	Intergenic	-0.15	5.27×10^{-9}
rs67050821	4	123,674,383	Intergenic	-0.21	7.11×10^{-12}
rs1849430	4	123,674,701	Intergenic	-0.16	9.55×10^{-10}
rs1849431	4	123,674,762	Intergenic	-0.16	9.55×10^{-10}
rs13138520	4	123,675,483	Intergenic	-0.21	7.11×10^{-12}
rs309377	4	123,676,353	Intergenic	-0.16	9.55×10^{-10}
rs6815982	4	123,677,472	Intergenic	-0.22	5.16×10^{-14}
rs4833255	4	123,678,231	Intergenic	-0.16	1.24×10^{-9}
rs4833256	4	123,678,289	Intergenic	-0.16	1.24×10^{-9}
rs13142203	4	123,679,002	Intergenic	-0.24	1.54×10^{-15}
rs6853540	4	123,679,848	Intergenic	-0.24	1.72×10^{-15}
rs2034460	4	123,679,913	Intergenic	-0.17	7.12×10^{-11}
rs309376	4	123,680,191	Intergenic	-0.17	7.12×10^{-11}
rs309372	4	123,682,639	Intergenic	-0.17	7.12×10^{-11}
rs34518400	4	123,684,778	Intergenic	-0.22	3.07×10^{-13}
rs11737284	4	123,688,888	Intergenic	-0.23	5.76×10^{-14}
rs2122307	4	123,688,968	Intergenic	-0.23	3.64×10^{-16}
rs10084869	4	123,696,907	Intergenic	-0.23	6.72×10^{-16}
rs10019442	4	123,700,613	Intergenic	-0.23	2.69×10^{-16}
rs10019552	4	123,700,726	Intergenic	-0.22	1.30×10^{-14}
rs13102478	4	123,702,041	Intergenic	-0.23	1.85×10^{-14}

	rs13108771	4	123,706,024	Intergenic	-0.23	2.07×10^{-14}
	rs71631415	4	123,707,721	Intergenic	-0.24	1.35×10^{-16}
	rs13122694	4	123,707,937	Intergenic	-0.24	1.35×10^{-16}
	rs13130243	4	123,708,911	Intergenic	-0.23	3.79×10^{-16}
	rs13138349	4	123,710,297	Intergenic	-0.23	1.85×10^{-14}
	rs7673491	4	123,710,875	Intergenic	-0.23	2.69×10^{-16}
	rs13145003	4	123,711,260	Intergenic	-0.23	2.69×10^{-16}
	rs10005301	4	123,715,055	Intergenic	-0.23	2.69×10^{-16}
	rs732676	4	123,717,476	Intergenic	-0.24	1.35×10^{-16}
	rs34518478	4	123,719,999	Intergenic	-0.23	1.85×10^{-14}
	rs13127908	4	123,720,933	Intergenic	-0.24	1.35×10^{-16}
	rs13128246	4	123,721,306	Intergenic	-0.23	1.85×10^{-14}
	rs7678199	4	123,724,239	Intergenic	-0.23	1.40×10^{-15}
	rs308447	4	123,738,546	Intergenic	-0.20	1.54×10^{-13}
	rs13137174	4	123,739,937	Intergenic	-0.23	4.01×10^{-14}
	rs308393	4	123,746,619	Intergenic	-0.19	2.94×10^{-8}
	rs2922979	4	123,748,616	*FGF2*	-0.25	4.24×10^{-20}
	rs308403	4	123,757,748	*FGF2*	-0.25	1.06×10^{-19}
FGF8	rs1495371	3	55,734,673	*ERC2*	0.11	4.47×10^{-8}
	rs12763589	10	134,262,247	Intergenic	0.10	1.99×10^{-9}
FN1	rs149336594	1	164,569,576	*PBX1*	0.68	1.26×10^{-8}
	rs76084430	1	164,573,875	*PBX1*	0.68	1.26×10^{-8}
	rs76850880	1	164,574,281	*PBX1*	0.68	1.26×10^{-8}
	rs111524522	1	164,575,599	*PBX1*	0.68	1.26×10^{-8}
	rs12127332	1	164,597,407	*PBX1*	0.66	2.26×10^{-8}
	rs7546174	1	164,995,745	Intergenic	0.62	2.57×10^{-8}
	rs36113415	1	167,300,638	*POU2F1*	0.70	9.33×10^{-9}
	rs858542	1	167,467,782	*CD247*	0.66	4.77×10^{-8}
	rs7514352	1	168,355,221	Intergenic	0.64	2.24×10^{-8}
	rs78578111	1	171,615,334	*MYOC*	0.70	2.25×10^{-8}
	rs72717889	1	171,656,294	Intergenic	0.72	9.11×10^{-9}
	rs146228539	1	171,997,318	*DNM3*	0.66	3.92×10^{-8}
	rs75874321	1	172,002,661	*DNM3*	0.66	3.92×10^{-8}
	rs41263724	1	172,007,376	*DNM3*	0.66	3.92×10^{-8}
	rs75531597	1	172,008,716	*DNM3*	0.66	3.92×10^{-8}
	rs79947128	1	172,011,395	*DNM3*	0.66	3.92×10^{-8}
	rs114380887	1	172,017,490	*DNM3*	0.66	3.92×10^{-8}
	rs79946145	1	172,020,587	*DNM3*	0.66	3.92×10^{-8}
	rs186343417	1	172,025,621	*DNM3*	0.70	1.25×10^{-8}
	rs80055675	1	172,040,839	*DNM3*	0.66	3.92×10^{-8}
	rs189271639	1	172,201,885	*DNM3*	0.68	2.36×10^{-8}
	rs79295525	1	177,978,859	*LOC730102*	0.59	2.40×10^{-8}
	rs2457581	1	178,457,349	Intergenic	0.73	1.42×10^{-9}
	rs3767181	1	180,152,242	*QSOX1*	0.74	2.91×10^{-9}
	rs3753796	1	180,164,615	*QSOX1*	0.68	3.04×10^{-8}
	rs73040489	1	180,168,206	*FLJ23867*	0.68	3.04×10^{-8}
	rs10913945	1	180,169,108	*FLJ23867*	0.68	3.04×10^{-8}
	rs12057162	1	180,169,949	Intergenic	0.68	3.04×10^{-8}
	rs41268450	1	180,945,052	*STX6*	0.54	1.91×10^{-8}

rs34246004	1	181,144,537	Intergenic	0.63	4.26×10^{-8}
rs72735694	1	184,452,687	*C1orf21*	0.63	4.14×10^{-8}
rs72735697	1	184,472,312	*C1orf21*	0.63	4.14×10^{-8}
rs17379691	1	184,985,263	Intergenic	0.68	7.96×10^{-9}
rs147713217	1	185,010,111	Intergenic	0.61	3.38×10^{-9}
rs12563184	1	187,415,069	Intergenic	0.64	5.31×10^{-9}
rs73067363	1	187,422,856	Intergenic	0.64	5.31×10^{-9}
rs115715580	1	187,883,107	Intergenic	0.62	1.45×10^{-8}
rs139575192	1	187,883,883	Intergenic	0.62	1.45×10^{-8}
rs74924399	1	187,885,556	Intergenic	0.62	1.45×10^{-8}
rs78858501	1	187,889,836	Intergenic	0.62	1.45×10^{-8}
rs116022184	1	187,890,912	Intergenic	0.62	1.45×10^{-8}
rs80341925	1	187,892,569	Intergenic	0.62	1.45×10^{-8}
rs11802712	1	187,894,191	Intergenic	0.63	1.17×10^{-8}
rs113828939	1	187,895,630	Intergenic	0.63	1.17×10^{-8}
rs12071106	1	187,895,938	Intergenic	0.63	1.17×10^{-8}
rs12126409	1	187,895,942	Intergenic	0.63	1.17×10^{-8}
rs147819641	1	187,896,530	Intergenic	0.62	2.03×10^{-8}
rs12084007	1	187,901,778	Intergenic	0.63	1.04×10^{-8}
rs111688623	1	191,429,759	Intergenic	0.65	2.03×10^{-8}
rs1936674	1	192,317,769	*RGS21*	0.64	1.59×10^{-8}
rs142108784	1	193,844,681	Intergenic	0.67	1.17×10^{-8}
rs144303499	1	193,844,682	Intergenic	0.67	1.17×10^{-8}
rs149014981	1	196,319,931	*KCNT2*	0.65	4.70×10^{-8}
rs45617133	1	198,776,670	Intergenic	0.73	4.88×10^{-9}
rs74137227	1	198,784,306	*MIR181A1HG*	0.73	4.88×10^{-9}
rs11581055	1	198,785,309	*MIR181A1HG*	0.73	4.88×10^{-9}
rs16843870	1	198,787,116	*MIR181A1HG*	0.73	4.88×10^{-9}
rs768035	1	198,792,641	*MIR181A1HG*	0.73	4.88×10^{-9}
rs58313139	1	198,797,617	*MIR181A1HG*	0.76	2.31×10^{-9}
rs74137228	1	198,801,630	*MIR181A1HG*	0.74	4.78×10^{-9}
rs4141743	1	198,807,259	*MIR181A1HG*	0.74	4.78×10^{-9}
rs7550566	1	198,809,666	*MIR181A1HG*	0.74	4.78×10^{-9}
rs74134903	1	198,810,628	*MIR181A1HG*	0.74	4.78×10^{-9}
rs74134904	1	198,810,667	*MIR181A1HG*	0.74	4.78×10^{-9}
rs74134906	1	198,812,355	*MIR181A1HG*	0.74	4.78×10^{-9}
rs115725120	1	203,397,372	Intergenic	0.61	3.37×10^{-8}
rs12408558	1	203,601,502	*ATP2B4*	0.53	4.15×10^{-8}
rs74434569	1	204,188,658	*PLEKHA6*	0.83	1.86×10^{-10}
rs45492196	1	204,434,438	*PIK3C2B*	0.69	2.08×10^{-8}
rs12143020	1	205,897,408	*SLC26A9*	0.67	1.03×10^{-8}
rs12164617	1	205,898,644	*SLC26A9*	0.69	5.38×10^{-9}
rs12135835	1	205,900,216	*SLC26A9*	0.69	5.38×10^{-9}
rs41305064	1	205,902,242	*SLC26A9*	0.68	1.11×10^{-8}
rs74739480	1	205,905,276	*SLC26A9*	0.68	1.11×10^{-8}
rs75422336	1	205,906,901	*SLC26A9*	0.68	1.11×10^{-8}
rs79129786	1	208,212,056	*PLXNA2*	0.64	1.20×10^{-8}
rs74957903	1	209,513,968	Intergenic	0.70	8.23×10^{-9}
rs75085117	1	209,514,761	Intergenic	0.70	8.23×10^{-9}

rs79126955	1	211,671,409	Intergenic	0.67	4.26×10^{-8}
rs75347938	1	215,047,801	Intergenic	0.69	2.22×10^{-8}
rs17042153	1	215,051,178	Intergenic	0.69	2.22×10^{-8}
rs186460432	1	215,052,747	Intergenic	0.69	2.22×10^{-8}
rs141218710	1	215,053,983	Intergenic	0.69	2.22×10^{-8}
rs17023610	1	215,059,554	Intergenic	0.69	2.22×10^{-8}
rs79590827	1	215,062,744	Intergenic	0.69	2.22×10^{-8}
rs76009568	1	215,063,753	Intergenic	0.69	2.22×10^{-8}
rs1336948	1	215,068,688	Intergenic	0.69	2.22×10^{-8}
rs17023635	1	215,069,367	Intergenic	0.69	2.22×10^{-8}
rs114335567	1	215,071,242	Intergenic	0.69	2.22×10^{-8}
rs138291070	1	216,935,692	*ESRRG*	0.59	1.22×10^{-8}
rs78488479	1	219,610,897	Intergenic	0.69	2.43×10^{-9}
rs77739064	1	219,653,292	Intergenic	0.82	3.67×10^{-11}
rs77135156	1	222,965,534	Intergenic	0.67	4.77×10^{-8}
rs59615821	1	225,025,480	Intergenic	0.68	8.00×10^{-9}
rs74935577	1	228,994,317	Intergenic	0.67	3.73×10^{-8}
rs12037495	1	229,838,715	Intergenic	0.67	4.75×10^{-9}
rs74142810	1	229,839,317	Intergenic	0.67	4.75×10^{-9}
rs12044686	1	229,843,408	Intergenic	0.65	8.67×10^{-9}
rs16850138	1	229,845,060	Intergenic	0.65	8.67×10^{-9}
rs4847021	1	230,959,298	Intergenic	0.65	1.72×10^{-8}
rs12407450	1	230,995,107	*C1orf198*	0.65	2.96×10^{-8}
rs77274688	1	232,186,522	Intergenic	0.65	1.64×10^{-8}
rs12139739	1	232,792,783	Intergenic	0.68	1.30×10^{-8}
rs55900018	1	233,012,675	Intergenic	0.64	4.47×10^{-8}
rs55770257	1	233,012,803	Intergenic	0.64	4.47×10^{-8}
rs56409532	1	233,012,991	Intergenic	0.64	4.47×10^{-8}
rs72757855	1	233,019,044	Intergenic	0.66	1.85×10^{-8}
rs58562010	1	233,020,507	Intergenic	0.66	2.87×10^{-8}
rs56065775	1	233,022,255	Intergenic	0.66	2.87×10^{-8}
rs74730468	1	233,173,214	*PCNXL2*	0.68	3.73×10^{-8}
rs74506265	1	233,173,744	*PCNXL2*	0.68	3.73×10^{-8}
rs4274054	1	235,779,725	*GNG4*	0.69	1.47×10^{-8}
rs79379588	1	238,754,098	Intergenic	0.71	2.41×10^{-9}
rs114571786	1	238,756,643	Intergenic	0.69	3.71×10^{-9}
rs74728354	1	238,761,549	Intergenic	0.69	3.71×10^{-9}
rs59627173	1	238,762,069	Intergenic	0.68	4.47×10^{-9}
rs74985156	1	238,762,444	Intergenic	0.69	3.71×10^{-9}
rs4659879	1	238,762,517	Intergenic	0.68	4.47×10^{-9}
rs4659534	1	238,762,549	Intergenic	0.68	4.47×10^{-9}
rs78318745	1	238,764,235	Intergenic	0.71	2.09×10^{-9}
rs77760738	1	238,768,875	Intergenic	0.70	2.66×10^{-9}
rs4659903	1	239,298,202	Intergenic	0.80	2.67×10^{-10}
rs78278538	1	239,586,677	Intergenic	0.70	9.06×10^{-9}
rs77665737	1	243,088,833	Intergenic	0.62	3.25×10^{-8}
rs184276665	1	245,985,292	*SMYD3*	0.72	9.96×10^{-9}
rs6672861	1	247,025,738	*AHCTF1*	0.58	6.97×10^{-10}
rs10888281	1	248,113,026	*OR2L8; OR2L13*	0.67	3.05×10^{-8}

rs3927659	1	248,115,884	*OR2L13*	0.75	1.97×10^{-9}
rs138688946	1	248,121,566	*OR2L13*	0.67	2.53×10^{-8}
rs6587408	1	248,122,312	*OR2L13*	0.74	3.29×10^{-9}
rs116660969	1	248,232,035	*OR2L13*	0.70	7.40×10^{-9}
rs146005063	1	248,232,075	*OR2L13*	0.70	7.40×10^{-9}
rs4471301	1	248,232,530	*OR2L13*	0.71	8.48×10^{-9}
rs115484985	1	248,233,073	*OR2L13*	0.81	3.97×10^{-10}
rs188697725	1	248,236,523	*OR2L13*	0.73	4.52×10^{-9}
rs193191589	1	248,236,524	*OR2L13*	0.73	4.52×10^{-9}
rs140976623	1	248,238,987	*OR2L13*	0.79	7.06×10^{-10}
rs145052285	1	248,239,012	*OR2L13*	0.79	7.06×10^{-10}
rs12611657	2	11,110,986	Intergenic	0.70	1.14×10^{-8}
rs13023820	2	47,143,052	*MCFD2*	0.68	1.60×10^{-8}
rs35073682	2	73,273,059	*SFXN5*	0.63	3.22×10^{-8}
rs56170128	2	111,742,068	*ACOXL*	0.67	2.84×10^{-8}
rs72834562	2	111,745,313	*ACOXL*	0.67	2.84×10^{-8}
rs76056446	2	111,747,449	*ACOXL*	0.67	2.84×10^{-8}
rs67234811	2	111,748,471	*ACOXL*	0.67	2.84×10^{-8}
rs72834571	2	111,750,743	*ACOXL*	0.67	2.84×10^{-8}
rs72834576	2	111,754,545	*ACOXL*	0.67	2.84×10^{-8}
rs72834578	2	111,755,381	*ACOXL*	0.67	2.84×10^{-8}
rs67250604	2	111,756,913	*ACOXL*	0.67	2.84×10^{-8}
rs72834584	2	111,757,071	*ACOXL*	0.67	2.84×10^{-8}
rs66509834	2	111,757,099	*ACOXL*	0.67	2.84×10^{-8}
rs1384791	2	111,757,442	*ACOXL*	0.67	2.84×10^{-8}
rs59486976	2	111,762,409	*ACOXL*	0.67	2.84×10^{-8}
rs68080118	2	111,763,417	*ACOXL*	0.67	2.84×10^{-8}
rs72834594	2	111,764,337	*ACOXL*	0.67	2.84×10^{-8}
rs72946240	2	111,764,395	*ACOXL*	0.67	2.84×10^{-8}
rs72834595	2	111,765,336	*ACOXL*	0.67	2.84×10^{-8}
rs1384794	2	111,768,193	*ACOXL*	0.67	2.84×10^{-8}
rs7420839	2	111,768,423	*ACOXL*	0.67	2.84×10^{-8}
rs55833189	2	111,768,485	*ACOXL*	0.67	2.84×10^{-8}
rs6730256	2	111,771,811	*ACOXL*	0.67	2.84×10^{-8}
rs56105404	2	111,771,893	*ACOXL*	0.67	2.84×10^{-8}
rs61145952	2	111,777,279	*ACOXL*	0.67	2.84×10^{-8}
rs75932453	2	145,086,473	*GTDC1*	0.67	3.64×10^{-8}
rs115578534	2	145,118,293	Intergenic	0.72	7.06×10^{-9}
rs114878534	2	145,120,773	Intergenic	0.69	1.91×10^{-8}
rs13024167	2	216,146,204	Intergenic	0.66	3.08×10^{-8}
rs76447806	3	62,507,973	*CADPS*	0.58	3.41×10^{-8}
rs2568839	3	71,274,820	*FOXP1*	0.70	1.03×10^{-8}
rs189283961	3	79,898,011	Intergenic	0.61	8.59×10^{-9}
rs9883715	3	129,123,803	*EFCAB12*	0.78	6.35×10^{-11}
rs6794947	3	130,934,108	*NEK11*	0.64	1.97×10^{-8}
rs6807331	3	130,934,115	*NEK11*	0.68	6.34×10^{-9}
rs6794775	3	130,934,122	*NEK11*	0.70	2.20×10^{-9}
rs13078961	3	137,687,685	Intergenic	0.68	1.43×10^{-8}
rs9831917	3	144,108,879	Intergenic	0.74	7.87×10^{-9}

rs139990714	3	148,709,130	Intergenic	0.72	5.98×10^{-9}
rs73866929	3	148,710,123	*GYG1*	0.75	2.22×10^{-9}
rs6776112	3	148,710,903	*GYG1*	0.75	2.22×10^{-9}
rs59698464	3	148,714,887	*GYG1*	0.75	2.22×10^{-9}
rs58928967	3	148,715,114	*GYG1*	0.75	2.22×10^{-9}
rs7647629	3	148,820,658	Intergenic	0.75	2.22×10^{-9}
rs57249105	3	149,792,830	Intergenic	0.63	1.89×10^{-8}
rs66547256	3	149,795,863	Intergenic	0.63	1.89×10^{-8}
rs73157086	3	149,796,120	Intergenic	0.63	1.89×10^{-8}
rs67452147	3	149,796,705	Intergenic	0.63	1.89×10^{-8}
rs57649841	3	149,797,370	Intergenic	0.76	4.31×10^{-10}
rs6440647	3	149,801,354	Intergenic	0.63	7.89×10^{-9}
rs60629227	3	149,804,510	Intergenic	0.61	1.70×10^{-8}
rs7637247	3	149,807,158	Intergenic	0.63	8.36×10^{-9}
rs17423099	4	11,414,095	*HS3ST1*	0.72	2.54×10^{-9}
rs13135334	4	14,798,393	Intergenic	0.66	2.38×10^{-9}
rs73806008	4	27,936,017	Intergenic	0.58	3.11×10^{-8}
rs3978140	4	132,645,886	Intergenic	0.69	1.33×10^{-8}
rs150051783	4	160,444,249	Intergenic	0.67	5.66×10^{-9}
rs10214354	5	114,015,002	Intergenic	0.65	2.03×10^{-8}
rs78503839	5	120,484,882	Intergenic	0.61	1.83×10^{-8}
rs6887838	5	121,109,221	Intergenic	0.67	4.42×10^{-9}
rs73791866	5	121,109,539	Intergenic	0.67	4.42×10^{-9}
rs56013991	5	121,110,001	Intergenic	0.67	4.42×10^{-9}
rs7723698	5	145,453,133	Intergenic	0.67	4.76×10^{-8}
rs138516705	5	145,620,060	*RBM27*	0.69	2.86×10^{-8}
rs56667063	5	164,982,360	Intergenic	0.66	2.19×10^{-8}
rs73344225	5	164,989,490	Intergenic	0.64	3.27×10^{-8}
rs112571602	6	15,218,273	Intergenic	0.60	4.33×10^{-9}
rs115649989	6	32,431,488	Intergenic	0.63	4.71×10^{-8}
rs10948332	6	47,081,781	Intergenic	0.66	2.51×10^{-8}
rs74769936	6	47,322,096	Intergenic	0.58	3.15×10^{-8}
rs9369677	6	47,323,254	Intergenic	0.69	1.03×10^{-9}
rs9381536	6	47,325,161	Intergenic	0.63	7.12×10^{-9}
rs9296545	6	47,325,611	Intergenic	0.69	1.03×10^{-9}
rs7741773	6	47,327,079	Intergenic	0.69	1.03×10^{-9}
rs9369678	6	47,332,915	Intergenic	0.56	4.65×10^{-8}
rs9381542	6	47,334,713	Intergenic	0.56	4.65×10^{-8}
rs12661444	6	47,335,216	Intergenic	0.56	4.65×10^{-8}
rs4422623	6	47,344,280	Intergenic	0.56	4.92×10^{-8}
rs9369680	6	47,345,591	Intergenic	0.58	2.80×10^{-8}
rs9395253	6	47,348,783	Intergenic	0.56	4.92×10^{-8}
rs77652558	6	47,488,433	*CD2AP*	0.69	6.25×10^{-9}
rs76152299	6	47,542,679	*CD2AP*	0.67	1.28×10^{-8}
rs72873836	6	49,602,190	*RHAG*	0.67	4.93×10^{-9}
rs151062730	6	49,760,770	Intergenic	0.66	3.02×10^{-8}
rs35289230	6	84,718,901	Intergenic	0.70	2.64×10^{-8}
rs10455443	6	86,018,723	Intergenic	0.65	2.49×10^{-8}
rs71570828	6	117,224,212	*RFX6*	0.63	1.60×10^{-8}

rs17210773	6	117,271,489	Intergenic	0.60	3.08×10^{-8}
rs13212970	6	117,417,056	Intergenic	0.59	4.34×10^{-8}
rs144514109	6	128,619,539	*PTPRK*	0.62	3.13×10^{-8}
rs62425725	6	128,742,089	*PTPRK*	0.65	2.15×10^{-8}
rs72990913	6	129,455,490	*LAMA2*	0.76	9.17×10^{-10}
rs62428442	6	133,286,067	Intergenic	0.61	4.97×10^{-8}
rs77748084	6	144,048,299	*PHACTR2*	0.62	1.55×10^{-8}
rs78228935	6	144,050,074	*PHACTR2*	0.74	7.31×10^{-10}
rs73579888	6	144,051,801	*PHACTR2*	0.74	7.31×10^{-10}
rs112853525	6	144,052,488	*PHACTR2*	0.74	7.31×10^{-10}
rs111412914	6	144,052,585	*PHACTR2*	0.72	1.30×10^{-9}
rs77297840	6	144,094,414	*PHACTR2*	0.75	5.48×10^{-10}
rs7453812	6	153,405,199	*RGS17*	0.71	6.24×10^{-9}
rs76779653	6	161,608,252	*AGPAT4*	0.58	2.22×10^{-8}
rs118093961	6	161,634,420	*AGPAT4*	0.74	2.76×10^{-10}
rs62447743	7	9,039,330	Intergenic	0.76	3.88×10^{-10}
rs75967089	7	9,071,034	Intergenic	0.70	1.17×10^{-8}
rs62438781	7	9,072,918	Intergenic	0.67	2.39×10^{-8}
rs7799292	7	9,250,808	Intergenic	0.60	4.54×10^{-8}
rs78165033	7	9,262,726	Intergenic	0.63	2.94×10^{-8}
rs75380079	7	70,118,887	*AUTS2*	0.65	3.36×10^{-8}
rs117449388	7	71,865,742	*CALN1*	0.66	3.13×10^{-8}
rs73137258	7	78,461,166	*MAGI2*	0.56	8.70×10^{-9}
rs113886669	7	78,462,096	*MAGI2*	0.58	5.70×10^{-9}
rs73137287	7	78,468,543	*MAGI2*	0.57	8.87×10^{-9}
rs17455327	7	78,469,638	*MAGI2*	0.57	8.87×10^{-9}
rs73137294	7	78,472,503	*MAGI2*	0.57	8.87×10^{-9}
rs6951658	7	78,474,434	*MAGI2*	0.57	8.87×10^{-9}
rs6951989	7	78,474,634	*MAGI2*	0.57	8.87×10^{-9}
rs17390355	7	78,479,384	*MAGI2*	0.57	8.87×10^{-9}
rs73143027	7	78,479,732	*MAGI2*	0.57	8.87×10^{-9}
rs117038961	7	78,482,899	*MAGI2*	0.54	2.43×10^{-8}
rs6957268	7	78,484,313	*MAGI2*	0.54	2.43×10^{-8}
rs141650299	7	78,484,930	*MAGI2*	0.54	2.43×10^{-8}
rs142475793	7	78,486,327	*MAGI2*	0.54	2.43×10^{-8}
rs117190204	7	78,488,499	*MAGI2*	0.54	2.43×10^{-8}
rs73143039	7	78,492,050	*MAGI2*	0.54	2.43×10^{-8}
rs73143040	7	78,492,107	*MAGI2*	0.54	2.43×10^{-8}
rs73143043	7	78,494,960	*MAGI2*	0.54	2.43×10^{-8}
rs138002846	7	78,498,316	*MAGI2*	0.53	3.58×10^{-8}
rs73143055	7	78,499,447	*MAGI2*	0.54	2.43×10^{-8}
rs150944644	7	78,500,502	*MAGI2*	0.54	2.43×10^{-8}
rs73143059	7	78,501,805	*MAGI2*	0.54	2.43×10^{-8}
rs73143070	7	78,504,838	*MAGI2*	0.54	2.43×10^{-8}
rs73143084	7	78,509,543	*MAGI2*	0.54	2.43×10^{-8}
rs113459111	7	78,510,769	*MAGI2*	0.53	3.18×10^{-8}
rs7789403	7	78,513,786	*MAGI2*	0.55	1.51×10^{-8}
rs7790077	7	78,514,243	*MAGI2*	0.56	1.17×10^{-8}
rs73144911	7	78,514,783	*MAGI2*	0.56	1.17×10^{-8}

rs7801273	7	78,517,523	*MAGI2*	0.55	1.81×10^{-8}
rs138424419	7	78,518,070	*MAGI2*	0.55	1.81×10^{-8}
rs145334411	7	78,520,514	*MAGI2*	0.55	1.81×10^{-8}
rs111296157	7	78,523,064	*MAGI2*	0.55	1.81×10^{-8}
rs73144917	7	78,525,452	*MAGI2*	0.56	1.17×10^{-8}
rs17469166	7	78,528,822	*MAGI2*	0.55	1.38×10^{-8}
rs17151649	7	78,537,119	*MAGI2*	0.54	1.98×10^{-8}
rs12112897	7	78,537,885	*MAGI2*	0.53	3.32×10^{-8}
rs73173756	7	101,014,050	*COL26A1*	0.65	7.52×10^{-9}
rs17643058	8	18,354,207	Intergenic	0.64	3.30×10^{-8}
rs79160222	8	18,358,216	Intergenic	0.64	3.55×10^{-8}
rs10097130	8	58,071,298	Intergenic	0.67	1.03×10^{-8}
rs2733726	8	77,727,127	*ZFHX4*	0.63	9.12×10^{-9}
rs2733727	8	77,727,516	*ZFHX4*	0.63	9.12×10^{-9}
rs76060276	8	77,737,131	*ZFHX4*	0.62	9.58×10^{-9}
rs78040517	8	89,819,870	Intergenic	0.80	8.47×10^{-11}
rs139191195	8	101,640,398	*SNX31*	0.68	3.58×10^{-8}
rs13282227	8	135,467,446	Intergenic	0.69	2.48×10^{-10}
rs77317861	8	136,303,364	*LOC286094*	0.65	1.38×10^{-8}
rs111656004	8	142,211,156	Intergenic	0.63	2.39×10^{-8}
rs7860723	9	6,656,619	Intergenic	0.61	2.89×10^{-8}
rs112382558	9	6,657,785	Intergenic	0.61	2.89×10^{-8}
rs117253196	9	18,416,216	Intergenic	0.71	9.57×10^{-9}
rs115108225	9	21,594,061	Intergenic	0.69	2.48×10^{-8}
rs34331573	9	31,989,742	Intergenic	0.64	3.34×10^{-8}
rs116897696	9	78,896,852	*PCSK5*	0.65	3.93×10^{-8}
rs11139445	9	84,485,690	Intergenic	0.58	1.40×10^{-8}
rs1323769	9	85,825,804	Intergenic	0.69	1.35×10^{-8}
rs2378656	9	85,870,424	*FRMD3*	0.66	3.15×10^{-8}
rs186328302	9	108,135,047	*SLC44A1*	0.73	5.06×10^{-10}
rs151220028	9	112,096,628	Intergenic	0.70	3.24×10^{-9}
rs972776	9	112,107,823	Intergenic	0.72	1.90×10^{-9}
rs76067522	9	129,467,424	Intergenic	0.73	6.44×10^{-10}
rs34778470	9	129,474,661	Intergenic	0.70	1.39×10^{-9}
rs17593794	9	138,518,203	*GLT6D1*	0.60	4.94×10^{-8}
rs187250631	10	6,840,729	*LINC00707*	0.82	7.03×10^{-10}
rs17339635	10	14,487,154	Intergenic	0.72	9.85×10^{-10}
rs11033556	11	4,747,517	Intergenic	0.67	2.56×10^{-8}
rs72923838	11	34,067,866	Intergenic	0.66	3.31×10^{-8}
rs113897841	11	34,082,842	*CAPRIN1*	0.68	1.96×10^{-8}
rs182807187	11	62,880,585	*SLC22A24*	0.64	8.10×10^{-9}
rs17149462	11	68,683,774	*IGHMBP2*	0.68	1.24×10^{-8}
rs80068116	11	68,733,360	Intergenic	0.70	8.49×10^{-9}
rs112934532	11	68,733,800	Intergenic	0.70	8.49×10^{-9}
rs11228432	11	68,743,990	Intergenic	0.70	8.49×10^{-9}
rs12364038	11	68,786,138	Intergenic	0.67	4.04×10^{-8}
rs12790927	11	76,429,796	*GUCY2EP*	0.65	4.06×10^{-8}
rs150227494	11	89,431,458	*FOLH1B*	0.54	3.24×10^{-8}
rs561590	11	111,047,295	Intergenic	0.68	2.38×10^{-8}

rs112568086	12	45,461,201	Intergenic	0.59	7.59×10^{-9}
rs117049824	12	45,464,311	Intergenic	0.59	7.59×10^{-9}
rs56739303	12	45,465,477	Intergenic	0.59	7.59×10^{-9}
rs74080767	12	45,469,374	Intergenic	0.59	7.59×10^{-9}
rs78081649	12	45,469,842	Intergenic	0.59	7.59×10^{-9}
rs117053246	12	45,474,410	Intergenic	0.59	7.59×10^{-9}
rs117076294	12	45,476,517	Intergenic	0.59	7.59×10^{-9}
rs118080386	12	45,476,941	Intergenic	0.59	7.59×10^{-9}
rs150639746	12	45,478,296	Intergenic	0.59	7.59×10^{-9}
rs118048013	12	45,485,493	Intergenic	0.59	7.59×10^{-9}
rs56292866	12	45,489,207	Intergenic	0.59	7.59×10^{-9}
rs74080208	12	45,490,369	Intergenic	0.59	7.59×10^{-9}
rs74909886	12	45,492,590	Intergenic	0.59	7.59×10^{-9}
rs117583035	12	45,493,080	Intergenic	0.59	7.59×10^{-9}
rs75558935	12	45,494,940	Intergenic	0.59	7.59×10^{-9}
rs74080215	12	45,496,680	Intergenic	0.59	7.59×10^{-9}
rs2042098	12	45,496,855	Intergenic	0.57	1.45×10^{-8}
rs116864104	12	45,497,356	Intergenic	0.59	7.59×10^{-9}
rs74080216	12	45,498,195	Intergenic	0.59	7.59×10^{-9}
rs79438849	12	45,498,932	Intergenic	0.59	7.59×10^{-9}
rs75280014	12	45,499,403	Intergenic	0.59	7.59×10^{-9}
rs75028921	12	45,499,530	Intergenic	0.57	1.45×10^{-8}
rs12300558	12	45,499,748	Intergenic	0.57	1.45×10^{-8}
rs12313919	12	45,500,336	Intergenic	0.57	1.45×10^{-8}
rs78209723	12	45,501,480	Intergenic	0.57	1.45×10^{-8}
rs12306609	12	45,503,474	Intergenic	0.58	1.06×10^{-8}
rs79113099	12	45,504,359	Intergenic	0.60	5.39×10^{-9}
rs7969620	12	45,507,203	Intergenic	0.57	1.75×10^{-8}
rs35390084	12	71,078,528	*PTPRR*	0.75	1.83×10^{-9}
rs73183752	12	104,830,632	Intergenic	0.68	1.99×10^{-9}
rs10850126	12	113,492,978	Intergenic	0.68	3.57×10^{-9}
rs11066490	12	113,516,446	*DTX1*	0.62	4.82×10^{-8}
rs73233032	12	127,063,711	Intergenic	0.64	1.58×10^{-8}
rs78774634	13	26,268,359	*ATP8A2*	0.74	2.79×10^{-9}
rs12876957	13	27,035,753	Intergenic	0.68	2.93×10^{-8}
rs140598505	13	35,318,120	Intergenic	0.64	1.53×10^{-8}
rs75638019	13	47,354,463	*ESD*	0.69	2.45×10^{-8}
rs61948301	13	47,369,199	*ESD*	0.69	2.45×10^{-8}
rs117772372	13	47,394,754	Intergenic	0.68	4.59×10^{-8}
rs61948307	13	47,405,736	*HTR2A*	0.70	2.10×10^{-8}
rs989959	13	75,422,522	Intergenic	0.66	1.68×10^{-8}
rs74559132	13	78,375,712	Intergenic	0.67	4.08×10^{-8}
rs75716101	13	78,378,790	Intergenic	0.67	4.08×10^{-8}
rs17778592	13	78,384,414	Intergenic	0.67	4.08×10^{-8}
rs9544634	13	78,495,934	*EDNRB*	0.69	2.05×10^{-8}
rs7358970	13	78,501,165	*EDNRB*	0.69	2.05×10^{-8}
rs77156815	13	78,505,585	*EDNRB*	0.69	2.05×10^{-8}
rs13378946	13	78,510,001	*EDNRB*	0.69	2.05×10^{-8}
rs147500560	13	78,511,320	*EDNRB*	0.69	2.05×10^{-8}

rs7491671	13	78,522,040	*EDNRB*	0.69	2.05×10^{-8}
rs9544646	13	78,531,839	*EDNRB*	0.69	2.05×10^{-8}
rs9530714	13	78,598,087	*LINC00446*	0.69	2.05×10^{-8}
rs9544682	13	78,638,833	*RNF219-AS1*	0.68	4.18×10^{-8}
rs9600959	13	78,642,191	*RNF219-AS1*	0.68	4.18×10^{-8}
rs9544685	13	78,647,303	*RNF219-AS1*	0.68	4.18×10^{-8}
rs9544686	13	78,649,666	*RNF219-AS1*	0.68	4.18×10^{-8}
rs9600960	13	78,653,341	*RNF219-AS1*	0.68	4.18×10^{-8}
rs9593272	13	78,656,249	*RNF219-AS1*	0.68	4.18×10^{-8}
rs9544693	13	78,662,484	*RNF219-AS1*	0.68	4.18×10^{-8}
rs9593273	13	78,672,260	*RNF219-AS1*	0.68	4.18×10^{-8}
rs17188711	13	93,563,911	Intergenic	0.58	2.46×10^{-8}
rs28498169	14	56,956,283	Intergenic	0.61	3.92×10^{-8}
rs8013503	14	56,957,478	Intergenic	0.61	3.92×10^{-8}
rs190683432	14	105,703,160	*BRF1*	0.61	2.57×10^{-8}
rs56267093	15	85,167,756	Intergenic	0.65	2.06×10^{-8}
rs75762958	15	85,177,464	*SCAND2P*	0.65	2.06×10^{-8}
rs72750865	15	85,180,800	*SCAND2P*	0.65	2.06×10^{-8}
rs66810580	15	85,198,350	Intergenic	0.65	2.06×10^{-8}
rs17598561	15	85,198,606	*NMB*	0.65	2.06×10^{-8}
rs1470807	15	87,328,117	*AGBL1*	0.70	1.62×10^{-9}
rs74517834	15	87,338,509	*AGBL1*	0.69	2.02×10^{-9}
rs11638907	15	92,590,756	*SLCO3A1*	0.68	5.87×10^{-9}
rs1060205	15	92,708,528	*SLCO3A1*	0.64	3.37×10^{-8}
rs56255214	16	1,026,166	*LMF1*	0.75	2.94×10^{-9}
rs3809673	16	1,029,912	*LMF1*	0.73	5.87×10^{-9}
rs72769452	16	1,035,591	*SOX8*	0.71	4.49×10^{-9}
rs74802497	16	6,030,025	Intergenic	0.58	4.38×10^{-8}
rs113285677	16	6,032,530	Intergenic	0.60	3.10×10^{-8}
rs116885412	16	8,606,507	Intergenic	0.72	8.13×10^{-10}
rs62020598	16	8,607,892	Intergenic	0.69	1.95×10^{-9}
rs114295607	16	10,764,486	*TEKT5*	0.73	3.60×10^{-11}
rs116456718	16	10,766,215	*TEKT5*	0.73	3.60×10^{-11}
rs17762887	16	10,768,084	*TEKT5*	0.73	3.60×10^{-11}
rs76655408	16	10,768,154	*TEKT5*	0.65	6.57×10^{-10}
rs79842190	16	10,768,185	*TEKT5*	0.65	6.57×10^{-10}
rs79225660	16	10,768,362	*TEKT5*	0.73	3.60×10^{-11}
rs117536175	16	10,770,878	*TEKT5*	0.72	2.07×10^{-10}
rs149367713	16	10,771,340	*TEKT5*	0.66	1.56×10^{-9}
rs16957526	16	10,774,378	*TEKT5*	0.77	2.09×10^{-11}
rs56071143	16	88,479,046	Intergenic	0.75	2.06×10^{-9}
rs80287397	16	88,480,148	Intergenic	0.75	2.06×10^{-9}
rs74556181	16	88,482,961	Intergenic	0.73	3.88×10^{-9}
rs76435067	16	88,483,007	Intergenic	0.73	3.88×10^{-9}
rs77767220	16	88,483,317	Intergenic	0.73	3.88×10^{-9}
rs77330683	16	88,485,006	Intergenic	0.73	3.88×10^{-9}
rs79291559	16	88,487,275	Intergenic	0.73	3.45×10^{-9}
rs55913039	16	88,489,283	Intergenic	0.63	4.40×10^{-8}
rs79081332	16	88,573,383	*ZFPM1*	0.75	1.94×10^{-9}

rs116292333	17	1,490,973	*SLC43A2*	0.74	7.23×10^{-10}
rs72820332	17	1,493,771	*SLC43A2*	0.68	4.40×10^{-9}
rs74702959	17	5,809,373	*LOC339166*	0.68	1.09×10^{-8}
rs17714829	17	48,452,481	*EME1*	0.65	3.02×10^{-9}
rs34927998	17	56,030,583	*CUEDC1*	0.72	1.54×10^{-9}
rs34778976	17	56,041,839	Intergenic	0.72	1.93×10^{-9}
rs12451676	17	56,044,061	Intergenic	0.76	4.29×10^{-10}
rs12953049	17	56,046,783	Intergenic	0.63	1.34×10^{-8}
rs12451572	17	56,046,844	Intergenic	0.70	2.92×10^{-9}
rs12942122	17	56,052,859	*VEZF1*	0.72	1.60×10^{-9}
rs12939279	17	56,056,706	*VEZF1*	0.71	1.83×10^{-9}
rs35999514	17	56,056,968	*VEZF1*	0.70	2.92×10^{-9}
rs12948279	17	56,057,181	*VEZF1*	0.71	1.83×10^{-9}
rs12453137	17	56,057,773	*VEZF1*	0.71	1.83×10^{-9}
rs12943664	17	56,059,883	*VEZF1*	0.70	2.92×10^{-9}
rs2228270	17	56,060,215	*VEZF1*	0.70	3.29×10^{-9}
rs35869410	17	56,060,793	*VEZF1*	0.71	1.83×10^{-9}
rs67275307	17	56,062,440	*VEZF1*	0.70	2.92×10^{-9}
rs35614268	17	56,066,132	Intergenic	0.70	2.92×10^{-9}
rs34649579	17	56,066,168	Intergenic	0.70	2.92×10^{-9}
rs35486142	17	56,071,681	Intergenic	0.70	2.92×10^{-9}
rs8071344	17	56,073,897	Intergenic	0.70	3.29×10^{-9}
rs8072156	17	56,074,128	Intergenic	0.70	3.29×10^{-9}
rs34976719	17	56,074,313	Intergenic	0.64	2.13×10^{-8}
rs12453846	17	56,075,369	Intergenic	0.70	2.92×10^{-9}
rs34474679	17	56,075,519	Intergenic	0.70	2.92×10^{-9}
rs34592492	17	56,079,037	*SRSF1*	0.70	3.29×10^{-9}
rs16942573	17	56,080,449	*SRSF1*	0.72	2.12×10^{-9}
rs34632420	17	56,089,235	Intergenic	0.72	1.60×10^{-9}
rs72839927	17	56,096,704	Intergenic	0.72	1.60×10^{-9}
rs12943228	17	56,098,978	Intergenic	0.72	1.60×10^{-9}
rs12944034	17	56,099,044	Intergenic	0.72	1.60×10^{-9}
rs35125995	17	56,110,445	Intergenic	0.72	1.60×10^{-9}
rs35614671	17	56,114,263	Intergenic	0.71	1.83×10^{-9}
rs66673146	17	56,126,589	Intergenic	0.60	2.15×10^{-8}
rs2585836	17	56,131,755	Intergenic	0.60	2.13×10^{-8}
rs12948186	17	56,175,361	Intergenic	0.75	3.41×10^{-10}
rs35552706	17	56,185,153	Intergenic	0.73	1.17×10^{-9}
rs36089182	17	56,227,116	Intergenic	0.74	7.29×10^{-10}
rs17836650	17	70,995,169	*SLC39A11*	0.66	2.98×10^{-8}
rs116636590	18	14,369,747	Intergenic	0.66	1.23×10^{-8}
rs146523855	18	75,133,589	Intergenic	0.78	1.34×10^{-10}
rs151193621	18	75,133,708	Intergenic	0.74	4.20×10^{-10}
rs73494356	18	75,135,405	Intergenic	0.56	3.35×10^{-8}
rs72993240	18	75,177,038	Intergenic	0.73	2.84×10^{-10}
rs2941814	18	76,568,753	Intergenic	0.70	1.33×10^{-9}
rs10418612	19	777,925	Intergenic	0.73	5.71×10^{-10}
rs10424126	19	777,986	Intergenic	0.73	5.71×10^{-10}
rs111413644	19	33,494,696	*RHPN2*	0.75	2.57×10^{-10}

	rs59559602	19	47,001,057	*PPP5D1*	0.58	6.77×10^{-9}
	rs74838769	19	47,008,425	*PPP5D1*	0.55	1.97×10^{-8}
	rs185099468	19	47,009,279	*PPP5D1*	0.72	8.91×10^{-11}
	rs2124908	19	52,037,559	Intergenic	0.75	9.05×10^{-10}
	rs1457093	19	52,047,847	Intergenic	0.70	2.92×10^{-9}
	rs3752133	19	52,049,899	Intergenic	0.70	2.92×10^{-9}
	rs1840826	19	52,051,381	Intergenic	0.65	1.13×10^{-8}
	rs74539314	19	52,081,522	*ZNF175*	0.70	2.65×10^{-9}
	rs77628825	19	52,081,523	*ZNF175*	0.68	4.88×10^{-9}
	rs34845045	19	55,430,877	Intergenic	0.66	7.96×10^{-9}
	rs11880295	19	55,432,529	Intergenic	0.70	2.86×10^{-9}
	rs4806624	19	55,436,805	*NLRP7*	0.70	2.86×10^{-9}
	rs4806625	19	55,436,977	*NLRP7*	0.70	2.86×10^{-9}
	rs34582807	19	55,437,996	*NLRP7*	0.68	5.25×10^{-9}
	rs12979871	19	55,439,321	*NLRP7*	0.70	2.86×10^{-9}
	rs11879978	19	55,444,231	*NLRP7*	0.76	5.58×10^{-10}
	rs13041373	20	8,115,957	*PLCB1*	0.55	2.97×10^{-8}
	rs148424791	20	19,004,606	Intergenic	0.70	2.80×10^{-10}
	rs149259960	20	22,508,182	Intergenic	0.67	7.36×10^{-9}
	rs73300991	20	22,551,326	*LINC00261*	0.63	3.97×10^{-8}
	rs73145706	20	61,633,711	Intergenic	0.56	3.89×10^{-8}
	rs74629807	21	32,437,494	Intergenic	0.58	2.88×10^{-8}
	rs5992995	22	18,565,727	*PEX26*	0.62	1.38×10^{-8}
	rs5992169	22	18,572,431	*PEX26*	0.63	1.02×10^{-8}
	rs1043278	22	18,573,967	Intergenic	0.61	2.25×10^{-8}
	rs12160860	22	18,575,941	Intergenic	0.61	2.25×10^{-8}
	rs5992997	22	18,576,389	Intergenic	0.61	2.25×10^{-8}
	rs12157397	22	18,578,703	Intergenic	0.63	1.02×10^{-8}
	rs113679997	22	18,580,190	Intergenic	0.65	5.95×10^{-9}
	rs12160466	22	18,580,460	Intergenic	0.62	1.38×10^{-8}
	rs5992174	22	18,580,972	Intergenic	0.63	9.66×10^{-9}
	rs28839	22	18,582,402	Intergenic	0.65	5.95×10^{-9}
	rs467998	22	18,582,650	Intergenic	0.65	5.95×10^{-9}
	rs467937	22	18,583,720	Intergenic	0.62	1.62×10^{-8}
	rs467924	22	18,583,894	Intergenic	0.66	4.77×10^{-9}
	rs468219	22	18,585,577	Intergenic	0.65	6.87×10^{-9}
	rs468235	22	18,585,987	Intergenic	0.66	4.77×10^{-9}
	rs468789	22	18,586,169	Intergenic	0.66	4.77×10^{-9}
	rs5992176	22	18,589,643	Intergenic	0.66	4.77×10^{-9}
	rs462187	22	18,591,546	Intergenic	0.66	4.77×10^{-9}
	rs404145	22	18,591,678	Intergenic	0.66	4.77×10^{-9}
	rs425889	22	18,591,891	Intergenic	0.66	4.77×10^{-9}
	rs536730	22	27,719,151	Intergenic	0.73	1.16×10^{-9}
	rs531862	22	27,719,198	Intergenic	0.73	1.16×10^{-9}
	rs6005434	22	27,833,053	Intergenic	0.53	1.17×10^{-8}
	rs5997248	22	27,833,402	Intergenic	0.53	1.17×10^{-8}
	rs6005435	22	27,833,459	Intergenic	0.53	1.17×10^{-8}
	rs55844758	22	31,034,084	*SLC35E4*	0.68	2.64×10^{-8}
GHR	rs34143150	1	22,514,071	Intergenic	1.79	1.39×10^{-8}

rs17356177	1	22,549,764	Intergenic	1.74	2.69×10^{-8}
rs7532303	1	44,864,049	Intergenic	1.65	3.50×10^{-8}
rs12405914	1	44,871,575	*RNF220*	1.65	3.50×10^{-8}
rs77106564	1	44,872,010	*RNF220*	1.65	3.50×10^{-8}
rs77924537	1	44,872,215	*RNF220*	1.65	3.50×10^{-8}
rs12026556	1	70,282,490	*LRRC7*	1.61	2.09×10^{-8}
rs11209550	1	70,286,394	*LRRC7*	1.61	2.09×10^{-8}
rs148813684	1	70,287,359	*LRRC7*	1.62	2.54×10^{-8}
rs77207864	1	70,293,796	*LRRC7*	1.77	2.92×10^{-9}
rs1938581	1	70,294,568	*LRRC7*	1.77	2.92×10^{-9}
rs2175743	1	70,295,901	*LRRC7*	1.77	2.92×10^{-9}
rs78898899	1	70,297,315	*LRRC7*	1.77	2.92×10^{-9}
rs78975854	1	70,304,664	*LRRC7*	1.77	2.92×10^{-9}
rs12024702	1	70,305,436	*LRRC7*	1.77	2.92×10^{-9}
rs79416278	1	70,309,138	*LRRC7*	1.77	2.92×10^{-9}
rs6658642	1	70,311,793	*LRRC7*	1.77	2.92×10^{-9}
rs76484547	1	70,316,902	*LRRC7*	1.77	2.92×10^{-9}
rs1317127	1	70,320,076	*LRRC7*	1.73	5.25×10^{-9}
rs149196274	1	70,321,381	*LRRC7*	1.73	5.25×10^{-9}
rs12048784	1	70,321,965	*LRRC7*	1.73	5.25×10^{-9}
rs12035080	1	70,326,207	*LRRC7*	1.73	5.25×10^{-9}
rs17131060	1	70,329,599	*LRRC7*	1.73	5.25×10^{-9}
rs17131061	1	70,330,376	*LRRC7*	1.73	5.25×10^{-9}
rs75258903	1	70,332,350	*LRRC7*	1.73	5.25×10^{-9}
rs11209566	1	70,333,440	*LRRC7*	1.73	5.25×10^{-9}
rs17131066	1	70,337,409	*LRRC7*	1.73	5.25×10^{-9}
rs17425958	1	102,107,148	Intergenic	1.60	3.68×10^{-8}
rs61804497	1	102,113,725	Intergenic	1.58	4.87×10^{-8}
rs79442714	1	113,833,382	Intergenic	1.73	4.05×10^{-8}
rs12756965	1	113,839,149	Intergenic	1.73	4.05×10^{-8}
rs12045733	1	113,848,704	Intergenic	1.73	4.05×10^{-8}
rs12038527	1	113,848,729	Intergenic	1.73	4.05×10^{-8}
rs4839306	1	113,849,470	Intergenic	1.73	4.05×10^{-8}
rs12048530	1	113,851,139	Intergenic	1.73	4.05×10^{-8}
rs12046318	1	113,854,250	Intergenic	1.73	4.05×10^{-8}
rs35059446	1	113,858,904	Intergenic	1.73	4.05×10^{-8}
rs2226155	1	113,865,778	Intergenic	1.65	1.03×10^{-8}
rs1361765	1	113,866,202	Intergenic	1.65	1.03×10^{-8}
rs12024820	1	113,866,431	Intergenic	1.65	1.03×10^{-8}
rs6674942	1	113,866,749	Intergenic	1.65	1.03×10^{-8}
rs12025961	1	113,867,828	Intergenic	1.65	1.03×10^{-8}
rs12047722	1	113,868,398	Intergenic	1.65	1.03×10^{-8}
rs4838983	1	113,870,537	Intergenic	1.65	1.03×10^{-8}
rs4839309	1	113,871,003	Intergenic	1.65	1.03×10^{-8}
rs142534849	1	181,041,664	Intergenic	1.72	3.24×10^{-8}
rs6673310	1	192,597,985	Intergenic	1.78	2.63×10^{-8}
rs55992816	2	2,509,177	Intergenic	1.79	2.15×10^{-8}
rs60327756	2	16,408,983	Intergenic	1.85	5.46×10^{-9}
rs58629855	2	16,408,984	Intergenic	1.85	5.46×10^{-9}

rs4832659	2	16,419,751	Intergenic	1.74	3.72×10^{-8}
rs62152138	2	103,324,335	*SLC9A2*	1.79	3.32×10^{-8}
rs62175148	2	174,367,561	Intergenic	1.42	4.18×10^{-9}
rs17753758	2	174,381,005	Intergenic	1.36	8.93×10^{-9}
rs17364234	2	214,155,327	*SPAG16*	1.62	2.42×10^{-8}
rs77999946	2	214,263,687	*SPAG16*	1.63	1.96×10^{-8}
rs17701917	2	214,439,806	*SPAG16*	1.52	4.88×10^{-8}
rs189859464	3	1,749,654	Intergenic	1.60	3.33×10^{-9}
rs1534246	3	73,947,467	Intergenic	1.49	2.85×10^{-8}
rs72912050	3	89,776,155	Intergenic	1.69	4.77×10^{-8}
rs12631105	3	89,776,624	Intergenic	1.69	4.77×10^{-8}
rs6781208	3	89,778,038	Intergenic	1.69	4.77×10^{-8}
rs6781309	3	89,778,128	Intergenic	1.69	4.77×10^{-8}
rs79778210	3	89,779,169	Intergenic	1.69	4.77×10^{-8}
rs57036056	3	89,783,903	Intergenic	1.69	4.77×10^{-8}
rs72912072	3	89,784,905	Intergenic	1.69	4.77×10^{-8}
rs10511146	3	89,790,208	Intergenic	1.69	4.77×10^{-8}
rs17027205	3	89,790,574	Intergenic	1.69	4.77×10^{-8}
rs6551423	3	89,792,790	Intergenic	1.69	4.77×10^{-8}
rs6773084	3	89,795,298	Intergenic	1.78	2.24×10^{-8}
rs7430934	3	89,800,760	Intergenic	1.78	2.24×10^{-8}
rs144610471	3	89,801,865	Intergenic	1.78	2.33×10^{-8}
rs76069798	3	89,818,218	Intergenic	1.78	2.24×10^{-8}
rs77479756	3	89,818,982	Intergenic	1.78	2.24×10^{-8}
rs12636191	3	89,823,336	Intergenic	1.78	2.24×10^{-8}
rs111303123	3	89,826,743	Intergenic	1.78	2.24×10^{-8}
rs6771600	3	89,828,209	Intergenic	1.78	2.24×10^{-8}
rs74986556	3	89,830,249	Intergenic	1.78	2.24×10^{-8}
rs76256245	3	89,830,289	Intergenic	1.78	2.24×10^{-8}
rs1915728	3	89,832,810	Intergenic	1.78	2.24×10^{-8}
rs1851051	3	89,840,346	Intergenic	1.78	2.24×10^{-8}
rs17027228	3	89,841,166	Intergenic	1.78	2.24×10^{-8}
rs74541906	3	89,854,686	Intergenic	1.78	2.24×10^{-8}
rs79617218	3	89,858,299	Intergenic	1.78	2.24×10^{-8}
rs6806871	3	89,861,343	Intergenic	1.78	2.24×10^{-8}
rs74718075	3	89,862,089	Intergenic	1.78	2.24×10^{-8}
rs79036643	3	89,862,391	Intergenic	1.78	2.24×10^{-8}
rs150763639	3	89,864,338	Intergenic	1.78	2.24×10^{-8}
rs80172038	3	89,865,683	Intergenic	1.78	2.24×10^{-8}
rs2177058	3	89,880,144	Intergenic	1.78	2.24×10^{-8}
rs6551435	3	89,882,196	Intergenic	1.78	2.24×10^{-8}
rs149571900	3	89,883,366	Intergenic	1.78	2.24×10^{-8}
rs79174609	3	89,884,518	Intergenic	1.78	2.24×10^{-8}
rs9683159	3	89,907,687	Intergenic	1.78	2.24×10^{-8}
rs113766991	3	89,914,798	Intergenic	1.78	2.24×10^{-8}
rs7637958	3	89,925,048	Intergenic	1.78	2.24×10^{-8}
rs76381775	3	89,960,211	Intergenic	1.78	2.24×10^{-8}
rs1949650	3	89,963,168	Intergenic	1.78	2.24×10^{-8}
rs74494604	3	89,967,879	Intergenic	1.78	2.24×10^{-8}

rs79318377	3	144,035,182	Intergenic	1.75	2.32×10^{-8}
rs76121376	3	171,716,427	Intergenic	1.51	1.78×10^{-8}
rs2720910	3	194,734,651	Intergenic	1.57	3.14×10^{-8}
rs116486576	4	7,350,579	*SORCS2*	2.15	3.26×10^{-12}
rs12513387	4	24,243,680	Intergenic	1.72	4.23×10^{-8}
rs186976037	4	31,817,981	Intergenic	1.68	1.05×10^{-9}
rs13129886	4	130,922,726	Intergenic	1.92	7.38×10^{-10}
rs13113563	4	130,929,195	Intergenic	1.92	7.38×10^{-10}
rs13147782	4	131,034,260	Intergenic	1.90	9.04×10^{-9}
rs10000443	4	131,064,600	Intergenic	1.74	1.15×10^{-8}
rs10011007	4	131,064,690	Intergenic	1.81	4.51×10^{-9}
rs28592799	4	131,065,258	Intergenic	1.81	4.51×10^{-9}
rs10008557	4	131,072,168	Intergenic	1.81	4.51×10^{-9}
rs11947093	4	131,087,342	Intergenic	1.85	2.89×10^{-9}
rs28376800	4	131,095,591	Intergenic	1.85	2.89×10^{-9}
rs72714251	4	141,075,184	*MAML3*	2.09	9.59×10^{-13}
rs72719967	4	153,222,778	Intergenic	1.80	3.46×10^{-8}
rs7678272	4	153,229,868	Intergenic	1.77	4.18×10^{-8}
rs115973927	4	181,655,290	Intergenic	1.79	4.36×10^{-8}
rs13144644	4	186,663,922	*SORBS2*	1.84	1.82×10^{-9}
rs35991414	4	186,697,952	*SORBS2*	1.74	3.46×10^{-9}
rs185793609	5	30,359,017	Intergenic	1.63	3.50×10^{-8}
rs73246227	5	117,457,849	*LOC102467224*	1.85	1.03×10^{-8}
rs78962704	5	117,463,322	*LOC102467224*	1.77	2.30×10^{-8}
rs111720900	5	117,484,254	*LOC102467224*	1.77	2.30×10^{-8}
rs59291909	5	117,486,947	*LOC102467224*	1.77	2.30×10^{-8}
rs58706630	5	117,488,641	*LOC102467224*	1.77	2.30×10^{-8}
rs1382724	5	117,492,656	*LOC102467224*	1.77	2.30×10^{-8}
rs1382723	5	117,492,859	*LOC102467224*	1.77	2.30×10^{-8}
rs6894503	5	117,492,998	*LOC102467224*	1.77	2.30×10^{-8}
rs73246132	5	117,494,765	*LOC102467224*	1.77	2.30×10^{-8}
rs151145004	5	117,510,361	*LOC102467224*	1.76	2.44×10^{-8}
rs73246174	5	117,510,430	*LOC102467224*	1.76	2.44×10^{-8}
rs73246178	5	117,510,611	*LOC102467224*	1.76	2.44×10^{-8}
rs73246751	5	117,518,968	*LOC102467224*	1.76	2.44×10^{-8}
rs74569203	5	117,521,908	*LOC102467224*	1.71	3.36×10^{-8}
rs17143707	5	117,522,041	*LOC102467224*	1.74	2.56×10^{-8}
rs115412659	5	148,150,080	Intergenic	1.70	4.98×10^{-8}
rs111934886	6	14,339,896	Intergenic	2.13	7.51×10^{-11}
rs45613533	6	37,359,222	*RNF8*	1.70	1.20×10^{-8}
rs73024503	6	162,326,020	*PARK2*	1.55	1.37×10^{-8}
rs76821212	6	165,921,617	*PDE10A*	2.03	2.31×10^{-10}
rs76570644	7	1,178,838	Intergenic	1.90	1.46×10^{-10}
rs189549017	7	10,620,006	Intergenic	1.83	1.08×10^{-8}
rs62446904	7	30,754,538	Intergenic	1.85	1.32×10^{-9}
rs74657816	7	46,670,682	Intergenic	1.76	9.00×10^{-9}
rs35473791	7	78,380,708	*MAGI2*	1.50	2.52×10^{-8}
rs12705407	7	106,817,433	*HBP1*	2.14	5.60×10^{-12}
rs12705408	7	106,871,952	*COG5*	2.09	1.00×10^{-11}

rs11762802	7	106,961,882	*COG5*	2.30	6.62×10^{-13}
rs73207299	8	11,691,145	*FDFT1*	1.54	2.79×10^{-8}
rs73209015	8	11,700,967	*CTSB*	1.54	2.79×10^{-8}
rs7838546	8	31,947,880	*NRG1; NRG1-IT1*	1.50	3.64×10^{-8}
rs112981873	8	74,798,939	Intergenic	1.58	4.12×10^{-8}
rs62518566	8	124,654,036	Intergenic	1.52	2.06×10^{-8}
rs182222543	9	66,272,947	Intergenic	1.48	3.43×10^{-9}
rs4747848	10	10,161,275	Intergenic	2.04	1.32×10^{-11}
rs140459811	10	24,183,910	*KIAA1217*	1.59	4.56×10^{-8}
rs7917505	10	70,974,481	Intergenic	1.91	3.33×10^{-9}
rs10823525	10	72,114,565	*LRRC20*	1.58	2.17×10^{-8}
rs78745769	10	72,115,208	*LRRC20*	1.45	2.33×10^{-8}
rs10823526	10	72,115,475	*LRRC20*	1.48	1.78×10^{-8}
rs10823528	10	72,115,839	*LRRC20*	1.48	1.78×10^{-8}
rs115151048	10	72,121,454	*LRRC20*	1.48	1.78×10^{-8}
rs76702380	10	72,129,054	*LRRC20*	1.48	1.78×10^{-8}
rs2127584	10	72,132,013	*LRRC20*	1.48	1.78×10^{-8}
rs45450199	10	72,182,110	*EIF4EBP2*	1.41	4.27×10^{-8}
rs11592130	10	89,520,241	*ATAD1*	1.81	1.94×10^{-9}
rs11185946	10	91,744,898	Intergenic	1.33	2.29×10^{-8}
rs72819163	10	108,082,813	Intergenic	2.14	9.09×10^{-11}
rs79988456	10	108,446,150	*SORCS1*	1.72	3.60×10^{-8}
rs117723370	10	108,556,637	*SORCS1*	1.76	2.08×10^{-8}
rs17181929	10	108,559,676	*SORCS1*	1.82	1.15×10^{-8}
rs142910305	11	89,332,684	Intergenic	1.65	1.35×10^{-8}
rs2000916	11	106,403,449	Intergenic	1.59	6.96×10^{-9}
rs2000917	11	106,403,663	Intergenic	2.11	3.05×10^{-11}
rs1945075	11	106,406,584	Intergenic	1.47	2.99×10^{-8}
rs35891003	11	106,407,376	Intergenic	2.18	2.40×10^{-11}
rs35591067	11	106,407,476	Intergenic	2.18	2.40×10^{-11}
rs71488218	11	106,408,734	Intergenic	2.18	2.40×10^{-11}
rs111536413	11	106,408,764	Intergenic	1.57	1.25×10^{-8}
rs67340024	11	106,409,125	Intergenic	1.47	2.99×10^{-8}
rs35059716	11	106,409,535	Intergenic	1.47	2.99×10^{-8}
rs1945066	11	106,410,615	Intergenic	2.18	2.40×10^{-11}
rs60781140	11	106,410,810	Intergenic	1.47	2.99×10^{-8}
rs1945065	11	106,411,576	Intergenic	1.47	2.99×10^{-8}
rs1945064	11	106,411,935	Intergenic	1.47	2.99×10^{-8}
rs111372550	11	106,412,481	Intergenic	1.47	2.99×10^{-8}
rs151104176	11	106,413,006	Intergenic	1.47	2.99×10^{-8}
rs112829654	11	106,413,193	Intergenic	2.14	3.45×10^{-11}
rs17105823	11	106,413,556	Intergenic	1.47	2.99×10^{-8}
rs1945063	11	106,414,112	Intergenic	1.47	2.99×10^{-8}
rs1945062	11	106,414,284	Intergenic	1.47	2.99×10^{-8}
rs71488219	11	106,414,727	Intergenic	2.18	2.40×10^{-11}
rs66746934	11	106,415,469	Intergenic	1.47	2.99×10^{-8}
rs35064702	11	106,416,694	Intergenic	2.18	2.40×10^{-11}
rs34917464	11	106,417,187	Intergenic	1.47	2.99×10^{-8}
rs35182337	11	106,418,566	Intergenic	2.18	2.40×10^{-11}

rs35990922	11	106,418,626	Intergenic	1.47	2.99×10^{-8}
rs12795470	11	106,418,877	Intergenic	1.47	2.99×10^{-8}
rs12795694	11	106,418,978	Intergenic	1.47	2.99×10^{-8}
rs7949190	11	124,596,741	Intergenic	1.61	2.53×10^{-8}
rs7949826	11	124,597,044	Intergenic	1.59	3.37×10^{-8}
rs73575692	11	128,723,557	*KCNJ1*	1.75	3.83×10^{-8}
rs113085163	11	128,723,879	*KCNJ1*	1.75	3.83×10^{-8}
rs72472267	11	133,455,513	Intergenic	1.69	3.54×10^{-9}
rs75193006	12	32,361,562	*BICD1*	1.73	1.92×10^{-8}
rs10745477	12	88,741,050	Intergenic	1.55	3.37×10^{-8}
rs10745478	12	88,746,811	Intergenic	1.64	9.37×10^{-9}
rs141073155	12	94,740,098	*CEP83*	1.67	1.19×10^{-8}
rs142713725	13	88,877,310	Intergenic	1.72	1.53×10^{-8}
rs1959395	14	83,928,612	Intergenic	1.69	3.71×10^{-8}
rs75782643	14	90,785,989	*NRDE2*	1.79	4.17×10^{-8}
rs79684894	14	90,791,704	*NRDE2*	1.79	4.17×10^{-8}
rs143398009	14	98,470,614	Intergenic	1.49	4.99×10^{-8}
rs141134139	14	106,800,176	Intergenic	1.56	9.18×10^{-9}
rs35279276	15	56,383,205	*RFX7*	1.69	1.31×10^{-8}
rs34161200	15	56,784,198	Intergenic	1.72	8.78×10^{-9}
rs12899275	15	85,521,127	Intergenic	1.64	3.56×10^{-8}
rs17657561	15	88,003,592	Intergenic	1.78	4.93×10^{-8}
rs80080592	15	92,792,642	Intergenic	1.56	1.29×10^{-8}
rs12050999	16	5,245,550	Intergenic	1.82	1.06×10^{-8}
rs10852650	16	5,251,168	Intergenic	1.69	3.62×10^{-8}
rs10852651	16	5,251,175	Intergenic	1.69	3.62×10^{-8}
rs78649715	16	5,261,545	Intergenic	1.81	1.20×10^{-8}
rs75893813	16	5,264,690	Intergenic	1.81	1.20×10^{-8}
rs60465971	16	5,267,215	Intergenic	1.73	2.78×10^{-8}
rs56256368	16	5,268,538	Intergenic	1.77	2.01×10^{-8}
rs56208627	16	5,268,667	Intergenic	1.77	2.01×10^{-8}
rs9932433	16	5,269,502	Intergenic	1.77	2.01×10^{-8}
rs13331940	16	5,269,820	Intergenic	1.77	2.01×10^{-8}
rs13331989	16	5,269,898	Intergenic	1.77	2.01×10^{-8}
rs9928451	16	5,271,022	Intergenic	1.77	2.01×10^{-8}
rs12232454	16	5,271,209	Intergenic	1.77	2.01×10^{-8}
rs59338239	16	5,271,629	Intergenic	1.77	2.01×10^{-8}
rs74783278	16	5,273,452	Intergenic	1.77	2.01×10^{-8}
rs77004033	16	5,276,073	Intergenic	1.77	2.01×10^{-8}
rs78047823	16	5,276,457	Intergenic	1.77	2.01×10^{-8}
rs117811257	16	5,276,484	Intergenic	1.77	2.01×10^{-8}
rs74249180	16	5,276,532	Intergenic	1.77	2.01×10^{-8}
rs12596315	16	19,488,434	*TMC5*	1.75	8.82×10^{-11}
rs62026182	16	19,519,911	*GDE1*	1.72	2.54×10^{-10}
rs72798056	16	50,184,574	Intergenic	1.62	4.53×10^{-8}
rs181029676	17	64,558,223	*PRKCA*	1.85	5.61×10^{-9}
rs62081992	18	14,257,266	Intergenic	1.59	1.62×10^{-8}
rs6131728	20	15,808,487	*MACROD2*	1.84	2.67×10^{-9}
rs16996627	20	15,808,549	*MACROD2*	1.84	2.67×10^{-9}

	rs6135568	20	15,829,764	*MACROD2*	1.81	4.91×10^{-9}
	rs73898562	20	15,843,015	*MACROD2*	1.65	3.08×10^{-8}
	rs6131734	20	15,846,783	*MACROD2*	1.62	4.78×10^{-8}
	rs12624918	20	15,854,901	*MACROD2*	1.75	1.78×10^{-8}
	rs12624919	20	15,854,910	*MACROD2*	1.63	4.65×10^{-8}
	rs12106174	20	16,508,484	*KIF16B*	1.19	1.76×10^{-8}
	rs11699351	20	30,414,578	*MYLK2*	2.29	2.31×10^{-12}
	rs75200100	20	57,841,857	Intergenic	1.49	2.34×10^{-9}
	rs79138162	20	57,853,496	Intergenic	1.35	1.59×10^{-8}
	rs112716132	22	27,463,211	Intergenic	1.76	1.14×10^{-8}
	rs6006621	22	44,419,407	*PARVB*	1.88	5.88×10^{-9}
GNG12	rs2404215	1	53,634,228	Intergenic	0.25	3.48×10^{-8}
	rs75565762	1	60,841,810	Intergenic	0.22	4.09×10^{-9}
	rs79676211	1	60,842,214	Intergenic	0.22	4.09×10^{-9}
	rs113917246	1	72,176,973	*NEGR1*	0.31	2.55×10^{-10}
	rs72944123	1	72,184,768	*NEGR1*	0.31	2.55×10^{-10}
	rs57583829	1	72,187,974	*NEGR1*	0.31	2.55×10^{-10}
	rs72944128	1	72,191,623	*NEGR1*	0.31	2.55×10^{-10}
	rs72944129	1	72,195,446	*NEGR1*	0.31	2.55×10^{-10}
	rs74782184	1	72,197,134	*NEGR1*	0.31	2.55×10^{-10}
	rs12039538	1	72,198,671	*NEGR1*	0.28	2.22×10^{-9}
	rs72944192	1	72,201,200	*NEGR1*	0.31	2.55×10^{-10}
	rs59851000	1	72,203,126	*NEGR1*	0.31	2.55×10^{-10}
	rs79404144	1	72,206,083	*NEGR1*	0.32	1.46×10^{-10}
	rs12037107	1	72,206,302	*NEGR1*	0.31	2.55×10^{-10}
	rs12030035	1	72,208,083	*NEGR1*	0.31	2.55×10^{-10}
	rs17091704	1	72,208,917	*NEGR1*	0.31	2.55×10^{-10}
	rs72944200	1	72,211,415	*NEGR1*	0.31	2.55×10^{-10}
	rs17091710	1	72,211,943	*NEGR1*	0.31	2.55×10^{-10}
	rs60397116	1	72,212,080	*NEGR1*	0.31	2.55×10^{-10}
	rs72946003	1	72,214,403	*NEGR1*	0.31	2.55×10^{-10}
	rs12043796	1	72,215,029	*NEGR1*	0.31	2.55×10^{-10}
	rs12023733	1	72,215,994	*NEGR1*	0.31	2.55×10^{-10}
	rs12023757	1	72,216,111	*NEGR1*	0.31	2.55×10^{-10}
	rs6671557	1	72,220,321	*NEGR1*	0.32	1.46×10^{-10}
	rs6697610	1	72,224,124	*NEGR1*	0.31	2.55×10^{-10}
	rs1459796	1	72,225,877	*NEGR1*	0.31	2.55×10^{-10}
	rs6424447	1	72,227,685	*NEGR1*	0.31	2.55×10^{-10}
	rs72946013	1	72,229,829	*NEGR1*	0.32	1.37×10^{-10}
	rs75459942	1	72,229,975	*NEGR1*	0.32	1.37×10^{-10}
	rs7541406	1	72,230,600	*NEGR1*	0.32	1.37×10^{-10}
	rs7546909	1	72,231,804	*NEGR1*	0.29	1.02×10^{-9}
	rs72946022	1	72,235,227	*NEGR1*	0.32	1.37×10^{-10}
	rs72946034	1	72,238,267	*NEGR1*	0.32	1.37×10^{-10}
	rs6697370	1	72,238,691	*NEGR1*	0.32	1.37×10^{-10}
	rs12048869	1	72,244,453	*NEGR1*	0.32	1.37×10^{-10}
	rs7528030	1	72,247,277	*NEGR1*	0.32	1.37×10^{-10}
	rs12091362	1	72,249,523	*NEGR1*	0.29	7.25×10^{-10}
	rs12024388	1	72,253,577	*NEGR1*	0.32	1.37×10^{-10}

	rs17091758	1	72,254,435	*NEGR1*	0.32	1.37×10^{-10}
	rs11584866	1	72,255,977	*NEGR1*	0.32	1.37×10^{-10}
	rs11588321	1	72,273,615	*NEGR1; NEGR1-IT1*	0.31	1.50×10^{-10}
	rs57227393	1	72,274,874	*NEGR1; NEGR1-IT1*	0.31	1.50×10^{-10}
	rs57839383	1	72,275,162	*NEGR1; NEGR1-IT1*	0.31	1.50×10^{-10}
	rs6701156	1	72,275,559	*NEGR1; NEGR1-IT1*	0.31	1.50×10^{-10}
	rs12074943	1	72,278,754	*NEGR1; NEGR1-IT1*	0.31	1.50×10^{-10}
	rs11209848	1	72,279,522	*NEGR1; NEGR1-IT1*	0.31	1.50×10^{-10}
	rs12078969	1	72,281,171	*NEGR1; NEGR1-IT1*	0.31	1.50×10^{-10}
	rs11902461	2	101,387,569	Intergenic	0.30	4.81×10^{-10}
	rs72932979	2	159,387,819	*PKP4*	0.23	4.37×10^{-8}
	rs72936956	2	159,416,104	*PKP4*	0.23	4.64×10^{-8}
	rs111619399	2	159,455,245	*PKP4*	0.23	4.64×10^{-8}
	rs147035326	3	40,655,466	Intergenic	0.21	2.18×10^{-8}
	rs79410835	4	155,324,656	*DCHS2*	0.28	1.18×10^{-8}
	rs73854735	4	155,325,552	*DCHS2*	0.28	1.18×10^{-8}
	rs114203084	4	155,326,270	*DCHS2*	0.28	1.18×10^{-8}
	rs114131286	4	155,326,645	*DCHS2*	0.28	1.18×10^{-8}
	rs56155318	4	155,326,731	*DCHS2*	0.28	1.18×10^{-8}
	rs77693626	4	155,328,505	*DCHS2*	0.28	1.18×10^{-8}
	rs73854737	4	155,328,557	*DCHS2*	0.28	1.18×10^{-8}
	rs28423376	4	155,330,056	*DCHS2*	0.28	1.18×10^{-8}
	rs10007868	4	155,330,651	*DCHS2*	0.28	1.18×10^{-8}
	rs10019687	4	155,332,367	*DCHS2*	0.28	1.18×10^{-8}
	rs6839125	4	155,335,578	*DCHS2*	0.28	2.63×10^{-8}
	rs114256572	4	155,335,897	*DCHS2*	0.28	2.63×10^{-8}
	rs6536005	4	155,336,095	*DCHS2*	0.28	2.63×10^{-8}
	rs74588432	6	140,249,098	Intergenic	0.27	1.39×10^{-8}
	rs34231471	7	141,865,701	Intergenic	0.29	1.27×10^{-9}
	rs73174816	13	41,352,622	Intergenic	0.24	4.11×10^{-9}
	rs72646376	13	93,830,757	Intergenic	0.26	3.70×10^{-9}
	rs12597420	16	81,171,330	*PKD1L2*	0.28	9.05×10^{-9}
	rs11150347	16	81,171,411	*PKD1L2*	0.28	9.05×10^{-9}
	rs28575151	16	81,171,903	*PKD1L2*	0.28	9.05×10^{-9}
	rs4889243	16	81,173,030	*PKD1L2*	0.28	9.05×10^{-9}
	rs77104177	16	81,173,051	*PKD1L2*	0.28	9.05×10^{-9}
	rs12918619	16	81,173,136	*PKD1L2*	0.28	9.05×10^{-9}
	rs28670519	16	81,173,741	*PKD1L2*	0.28	1.76×10^{-9}
	rs76564490	16	81,177,874	*PKD1L2*	0.25	4.08×10^{-8}
	rs7203293	16	81,179,280	*PKD1L2*	0.24	3.87×10^{-8}
	rs140500028	16	88,527,691	*ZFPM1*	0.28	1.46×10^{-8}
	rs75778010	19	48,268,900	Intergenic	0.31	1.70×10^{-10}
GNG8	rs143050684	2	161,264,315	*RBMS1*	0.50	4.35×10^{-8}
	rs73018782	2	169,844,794	*ABCB11*	0.55	1.07×10^{-9}
	rs60897043	2	169,845,099	*ABCB11*	0.55	1.07×10^{-9}
	rs28846465	2	169,846,180	*ABCB11*	0.55	1.07×10^{-9}
	rs4148780	2	169,847,203	*ABCB11*	0.55	1.07×10^{-9}
	rs2287613	2	169,847,758	*ABCB11*	0.55	1.07×10^{-9}
	rs61504845	2	169,856,729	*ABCB11*	0.55	1.07×10^{-9}

	rs73020774	2	169,857,087	*ABCB11*	0.55	1.07×10^{-9}
	rs13400716	2	169,857,476	*ABCB11*	0.55	1.07×10^{-9}
	rs6733156	2	169,857,566	*ABCB11*	0.55	1.07×10^{-9}
	rs11898632	2	169,858,577	*ABCB11*	0.55	1.07×10^{-9}
	rs112779928	2	169,859,716	*ABCB11*	0.55	1.07×10^{-9}
	rs2892806	2	169,860,302	*ABCB11*	0.55	1.07×10^{-9}
	rs56706166	2	169,860,690	*ABCB11*	0.55	1.07×10^{-9}
	rs10200158	2	169,860,718	*ABCB11*	0.55	1.07×10^{-9}
	rs11890609	2	169,861,727	*ABCB11*	0.55	1.07×10^{-9}
	rs10196426	2	169,863,259	*ABCB11*	0.55	1.20×10^{-9}
	rs10184113	2	169,863,381	*ABCB11*	0.55	1.07×10^{-9}
	rs73020800	2	169,864,638	*ABCB11*	0.55	1.07×10^{-9}
	rs73022704	2	169,864,985	*ABCB11*	0.55	1.07×10^{-9}
	rs12613034	2	169,866,414	*ABCB11*	0.55	1.07×10^{-9}
	rs6751775	2	169,867,230	*ABCB11*	0.55	1.07×10^{-9}
	rs3770600	2	169,868,253	*ABCB11*	0.55	1.07×10^{-9}
	rs10179758	2	169,868,814	*ABCB11*	0.55	1.07×10^{-9}
	rs6759871	2	169,869,331	*ABCB11*	0.55	1.07×10^{-9}
	rs2241339	2	169,870,295	*ABCB11*	0.55	1.07×10^{-9}
	rs4148776	2	169,870,882	*ABCB11*	0.55	1.07×10^{-9}
	rs10172795	2	169,872,462	*ABCB11*	0.55	1.07×10^{-9}
	rs11892966	2	169,875,736	*ABCB11*	0.55	1.07×10^{-9}
	rs73024744	2	169,877,791	*ABCB11*	0.55	1.07×10^{-9}
	rs13387523	2	169,878,438	*ABCB11*	0.55	3.12×10^{-10}
IGF1R	rs17864188	7	126,945,192	Intergenic	0.26	4.82×10^{-8}
	rs111519389	7	126,950,238	Intergenic	0.26	4.82×10^{-8}
	rs17862354	7	126,962,160	Intergenic	0.26	4.82×10^{-8}
	rs17862357	7	126,973,697	Intergenic	0.26	4.82×10^{-8}
	rs17862363	7	127,001,382	Intergenic	0.26	4.82×10^{-8}
	rs17867837	7	127,006,827	Intergenic	0.26	4.82×10^{-8}
	rs17867838	7	127,007,978	Intergenic	0.26	4.82×10^{-8}
	rs117187446	7	127,066,432	Intergenic	0.27	4.67×10^{-8}
	rs117612403	7	127,066,906	Intergenic	0.27	4.67×10^{-8}
	rs80126810	12	11,346,513	Intergenic	0.28	3.88×10^{-10}
IKBKB	rs112759756	18	6,780,462	Intergenic	7.09	2.51×10^{-8}
	rs118019062	20	41,640,413	*PTPRT*	6.72	7.63×10^{-9}
IKBKG	rs911503	14	36,485,635	Intergenic	1.96	3.12×10^{-8}
IL2RA	rs11762786	7	156,382,428	*LINC01006*	2.97	6.51×10^{-9}
IL4R	rs10824468	10	78,671,628	*KCNMA1*	3.00	4.28×10^{-8}
IL6R	rs112677917	11	40,221,687	*LRRC4C*	6.68	9.76×10^{-11}
	rs72795903	16	28,989,633	*SPNS1*	6.33	2.06×10^{-9}
ITGA11	rs7532303	1	44,864,049	Intergenic	0.31	1.67×10^{-8}
	rs12405914	1	44,871,575	*RNF220*	0.31	1.67×10^{-8}
	rs77106564	1	44,872,010	*RNF220*	0.31	1.67×10^{-8}
	rs77924537	1	44,872,215	*RNF220*	0.31	1.67×10^{-8}
	rs112437613	1	57,647,715	*DAB1*	0.29	3.41×10^{-8}
	rs10889183	1	60,999,977	Intergenic	0.33	4.13×10^{-8}
	rs4454572	1	61,003,101	Intergenic	0.33	4.13×10^{-8}
	rs112593979	1	72,663,293	*NEGR1*	0.35	1.41×10^{-9}

rs11164685	1	103,598,908	Intergenic	0.30	3.03×10^{-8}
rs79442714	1	113,833,382	Intergenic	0.33	5.60×10^{-9}
rs12756965	1	113,839,149	Intergenic	0.33	5.60×10^{-9}
rs12045733	1	113,848,704	Intergenic	0.33	5.60×10^{-9}
rs12038527	1	113,848,729	Intergenic	0.33	5.60×10^{-9}
rs4839306	1	113,849,470	Intergenic	0.33	5.60×10^{-9}
rs12048530	1	113,851,139	Intergenic	0.33	5.60×10^{-9}
rs12046318	1	113,854,250	Intergenic	0.33	5.60×10^{-9}
rs35059446	1	113,858,904	Intergenic	0.33	5.60×10^{-9}
rs142534849	1	181,041,664	Intergenic	0.34	1.60×10^{-9}
rs78878780	1	219,017,425	Intergenic	0.38	3.78×10^{-11}
rs80220156	1	219,017,470	Intergenic	0.38	3.78×10^{-11}
rs17574573	1	219,024,035	Intergenic	0.38	3.78×10^{-11}
rs76709448	1	219,028,085	Intergenic	0.38	3.78×10^{-11}
rs74329588	1	219,030,178	Intergenic	0.38	3.78×10^{-11}
rs80261364	1	219,039,895	Intergenic	0.36	1.15×10^{-10}
rs17574961	1	219,040,761	Intergenic	0.36	1.15×10^{-10}
rs79769748	1	219,042,771	Intergenic	0.36	1.15×10^{-10}
rs79112139	1	219,043,164	Intergenic	0.36	1.15×10^{-10}
rs80121977	1	219,046,348	Intergenic	0.36	1.15×10^{-10}
rs924269	1	219,051,503	Intergenic	0.36	1.15×10^{-10}
rs17575281	1	219,053,082	Intergenic	0.34	3.78×10^{-10}
rs59987725	1	219,055,174	Intergenic	0.35	2.21×10^{-10}
rs115992574	2	14,798,479	Intergenic	0.34	5.07×10^{-10}
rs76404547	2	14,848,655	Intergenic	0.31	3.41×10^{-9}
rs79873290	2	14,956,721	Intergenic	0.34	6.23×10^{-10}
rs75893593	2	32,767,202	*BIRC6*	0.27	2.12×10^{-8}
rs17011993	2	32,769,351	*BIRC6*	0.33	5.28×10^{-10}
rs3917268	2	102,778,862	*IL1R1*	0.32	1.21×10^{-8}
rs3917291	2	102,782,166	*IL1R1*	0.31	1.93×10^{-8}
rs3917314	2	102,790,863	*IL1R1*	0.30	4.43×10^{-8}
rs3917320	2	102,792,875	*IL1R1*	0.31	1.93×10^{-8}
rs3917325	2	102,793,907	*IL1R1*	0.31	1.93×10^{-8}
rs3917328	2	102,794,541	*IL1R1*	0.31	1.93×10^{-8}
rs13003489	2	102,801,502	Intergenic	0.31	1.93×10^{-8}
rs13017584	2	102,803,444	*IL1RL2*	0.31	1.93×10^{-8}
rs13009757	2	102,912,174	Intergenic	0.30	9.56×10^{-9}
rs62152138	2	103,324,335	*SLC9A2*	0.34	5.60×10^{-9}
rs7562520	2	160,019,065	*TANC1*	0.31	3.53×10^{-8}
rs116041633	2	233,240,889	Intergenic	0.31	2.37×10^{-8}
rs56035150	3	2,357,581	*CNTN4*	0.34	7.46×10^{-11}
rs77976373	3	2,372,455	*CNTN4*	0.34	7.46×10^{-11}
rs79007158	3	2,380,198	*CNTN4*	0.34	7.46×10^{-11}
rs75549677	3	2,385,042	*CNTN4*	0.27	7.73×10^{-9}
rs75533829	3	2,410,457	*CNTN4*	0.27	7.80×10^{-9}
rs3913588	3	2,416,252	*CNTN4*	0.28	4.85×10^{-9}
rs17013461	3	2,416,684	*CNTN4*	0.28	4.85×10^{-9}
rs17013465	3	2,416,697	*CNTN4*	0.28	4.85×10^{-9}
rs17013467	3	2,417,521	*CNTN4*	0.28	4.85×10^{-9}

rs17013472	3	2,418,494	*CNTN4*	0.28	4.85×10^{-9}
rs57270584	3	2,419,885	*CNTN4*	0.28	4.85×10^{-9}
rs1827452	3	2,420,109	*CNTN4*	0.28	4.85×10^{-9}
rs17013494	3	2,420,610	*CNTN4*	0.28	4.85×10^{-9}
rs79739371	3	2,421,656	*CNTN4*	0.28	4.85×10^{-9}
rs17013524	3	2,423,127	*CNTN4*	0.28	4.85×10^{-9}
rs78855680	3	2,423,237	*CNTN4*	0.28	4.85×10^{-9}
rs74691141	3	2,424,326	*CNTN4*	0.27	8.23×10^{-9}
rs11926996	3	2,426,089	*CNTN4*	0.29	2.18×10^{-9}
rs74854613	3	32,344,350	*CMTM8*	0.29	3.05×10^{-8}
rs34406434	3	89,432,122	*EPHA3*	0.30	1.69×10^{-8}
rs9880275	3	90,039,009	Intergenic	0.32	1.14×10^{-8}
rs9815846	3	90,040,987	Intergenic	0.32	1.14×10^{-8}
rs7620479	3	90,180,643	Intergenic	0.31	3.09×10^{-9}
rs7428470	3	90,207,606	Intergenic	0.27	2.78×10^{-8}
rs9828400	3	90,214,028	Intergenic	0.28	9.96×10^{-9}
rs6798310	3	90,224,813	Intergenic	0.28	9.96×10^{-9}
rs6787796	3	90,225,446	Intergenic	0.28	9.96×10^{-9}
rs7653160	3	90,242,405	Intergenic	0.28	9.96×10^{-9}
rs7432423	3	90,247,905	Intergenic	0.28	9.96×10^{-9}
rs6803112	3	90,264,768	Intergenic	0.28	9.96×10^{-9}
rs9865441	3	90,266,420	Intergenic	0.28	9.96×10^{-9}
rs7429659	3	90,274,805	Intergenic	0.28	9.96×10^{-9}
rs6551462	3	90,287,332	Intergenic	0.28	9.96×10^{-9}
rs2316811	3	90,313,054	Intergenic	0.28	1.42×10^{-8}
rs112050167	3	90,324,265	Intergenic	0.28	1.27×10^{-8}
rs115336235	3	90,358,752	Intergenic	0.30	4.40×10^{-9}
rs137963600	3	90,360,952	Intergenic	0.28	1.42×10^{-8}
rs149438476	3	90,362,869	Intergenic	0.30	5.70×10^{-9}
rs116179516	3	90,402,280	Intergenic	0.28	1.42×10^{-8}
rs144390463	3	90,420,614	Intergenic	0.28	1.42×10^{-8}
rs150306267	3	90,424,253	Intergenic	0.28	1.42×10^{-8}
rs141135945	3	90,428,237	Intergenic	0.29	5.56×10^{-10}
rs150483042	3	90,431,083	Intergenic	0.31	2.36×10^{-9}
rs184655552	3	90,432,562	Intergenic	0.31	3.14×10^{-9}
rs144677859	3	90,435,679	Intergenic	0.31	2.22×10^{-9}
rs141940565	3	90,435,940	Intergenic	0.31	2.22×10^{-9}
rs35668670	3	90,437,848	Intergenic	0.28	1.42×10^{-8}
rs115126456	3	90,441,404	Intergenic	0.28	1.42×10^{-8}
rs115941982	3	90,468,230	Intergenic	0.28	1.27×10^{-8}
rs58841348	3	90,470,443	Intergenic	0.28	1.27×10^{-8}
rs141308579	3	90,483,384	Intergenic	0.31	2.56×10^{-8}
rs143661400	3	90,483,386	Intergenic	0.31	2.56×10^{-8}
rs116080896	3	90,484,094	Intergenic	0.28	1.29×10^{-8}
rs114196277	3	188,458,771	*LPP*	0.32	1.30×10^{-9}
rs2720915	3	194,722,729	Intergenic	0.29	1.91×10^{-8}
rs2720914	3	194,722,866	Intergenic	0.29	1.91×10^{-8}
rs2720911	3	194,726,720	Intergenic	0.29	1.91×10^{-8}
rs2676832	3	194,727,026	Intergenic	0.29	1.91×10^{-8}

rs114358991	4	1,047,424	Intergenic	0.33	2.63×10^{-8}
rs13128743	4	7,301,089	*SORCS2*	0.33	2.95×10^{-9}
rs10937801	4	7,302,000	*SORCS2*	0.25	1.98×10^{-8}
rs1532665	4	7,303,572	*SORCS2*	0.24	3.17×10^{-8}
rs1532664	4	7,303,726	*SORCS2*	0.24	3.17×10^{-8}
rs1546266	4	7,304,807	*SORCS2*	0.24	4.91×10^{-8}
rs36192013	4	8,740,880	Intergenic	0.31	1.02×10^{-8}
rs7669150	4	16,085,522	*PROM1*	0.27	4.41×10^{-8}
rs55932807	4	90,753,339	*SNCA*	0.30	2.89×10^{-8}
rs79507245	4	102,046,408	*PPP3CA*	0.34	2.36×10^{-9}
rs74675949	4	102,046,629	*PPP3CA*	0.34	2.36×10^{-9}
rs78209620	4	102,047,770	*PPP3CA*	0.34	2.36×10^{-9}
rs78888749	4	102,047,812	*PPP3CA*	0.34	2.36×10^{-9}
rs113609659	4	102,049,484	*PPP3CA*	0.33	4.82×10^{-9}
rs115008357	4	102,050,892	*PPP3CA*	0.34	2.36×10^{-9}
rs142191092	4	102,052,036	*PPP3CA*	0.34	2.36×10^{-9}
rs139686096	4	102,054,847	*PPP3CA*	0.34	2.36×10^{-9}
rs113473645	4	102,057,622	*PPP3CA*	0.34	2.36×10^{-9}
rs111885453	4	102,059,408	*PPP3CA*	0.34	2.36×10^{-9}
rs150119589	4	102,060,085	*PPP3CA*	0.34	2.36×10^{-9}
rs113188556	4	102,060,940	*PPP3CA*	0.34	2.36×10^{-9}
rs113496555	4	102,061,144	*PPP3CA*	0.34	2.36×10^{-9}
rs112039584	4	102,061,563	*PPP3CA*	0.34	2.36×10^{-9}
rs80183680	4	102,061,572	*PPP3CA*	0.34	2.36×10^{-9}
rs116229517	4	102,061,766	*PPP3CA*	0.34	2.36×10^{-9}
rs112122964	4	102,061,822	*PPP3CA*	0.34	2.36×10^{-9}
rs74631130	4	102,062,069	*PPP3CA*	0.34	2.36×10^{-9}
rs111752790	4	102,063,270	*PPP3CA*	0.34	2.36×10^{-9}
rs113744576	4	102,064,126	*PPP3CA*	0.34	2.36×10^{-9}
rs112973280	4	102,064,899	*PPP3CA*	0.34	2.36×10^{-9}
rs112695279	4	102,065,144	*PPP3CA*	0.34	2.36×10^{-9}
rs75372805	4	102,065,533	*PPP3CA*	0.34	2.36×10^{-9}
rs17240082	4	102,065,719	*PPP3CA*	0.34	2.36×10^{-9}
rs113471888	4	102,069,519	*PPP3CA*	0.34	2.36×10^{-9}
rs112522326	4	102,071,777	*PPP3CA*	0.34	2.36×10^{-9}
rs111792710	4	102,073,273	*PPP3CA*	0.34	2.36×10^{-9}
rs192402944	4	129,628,719	Intergenic	0.29	4.58×10^{-8}
rs184741912	4	129,628,720	Intergenic	0.29	4.58×10^{-8}
rs189724000	4	129,628,727	Intergenic	0.29	3.39×10^{-8}
rs13147782	4	131,034,260	Intergenic	0.33	3.88×10^{-8}
rs116135844	4	168,179,343	Intergenic	0.37	4.27×10^{-10}
rs59370581	4	168,846,612	Intergenic	0.36	4.29×10^{-11}
rs115973927	4	181,655,290	Intergenic	0.37	3.48×10^{-10}
rs184754365	4	182,551,359	Intergenic	0.30	2.24×10^{-8}
rs72706718	5	1,884,638	*IRX4*	0.32	2.48×10^{-8}
rs72706719	5	1,885,964	*IRX4*	0.32	2.48×10^{-8}
rs16899797	5	31,804,402	*PDZD2*	0.28	4.53×10^{-8}
rs4867383	5	31,807,304	*PDZD2*	0.29	3.88×10^{-8}
rs72755993	5	41,398,835	*PLCXD3*	0.29	2.31×10^{-9}

rs252751	5	77,389,213	*AP3B1*	0.29	3.30×10^{-8}
rs73246227	5	117,457,849	*LOC102467224*	0.33	2.65×10^{-8}
rs151145004	5	117,510,361	*LOC102467224*	0.32	2.81×10^{-8}
rs73246174	5	117,510,430	*LOC102467224*	0.32	2.81×10^{-8}
rs73246178	5	117,510,611	*LOC102467224*	0.32	2.81×10^{-8}
rs73246751	5	117,518,968	*LOC102467224*	0.32	2.81×10^{-8}
rs17143707	5	117,522,041	*LOC102467224*	0.31	4.60×10^{-8}
rs11948947	5	150,971,790	Intergenic	0.32	2.21×10^{-8}
rs9502434	6	6,326,042	Intergenic	0.31	1.89×10^{-8}
rs9504762	6	6,332,865	Intergenic	0.29	3.90×10^{-8}
rs17379732	6	6,342,180	Intergenic	0.30	2.42×10^{-8}
rs17142071	6	6,342,377	Intergenic	0.30	2.42×10^{-8}
rs111934886	6	14,339,896	Intergenic	0.34	8.49×10^{-9}
rs149324894	6	32,481,795	Intergenic	0.23	1.65×10^{-8}
rs12207336	6	65,882,792	*EYS*	0.31	1.47×10^{-8}
rs79091857	6	115,205,433	Intergenic	0.31	6.63×10^{-9}
rs76821212	6	165,921,617	*PDE10A*	0.34	3.45×10^{-9}
rs62436764	6	167,456,485	Intergenic	0.34	3.15×10^{-9}
rs55782534	7	9,381,826	Intergenic	0.32	4.69×10^{-8}
rs73088242	7	25,197,526	*C7orf31*	0.28	2.96×10^{-8}
rs62446904	7	30,754,538	Intergenic	0.34	1.44×10^{-9}
rs74657816	7	46,670,682	Intergenic	0.34	5.87×10^{-10}
rs73158622	7	139,449,008	*HIPK2*	0.34	1.79×10^{-9}
rs73739645	7	156,044,610	Intergenic	0.31	1.30×10^{-8}
rs11974690	7	156,047,516	Intergenic	0.31	1.30×10^{-8}
rs10234772	7	156,047,717	Intergenic	0.31	1.68×10^{-8}
rs11973416	7	156,047,826	Intergenic	0.31	1.30×10^{-8}
rs57629563	7	156,048,245	Intergenic	0.31	1.30×10^{-8}
rs57178781	7	156,048,937	Intergenic	0.31	1.30×10^{-8}
rs73739653	7	156,049,991	Intergenic	0.31	1.30×10^{-8}
rs11973944	7	156,053,063	Intergenic	0.31	1.30×10^{-8}
rs73739660	7	156,056,493	Intergenic	0.29	3.59×10^{-8}
rs73739661	7	156,056,802	Intergenic	0.29	3.59×10^{-8}
rs11977766	7	156,057,327	Intergenic	0.29	3.59×10^{-8}
rs55693286	7	156,059,923	Intergenic	0.30	2.62×10^{-8}
rs55906846	8	8,922,476	Intergenic	0.31	7.06×10^{-9}
rs11777327	8	8,996,329	*PPP1R3B*	0.30	1.09×10^{-8}
rs17704885	8	8,997,562	*PPP1R3B*	0.30	8.04×10^{-9}
rs4289770	8	10,487,222	*RP1L1*	0.32	6.52×10^{-9}
rs17222373	8	74,546,481	*STAU2*	0.32	4.58×10^{-9}
rs112655948	8	81,512,601	Intergenic	0.31	1.74×10^{-8}
rs75425780	8	81,514,508	Intergenic	0.30	2.20×10^{-8}
rs80217028	8	81,517,118	Intergenic	0.30	2.20×10^{-8}
rs113743401	8	81,517,545	Intergenic	0.30	2.20×10^{-8}
rs79125477	8	81,523,135	Intergenic	0.31	2.08×10^{-8}
rs150034497	8	82,482,289	Intergenic	0.32	4.84×10^{-8}
rs10093136	8	99,401,982	Intergenic	0.32	3.52×10^{-8}
rs72734082	8	136,718,766	Intergenic	0.32	4.33×10^{-9}
rs147486424	9	92,945,616	Intergenic	0.28	1.78×10^{-8}

rs79540864	9	92,948,067	Intergenic	0.27	2.84×10^{-8}
rs147414187	9	92,952,046	Intergenic	0.27	3.66×10^{-8}
rs56244040	9	92,953,458	Intergenic	0.27	3.66×10^{-8}
rs111396481	9	92,967,753	Intergenic	0.27	3.66×10^{-8}
rs112441344	9	92,969,766	Intergenic	0.27	3.66×10^{-8}
rs144166209	9	92,975,418	Intergenic	0.28	1.81×10^{-8}
rs117943175	9	92,979,660	Intergenic	0.27	4.24×10^{-8}
rs74882880	9	92,986,030	Intergenic	0.27	1.90×10^{-8}
rs9696920	9	94,829,588	*SPTLC1*	0.31	4.00×10^{-8}
rs7919350	10	6,363,948	Intergenic	0.32	2.35×10^{-9}
rs78967058	10	54,404,231	Intergenic	0.30	1.48×10^{-8}
rs116983082	10	60,466,398	*BICC1*	0.31	5.71×10^{-9}
rs7917505	10	70,974,481	Intergenic	0.33	2.43×10^{-8}
rs61873932	10	102,903,882	Intergenic	0.28	3.18×10^{-8}
rs61873933	10	102,904,521	Intergenic	0.28	3.18×10^{-8}
rs143946272	10	103,116,650	*BTRC*	0.25	2.72×10^{-8}
rs77691531	10	103,123,526	*BTRC*	0.25	2.72×10^{-8}
rs149480420	10	103,134,103	*BTRC*	0.24	3.63×10^{-8}
rs138970392	10	103,138,438	*BTRC*	0.25	2.72×10^{-8}
rs17760556	10	103,138,615	*BTRC*	0.25	2.72×10^{-8}
rs80151341	10	103,139,960	*BTRC*	0.25	2.72×10^{-8}
rs76838593	10	103,143,264	*BTRC*	0.25	2.72×10^{-8}
rs117932321	10	103,149,531	*BTRC*	0.25	2.72×10^{-8}
rs17760784	10	103,156,416	*BTRC*	0.25	2.72×10^{-8}
rs77334916	10	103,178,507	*BTRC*	0.25	2.72×10^{-8}
rs78810836	10	103,178,514	*BTRC*	0.25	2.72×10^{-8}
rs149161195	10	103,181,917	*BTRC*	0.25	2.72×10^{-8}
rs117998275	10	103,183,891	*BTRC*	0.25	2.72×10^{-8}
rs7900797	10	103,192,220	*BTRC*	0.25	2.72×10^{-8}
rs7098584	10	103,209,336	*BTRC*	0.25	2.72×10^{-8}
rs79528180	10	103,218,179	*BTRC*	0.25	2.72×10^{-8}
rs76114299	10	103,219,315	*BTRC*	0.25	2.72×10^{-8}
rs4387286	10	103,220,793	*BTRC*	0.25	2.72×10^{-8}
rs148303736	10	103,230,149	*BTRC*	0.26	2.09×10^{-8}
rs76807333	10	103,234,534	*BTRC*	0.25	2.72×10^{-8}
rs74415794	10	103,238,858	*BTRC*	0.25	2.72×10^{-8}
rs75820490	10	103,253,308	*BTRC*	0.25	2.72×10^{-8}
rs77012276	10	103,256,869	*BTRC*	0.25	2.72×10^{-8}
rs116952915	10	103,265,796	*BTRC*	0.25	2.72×10^{-8}
rs17767748	10	103,285,900	*BTRC*	0.25	2.72×10^{-8}
rs75919232	10	103,289,312	*BTRC*	0.25	2.72×10^{-8}
rs7073902	10	103,291,893	*BTRC*	0.25	2.72×10^{-8}
rs77209663	10	103,300,166	*BTRC*	0.25	2.72×10^{-8}
rs79010936	10	103,300,909	*BTRC*	0.25	1.93×10^{-8}
rs77325250	10	103,320,638	Intergenic	0.25	2.72×10^{-8}
rs77564076	10	103,320,818	Intergenic	0.25	2.72×10^{-8}
rs79692612	10	103,322,878	Intergenic	0.25	2.72×10^{-8}
rs114076639	10	103,333,323	Intergenic	0.24	3.83×10^{-8}
rs3730478	10	103,339,142	*POLL*	0.24	3.83×10^{-8}

rs3730463	10	103,344,589	*POLL*	0.24	3.83×10^{-8}
rs117416511	10	103,351,588	*DPCD*	0.24	3.83×10^{-8}
rs116357148	10	103,352,082	*DPCD*	0.24	3.83×10^{-8}
rs190065357	10	134,951,797	Intergenic	0.37	1.68×10^{-10}
rs192694504	11	2,215,419	Intergenic	0.36	2.67×10^{-9}
rs57499191	11	42,983,878	Intergenic	0.38	5.13×10^{-11}
rs61904091	11	95,383,824	Intergenic	0.25	1.00×10^{-8}
rs17799724	11	95,387,178	Intergenic	0.25	2.27×10^{-8}
rs1941401	11	114,173,810	*NNMT*	0.30	2.80×10^{-8}
rs1941400	11	114,173,835	*NNMT*	0.30	2.80×10^{-8}
rs11214940	11	114,182,670	*NNMT*	0.30	2.80×10^{-8}
rs76634920	11	114,184,587	Intergenic	0.30	2.80×10^{-8}
rs11611293	12	13,113,491	Intergenic	0.25	3.68×10^{-8}
rs67897587	12	13,293,153	Intergenic	0.43	1.72×10^{-13}
rs76519925	12	83,937,790	Intergenic	0.29	2.41×10^{-8}
rs35480839	12	85,297,523	*SLC6A15*	0.35	2.42×10^{-9}
rs12823911	12	85,320,802	Intergenic	0.34	2.94×10^{-9}
rs1703078	12	89,018,883	Intergenic	0.33	6.70×10^{-9}
rs12372274	12	89,049,195	Intergenic	0.31	4.43×10^{-8}
rs7960533	12	114,087,473	Intergenic	0.31	3.58×10^{-8}
rs16942976	12	114,088,724	Intergenic	0.31	3.58×10^{-8}
rs12312601	12	114,088,737	Intergenic	0.31	3.58×10^{-8}
rs12314110	12	114,088,797	Intergenic	0.31	3.58×10^{-8}
rs28659869	12	114,089,625	Intergenic	0.31	3.58×10^{-8}
rs35188057	12	114,092,777	Intergenic	0.31	3.58×10^{-8}
rs192947014	12	121,365,216	Intergenic	0.31	4.55×10^{-9}
rs10507309	13	22,996,106	Intergenic	0.29	4.56×10^{-8}
rs117463805	13	50,803,819	*DLEU1*	0.31	1.11×10^{-8}
rs72658018	13	113,230,049	*TUBGCP3*	0.32	3.81×10^{-8}
rs116205966	14	37,034,979	Intergenic	0.29	3.50×10^{-8}
rs77290882	14	37,035,012	Intergenic	0.29	3.50×10^{-8}
rs80232634	14	37,038,801	Intergenic	0.29	3.50×10^{-8}
rs79658468	14	37,039,439	Intergenic	0.29	3.50×10^{-8}
rs3809435	14	37,052,632	Intergenic	0.29	3.50×10^{-8}
rs3809438	14	37,053,331	Intergenic	0.29	3.50×10^{-8}
rs3809439	14	37,053,602	Intergenic	0.29	3.50×10^{-8}
rs17104784	14	37,059,309	Intergenic	0.29	3.50×10^{-8}
rs75413287	14	37,072,002	Intergenic	0.29	3.35×10^{-8}
rs149692925	14	37,072,933	Intergenic	0.29	3.35×10^{-8}
rs79009436	14	37,073,674	Intergenic	0.29	3.35×10^{-8}
rs74610566	14	37,075,302	Intergenic	0.30	2.21×10^{-8}
rs75729501	14	37,078,409	Intergenic	0.30	2.21×10^{-8}
rs117276383	14	37,079,764	Intergenic	0.30	2.21×10^{-8}
rs117704811	14	37,084,482	Intergenic	0.30	2.08×10^{-8}
rs118083282	14	37,084,791	Intergenic	0.30	2.21×10^{-8}
rs79056984	14	37,085,147	Intergenic	0.30	2.21×10^{-8}
rs78936886	14	37,085,442	Intergenic	0.30	2.21×10^{-8}
rs75327183	15	56,814,218	Intergenic	0.33	2.98×10^{-8}
rs58074387	15	74,191,225	Intergenic	0.31	4.46×10^{-8}

	rs3987678	16	28,652,709	Intergenic	0.31	1.53×10^{-8}
	rs72798056	16	50,184,574	Intergenic	0.30	3.37×10^{-8}
	rs17281013	16	56,320,435	*GNAO1*	0.29	2.64×10^{-8}
	rs8043572	16	56,323,232	*GNAO1*	0.29	2.64×10^{-8}
	rs192073405	17	69,639,501	Intergenic	0.41	4.59×10^{-13}
	rs143620169	17	73,224,653	*NUP85*	0.35	1.24×10^{-9}
	rs79858696	17	73,233,599	*GGA3*	0.35	9.28×10^{-10}
	rs149164	20	12,894,601	*LOC101929486*	0.32	3.46×10^{-8}
	rs636066	20	12,903,104	*LOC101929486*	0.32	3.46×10^{-8}
	rs181717	20	12,917,095	*LOC101929486; LOC102606466*	0.32	3.37×10^{-8}
	rs56302497	20	53,086,256	Intergenic	0.30	2.36×10^{-8}
	rs68091706	20	53,087,669	Intergenic	0.29	3.40×10^{-8}
	rs6023266	20	53,088,542	Intergenic	0.29	3.40×10^{-8}
	rs186046502	20	60,898,308	*LAMA5*	0.31	2.14×10^{-8}
	rs112629906	20	61,199,642	Intergenic	0.37	1.49×10^{-10}
	rs112886784	20	61,206,397	Intergenic	0.33	2.94×10^{-9}
	rs117388418	20	61,279,131	*SLCO4A1*	0.34	5.54×10^{-11}
	rs34419428	20	61,288,015	*SLCO4A1*	0.39	5.17×10^{-12}
	rs75000682	21	42,662,479	Intergenic	0.35	2.85×10^{-9}
	rs75896199	21	42,678,194	Intergenic	0.34	4.38×10^{-9}
	rs57347504	21	42,678,481	Intergenic	0.34	4.38×10^{-9}
	rs112075738	21	42,680,958	Intergenic	0.34	4.38×10^{-9}
	rs11910683	21	42,690,922	*FAM3B*	0.34	4.38×10^{-9}
	rs2838011	21	42,694,667	*FAM3B*	0.33	8.39×10^{-9}
	rs2838013	21	42,695,161	*FAM3B*	0.33	8.39×10^{-9}
	rs6517664	21	42,704,983	*FAM3B*	0.32	2.39×10^{-8}
	rs8129297	21	42,709,596	*FAM3B*	0.33	8.39×10^{-9}
	rs28478237	21	42,712,315	*FAM3B*	0.35	2.00×10^{-10}
	rs28476518	21	42,712,517	*FAM3B*	0.35	2.00×10^{-10}
	rs28451078	21	42,712,519	*FAM3B*	0.35	2.00×10^{-10}
	rs75055531	21	42,712,996	*FAM3B*	0.35	2.00×10^{-10}
	rs77974492	21	42,713,863	*FAM3B*	0.35	2.00×10^{-10}
	rs28478227	21	42,714,108	*FAM3B*	0.34	2.90×10^{-10}
	rs112716132	22	27,463,211	Intergenic	0.32	1.24×10^{-8}
	rs6006621	22	44,419,407	*PARVB*	0.33	1.52×10^{-8}
ITGA6	rs3127819	10	46,999,019	*GPRIN2*	-0.92	1.92×10^{-9}
	rs3127682	10	46,999,465	*GPRIN2*	-0.92	1.92×10^{-9}
	rs3127821	10	46,999,747	*GPRIN2*	-0.92	1.92×10^{-9}
	rs3127823	10	47,000,079	*GPRIN2*	-0.92	1.92×10^{-9}
	rs7912299	10	47,000,239	*GPRIN2*	-0.92	1.92×10^{-9}
ITGAV	rs35073780	1	117,411,009	Intergenic	1.21	1.55×10^{-8}
	rs6544949	2	23,787,723	*KLHL29*	1.61	7.90×10^{-9}
	rs72849620	2	23,788,815	*KLHL29*	1.61	7.90×10^{-9}
	rs72849625	2	23,791,785	*KLHL29*	1.61	7.90×10^{-9}
	rs72849631	2	23,793,448	*KLHL29*	1.61	7.90×10^{-9}
	rs76903023	2	85,615,750	*ELMOD3*	1.62	2.24×10^{-8}
	rs72646053	4	59,214,206	Intergenic	1.63	2.72×10^{-8}
	rs142312273	7	69,689,322	*AUTS2*	1.58	4.06×10^{-8}
	rs75454689	8	33,827,581	Intergenic	1.56	1.70×10^{-9}

	rs113896449	8	112,372,300	Intergenic	1.12	2.84×10^{-9}
	rs73709198	8	119,754,591	Intergenic	1.61	1.45×10^{-8}
	rs77178878	9	138,197,316	Intergenic	1.29	4.12×10^{-8}
	rs12360054	10	10,510,697	Intergenic	1.61	4.65×10^{-9}
	rs11013721	10	24,099,710	*KIAA1217*	1.58	9.61×10^{-9}
	rs61878765	11	44,624,149	*CD82*	1.56	3.77×10^{-8}
	rs7980462	12	56,535,326	*ESYT1*	1.38	2.72×10^{-8}
	rs9541625	13	69,490,725	Intergenic	1.71	8.72×10^{-9}
	rs17621546	17	35,407,828	*AATF*	1.41	7.61×10^{-9}
	rs2187048	18	35,033,025	*CELF4*	1.23	1.79×10^{-8}
	rs7236596	18	35,033,611	*CELF4*	1.23	1.79×10^{-8}
ITGB5	rs74807395	1	72,243,867	*NEGR1*	1.65	4.35×10^{-8}
	rs55705629	1	81,822,629	Intergenic	1.15	4.26×10^{-8}
	rs12756555	1	81,827,251	Intergenic	1.42	3.72×10^{-10}
	rs61768907	1	81,841,624	Intergenic	1.13	4.68×10^{-8}
	rs55938551	2	242,033,593	*MTERFD2; SNED1*	1.63	3.21×10^{-8}
	rs59495246	3	112,050,638	Intergenic	1.66	3.39×10^{-9}
	rs61651681	3	112,050,646	Intergenic	1.66	3.39×10^{-9}
	rs73856005	3	112,052,833	*CD200*	1.70	5.06×10^{-9}
	rs16859476	3	112,060,256	*CD200*	1.65	1.00×10^{-8}
	rs12106675	3	112,061,773	*CD200*	1.65	1.00×10^{-8}
	rs4575866	3	112,062,307	*CD200*	1.65	1.00×10^{-8}
	rs4582023	3	112,062,449	*CD200*	1.65	1.00×10^{-8}
	rs16859484	3	112,063,485	*CD200*	1.65	1.00×10^{-8}
	rs3817425	3	112,063,705	*CD200*	1.65	1.00×10^{-8}
	rs11921546	3	112,064,263	*CD200*	1.65	1.00×10^{-8}
	rs60497880	3	112,064,266	*CD200*	1.65	1.00×10^{-8}
	rs60673403	3	112,064,830	*CD200*	1.65	1.00×10^{-8}
	rs59863561	3	112,064,879	*CD200*	1.62	1.59×10^{-8}
	rs1050572	3	112,066,562	*CD200*	1.65	1.00×10^{-8}
	rs2399417	3	112,067,241	*CD200*	1.62	1.17×10^{-8}
	rs2399418	3	112,067,438	*CD200*	1.65	1.00×10^{-8}
	rs59981538	3	112,068,064	*CD200*	1.65	1.00×10^{-8}
	rs60377655	3	112,068,440	*CD200*	1.65	1.00×10^{-8}
	rs57404826	3	112,068,517	*CD200*	1.65	1.00×10^{-8}
	rs113182723	3	179,242,704	Intergenic	1.94	1.98×10^{-9}
	rs150745577	3	179,242,808	Intergenic	1.94	1.98×10^{-9}
	rs4687412	3	193,079,117	*ATP13A5*	1.84	6.58×10^{-9}
	rs1521275	3	193,079,424	*ATP13A5*	1.84	6.58×10^{-9}
	rs4234629	3	193,081,516	*ATP13A5*	1.84	6.58×10^{-9}
	rs4234630	3	193,081,748	*ATP13A5*	1.84	6.58×10^{-9}
	rs923496	3	193,081,830	*ATP13A5*	1.84	6.58×10^{-9}
	rs2037427	3	193,082,557	*ATP13A5*	1.84	6.58×10^{-9}
	rs1489929	3	193,083,021	*ATP13A5*	1.84	6.58×10^{-9}
	rs1489928	3	193,083,140	*ATP13A5*	1.84	6.58×10^{-9}
	rs1813604	3	193,083,891	*ATP13A5*	1.84	6.58×10^{-9}
	rs1352631	3	193,084,066	*ATP13A5*	1.84	6.58×10^{-9}
	rs1352629	3	193,084,295	*ATP13A5*	1.84	6.58×10^{-9}
	rs6778209	3	193,084,914	*ATP13A5*	1.84	6.58×10^{-9}

rs6778217	3	193,084,920	*ATP13A5*	1.84	6.58×10^{-9}
rs7638331	3	193,086,512	*ATP13A5*	1.81	9.04×10^{-9}
rs2220523	3	193,087,044	*ATP13A5*	1.77	1.01×10^{-8}
rs9873417	3	193,087,357	*ATP13A5*	1.84	6.58×10^{-9}
rs1387706	3	193,087,860	*ATP13A5*	1.84	6.58×10^{-9}
rs1851217	3	193,088,011	*ATP13A5*	1.84	6.58×10^{-9}
rs1387708	3	193,088,442	*ATP13A5*	1.84	6.58×10^{-9}
rs1387707	3	193,088,509	*ATP13A5*	1.84	6.58×10^{-9}
rs4687413	3	193,088,870	*ATP13A5*	1.84	6.58×10^{-9}
rs1489939	3	193,089,281	*ATP13A5*	1.84	6.58×10^{-9}
rs1489937	3	193,089,495	*ATP13A5*	1.84	6.58×10^{-9}
rs4687414	3	193,089,580	*ATP13A5*	1.84	6.58×10^{-9}
rs2171848	3	193,090,255	*ATP13A5*	1.84	6.58×10^{-9}
rs6444712	3	193,090,260	*ATP13A5*	1.84	6.58×10^{-9}
rs1565354	3	193,090,398	*ATP13A5*	1.84	6.58×10^{-9}
rs1565353	3	193,090,633	*ATP13A5*	1.84	6.58×10^{-9}
rs7621312	3	193,090,834	*ATP13A5*	1.84	6.58×10^{-9}
rs7621397	3	193,090,858	*ATP13A5*	1.84	6.58×10^{-9}
rs4687416	3	193,091,558	*ATP13A5*	1.84	6.58×10^{-9}
rs9844823	3	193,092,603	*ATP13A5*	1.84	6.58×10^{-9}
rs1602189	3	193,092,922	*ATP13A5*	1.84	6.58×10^{-9}
rs1915571	3	193,093,271	*ATP13A5*	1.84	6.58×10^{-9}
rs4687417	3	193,094,381	*ATP13A5*	1.70	3.16×10^{-8}
rs4686634	3	193,094,411	*ATP13A5*	1.84	6.58×10^{-9}
rs1489935	3	193,096,077	*ATP13A5*	1.79	1.18×10^{-8}
rs4687420	3	193,098,050	Intergenic	1.79	1.18×10^{-8}
rs4687421	3	193,098,217	Intergenic	1.79	1.26×10^{-8}
rs6444713	3	193,098,911	Intergenic	1.74	2.22×10^{-8}
rs6444714	3	193,100,563	Intergenic	1.79	1.26×10^{-8}
rs16879739	6	17,456,735	*CAP2*	1.93	1.78×10^{-8}
rs139141718	6	20,874,637	*CDKAL1*	2.01	8.11×10^{-9}
rs78104904	9	111,991,227	*EPB41L4B*	1.82	3.98×10^{-8}
rs61606572	11	3,855,142	*RHOG*	1.59	3.45×10^{-8}
rs148149865	11	58,488,436	*GLYAT*	2.11	1.25×10^{-9}
rs1333541	13	71,291,081	Intergenic	1.61	4.82×10^{-9}
rs2792213	14	65,771,339	Intergenic	2.03	2.19×10^{-8}
rs117147549	14	65,912,463	*FUT8*	1.89	1.36×10^{-8}
rs10146602	14	89,693,105	*FOXN3*	1.90	3.52×10^{-8}
rs11624027	14	89,694,080	*FOXN3*	1.90	3.52×10^{-8}
rs185036609	14	106,471,245	Intergenic	2.27	1.08×10^{-14}
rs190814253	14	106,471,253	Intergenic	2.18	5.41×10^{-14}
rs141136074	14	106,471,280	Intergenic	2.18	5.41×10^{-14}
rs116043222	14	106,600,403	Intergenic	1.89	9.22×10^{-9}
rs115327084	14	106,600,426	Intergenic	2.14	5.50×10^{-10}
rs35607576	14	106,871,303	Intergenic	1.75	1.59×10^{-8}
rs74901541	15	71,514,019	*THSD4*	1.79	4.85×10^{-9}
rs28700084	15	71,514,666	*THSD4*	1.79	4.85×10^{-9}
rs28674550	15	71,526,871	*THSD4*	1.85	4.56×10^{-9}
rs28578042	15	71,527,010	*THSD4*	1.85	4.56×10^{-9}

	rs16955314	15	71,527,133	*THSD4*	1.85	4.56×10^{-9}
	rs181977918	18	75,133,321	Intergenic	1.79	1.76×10^{-8}
	rs74891480	19	20,660,656	Intergenic	1.86	1.16×10^{-9}
	rs117782107	20	13,117,956	*SPTLC3*	1.88	2.73×10^{-9}
KITLG	rs74436626	1	20,459,647	Intergenic	0.15	1.14×10^{-8}
	rs11581920	1	113,508,066	Intergenic	0.16	2.05×10^{-8}
	rs3753428	1	147,010,018	Intergenic	0.17	1.05×10^{-9}
	rs28633140	1	147,015,933	*BCL9*	0.16	5.91×10^{-10}
	rs11240077	1	147,016,448	*BCL9*	0.17	2.50×10^{-10}
	rs10494252	1	147,016,936	*BCL9*	0.17	2.50×10^{-10}
	rs138996047	1	147,018,018	*BCL9*	0.16	7.22×10^{-10}
	rs1027423	1	147,018,529	*BCL9*	0.17	1.33×10^{-10}
	rs12076319	1	147,018,782	*BCL9*	0.17	1.33×10^{-10}
	rs17160408	1	147,020,638	*BCL9*	0.17	1.33×10^{-10}
	rs12081028	1	147,022,697	*BCL9*	0.17	1.33×10^{-10}
	rs10494251	1	147,023,894	*BCL9*	0.17	1.33×10^{-10}
	rs10494250	1	147,024,047	*BCL9*	0.17	1.33×10^{-10}
	rs76170364	1	147,039,994	*BCL9*	0.17	1.33×10^{-10}
	rs17160461	1	147,049,876	*BCL9*	0.17	2.09×10^{-10}
	rs78653484	1	147,183,927	Intergenic	0.15	8.16×10^{-9}
	rs16848623	1	201,426,353	Intergenic	0.14	7.51×10^{-9}
	rs144854914	1	211,072,012	*KCNH1*	0.15	1.45×10^{-8}
	rs73125648	1	244,224,801	Intergenic	0.17	1.87×10^{-9}
	rs112097301	2	31,923,217	Intergenic	0.13	2.55×10^{-8}
	rs7573162	2	31,958,666	Intergenic	0.12	1.55×10^{-8}
	rs75782206	2	36,157,474	Intergenic	0.16	6.85×10^{-9}
	rs77147626	2	36,158,212	Intergenic	0.16	6.85×10^{-9}
	rs79879987	2	36,160,879	Intergenic	0.16	6.85×10^{-9}
	rs76460982	2	36,164,286	Intergenic	0.15	1.15×10^{-8}
	rs79777103	2	36,164,694	Intergenic	0.16	6.85×10^{-9}
	rs75971787	2	36,164,696	Intergenic	0.16	6.85×10^{-9}
	rs114687343	2	36,165,218	Intergenic	0.16	6.85×10^{-9}
	rs17017873	2	36,166,844	Intergenic	0.16	6.85×10^{-9}
	rs74319278	2	36,167,584	Intergenic	0.16	6.85×10^{-9}
	rs79170974	2	36,169,513	Intergenic	0.16	6.85×10^{-9}
	rs76432971	2	36,170,064	Intergenic	0.16	6.85×10^{-9}
	rs17017878	2	36,171,216	Intergenic	0.16	6.85×10^{-9}
	rs80230366	2	36,172,457	Intergenic	0.16	6.85×10^{-9}
	rs17017881	2	36,173,043	Intergenic	0.16	6.85×10^{-9}
	rs17017884	2	36,173,089	Intergenic	0.16	6.85×10^{-9}
	rs17017886	2	36,173,264	Intergenic	0.16	6.85×10^{-9}
	rs17017891	2	36,174,296	Intergenic	0.16	6.85×10^{-9}
	rs17017892	2	36,174,910	Intergenic	0.16	6.85×10^{-9}
	rs78913475	2	36,175,055	Intergenic	0.16	6.85×10^{-9}
	rs75521975	2	36,175,135	Intergenic	0.16	6.85×10^{-9}
	rs114580394	2	36,175,146	Intergenic	0.16	6.85×10^{-9}
	rs17017897	2	36,175,841	Intergenic	0.16	6.85×10^{-9}
	rs75475237	2	36,176,113	Intergenic	0.16	6.85×10^{-9}
	rs17017901	2	36,176,629	Intergenic	0.16	6.85×10^{-9}

rs17017915	2	36,176,892	Intergenic	0.16	6.85×10^{-9}
rs77156921	2	36,176,910	Intergenic	0.16	6.85×10^{-9}
rs78912121	2	36,177,115	Intergenic	0.16	6.85×10^{-9}
rs75749575	2	36,177,214	Intergenic	0.16	6.85×10^{-9}
rs77250066	2	36,177,653	Intergenic	0.16	6.85×10^{-9}
rs17838357	2	36,177,785	Intergenic	0.16	6.85×10^{-9}
rs115733761	2	36,177,977	Intergenic	0.16	6.85×10^{-9}
rs143729842	2	36,178,146	Intergenic	0.16	6.85×10^{-9}
rs76855363	2	36,178,567	Intergenic	0.16	6.85×10^{-9}
rs77734410	4	81,038,162	Intergenic	0.17	2.92×10^{-9}
rs7683141	4	111,100,198	*ELOVL6*	0.15	2.28×10^{-8}
rs72672363	4	115,228,560	Intergenic	0.13	4.39×10^{-8}
rs4835576	4	145,043,098	*GYPA*	0.16	2.25×10^{-8}
rs7696242	4	145,044,186	*GYPA*	0.16	2.25×10^{-8}
rs79882885	4	187,260,837	*F11-AS1*	0.15	3.31×10^{-8}
rs75171718	5	79,990,131	*MSH3*	0.15	1.19×10^{-9}
rs5003758	5	83,182,193	Intergenic	0.16	2.35×10^{-9}
rs72791183	5	111,963,677	Intergenic	0.14	4.21×10^{-8}
rs13160323	5	138,341,236	*SIL1*	0.15	1.26×10^{-8}
rs149810160	5	147,918,965	*HTR4*	0.14	3.93×10^{-8}
rs34182166	5	150,582,494	*CCDC69*	0.15	1.99×10^{-8}
rs3756644	5	153,780,979	*GALNT10; SAP30L-AS1*	0.14	4.07×10^{-8}
rs74530243	5	157,594,148	Intergenic	0.15	1.72×10^{-9}
rs927465	5	157,596,056	Intergenic	0.15	1.72×10^{-9}
rs17242549	5	157,597,476	Intergenic	0.15	1.72×10^{-9}
rs11742423	5	157,598,895	Intergenic	0.15	1.72×10^{-9}
rs11741492	5	157,606,532	Intergenic	0.15	1.72×10^{-9}
rs56014022	5	157,610,571	Intergenic	0.15	1.72×10^{-9}
rs147220874	5	157,611,807	Intergenic	0.15	4.71×10^{-9}
rs11743966	5	157,614,481	Intergenic	0.15	1.72×10^{-9}
rs11748189	5	157,614,896	Intergenic	0.15	1.72×10^{-9}
rs72802571	5	157,615,035	Intergenic	0.15	1.72×10^{-9}
rs4588578	5	157,615,637	Intergenic	0.15	1.72×10^{-9}
rs1874161	5	157,615,710	Intergenic	0.15	1.72×10^{-9}
rs1874160	5	157,616,148	Intergenic	0.15	1.72×10^{-9}
rs12110218	5	157,617,896	Intergenic	0.15	1.72×10^{-9}
rs12110224	5	157,618,221	Intergenic	0.15	1.72×10^{-9}
rs10055637	5	157,618,288	Intergenic	0.15	1.72×10^{-9}
rs11747817	5	157,618,680	Intergenic	0.15	1.72×10^{-9}
rs115960274	5	164,005,709	Intergenic	0.16	2.89×10^{-8}
rs72817570	5	164,584,901	Intergenic	0.14	3.26×10^{-8}
rs72817572	5	164,585,655	Intergenic	0.14	3.26×10^{-8}
rs34223351	5	164,586,498	Intergenic	0.14	3.26×10^{-8}
rs34233411	5	164,586,654	Intergenic	0.14	3.26×10^{-8}
rs8180513	5	168,749,112	Intergenic	0.15	1.64×10^{-8}
rs2057064	6	41,632,020	Intergenic	0.15	1.18×10^{-10}
rs13216349	6	75,151,471	*LOC101928516*	0.13	3.96×10^{-8}
rs2533450	7	69,086,665	*AUTS2*	0.14	1.05×10^{-8}
rs653929	7	69,187,050	*AUTS2*	0.14	6.42×10^{-9}

rs802046	7	86,951,809	Intergenic	0.15	1.25×10^{-8}
rs112714763	7	86,969,315	*TP53TG1*	0.15	2.63×10^{-8}
rs112796991	7	86,983,328	*CROT*	0.15	2.63×10^{-8}
rs802024	7	86,985,719	*CROT*	0.15	2.63×10^{-8}
rs802025	7	86,986,632	*CROT*	0.15	2.63×10^{-8}
rs117766049	7	129,684,199	*ZC3HC1*	0.15	1.23×10^{-8}
rs117814185	7	130,532,260	Intergenic	0.16	3.28×10^{-9}
rs113314246	7	135,418,147	*FAM180A*	0.14	5.28×10^{-9}
rs75170699	7	135,425,191	*FAM180A*	0.14	5.28×10^{-9}
rs80096630	7	135,432,784	*FAM180A*	0.14	3.28×10^{-9}
rs112692348	7	135,434,483	Intergenic	0.14	5.28×10^{-9}
rs73169197	7	155,701,424	Intergenic	0.15	1.56×10^{-8}
rs78876270	8	8,724,296	*MFHAS1*	0.13	4.01×10^{-8}
rs6474703	9	12,336,801	Intergenic	0.16	9.42×10^{-10}
rs6474704	9	12,336,809	Intergenic	0.15	2.32×10^{-9}
rs76483310	9	18,804,514	*ADAMTSL1*	0.15	2.05×10^{-8}
rs9410528	9	82,125,143	Intergenic	0.15	1.88×10^{-8}
rs2779541	9	101,389,839	*GABBR2*	0.13	4.93×10^{-8}
rs2778917	9	101,390,273	*GABBR2*	0.13	4.93×10^{-8}
rs1930138	9	101,429,694	*GABBR2*	0.13	4.96×10^{-8}
rs516930	9	101,444,133	*GABBR2*	0.13	1.97×10^{-8}
rs12775723	10	12,492,177	*CAMK1D*	0.15	2.00×10^{-8}
rs73595808	10	19,716,100	*MALRD1*	0.16	1.01×10^{-8}
rs3004203	10	27,689,092	*PTCHD3*	0.15	1.72×10^{-8}
rs7125369	11	42,771,556	Intergenic	0.15	2.23×10^{-8}
rs60965002	11	42,772,716	Intergenic	0.15	2.23×10^{-8}
rs60522351	11	42,772,925	Intergenic	0.15	2.23×10^{-8}
rs148493095	11	42,774,590	Intergenic	0.15	2.23×10^{-8}
rs146594721	11	42,775,170	Intergenic	0.15	2.23×10^{-8}
rs59994772	11	42,778,594	Intergenic	0.15	2.23×10^{-8}
rs113768087	11	81,026,144	Intergenic	0.16	3.28×10^{-9}
rs76951239	11	81,032,805	Intergenic	0.17	1.57×10^{-9}
rs7103369	11	81,122,821	Intergenic	0.15	4.71×10^{-9}
rs36124182	12	24,214,934	*SOX5*	0.15	1.11×10^{-8}
rs12319043	12	24,233,568	*SOX5*	0.14	2.99×10^{-8}
rs75342935	12	24,246,016	*SOX5*	0.15	1.36×10^{-8}
rs17408255	12	24,255,994	*SOX5*	0.15	1.36×10^{-8}
rs11047261	12	24,257,228	*SOX5*	0.15	1.36×10^{-8}
rs12313817	12	24,260,884	*SOX5*	0.15	1.36×10^{-8}
rs75673453	12	118,989,792	Intergenic	0.15	3.39×10^{-8}
rs184647600	13	49,635,517	*FNDC3A*	0.14	1.97×10^{-8}
rs55812543	13	113,745,989	*MCF2L*	0.15	1.92×10^{-8}
rs75027256	14	51,976,087	*FRMD6; FRMD6-AS2*	0.14	7.24×10^{-9}
rs28702736	14	62,435,456	Intergenic	0.15	7.23×10^{-9}
rs2372425	14	83,962,756	Intergenic	0.15	2.30×10^{-8}
rs17694128	14	83,977,073	Intergenic	0.15	2.30×10^{-8}
rs17621157	14	83,978,650	Intergenic	0.15	2.30×10^{-8}
rs76414300	14	83,980,412	Intergenic	0.15	2.30×10^{-8}
rs185357657	15	30,893,443	Intergenic	0.15	3.96×10^{-8}

rs166723	15	39,520,759	Intergenic	0.15	1.91×10^{-8}
rs78540980	15	65,418,010	*PDCD7*	0.13	4.05×10^{-8}
rs11637917	15	65,856,459	*PTPLAD1*	0.15	9.23×10^{-9}
rs112556408	15	66,050,927	*DENND4A*	0.16	1.72×10^{-9}
rs74890395	15	66,052,091	*DENND4A*	0.17	6.35×10^{-10}
rs112538248	15	66,124,595	Intergenic	0.15	1.88×10^{-8}
rs78865086	15	66,126,145	Intergenic	0.15	1.88×10^{-8}
rs73475467	15	66,128,255	Intergenic	0.15	1.88×10^{-8}
rs11635670	15	66,286,114	*MEGF11*	0.15	2.46×10^{-8}
rs79955167	15	66,287,444	*MEGF11*	0.15	2.46×10^{-8}
rs77751535	15	66,288,134	*MEGF11*	0.15	2.46×10^{-8}
rs74334280	15	66,288,184	*MEGF11*	0.15	2.46×10^{-8}
rs28564180	15	66,288,726	*MEGF11*	0.15	4.04×10^{-8}
rs76620910	15	66,295,960	*MEGF11*	0.15	2.46×10^{-8}
rs76394551	15	66,304,999	*MEGF11*	0.15	2.42×10^{-8}
rs2160305	16	54,006,217	*FTO*	0.15	4.96×10^{-8}
rs75990376	16	79,792,970	*LOC102467146*	0.16	2.84×10^{-10}
rs9925929	16	79,798,511	*LOC102467146*	0.16	2.84×10^{-10}
rs78303331	17	5,771,490	*LOC339166*	0.15	4.59×10^{-8}
rs36122742	17	60,567,313	*TLK2*	0.15	3.55×10^{-8}
rs117969406	17	77,688,797	Intergenic	0.15	1.12×10^{-8}
rs4889986	17	77,692,127	Intergenic	0.15	1.12×10^{-8}
rs72490478	17	77,692,979	Intergenic	0.15	2.56×10^{-8}
rs62076895	18	3,199,538	*MYOM1*	0.14	4.95×10^{-8}
rs9964300	18	3,215,030	*MYOM1*	0.17	2.88×10^{-9}
rs1662313	18	3,217,953	*MYOM1*	0.15	1.10×10^{-8}
rs1791068	18	3,219,215	*MYOM1*	0.15	1.10×10^{-8}
rs1791062	18	3,219,744	*MYOM1*	0.15	1.10×10^{-8}
rs1791133	18	3,220,050	*MYOM1*	0.15	1.10×10^{-8}
rs1791131	18	3,220,354	Intergenic	0.15	1.10×10^{-8}
rs1662312	18	3,220,450	Intergenic	0.15	1.10×10^{-8}
rs9963109	18	3,220,497	Intergenic	0.15	1.10×10^{-8}
rs9955283	18	3,220,731	Intergenic	0.15	1.10×10^{-8}
rs9963511	18	3,220,885	Intergenic	0.15	1.41×10^{-8}
rs1808916	18	3,221,035	Intergenic	0.15	1.10×10^{-8}
rs1808915	18	3,221,166	Intergenic	0.15	1.41×10^{-8}
rs894565	18	3,221,660	Intergenic	0.15	1.10×10^{-8}
rs894564	18	3,221,739	Intergenic	0.15	1.41×10^{-8}
rs9959375	18	3,222,795	Intergenic	0.15	1.10×10^{-8}
rs9950110	18	3,223,005	Intergenic	0.15	1.10×10^{-8}
rs9949484	18	3,223,132	Intergenic	0.15	1.10×10^{-8}
rs9962345	18	3,223,413	Intergenic	0.15	1.10×10^{-8}
rs1662310	18	3,223,762	Intergenic	0.15	1.10×10^{-8}
rs7238780	18	3,224,807	Intergenic	0.14	1.86×10^{-8}
rs752396	18	3,226,089	Intergenic	0.15	1.10×10^{-8}
rs11664526	18	3,227,554	Intergenic	0.15	5.18×10^{-9}
rs1662307	18	3,228,096	Intergenic	0.16	3.97×10^{-9}
rs9957993	18	3,228,493	Intergenic	0.16	3.97×10^{-9}
rs1662305	18	3,229,023	Intergenic	0.16	3.97×10^{-9}

	rs28552959	18	3,229,690	Intergenic	0.15	5.75×10^{-9}
	rs2920346	18	3,229,743	Intergenic	0.16	3.97×10^{-9}
	rs62076898	18	3,231,615	Intergenic	0.16	3.97×10^{-9}
	rs35500334	18	3,233,236	Intergenic	0.16	1.58×10^{-9}
	rs1791069	18	3,233,609	Intergenic	0.16	1.58×10^{-9}
	rs34730670	18	3,237,947	Intergenic	0.16	2.61×10^{-9}
	rs4447514	18	3,237,973	Intergenic	0.16	2.61×10^{-9}
	rs16976822	18	11,886,867	*MPPE1*	0.14	3.96×10^{-8}
	rs16976823	18	11,887,031	*MPPE1*	0.14	3.96×10^{-8}
	rs9951817	18	11,887,388	*MPPE1*	0.14	3.96×10^{-8}
	rs116582165	18	66,625,544	*CCDC102B*	0.16	1.44×10^{-8}
	rs12975335	19	22,846,680	*ZNF492*	0.16	1.17×10^{-9}
	rs62118831	19	22,846,697	*ZNF492*	0.17	8.06×10^{-11}
	rs73069649	19	56,777,592	Intergenic	0.15	4.75×10^{-8}
	rs9789836	20	1,178,409	Intergenic	0.16	1.18×10^{-8}
	rs74397637	20	54,837,046	Intergenic	0.12	3.00×10^{-8}
	rs17457993	20	54,837,218	Intergenic	0.12	3.00×10^{-8}
	rs77843524	20	54,837,236	Intergenic	0.12	3.00×10^{-8}
	rs78102068	20	54,837,294	Intergenic	0.11	4.96×10^{-8}
	rs75341483	20	54,837,471	Intergenic	0.12	3.00×10^{-8}
	rs75108000	20	54,838,516	Intergenic	0.12	2.08×10^{-8}
	rs17003413	22	43,313,954	*PACSIN2*	0.15	4.42×10^{-8}
KIT	rs113426339	1	178,947,559	Intergenic	0.06	3.52×10^{-8}
	rs12520827	5	113,396,378	Intergenic	0.05	5.54×10^{-9}
	rs145306580	9	4,190,807	*GLIS3*	0.07	5.15×10^{-9}
KRAS	rs4078119	1	101,819,489	Intergenic	1.72	5.75×10^{-9}
	rs12568209	1	101,820,149	Intergenic	1.72	5.75×10^{-9}
	rs58904095	1	101,825,899	Intergenic	1.77	3.22×10^{-9}
	rs2087709	1	101,827,992	Intergenic	1.77	3.22×10^{-9}
	rs115540792	1	101,828,584	Intergenic	1.77	3.22×10^{-9}
	rs7538660	1	101,833,755	Intergenic	1.77	3.22×10^{-9}
	rs76297543	1	101,834,525	Intergenic	1.77	3.22×10^{-9}
	rs11164181	1	101,845,846	Intergenic	1.92	3.80×10^{-9}
	rs111381284	5	44,496,612	Intergenic	1.84	5.06×10^{-9}
	rs17320222	5	44,499,291	Intergenic	1.77	1.01×10^{-8}
	rs78297490	5	44,542,953	Intergenic	1.80	8.73×10^{-9}
	rs11953011	5	44,627,658	Intergenic	1.74	9.65×10^{-9}
	rs181701912	5	44,888,831	Intergenic	1.45	4.49×10^{-9}
	rs4988787	7	8,994,004	Intergenic	1.71	1.93×10^{-10}
	rs917940	7	8,994,995	Intergenic	1.78	8.18×10^{-11}
	rs6972076	7	8,995,240	Intergenic	1.78	8.18×10^{-11}
	rs6972078	7	8,995,243	Intergenic	1.78	8.18×10^{-11}
	rs2192597	7	8,995,694	Intergenic	1.76	7.61×10^{-11}
	rs2110558	7	8,995,761	Intergenic	1.77	5.83×10^{-11}
	rs6463861	7	8,996,028	Intergenic	1.78	8.18×10^{-11}
	rs10238110	7	8,996,316	Intergenic	2.12	3.44×10^{-12}
	rs4033270	7	8,998,665	Intergenic	1.78	8.18×10^{-11}
	rs4725149	7	8,999,140	Intergenic	1.72	1.58×10^{-10}
	rs2024377	7	9,001,674	Intergenic	1.78	8.18×10^{-11}

	rs6971402	7	9,002,103	Intergenic	1.78	8.18×10^{-11}
	rs6971697	7	9,002,250	Intergenic	1.78	8.18×10^{-11}
	rs6959898	7	9,010,322	Intergenic	1.91	5.83×10^{-10}
	rs9655535	7	9,011,671	Intergenic	1.91	5.83×10^{-10}
LAMA1	rs6677231	1	35,042,287	Intergenic	0.10	4.62×10^{-8}
	rs74973423	1	41,905,705	Intergenic	0.12	1.27×10^{-8}
	rs76294340	1	57,014,332	*PPAP2B*	0.10	2.83×10^{-8}
	rs75346781	1	57,015,017	*PPAP2B*	0.10	2.83×10^{-8}
	rs4853431	2	79,297,474	Intergenic	0.12	6.89×10^{-9}
	rs67844640	2	154,003,627	Intergenic	0.10	1.18×10^{-8}
	rs6756148	2	199,371,044	Intergenic	0.08	3.67×10^{-8}
	rs78832831	3	15,546,568	*COLQ*	0.12	4.21×10^{-9}
	rs13087509	3	22,514,136	Intergenic	0.09	3.39×10^{-8}
	rs6441714	3	102,985,514	Intergenic	0.07	5.03×10^{-9}
	rs16829413	3	118,904,916	*UPK1B*	0.11	3.95×10^{-10}
	rs67108505	3	118,908,168	*UPK1B*	0.11	3.95×10^{-10}
	rs56193405	3	118,908,334	*UPK1B*	0.11	1.67×10^{-9}
	rs13320556	3	118,913,677	*UPK1B*	0.13	2.18×10^{-11}
	rs13324867	3	118,914,724	*UPK1B*	0.10	4.42×10^{-10}
	rs13315549	3	118,917,422	*UPK1B*	0.12	2.57×10^{-10}
	rs9844248	3	118,922,183	*UPK1B*	0.12	1.39×10^{-10}
	rs6766503	3	118,940,383	*B4GALT4*	0.12	2.57×10^{-10}
	rs28428903	3	118,941,387	*B4GALT4*	0.13	1.14×10^{-10}
	rs12487052	3	118,942,475	*B4GALT4*	0.12	2.57×10^{-10}
	rs4234656	3	118,948,155	*B4GALT4*	0.12	2.57×10^{-10}
	rs12492305	3	118,953,691	*B4GALT4*	0.12	2.57×10^{-10}
	rs6805930	3	118,955,611	*B4GALT4*	0.12	2.57×10^{-10}
	rs2594731	3	118,963,980	Intergenic	0.12	2.57×10^{-10}
	rs79728677	4	14,705,366	Intergenic	0.10	5.98×10^{-10}
	rs74991977	4	14,705,416	Intergenic	0.10	5.98×10^{-10}
	rs76707362	4	14,712,144	Intergenic	0.10	5.98×10^{-10}
	rs77620321	4	14,712,226	Intergenic	0.10	5.98×10^{-10}
	rs78279840	4	14,712,603	Intergenic	0.10	5.98×10^{-10}
	rs77645207	4	14,713,460	Intergenic	0.10	5.98×10^{-10}
	rs77618414	4	14,713,994	Intergenic	0.10	5.98×10^{-10}
	rs76479616	4	14,714,272	Intergenic	0.10	5.98×10^{-10}
	rs78172472	4	14,714,691	Intergenic	0.10	5.98×10^{-10}
	rs77695728	4	14,715,009	Intergenic	0.10	5.98×10^{-10}
	rs75488677	4	14,715,053	Intergenic	0.10	5.98×10^{-10}
	rs79204056	4	14,715,126	Intergenic	0.10	5.98×10^{-10}
	rs78559953	4	14,715,164	Intergenic	0.10	5.98×10^{-10}
	rs75981573	4	14,715,458	Intergenic	0.10	5.98×10^{-10}
	rs79970143	4	14,715,781	Intergenic	0.10	5.98×10^{-10}
	rs76717304	4	14,716,034	Intergenic	0.10	5.98×10^{-10}
	rs80065600	4	14,716,106	Intergenic	0.10	5.98×10^{-10}
	rs114770862	4	14,716,888	Intergenic	0.10	5.98×10^{-10}
	rs79625692	4	14,716,949	Intergenic	0.10	1.00×10^{-9}
	rs77503811	4	14,717,068	Intergenic	0.10	5.98×10^{-10}
	rs77289065	4	14,717,275	Intergenic	0.10	5.98×10^{-10}

rs74878175	4	14,717,391	Intergenic	0.10	5.98×10^{-10}
rs76034446	4	14,717,395	Intergenic	0.10	5.98×10^{-10}
rs77052203	4	14,717,643	Intergenic	0.10	5.98×10^{-10}
rs76478713	4	14,717,660	Intergenic	0.10	5.98×10^{-10}
rs80209817	4	14,717,676	Intergenic	0.10	5.98×10^{-10}
rs76602302	4	14,717,920	Intergenic	0.10	5.98×10^{-10}
rs75420187	4	14,718,318	Intergenic	0.10	5.98×10^{-10}
rs78067842	4	14,718,328	Intergenic	0.10	5.98×10^{-10}
rs143640407	4	14,726,454	Intergenic	0.12	1.94×10^{-11}
rs112694700	4	14,729,063	Intergenic	0.14	3.72×10^{-13}
rs79154919	4	14,732,089	Intergenic	0.14	4.17×10^{-13}
rs17623927	4	20,762,322	*KCNIP4*	0.11	3.06×10^{-9}
rs28651812	4	57,168,457	*KIAA1211*	0.09	3.49×10^{-8}
rs74403828	4	57,170,138	*KIAA1211*	0.09	1.77×10^{-8}
rs11736632	4	57,172,335	*KIAA1211*	0.11	6.28×10^{-10}
rs2697209	4	63,335,776	Intergenic	0.12	4.16×10^{-9}
rs77779319	4	182,508,854	Intergenic	0.13	8.53×10^{-11}
rs4958976	5	177,349,356	Intergenic	0.10	4.40×10^{-8}
rs74769936	6	47,322,096	Intergenic	0.11	3.59×10^{-9}
rs186415025	6	77,920,168	Intergenic	0.12	7.02×10^{-9}
rs72923610	6	91,438,094	Intergenic	0.09	3.31×10^{-8}
rs12664631	6	91,448,046	Intergenic	0.09	3.31×10^{-8}
rs143022854	6	91,448,290	Intergenic	0.09	3.31×10^{-8}
rs12206219	6	142,434,511	Intergenic	0.09	1.92×10^{-8}
rs77370456	6	142,485,489	*VTA1*	0.10	1.24×10^{-8}
rs12192519	6	142,521,654	*VTA1*	0.09	2.71×10^{-8}
rs78970535	6	142,855,375	*LOC153910*	0.11	9.41×10^{-9}
rs2627286	8	2,792,169	Intergenic	0.10	1.98×10^{-8}
rs7010713	8	2,796,574	*CSMD1*	0.11	2.19×10^{-8}
rs76931398	8	2,796,980	*CSMD1*	0.11	2.19×10^{-8}
rs75864120	8	2,806,636	*CSMD1*	0.11	9.10×10^{-9}
rs117094499	8	2,811,150	*CSMD1*	0.11	1.14×10^{-8}
rs151017158	8	2,831,641	*CSMD1*	0.12	4.28×10^{-9}
rs113798870	8	2,834,332	*CSMD1*	0.12	4.28×10^{-9}
rs79446325	8	2,840,584	*CSMD1*	0.12	4.28×10^{-9}
rs113799212	8	2,841,896	*CSMD1*	0.12	4.28×10^{-9}
rs112074569	8	2,841,956	*CSMD1*	0.12	4.28×10^{-9}
rs74370015	8	2,842,159	*CSMD1*	0.12	4.28×10^{-9}
rs76698791	8	2,843,303	*CSMD1*	0.12	4.28×10^{-9}
rs113222134	8	2,844,792	*CSMD1*	0.12	4.28×10^{-9}
rs74829597	8	2,845,476	*CSMD1*	0.11	5.48×10^{-9}
rs78173892	8	2,847,267	*CSMD1*	0.13	1.22×10^{-9}
rs75031764	8	2,847,383	*CSMD1*	0.13	1.22×10^{-9}
rs75583381	8	2,847,749	*CSMD1*	0.12	9.03×10^{-10}
rs675492	8	2,847,793	*CSMD1*	0.11	1.89×10^{-9}
rs113827317	8	2,848,915	*CSMD1*	0.12	2.39×10^{-9}
rs74726699	8	2,851,730	*CSMD1*	0.12	4.28×10^{-9}
rs111902488	8	2,852,710	*CSMD1*	0.12	4.28×10^{-9}
rs150958172	8	3,652,825	*CSMD1*	0.11	2.03×10^{-8}

rs73239493	8	27,121,839	Intergenic	0.12	2.02×10^{-9}
rs7843876	8	68,335,509	*CPA6*	0.12	9.92×10^{-9}
rs3886996	8	70,227,638	Intergenic	0.13	9.53×10^{-10}
rs34279900	8	128,650,492	Intergenic	0.14	1.64×10^{-12}
rs16907536	12	12,085,777	Intergenic	0.09	3.17×10^{-8}
rs11614805	12	20,745,536	*PDE3A*	0.12	7.43×10^{-9}
rs11608933	12	20,745,624	*PDE3A*	0.12	7.43×10^{-9}
rs60768792	12	20,751,178	*PDE3A*	0.12	7.43×10^{-9}
rs78215403	12	20,752,722	*PDE3A*	0.12	1.07×10^{-8}
rs75350945	12	20,754,415	*PDE3A*	0.12	1.07×10^{-8}
rs192706913	12	20,756,496	*PDE3A*	0.12	1.07×10^{-8}
rs80013638	12	20,756,767	*PDE3A*	0.12	1.07×10^{-8}
rs75758830	12	20,756,802	*PDE3A*	0.12	1.07×10^{-8}
rs117816662	12	20,757,173	*PDE3A*	0.12	6.72×10^{-9}
rs78190456	12	20,757,465	*PDE3A*	0.12	1.07×10^{-8}
rs11611208	12	20,758,613	*PDE3A*	0.12	1.07×10^{-8}
rs76021746	12	20,759,312	*PDE3A*	0.12	1.07×10^{-8}
rs117315355	12	20,759,533	*PDE3A*	0.11	1.93×10^{-8}
rs6487113	12	20,759,985	*PDE3A*	0.11	3.47×10^{-8}
rs7964099	12	20,764,100	*PDE3A*	0.12	1.07×10^{-8}
rs76357740	12	20,764,279	*PDE3A*	0.12	1.07×10^{-8}
rs7308381	12	20,766,685	*PDE3A*	0.11	2.36×10^{-8}
rs78245070	12	20,768,027	*PDE3A*	0.11	2.36×10^{-8}
rs76785459	12	20,769,617	*PDE3A*	0.11	2.36×10^{-8}
rs11610456	12	20,770,008	*PDE3A*	0.11	2.36×10^{-8}
rs9668712	12	20,776,764	*PDE3A*	0.11	2.36×10^{-8}
rs11611971	12	20,777,782	*PDE3A*	0.11	2.36×10^{-8}
rs76769379	12	20,784,241	*PDE3A*	0.12	6.65×10^{-9}
rs12307274	12	20,785,542	*PDE3A*	0.11	1.51×10^{-8}
rs76543675	12	20,789,278	*PDE3A*	0.12	1.10×10^{-8}
rs11615708	12	20,789,548	*PDE3A*	0.12	1.10×10^{-8}
rs80041598	12	20,794,447	*PDE3A*	0.11	2.42×10^{-8}
rs74528726	12	20,794,563	*PDE3A*	0.13	1.60×10^{-9}
rs61978204	14	70,642,537	*SLC8A3*	0.10	2.13×10^{-8}
rs9972608	15	27,905,337	Intergenic	0.09	6.68×10^{-9}
rs62001293	15	27,909,092	Intergenic	0.09	5.64×10^{-9}
rs11853117	15	27,910,801	Intergenic	0.09	7.18×10^{-9}
rs3097531	15	27,910,951	Intergenic	0.08	3.36×10^{-8}
rs12591822	15	27,911,483	Intergenic	0.09	7.18×10^{-9}
rs17746385	15	27,911,949	Intergenic	0.09	7.18×10^{-9}
rs3098578	15	27,912,716	Intergenic	0.08	3.36×10^{-8}
rs3098579	15	27,912,880	Intergenic	0.08	3.36×10^{-8}
rs3098582	15	27,913,398	Intergenic	0.08	3.36×10^{-8}
rs57784695	15	101,636,431	Intergenic	0.10	4.59×10^{-9}
rs117861357	17	25,308,787	Intergenic	0.11	2.30×10^{-8}
rs78695172	17	25,377,285	Intergenic	0.11	3.57×10^{-8}
rs8089682	18	28,574,472	*DSC3*	0.12	1.56×10^{-9}
rs12965995	18	28,575,626	*DSC3*	0.12	1.56×10^{-9}
rs1789051	18	28,576,533	*DSC3*	0.12	1.56×10^{-9}

	rs35877601	18	28,577,315	*DSC3*	0.12	1.56×10^{-9}
	rs17716604	18	47,654,952	*MYO5B*	0.12	1.56×10^{-9}
	rs12980482	19	19,792,816	Intergenic	0.13	1.49×10^{-9}
	rs112637369	22	24,026,240	*GUSBP11*	0.11	1.29×10^{-9}
	rs76947034	22	24,035,679	*GUSBP11; RGL4*	0.11	7.75×10^{-10}
	rs6004528	22	25,669,780	Intergenic	0.08	4.42×10^{-9}
	rs77216574	22	25,688,138	Intergenic	0.11	1.77×10^{-8}
	rs146186595	22	25,689,257	Intergenic	0.11	1.77×10^{-8}
LAMB1	rs72670917	1	39,077,160	Intergenic	1.39	1.40×10^{-8}
	rs72670919	1	39,077,554	Intergenic	1.39	1.40×10^{-8}
	rs75466349	1	39,077,626	Intergenic	1.39	1.40×10^{-8}
	rs72670920	1	39,077,644	Intergenic	1.39	1.40×10^{-8}
	rs56042036	1	39,077,914	Intergenic	1.39	1.40×10^{-8}
	rs67038721	1	39,087,924	Intergenic	1.33	3.91×10^{-8}
	rs72656105	1	39,108,736	Intergenic	1.33	3.91×10^{-8}
	rs11939141	4	96,380,589	*UNC5C*	1.45	4.16×10^{-8}
	rs17402898	7	8,309,069	Intergenic	1.31	3.01×10^{-8}
LAMB3	rs76510931	1	35,043,331	Intergenic	0.52	4.43×10^{-8}
	rs55751920	1	35,046,752	Intergenic	0.52	3.27×10^{-8}
	rs11983244	7	14,200,667	*DGKB*	0.49	1.77×10^{-8}
	rs139651235	8	109,993,018	Intergenic	0.51	4.98×10^{-8}
	rs67897587	12	13,293,153	Intergenic	0.52	1.30×10^{-8}
	rs17717574	16	6,012,336	Intergenic	0.42	1.28×10^{-9}
LAMC1	rs79375470	3	150,408,364	*ERICH6*	10.28	2.27×10^{-8}
	rs239509	6	80,691,070	Intergenic	8.91	3.79×10^{-8}
	rs183034640	17	48,378,305	Intergenic	8.12	4.54×10^{-8}
LAMC2	rs17386406	1	8,643,497	*RERE*	0.54	1.06×10^{-9}
	rs74060148	1	15,324,642	*KAZN*	0.50	4.69×10^{-8}
	rs11205415	1	249,120,684	Intergenic	0.49	2.40×10^{-8}
	rs11205416	1	249,120,794	Intergenic	0.53	5.28×10^{-9}
	rs7565217	2	47,248,466	*TTC7A*	0.42	3.35×10^{-8}
	rs7565682	2	47,248,833	*TTC7A*	0.42	2.67×10^{-8}
	rs7565683	2	47,248,834	*TTC7A*	0.42	2.67×10^{-8}
	rs78932664	3	22,064,740	Intergenic	0.53	4.74×10^{-8}
	rs142576515	3	121,765,754	Intergenic	0.50	1.11×10^{-8}
	rs55965158	3	175,006,562	*NAALADL2*	0.48	2.13×10^{-11}
	rs56250770	3	175,009,526	*NAALADL2*	0.48	2.13×10^{-11}
	rs10936837	3	175,014,005	*NAALADL2*	0.37	4.03×10^{-8}
	rs10936838	3	175,014,019	*NAALADL2*	0.37	4.03×10^{-8}
	rs76606542	3	178,110,638	Intergenic	0.56	3.28×10^{-8}
	rs9822877	3	178,111,729	Intergenic	0.56	3.28×10^{-8}
	rs13434209	3	178,112,406	Intergenic	0.56	3.28×10^{-8}
	rs77650620	3	178,117,420	Intergenic	0.56	3.28×10^{-8}
	rs76148250	3	178,119,737	Intergenic	0.56	3.28×10^{-8}
	rs79982808	3	178,119,788	Intergenic	0.56	3.28×10^{-8}
	rs67267562	4	67,128,141	Intergenic	0.47	2.75×10^{-9}
	rs34626813	4	67,128,705	Intergenic	0.47	2.75×10^{-9}
	rs67857601	4	67,142,691	Intergenic	0.46	3.32×10^{-9}
	rs34321123	4	67,142,703	Intergenic	0.46	3.32×10^{-9}

rs17218887	4	108,297,878	Intergenic	0.62	3.25×10^{-9}
rs75182356	4	128,539,231	Intergenic	0.42	1.80×10^{-8}
rs1370973	5	148,646,445	Intergenic	0.48	5.42×10^{-9}
rs10515624	5	148,646,653	Intergenic	0.49	2.51×10^{-9}
rs182201265	6	123,766,133	*LOC101927990; TRDN*	0.43	3.16×10^{-8}
rs113269145	7	68,890,254	Intergenic	0.48	3.07×10^{-8}
rs79708239	7	89,153,735	Intergenic	0.56	6.87×10^{-9}
rs1988969	7	89,169,683	Intergenic	0.58	4.19×10^{-9}
rs138171318	7	89,170,544	Intergenic	0.58	4.19×10^{-9}
rs35916261	7	89,180,706	Intergenic	0.58	4.19×10^{-9}
rs13238112	7	89,208,828	Intergenic	0.58	4.19×10^{-9}
rs73162118	7	145,894,179	*CNTNAP2*	0.58	8.12×10^{-9}
rs76410706	8	134,649,307	Intergenic	0.56	2.55×10^{-8}
rs17471115	9	2,270,173	Intergenic	0.45	3.19×10^{-8}
rs188411780	9	66,293,852	Intergenic	0.59	1.14×10^{-8}
rs114512480	9	66,313,626	Intergenic	0.57	1.25×10^{-8}
rs138787382	9	66,337,862	Intergenic	0.58	9.67×10^{-9}
rs141482512	9	66,338,242	Intergenic	0.59	5.84×10^{-9}
rs146714337	9	66,342,179	Intergenic	0.57	1.25×10^{-8}
rs184956483	9	66,343,556	Intergenic	0.56	9.76×10^{-9}
rs113589188	9	101,106,072	*GABBR2*	0.56	3.78×10^{-8}
rs72759648	9	101,106,440	*GABBR2*	0.56	3.78×10^{-8}
rs147918689	10	31,390,686	Intergenic	0.57	4.63×10^{-8}
rs10733783	10	64,833,459	Intergenic	0.59	1.36×10^{-8}
rs2136577	10	64,834,514	Intergenic	0.55	4.71×10^{-8}
rs2036790	10	64,834,786	Intergenic	0.57	2.29×10^{-8}
rs1396963	10	64,835,130	Intergenic	0.57	2.29×10^{-8}
rs10761699	10	64,840,056	Intergenic	0.57	2.29×10^{-8}
rs7893891	10	64,841,660	Intergenic	0.57	2.29×10^{-8}
rs10733784	10	64,844,186	Intergenic	0.57	2.29×10^{-8}
rs10761701	10	64,846,524	Intergenic	0.57	2.29×10^{-8}
rs113745971	10	64,847,058	Intergenic	0.56	1.18×10^{-8}
rs12263266	10	64,848,863	Intergenic	0.52	4.94×10^{-8}
rs72824931	10	64,849,482	Intergenic	0.52	4.94×10^{-8}
rs7896294	10	64,986,445	*JMJD1C*	0.57	3.05×10^{-8}
rs12259115	10	64,993,101	*JMJD1C*	0.57	3.05×10^{-8}
rs7907747	10	64,993,984	*JMJD1C*	0.57	3.05×10^{-8}
rs7907993	10	64,994,157	*JMJD1C*	0.57	3.05×10^{-8}
rs6479890	10	65,000,493	*JMJD1C*	0.57	3.05×10^{-8}
rs78745769	10	72,115,208	*LRRC20*	0.48	1.02×10^{-8}
rs10823526	10	72,115,475	*LRRC20*	0.50	4.57×10^{-9}
rs10823528	10	72,115,839	*LRRC20*	0.50	4.57×10^{-9}
rs115151048	10	72,121,454	*LRRC20*	0.50	4.57×10^{-9}
rs76702380	10	72,129,054	*LRRC20*	0.50	4.57×10^{-9}
rs2127584	10	72,132,013	*LRRC20*	0.50	4.57×10^{-9}
rs45450199	10	72,182,110	*EIF4EBP2*	0.51	1.21×10^{-9}
rs72824341	10	82,313,928	*SH2D4B*	0.54	2.33×10^{-8}
rs148744689	10	82,325,879	*SH2D4B*	0.53	2.58×10^{-8}
rs146230721	10	82,346,195	*SH2D4B*	0.54	2.07×10^{-8}

	rs192810909	10	82,346,690	*SH2D4B*	0.52	3.90×10^{-8}
	rs72805746	10	82,361,942	*SH2D4B*	0.51	3.68×10^{-8}
	rs72809868	10	83,292,537	Intergenic	0.66	3.23×10^{-10}
	rs56121726	10	83,295,594	Intergenic	0.66	3.23×10^{-10}
	rs59545274	10	83,314,472	Intergenic	0.60	8.44×10^{-9}
	rs7912649	10	83,315,909	Intergenic	0.61	9.35×10^{-9}
	rs72809901	10	83,319,884	Intergenic	0.63	3.48×10^{-9}
	rs66584082	10	83,324,483	Intergenic	0.59	8.72×10^{-9}
	rs72816049	10	83,351,851	Intergenic	0.64	2.81×10^{-9}
	rs7092774	10	83,352,442	Intergenic	0.64	2.81×10^{-9}
	rs72816051	10	83,352,644	Intergenic	0.64	2.81×10^{-9}
	rs72816056	10	83,354,081	Intergenic	0.64	2.81×10^{-9}
	rs72816059	10	83,355,333	Intergenic	0.64	2.81×10^{-9}
	rs72816060	10	83,355,414	Intergenic	0.64	2.81×10^{-9}
	rs72816064	10	83,356,665	Intergenic	0.64	2.81×10^{-9}
	rs72816065	10	83,357,520	Intergenic	0.64	2.81×10^{-9}
	rs78166894	10	83,358,335	Intergenic	0.64	2.81×10^{-9}
	rs141773517	10	83,358,825	Intergenic	0.64	2.81×10^{-9}
	rs74623904	10	83,359,635	Intergenic	0.64	2.81×10^{-9}
	rs72816069	10	83,359,891	Intergenic	0.64	2.81×10^{-9}
	rs72816070	10	83,360,089	Intergenic	0.64	2.81×10^{-9}
	rs72816072	10	83,360,186	Intergenic	0.64	2.81×10^{-9}
	rs139422498	10	83,360,572	Intergenic	0.64	2.81×10^{-9}
	rs72816073	10	83,362,338	Intergenic	0.64	2.81×10^{-9}
	rs72816074	10	83,362,400	Intergenic	0.64	2.81×10^{-9}
	rs72816076	10	83,362,421	Intergenic	0.64	2.81×10^{-9}
	rs72816078	10	83,362,599	Intergenic	0.64	2.81×10^{-9}
	rs72816080	10	83,363,337	Intergenic	0.64	2.81×10^{-9}
	rs72816082	10	83,363,718	Intergenic	0.64	2.81×10^{-9}
	rs72816093	10	83,366,246	Intergenic	0.62	6.14×10^{-9}
	rs117640372	10	83,369,504	Intergenic	0.58	1.98×10^{-8}
	rs56356362	10	83,371,521	Intergenic	0.64	2.90×10^{-9}
	rs72817825	10	83,374,827	Intergenic	0.55	4.22×10^{-8}
	rs151176250	10	83,376,295	Intergenic	0.62	4.71×10^{-9}
	rs183456282	10	83,395,561	Intergenic	0.64	1.94×10^{-9}
	rs4586108	11	26,765,167	Intergenic	0.46	1.14×10^{-8}
	rs10466389	11	26,771,716	Intergenic	0.45	1.32×10^{-8}
	rs74459769	11	95,987,098	*MAML2*	0.52	3.18×10^{-8}
	rs2076799	13	36,721,182	Intergenic	0.56	4.27×10^{-8}
	rs2322894	13	36,736,922	Intergenic	0.56	4.54×10^{-8}
	rs1410636	13	36,737,086	Intergenic	0.56	4.54×10^{-8}
	rs1410635	13	36,737,113	Intergenic	0.56	4.54×10^{-8}
	rs1898283	13	53,229,252	*SUGT1*	0.57	9.54×10^{-9}
	rs181119556	15	20,760,461	Intergenic	0.53	2.85×10^{-10}
	rs12149512	16	4,227,505	Intergenic	0.57	1.65×10^{-8}
	rs12324980	16	4,250,114	*SRL*	0.58	1.62×10^{-8}
	rs17136871	16	4,252,645	*SRL*	0.56	3.78×10^{-8}
LPAR5	rs191981859	11	116,809,251	*SIK3*	1.01	2.12×10^{-8}
MAPK1	rs72861432	11	13,187,603	Intergenic	5.99	3.42×10^{-8}

MET	rs73184769	3	175,061,183	*NAALADL2*	0.04	1.13×10^{-8}
	rs149410839	3	180,153,254	Intergenic	0.07	6.11×10^{-9}
	rs17339635	10	14,487,154	Intergenic	0.06	4.56×10^{-8}
	rs146615136	13	22,028,207	*ZDHHC20*	0.06	3.94×10^{-8}
	rs73159315	22	39,213,327	Intergenic	0.05	2.72×10^{-8}
MTOR	rs74327628	4	42,196,927	Intergenic	9.57	9.49×10^{-9}
	rs111501395	4	42,197,378	Intergenic	9.57	9.49×10^{-9}
MYB	rs148283323	1	24,329,617	Intergenic	2.97	1.03×10^{-9}
	rs147880708	1	73,704,258	Intergenic	2.88	1.44×10^{-8}
	rs61469378	1	85,331,918	*LPAR3*	3.06	7.55×10^{-10}
	rs787148	2	144,663,911	Intergenic	2.15	4.66×10^{-8}
	rs78148102	2	159,819,408	Intergenic	2.62	1.62×10^{-8}
	rs842073	2	159,824,573	Intergenic	2.60	1.56×10^{-8}
	rs1719079	2	159,841,372	*TANC1*	2.60	1.56×10^{-8}
	rs264599	2	159,882,085	*TANC1*	2.60	1.56×10^{-8}
	rs77910980	2	159,882,854	*TANC1*	2.60	1.56×10^{-8}
	rs77137053	2	159,921,122	*TANC1*	2.78	5.01×10^{-9}
	rs147441210	4	9,003,867	Intergenic	2.77	3.35×10^{-8}
	rs144540401	4	29,216,658	Intergenic	2.82	4.72×10^{-8}
	rs72910722	6	85,422,673	Intergenic	2.76	2.76×10^{-8}
	rs12215883	6	85,423,614	Intergenic	2.76	2.76×10^{-8}
	rs184854229	7	37,773	Intergenic	2.72	3.47×10^{-8}
	rs150989719	7	387,508	Intergenic	2.68	4.28×10^{-8}
	rs149979215	7	481,372	Intergenic	2.39	2.18×10^{-8}
	rs992616	8	17,996,141	Intergenic	2.64	2.75×10^{-8}
	rs1858723	8	17,996,624	Intergenic	2.64	2.75×10^{-8}
	rs75291628	8	20,729,845	Intergenic	2.77	1.59×10^{-8}
	rs117281412	8	20,732,974	Intergenic	2.77	1.59×10^{-8}
	rs79261861	8	20,734,514	Intergenic	2.77	1.59×10^{-8}
	rs17361080	8	35,999,348	Intergenic	2.74	4.17×10^{-8}
	rs182250531	8	52,338,369	*PXDNL*	2.82	2.70×10^{-8}
	rs17720343	8	70,509,789	*SULF1*	2.46	4.06×10^{-8}
	rs11015813	10	27,774,276	Intergenic	2.89	3.21×10^{-8}
	rs11015814	10	27,774,832	Intergenic	2.89	3.21×10^{-8}
	rs61285402	10	27,776,827	Intergenic	2.89	3.21×10^{-8}
	rs74969813	10	27,839,861	Intergenic	2.89	3.21×10^{-8}
	rs11015874	10	27,850,032	Intergenic	2.89	3.34×10^{-8}
	rs11015880	10	27,861,452	Intergenic	2.93	2.24×10^{-8}
	rs78740100	10	53,831,221	*PRKG1*	2.80	7.14×10^{-9}
	rs11227408	11	65,868,971	*PACS1*	2.80	3.26×10^{-8}
	rs7134150	12	20,591,332	*PDE3A*	2.67	2.03×10^{-8}
	rs73239573	12	20,592,268	*PDE3A*	3.00	1.50×10^{-9}
	rs11045247	12	20,592,624	*PDE3A*	2.57	1.66×10^{-8}
	rs12314390	12	20,597,977	*PDE3A*	3.03	1.38×10^{-9}
	rs117857467	17	41,836,456	Intergenic	2.76	3.75×10^{-9}
	rs62078607	17	41,848,271	*DUSP3*	2.70	5.80×10^{-9}
	rs192299491	17	43,560,815	*PLEKHM1*	2.79	3.00×10^{-9}
	rs71358642	18	62,485,590	Intergenic	2.69	1.59×10^{-8}
	rs71366981	18	62,489,177	Intergenic	2.69	1.59×10^{-8}

	rs12966734	18	62,492,981	Intergenic	2.49	2.66×10^{-8}
	rs112779919	21	44,024,054	Intergenic	3.00	2.02×10^{-9}
NGFR	rs181198447	5	154,659,520	Intergenic	0.10	2.13×10^{-8}
NR4A1	rs17570382	15	38,572,996	*SPRED1*	1.66	2.88×10^{-8}
PCK1	rs12122364	1	67,361,195	*WDR78*	0.03	5.16×10^{-9}
	rs56193101	6	151,263,035	*MTHFD1L*	0.03	3.85×10^{-8}
PDGFA	rs13147782	4	131,034,260	Intergenic	3.95	1.02×10^{-8}
	rs10425914	19	35,952,955	Intergenic	3.97	2.18×10^{-9}
PDGFB	rs5757584	22	39,662,550	Intergenic	-0.44	2.34×10^{-8}
	rs5757585	22	39,663,185	Intergenic	-0.44	2.18×10^{-8}
	rs5757587	22	39,663,513	Intergenic	-0.45	5.17×10^{-9}
	rs5750784	22	39,664,180	Intergenic	-0.45	3.93×10^{-9}
	rs1569495	22	39,665,142	Intergenic	-0.45	3.93×10^{-9}
	rs5757590	22	39,668,409	Intergenic	-0.45	3.93×10^{-9}
	rs968451	22	39,670,851	Intergenic	-0.45	4.25×10^{-9}
	rs5750785	22	39,671,141	Intergenic	-0.45	4.67×10^{-9}
	rs5757601	22	39,671,534	Intergenic	-0.46	3.57×10^{-9}
	rs17000962	22	39,680,187	Intergenic	-0.45	4.38×10^{-9}
	rs12628705	22	39,693,518	Intergenic	-0.44	1.00×10^{-8}
	rs11704615	22	39,696,834	Intergenic	-0.44	1.24×10^{-8}
	rs57078777	22	39,697,458	Intergenic	-0.44	1.24×10^{-8}
	rs11703434	22	39,698,915	Intergenic	-0.44	1.24×10^{-8}
	rs11704385	22	39,700,994	Intergenic	-0.43	1.61×10^{-8}
	rs62228376	22	39,701,097	Intergenic	-0.44	1.24×10^{-8}
	rs75712317	22	39,701,165	Intergenic	-0.44	1.00×10^{-8}
	rs62228377	22	39,701,703	Intergenic	-0.45	4.25×10^{-8}
	rs971964	22	39,702,473	Intergenic	-0.44	1.24×10^{-8}
	rs5757609	22	39,707,845	Intergenic	-0.43	6.58×10^{-9}
	rs5757610	22	39,707,979	Intergenic	-0.43	6.58×10^{-9}
	rs5757611	22	39,708,357	Intergenic	-0.43	6.58×10^{-9}
	rs2076125	22	39,711,351	*RPL3*	-0.43	6.58×10^{-9}
	rs2007418	22	39,712,394	*RPL3*	-0.43	6.58×10^{-9}
	rs12628367	22	39,717,104	Intergenic	-0.42	1.21×10^{-8}
	rs5757614	22	39,717,554	Intergenic	-0.43	6.58×10^{-9}
	rs12627970	22	39,721,745	Intergenic	-0.43	6.58×10^{-9}
	rs12627761	22	39,724,137	Intergenic	-0.43	6.58×10^{-9}
	rs5757618	22	39,724,551	Intergenic	-0.43	6.58×10^{-9}
	rs5757619	22	39,724,558	Intergenic	-0.43	6.58×10^{-9}
	rs5750789	22	39,725,280	Intergenic	-0.43	2.60×10^{-8}
	rs5757622	22	39,728,861	Intergenic	-0.43	2.60×10^{-8}
	rs28591484	22	39,729,249	Intergenic	-0.43	2.60×10^{-8}
	rs62228404	22	39,730,795	Intergenic	-0.43	2.60×10^{-8}
	rs12163078	22	39,731,680	Intergenic	-0.43	2.60×10^{-8}
	rs12627838	22	39,733,521	Intergenic	-0.43	2.60×10^{-8}
	rs5757624	22	39,737,350	Intergenic	-0.43	2.60×10^{-8}
	rs5757625	22	39,738,243	Intergenic	-0.44	1.06×10^{-8}
	rs74804869	22	39,741,840	Intergenic	-0.41	6.09×10^{-9}
	rs3747177	22	39,746,168	*SYNGR1*	-0.42	4.60×10^{-9}
	rs2267407	22	39,747,050	*SYNGR1*	-0.41	4.77×10^{-9}

	rs1807560	22	39,748,912	*SYNGR1*	-0.42	4.60×10^{-9}
	rs5757628	22	39,749,981	*SYNGR1*	-0.42	2.77×10^{-9}
	rs715505	22	39,751,251	*SYNGR1*	-0.41	6.48×10^{-9}
	rs61616683	22	39,755,773	*SYNGR1*	-0.42	2.77×10^{-9}
	rs2049985	22	39,756,650	*SYNGR1*	-0.42	2.77×10^{-9}
PDGFRA	rs2071997	1	4,758,169	*AJAP1*	0.94	2.99×10^{-9}
	rs72638828	1	4,767,563	*AJAP1*	0.88	1.20×10^{-8}
	rs77713630	1	13,932,387	*PDPN*	0.83	4.07×10^{-8}
	rs55965106	1	30,661,274	Intergenic	0.85	1.09×10^{-8}
	rs115303998	1	38,475,505	Intergenic	0.85	4.44×10^{-8}
	rs12067225	1	38,483,498	*UTP11L*	0.95	5.75×10^{-9}
	rs12083739	1	38,497,969	Intergenic	0.95	5.75×10^{-9}
	rs16824560	1	38,502,279	Intergenic	0.95	5.75×10^{-9}
	rs12093705	1	38,502,404	Intergenic	0.95	5.75×10^{-9}
	rs77019850	1	38,506,265	Intergenic	0.95	5.75×10^{-9}
	rs112597088	1	38,506,725	Intergenic	0.95	5.75×10^{-9}
	rs77878024	1	38,507,033	Intergenic	0.95	5.75×10^{-9}
	rs78118424	1	38,507,180	Intergenic	0.95	5.75×10^{-9}
	rs77098561	1	38,507,323	Intergenic	0.95	5.75×10^{-9}
	rs78948181	1	38,507,441	Intergenic	0.95	5.75×10^{-9}
	rs12095900	1	38,508,795	Intergenic	0.95	5.75×10^{-9}
	rs12091855	1	38,508,948	Intergenic	0.95	5.75×10^{-9}
	rs75290293	1	38,514,645	Intergenic	0.95	5.75×10^{-9}
	rs12093120	1	38,515,266	Intergenic	0.95	5.75×10^{-9}
	rs75320540	1	38,516,381	Intergenic	0.95	5.75×10^{-9}
	rs12058961	1	38,517,310	Intergenic	0.95	5.75×10^{-9}
	rs12095995	1	38,517,347	Intergenic	0.95	5.75×10^{-9}
	rs147391979	1	47,635,349	Intergenic	0.89	9.47×10^{-9}
	rs74754244	1	60,244,994	*LOC101926944*	0.93	2.49×10^{-9}
	rs11208345	1	64,518,525	*ROR1*	0.86	2.93×10^{-8}
	rs17557128	1	71,643,690	*ZRANB2-AS2*	0.94	3.66×10^{-9}
	rs17560420	1	71,835,333	Intergenic	1.01	7.91×10^{-10}
	rs34898186	1	81,146,298	Intergenic	0.89	5.47×10^{-9}
	rs148110752	1	85,653,530	*SYDE2*	0.85	1.22×10^{-8}
	rs7542970	1	99,907,647	Intergenic	0.90	1.26×10^{-8}
	rs17646665	1	109,912,051	*SORT1*	1.00	5.06×10^{-10}
	rs2272272	1	110,009,802	*SYPL2*	0.97	1.39×10^{-9}
	rs56018934	1	110,060,710	Intergenic	0.97	1.53×10^{-9}
	rs56074066	1	153,472,415	Intergenic	0.83	1.92×10^{-8}
	rs7535476	1	153,514,241	*S100A5*	0.88	1.43×10^{-8}
	rs1810765	1	153,515,120	Intergenic	0.86	2.30×10^{-8}
	rs743687	1	153,515,525	Intergenic	0.86	2.30×10^{-8}
	rs9660389	1	153,582,067	*S100A16*	1.00	7.85×10^{-10}
	rs9660416	1	153,582,264	*S100A16*	1.00	7.85×10^{-10}
	rs9728059	1	153,594,644	*S100A13*	0.92	4.65×10^{-9}
	rs9728400	1	153,595,056	*S100A13*	0.90	6.55×10^{-9}
	rs61805740	1	153,607,004	*CHTOP*	0.99	1.12×10^{-9}
	rs10158761	1	153,611,426	*CHTOP*	0.99	1.12×10^{-9}
	rs7549308	1	153,614,712	*CHTOP*	0.99	1.12×10^{-9}

rs12750815	1	200,624,809	*DDX59*	0.90	5.88×10^{-9}
rs34810707	1	222,950,692	Intergenic	0.80	3.28×10^{-8}
rs59287052	1	229,100,798	Intergenic	0.85	1.17×10^{-8}
rs7546521	1	229,103,599	Intergenic	0.85	1.59×10^{-8}
rs7546906	1	229,103,917	Intergenic	0.85	1.59×10^{-8}
rs12081816	1	229,104,679	Intergenic	0.85	1.59×10^{-8}
rs12086691	1	229,105,052	Intergenic	0.85	1.59×10^{-8}
rs61825064	1	229,105,822	Intergenic	0.81	4.66×10^{-8}
rs61825065	1	229,106,506	Intergenic	0.81	4.17×10^{-8}
rs61825066	1	229,106,644	Intergenic	0.81	4.17×10^{-8}
rs7513940	1	229,107,279	Intergenic	0.81	4.17×10^{-8}
rs111795175	1	229,107,829	Intergenic	0.81	4.17×10^{-8}
rs61825067	1	229,108,057	Intergenic	0.81	4.17×10^{-8}
rs61825068	1	229,108,108	Intergenic	0.81	4.17×10^{-8}
rs61825070	1	229,108,414	Intergenic	0.81	4.17×10^{-8}
rs145447338	1	237,493,720	*RYR2*	0.83	3.48×10^{-8}
rs72766846	1	242,752,720	Intergenic	0.88	9.40×10^{-9}
rs6734056	2	1,815,366	*MYT1L*	0.90	3.46×10^{-8}
rs113087455	2	3,720,117	*ALLC*	0.86	1.82×10^{-9}
rs184609957	2	57,418,227	Intergenic	0.80	1.34×10^{-8}
rs62150097	2	72,392,916	Intergenic	0.91	1.37×10^{-8}
rs57013742	2	72,401,702	Intergenic	0.95	5.69×10^{-9}
rs62150118	2	72,404,853	Intergenic	0.91	1.37×10^{-8}
rs62150119	2	72,407,793	*EXOC6B*	0.91	1.37×10^{-8}
rs62150120	2	72,411,493	*EXOC6B*	0.88	3.21×10^{-8}
rs17007927	2	72,413,594	*EXOC6B*	0.88	3.21×10^{-8}
rs6748698	2	72,414,107	*EXOC6B*	0.88	3.21×10^{-8}
rs6705261	2	72,414,565	*EXOC6B*	0.88	3.21×10^{-8}
rs6705347	2	72,414,575	*EXOC6B*	0.88	3.21×10^{-8}
rs58339219	2	72,415,328	*EXOC6B*	0.88	3.21×10^{-8}
rs62150123	2	72,416,709	*EXOC6B*	0.86	4.37×10^{-8}
rs55813510	2	72,417,191	*EXOC6B*	0.88	3.21×10^{-8}
rs17007929	2	72,418,724	*EXOC6B*	0.88	3.21×10^{-8}
rs13339845	2	72,419,864	*EXOC6B*	0.88	3.21×10^{-8}
rs17007933	2	72,420,151	*EXOC6B*	0.88	3.21×10^{-8}
rs17007934	2	72,424,769	*EXOC6B*	0.91	1.37×10^{-8}
rs55994075	2	72,425,112	*EXOC6B*	0.91	1.37×10^{-8}
rs6707549	2	72,427,119	*EXOC6B*	0.88	3.21×10^{-8}
rs10490039	2	72,429,239	*EXOC6B*	0.88	3.21×10^{-8}
rs17007937	2	72,432,759	*EXOC6B*	0.91	1.37×10^{-8}
rs2192012	2	72,433,660	*EXOC6B*	0.91	1.37×10^{-8}
rs72910329	2	72,436,567	*EXOC6B*	0.91	1.37×10^{-8}
rs72910330	2	72,437,144	*EXOC6B*	0.91	1.37×10^{-8}
rs62147574	2	72,437,602	*EXOC6B*	0.91	1.37×10^{-8}
rs58032693	2	72,439,600	*EXOC6B*	0.91	1.37×10^{-8}
rs58173605	2	72,439,951	*EXOC6B*	0.91	1.37×10^{-8}
rs61391258	2	72,440,145	*EXOC6B*	0.91	1.37×10^{-8}
rs62147580	2	72,441,356	*EXOC6B*	0.91	1.37×10^{-8}
rs62147581	2	72,441,476	*EXOC6B*	0.85	3.89×10^{-8}

rs62147586	2	72,447,285	*EXOC6B*	0.95	5.69×10^{-9}
rs72910350	2	72,448,427	*EXOC6B*	0.91	1.37×10^{-8}
rs10490040	2	72,449,443	*EXOC6B*	0.91	1.37×10^{-8}
rs62147587	2	72,449,468	*EXOC6B*	0.95	5.69×10^{-9}
rs62147588	2	72,449,484	*EXOC6B*	0.95	5.69×10^{-9}
rs62147590	2	72,451,666	*EXOC6B*	0.95	5.69×10^{-9}
rs1002415	2	72,455,057	*EXOC6B*	0.93	6.83×10^{-9}
rs13389007	2	72,455,964	*EXOC6B*	0.95	5.69×10^{-9}
rs59269913	2	72,457,217	*EXOC6B*	0.95	5.69×10^{-9}
rs56152907	2	72,459,305	*EXOC6B*	0.95	5.69×10^{-9}
rs62147598	2	72,459,614	*EXOC6B*	0.95	5.69×10^{-9}
rs77024759	2	72,461,309	*EXOC6B*	0.95	5.69×10^{-9}
rs62147600	2	72,461,497	*EXOC6B*	0.95	5.69×10^{-9}
rs62147601	2	72,463,898	*EXOC6B*	0.95	5.69×10^{-9}
rs62147622	2	72,466,296	*EXOC6B*	0.95	5.69×10^{-9}
rs62147623	2	72,467,134	*EXOC6B*	0.95	5.69×10^{-9}
rs62147624	2	72,471,378	*EXOC6B*	0.95	5.69×10^{-9}
rs62147625	2	72,471,453	*EXOC6B*	0.95	5.69×10^{-9}
rs10171231	2	72,480,536	*EXOC6B*	0.92	9.52×10^{-9}
rs62147627	2	72,485,205	*EXOC6B*	0.92	9.52×10^{-9}
rs62147630	2	72,489,047	*EXOC6B*	0.92	9.52×10^{-9}
rs62147631	2	72,492,585	*EXOC6B*	0.90	1.16×10^{-8}
rs61450122	2	72,495,697	*EXOC6B*	0.92	9.52×10^{-9}
rs62147637	2	72,500,268	*EXOC6B*	0.92	9.52×10^{-9}
rs62149279	2	72,558,981	*EXOC6B*	0.91	1.16×10^{-8}
rs62149280	2	72,559,455	*EXOC6B*	0.93	6.07×10^{-9}
rs62149330	2	72,704,811	*EXOC6B*	0.95	5.52×10^{-9}
rs62149336	2	72,745,105	*EXOC6B*	0.91	9.15×10^{-9}
rs189988206	2	108,346,116	Intergenic	0.88	1.10×10^{-8}
rs142867749	2	121,499,447	Intergenic	0.84	1.45×10^{-8}
rs78683586	2	121,520,061	Intergenic	0.85	1.48×10^{-8}
rs707493	2	121,521,899	Intergenic	0.83	2.71×10^{-8}
rs112657232	2	146,069,157	Intergenic	0.93	7.75×10^{-10}
rs76591903	2	164,613,084	Intergenic	0.93	3.18×10^{-9}
rs77276463	2	164,650,836	Intergenic	0.92	4.16×10^{-9}
rs77202089	2	213,767,601	Intergenic	0.85	3.29×10^{-8}
rs116802361	2	213,770,479	Intergenic	0.86	4.15×10^{-8}
rs7605968	2	217,843,682	Intergenic	0.89	2.33×10^{-8}
rs78077388	2	231,428,377	Intergenic	0.97	1.69×10^{-9}
rs80032159	2	231,430,248	Intergenic	0.97	1.69×10^{-9}
rs6707287	2	237,539,157	Intergenic	0.80	2.50×10^{-8}
rs143049900	3	29,401,020	*RBMS3*	0.81	1.12×10^{-8}
rs191333741	3	32,007,446	*OSBPL10*	0.81	1.82×10^{-8}
rs112887771	3	33,749,936	*CLASP2*	0.89	1.57×10^{-8}
rs62245694	3	65,850,448	*MAGI1*	0.87	2.30×10^{-8}
rs78486318	3	123,414,202	*MYLK*	0.79	3.79×10^{-8}
rs79737758	3	127,033,722	Intergenic	0.84	3.00×10^{-8}
rs111433903	3	185,588,107	Intergenic	0.84	1.16×10^{-8}
rs55639153	3	192,867,905	Intergenic	0.82	2.17×10^{-8}

rs73196836	3	192,897,226	Intergenic	0.81	2.55×10^{-8}
rs9680912	3	197,845,233	Intergenic	0.91	2.27×10^{-9}
rs13140818	4	30,837,989	*PCDH7*	0.96	2.47×10^{-9}
rs34547954	4	30,844,667	*PCDH7*	0.96	2.47×10^{-9}
rs56398319	4	45,671,692	Intergenic	0.87	1.90×10^{-8}
rs116590395	4	45,697,173	Intergenic	0.84	3.07×10^{-8}
rs74951460	4	45,754,024	Intergenic	0.99	9.15×10^{-10}
rs79977543	4	45,769,176	Intergenic	0.97	9.70×10^{-10}
rs17638375	4	45,777,947	Intergenic	0.97	9.70×10^{-10}
rs148521262	4	46,215,849	Intergenic	0.80	4.25×10^{-8}
rs114372540	4	46,297,741	*GABRA2*	0.85	1.75×10^{-8}
rs75533665	4	46,308,231	*GABRA2*	0.85	1.75×10^{-8}
rs78659165	4	46,336,973	*GABRA2*	0.85	1.75×10^{-8}
rs17537359	4	46,341,106	*GABRA2*	0.85	1.75×10^{-8}
rs79854595	4	46,374,069	*GABRA2*	0.95	3.39×10^{-9}
rs111660079	4	71,703,200	*GRSF1*	0.87	7.26×10^{-9}
rs79117691	4	71,810,431	*MOB1B*	0.86	9.57×10^{-9}
rs79509453	4	75,280,269	Intergenic	0.83	3.34×10^{-8}
rs75779450	4	75,281,932	Intergenic	0.91	8.52×10^{-9}
rs8180348	4	75,327,088	Intergenic	0.85	2.48×10^{-8}
rs78123185	4	91,654,081	*CCSER1*	0.94	7.40×10^{-9}
rs77704340	4	105,226,817	Intergenic	0.88	1.47×10^{-8}
rs2726175	4	108,632,261	*PAPSS1*	0.77	4.29×10^{-8}
rs17183807	4	182,234,918	Intergenic	0.84	2.06×10^{-8}
rs75281711	4	182,710,937	Intergenic	0.84	4.20×10^{-8}
rs7679898	4	190,355,762	Intergenic	0.82	1.37×10^{-8}
rs77561888	5	4,801,731	Intergenic	0.95	2.13×10^{-9}
rs79619750	5	17,821,874	Intergenic	0.82	4.94×10^{-8}
rs75226414	5	17,911,018	Intergenic	0.83	3.49×10^{-8}
rs10069923	5	18,034,385	Intergenic	0.84	9.52×10^{-9}
rs10066941	5	18,034,392	Intergenic	0.77	4.44×10^{-8}
rs10060996	5	18,034,395	Intergenic	0.82	1.75×10^{-8}
rs6889983	5	41,898,342	Intergenic	0.83	1.11×10^{-8}
rs78443884	5	55,221,681	Intergenic	0.94	8.12×10^{-9}
rs146746286	5	55,222,866	Intergenic	0.92	1.13×10^{-8}
rs78474013	5	55,226,602	Intergenic	0.90	2.11×10^{-8}
rs78973579	5	100,632,099	Intergenic	0.86	4.96×10^{-8}
rs145480261	5	100,632,978	Intergenic	0.86	4.96×10^{-8}
rs79583134	5	113,878,402	Intergenic	0.89	1.91×10^{-8}
rs142493716	5	114,161,962	Intergenic	0.85	4.20×10^{-8}
rs17465433	5	114,318,903	Intergenic	0.87	4.39×10^{-8}
rs67186626	5	118,616,559	*TNFAIP8*	0.84	3.04×10^{-8}
rs72786118	5	118,628,798	*TNFAIP8*	0.83	4.68×10^{-8}
rs6892980	5	118,635,662	*TNFAIP8*	0.83	4.68×10^{-8}
rs72786125	5	118,646,065	*TNFAIP8*	0.83	4.68×10^{-8}
rs260873	5	173,261,597	Intergenic	0.85	1.79×10^{-8}
rs260876	5	173,262,738	Intergenic	0.85	1.79×10^{-8}
rs2659190	5	173,264,384	Intergenic	0.85	1.79×10^{-8}
rs56022632	5	179,600,711	*RASGEF1C*	0.91	9.37×10^{-9}

rs55754257	6	5,271,455	*FARS2*	0.82	4.90×10^{-8}
rs7753956	6	20,538,044	*CDKAL1*	0.81	4.87×10^{-8}
rs7753670	6	20,538,045	*CDKAL1*	0.81	4.87×10^{-8}
rs77880726	6	20,618,098	*CDKAL1*	0.81	3.72×10^{-8}
rs115521560	6	32,019,382	*TNXB*	0.97	2.57×10^{-9}
rs190197731	6	33,009,588	Intergenic	0.86	2.39×10^{-8}
rs41273094	6	37,606,190	*MDGA1*	0.86	2.34×10^{-8}
rs11753500	6	37,606,827	*MDGA1*	0.86	2.34×10^{-8}
rs75714694	6	39,226,061	Intergenic	0.90	9.24×10^{-9}
rs1547079	6	135,749,202	*AHI1*	0.90	1.44×10^{-8}
rs1547080	6	135,749,315	*AHI1*	0.90	1.44×10^{-8}
rs2757640	6	135,752,813	*AHI1*	0.90	1.44×10^{-8}
rs737561	6	135,754,471	*AHI1*	0.90	1.44×10^{-8}
rs2614271	6	135,759,104	*AHI1*	0.90	1.44×10^{-8}
rs2614272	6	135,761,086	*AHI1*	0.90	1.44×10^{-8}
rs2757645	6	135,763,866	*AHI1*	0.90	1.44×10^{-8}
rs4896148	6	135,798,221	*AHI1*	0.85	3.69×10^{-8}
rs9385724	6	135,807,442	*AHI1*	0.90	1.44×10^{-8}
rs7769298	6	135,809,811	*AHI1*	0.90	1.44×10^{-8}
rs6928455	6	135,818,135	*AHI1*	0.90	1.44×10^{-8}
rs7771001	6	135,827,751	*LINC00271*	0.90	1.44×10^{-8}
rs4896151	6	135,829,796	*LINC00271*	0.90	1.44×10^{-8}
rs7753598	6	135,837,192	*LINC00271*	0.90	1.44×10^{-8}
rs73564174	6	135,844,235	*LINC00271*	0.88	1.60×10^{-8}
rs6899366	6	135,845,145	*LINC00271*	0.94	6.16×10^{-9}
rs9483847	6	135,872,148	*LINC00271*	0.90	1.44×10^{-8}
rs9494273	6	135,876,343	*LINC00271*	0.90	1.44×10^{-8}
rs79314354	6	135,876,368	*LINC00271*	0.90	1.44×10^{-8}
rs73548042	6	135,877,051	*LINC00271*	0.90	1.44×10^{-8}
rs9494274	6	135,880,480	*LINC00271*	0.90	1.44×10^{-8}
rs9483849	6	135,883,459	*LINC00271*	0.90	1.44×10^{-8}
rs9494278	6	135,885,150	*LINC00271*	0.90	1.44×10^{-8}
rs2327644	6	135,887,986	*LINC00271*	0.89	1.49×10^{-8}
rs9402720	6	135,893,950	*LINC00271*	0.90	1.44×10^{-8}
rs761358	6	135,895,908	*LINC00271*	0.90	1.44×10^{-8}
rs2092556	6	135,899,344	*LINC00271*	0.90	1.44×10^{-8}
rs2092557	6	135,899,406	*LINC00271*	0.85	3.57×10^{-8}
rs9385727	6	135,900,517	*LINC00271*	0.90	1.44×10^{-8}
rs4895453	6	135,903,717	*LINC00271*	0.90	1.44×10^{-8}
rs9376109	6	135,904,201	*LINC00271*	0.90	1.44×10^{-8}
rs9321510	6	135,904,344	*LINC00271*	0.90	1.44×10^{-8}
rs7738149	6	135,905,911	*LINC00271*	0.90	1.44×10^{-8}
rs10457011	6	135,907,028	*LINC00271*	0.90	1.44×10^{-8}
rs6939015	6	135,909,369	*LINC00271*	0.90	1.44×10^{-8}
rs6901371	6	135,910,246	*LINC00271*	0.90	1.44×10^{-8}
rs6570017	6	135,911,312	*LINC00271*	0.90	1.44×10^{-8}
rs7750367	6	135,911,705	*LINC00271*	0.90	1.44×10^{-8}
rs7750001	6	135,911,726	*LINC00271*	0.90	1.44×10^{-8}
rs7750455	6	135,912,007	*LINC00271*	0.90	1.44×10^{-8}

rs12199810	6	135,912,356	*LINC00271*	0.90	1.44×10^{-8}
rs73005187	6	148,213,847	Intergenic	0.96	1.41×10^{-9}
rs73009227	6	148,266,265	Intergenic	0.93	2.03×10^{-9}
rs7755568	6	153,076,955	*VIP*	0.85	4.76×10^{-8}
rs76286622	6	156,278,679	Intergenic	0.81	1.73×10^{-8}
rs183784545	6	170,728,095	Intergenic	0.92	1.00×10^{-8}
rs113342503	7	543,455	*PDGFA*	0.88	5.04×10^{-9}
rs3807507	7	16,791,813	Intergenic	0.92	1.01×10^{-8}
rs10227609	7	27,547,752	Intergenic	0.93	6.38×10^{-9}
rs10231814	7	27,548,852	Intergenic	0.92	6.69×10^{-9}
rs192570103	7	45,420,818	Intergenic	0.86	2.24×10^{-8}
rs73120083	7	51,406,354	Intergenic	0.90	1.98×10^{-9}
rs60180169	7	86,500,761	Intergenic	0.84	1.69×10^{-8}
rs74498030	7	92,056,778	Intergenic	0.93	9.99×10^{-9}
rs146492539	7	102,397,984	*FAM185A*	0.83	1.29×10^{-8}
rs111878132	7	129,412,870	Intergenic	0.91	2.25×10^{-8}
rs11509175	7	148,698,494	Intergenic	0.91	7.31×10^{-9}
rs142632040	7	151,230,546	Intergenic	0.83	2.29×10^{-8}
rs17501986	8	16,979,116	*MICU3*	0.82	2.72×10^{-8}
rs10088713	8	52,923,780	Intergenic	0.95	3.12×10^{-9}
rs148835832	8	69,626,660	*C8orf34*	0.96	1.62×10^{-9}
rs77747156	8	72,823,774	*LOC100132891*	0.86	2.84×10^{-8}
rs117318601	8	74,285,456	Intergenic	1.00	1.25×10^{-9}
rs35693157	8	103,207,648	Intergenic	0.81	3.29×10^{-8}
rs748020	8	125,615,666	*MTSS1*	0.90	4.92×10^{-9}
rs75912457	9	13,806,413	Intergenic	0.81	4.87×10^{-8}
rs76625048	9	111,187,033	Intergenic	0.80	3.48×10^{-8}
rs11789283	9	111,189,685	Intergenic	0.80	3.48×10^{-8}
rs75622013	9	111,191,289	Intergenic	0.80	3.48×10^{-8}
rs78988889	9	111,192,836	Intergenic	0.80	3.48×10^{-8}
rs116942211	9	111,196,400	Intergenic	0.80	3.48×10^{-8}
rs34455546	9	111,197,580	Intergenic	0.80	3.48×10^{-8}
rs78708592	9	111,198,211	Intergenic	0.80	3.48×10^{-8}
rs75369110	9	111,198,395	Intergenic	0.80	3.48×10^{-8}
rs11791386	9	111,199,204	Intergenic	0.80	3.48×10^{-8}
rs79289833	9	111,205,524	Intergenic	0.80	3.48×10^{-8}
rs117931220	9	111,208,303	Intergenic	0.82	2.77×10^{-8}
rs76437301	9	111,210,693	Intergenic	0.82	2.77×10^{-8}
rs75847842	9	111,211,027	Intergenic	0.82	2.77×10^{-8}
rs78059003	9	111,211,202	Intergenic	0.82	2.77×10^{-8}
rs11791681	9	111,211,790	Intergenic	0.82	2.77×10^{-8}
rs117228370	9	111,212,116	Intergenic	0.82	2.77×10^{-8}
rs11792813	9	111,213,713	Intergenic	0.82	2.77×10^{-8}
rs117001923	9	111,215,031	Intergenic	0.82	2.77×10^{-8}
rs117422220	9	111,215,240	Intergenic	0.82	2.77×10^{-8}
rs148524963	9	111,215,546	Intergenic	0.82	2.77×10^{-8}
rs75863099	9	111,216,637	Intergenic	0.82	2.77×10^{-8}
rs79150470	9	111,216,712	Intergenic	0.82	2.77×10^{-8}
rs76169971	9	111,216,881	Intergenic	0.82	2.77×10^{-8}

rs79953945	9	111,216,951	Intergenic	0.82	2.77×10^{-8}
rs77160051	9	111,217,312	Intergenic	0.82	2.77×10^{-8}
rs76011702	9	111,218,554	Intergenic	0.82	2.77×10^{-8}
rs11790776	9	111,218,815	Intergenic	0.82	2.77×10^{-8}
rs79702132	9	111,221,084	Intergenic	0.82	2.77×10^{-8}
rs79149655	9	111,221,452	Intergenic	0.82	2.77×10^{-8}
rs118050757	9	111,221,509	Intergenic	0.82	2.77×10^{-8}
rs11792603	9	111,231,437	Intergenic	0.82	2.77×10^{-8}
rs11795088	9	111,231,609	Intergenic	0.82	2.77×10^{-8}
rs11795100	9	111,231,677	Intergenic	0.82	2.77×10^{-8}
rs11795107	9	111,231,747	Intergenic	0.82	2.77×10^{-8}
rs75134352	9	111,232,022	Intergenic	0.82	2.77×10^{-8}
rs75824730	9	111,232,356	Intergenic	0.82	2.77×10^{-8}
rs79924554	9	111,232,431	Intergenic	0.82	2.77×10^{-8}
rs76572758	9	111,232,525	Intergenic	0.82	2.77×10^{-8}
rs76568758	9	111,232,587	Intergenic	0.82	2.77×10^{-8}
rs11793635	9	111,232,786	Intergenic	0.82	2.77×10^{-8}
rs11793660	9	111,232,885	Intergenic	0.82	2.77×10^{-8}
rs11793670	9	111,233,122	Intergenic	0.80	3.48×10^{-8}
rs76695330	9	111,234,152	Intergenic	0.82	2.77×10^{-8}
rs80285538	9	111,234,296	Intergenic	0.82	2.77×10^{-8}
rs76586402	9	111,234,988	Intergenic	0.82	2.77×10^{-8}
rs78099991	9	111,235,072	Intergenic	0.82	2.77×10^{-8}
rs75964459	9	111,235,327	Intergenic	0.82	2.77×10^{-8}
rs79399238	9	111,235,347	Intergenic	0.82	2.77×10^{-8}
rs78706615	9	111,235,494	Intergenic	0.82	2.77×10^{-8}
rs28472497	9	111,236,232	Intergenic	0.82	2.77×10^{-8}
rs79206216	9	111,236,663	Intergenic	0.82	2.77×10^{-8}
rs78100318	9	111,237,144	Intergenic	0.82	2.77×10^{-8}
rs78409632	9	111,237,210	Intergenic	0.82	2.77×10^{-8}
rs76463536	9	111,237,360	Intergenic	0.82	2.77×10^{-8}
rs78603953	9	111,238,375	Intergenic	0.82	2.77×10^{-8}
rs117548041	9	111,239,197	Intergenic	0.82	2.77×10^{-8}
rs80087940	9	111,239,406	Intergenic	0.82	2.77×10^{-8}
rs4842003	9	138,469,962	*LOC100130954*	0.85	1.45×10^{-8}
rs149710354	10	32,163,825	*ARHGAP12*	0.88	7.89×10^{-9}
rs138842148	10	44,635,901	Intergenic	0.86	2.48×10^{-8}
rs80229115	10	67,160,808	Intergenic	0.85	1.56×10^{-8}
rs148962069	10	67,943,910	*CTNNA3*	0.89	1.70×10^{-8}
rs118029533	10	67,953,378	*CTNNA3*	0.89	1.70×10^{-8}
rs75064987	10	67,963,587	*CTNNA3*	0.95	4.46×10^{-9}
rs116861785	10	68,000,935	*CTNNA3*	0.88	1.72×10^{-8}
rs78881304	10	68,012,194	*CTNNA3*	0.88	1.72×10^{-8}
rs117911839	10	68,051,162	*CTNNA3*	0.90	1.02×10^{-8}
rs72804042	10	73,660,795	Intergenic	0.88	1.44×10^{-8}
rs11814711	10	73,674,311	Intergenic	0.81	4.91×10^{-8}
rs61851387	10	80,971,456	*ZMIZ1*	0.83	3.24×10^{-8}
rs61851432	10	81,068,503	*ZMIZ1*	0.87	2.57×10^{-8}
rs34755137	10	104,326,807	*SUFU*	0.90	1.64×10^{-8}

rs11198464	10	120,269,140	Intergenic	0.82	3.71×10^{-8}
rs75622275	10	120,274,981	Intergenic	0.82	3.71×10^{-8}
rs1878427	10	120,278,805	Intergenic	0.82	3.71×10^{-8}
rs78191986	10	120,288,127	Intergenic	0.82	3.71×10^{-8}
rs76527683	10	120,288,291	Intergenic	0.82	3.71×10^{-8}
rs12255218	10	120,289,112	Intergenic	0.82	3.71×10^{-8}
rs55861953	10	120,334,998	Intergenic	0.82	3.71×10^{-8}
rs55742702	10	120,337,467	Intergenic	0.82	3.71×10^{-8}
rs2478396	10	122,223,465	*PPAPDC1A*	0.89	3.43×10^{-8}
rs2463157	10	122,230,436	*PPAPDC1A*	0.94	1.05×10^{-8}
rs12225521	11	398,487	*PKP3*	0.88	1.90×10^{-8}
rs112079256	11	18,163,766	Intergenic	0.81	3.11×10^{-8}
rs4328214	11	23,974,293	Intergenic	0.81	3.99×10^{-8}
rs71539700	11	64,614,053	Intergenic	0.87	1.18×10^{-8}
rs117156612	11	79,946,365	Intergenic	0.90	2.25×10^{-8}
rs77675320	11	79,965,253	Intergenic	0.90	1.90×10^{-8}
rs10458956	11	110,249,521	Intergenic	0.92	6.08×10^{-9}
rs12225141	11	110,250,955	Intergenic	0.87	2.18×10^{-8}
rs11213379	11	110,258,399	Intergenic	0.87	3.17×10^{-8}
rs4754442	11	110,258,883	Intergenic	0.88	1.94×10^{-8}
rs11213383	11	110,259,766	Intergenic	0.88	1.94×10^{-8}
rs10466564	11	110,260,925	Intergenic	0.88	1.94×10^{-8}
rs10466565	11	110,260,968	Intergenic	0.88	1.94×10^{-8}
rs10466566	11	110,261,035	Intergenic	0.88	1.94×10^{-8}
rs10128698	11	110,261,921	Intergenic	0.85	3.52×10^{-8}
rs10891096	11	110,262,743	Intergenic	0.88	1.94×10^{-8}
rs12295722	11	110,266,637	Intergenic	0.88	1.94×10^{-8}
rs12288784	11	110,266,787	Intergenic	0.85	3.52×10^{-8}
rs4753890	11	110,271,912	Intergenic	0.88	1.94×10^{-8}
rs111890121	11	110,276,553	Intergenic	0.88	1.94×10^{-8}
rs11600412	11	110,277,028	Intergenic	0.85	3.52×10^{-8}
rs12286289	11	110,284,974	Intergenic	0.88	1.94×10^{-8}
rs12279767	11	110,286,345	Intergenic	0.85	3.52×10^{-8}
rs11537140	11	110,290,587	Intergenic	0.85	2.97×10^{-8}
rs4612769	11	110,292,442	Intergenic	0.83	4.94×10^{-8}
rs4309126	11	110,292,583	Intergenic	0.88	1.94×10^{-8}
rs11213393	11	110,293,177	Intergenic	0.85	3.52×10^{-8}
rs11213396	11	110,296,603	Intergenic	0.88	1.94×10^{-8}
rs12271353	11	110,298,026	Intergenic	0.88	1.94×10^{-8}
rs3759018	11	110,299,437	Intergenic	0.90	1.70×10^{-8}
rs12294963	11	110,324,894	*FDX1*	0.88	1.94×10^{-8}
rs10891117	11	110,326,306	*FDX1*	0.88	1.94×10^{-8}
rs112562144	11	110,327,380	*FDX1*	0.88	1.94×10^{-8}
rs11213416	11	110,335,761	Intergenic	0.89	1.42×10^{-8}
rs11213417	11	110,335,791	Intergenic	0.88	1.94×10^{-8}
rs7930277	11	110,336,589	Intergenic	0.83	4.94×10^{-8}
rs12273095	11	110,337,897	Intergenic	0.88	1.94×10^{-8}
rs12280232	11	110,341,416	Intergenic	0.88	1.94×10^{-8}
rs11604389	11	110,343,502	Intergenic	0.98	1.51×10^{-9}

rs10789774	11	110,345,609	Intergenic	0.85	3.52×10^{-8}
rs11213426	11	110,349,093	Intergenic	0.88	1.94×10^{-8}
rs12279406	11	110,353,314	Intergenic	0.86	2.77×10^{-8}
rs10891123	11	110,354,193	Intergenic	0.88	1.94×10^{-8}
rs10891124	11	110,357,002	Intergenic	0.86	2.77×10^{-8}
rs11213434	11	110,357,423	Intergenic	0.88	1.94×10^{-8}
rs12416743	11	110,358,849	Intergenic	0.89	1.38×10^{-8}
rs73018155	11	110,359,517	Intergenic	0.88	1.94×10^{-8}
rs56261230	11	110,361,952	Intergenic	0.94	3.86×10^{-9}
rs894560	11	110,362,655	Intergenic	0.84	4.63×10^{-8}
rs79860085	11	123,577,937	Intergenic	0.88	1.27×10^{-8}
rs75522719	11	123,586,519	Intergenic	0.86	1.97×10^{-8}
rs77286281	11	123,616,807	Intergenic	0.84	3.07×10^{-8}
rs11605363	11	123,670,457	Intergenic	0.83	2.89×10^{-8}
rs11219462	11	123,990,245	*VWA5A*	0.93	1.55×10^{-9}
rs76833948	11	128,596,369	*FLI1*	0.88	2.26×10^{-8}
rs61909570	11	131,012,797	Intergenic	0.89	2.03×10^{-8}
rs79273268	11	134,223,355	*GLB1L2*	0.97	2.42×10^{-9}
rs72647360	12	861,105	Intergenic	0.83	2.85×10^{-8}
rs113260958	12	880,820	*WNK1*	0.83	3.80×10^{-8}
rs77936233	12	888,504	*WNK1*	1.00	1.12×10^{-9}
rs12314329	12	909,731	*WNK1*	0.87	1.18×10^{-8}
rs11064544	12	921,471	*WNK1*	0.87	1.18×10^{-8}
rs72648617	12	924,905	*WNK1*	0.87	1.18×10^{-8}
rs78772771	12	982,088	*WNK1*	0.94	3.42×10^{-9}
rs72650711	12	990,401	*WNK1*	0.94	3.42×10^{-9}
rs113230037	12	991,512	*WNK1*	0.94	3.42×10^{-9}
rs72650739	12	996,060	*WNK1*	0.94	3.42×10^{-9}
rs72650748	12	997,504	*WNK1*	0.94	3.42×10^{-9}
rs111850351	12	1,000,565	*WNK1*	0.91	5.94×10^{-9}
rs56291311	12	1,003,988	*WNK1*	0.91	5.94×10^{-9}
rs11571468	12	1,024,688	*RAD52*	0.91	5.94×10^{-9}
rs11571461	12	1,026,319	*RAD52*	0.91	5.94×10^{-9}
rs111359153	12	1,028,796	*RAD52*	0.91	5.94×10^{-9}
rs74809499	12	1,029,865	*RAD52*	0.91	5.94×10^{-9}
rs11571453	12	1,034,278	*RAD52*	0.91	5.94×10^{-9}
rs11571446	12	1,035,817	*RAD52*	0.91	5.94×10^{-9}
rs11571439	12	1,036,754	*RAD52*	0.91	5.94×10^{-9}
rs11571435	12	1,037,288	*RAD52*	0.91	5.94×10^{-9}
rs79707121	12	1,044,493	*RAD52*	0.91	5.94×10^{-9}
rs11571370	12	1,060,077	Intergenic	0.89	9.59×10^{-9}
rs111428366	12	1,064,889	Intergenic	0.95	2.03×10^{-9}
rs76202538	12	1,068,333	Intergenic	0.95	2.03×10^{-9}
rs77148121	12	1,078,064	Intergenic	0.84	2.33×10^{-8}
rs60762427	12	1,078,225	Intergenic	0.82	3.44×10^{-8}
rs76902794	12	21,885,429	Intergenic	0.84	2.64×10^{-8}
rs117373058	12	27,277,185	Intergenic	0.83	4.45×10^{-8}
rs56944757	12	27,757,515	*PPFIBP1*	0.85	1.71×10^{-8}
rs6487619	12	27,769,030	*PPFIBP1*	0.82	3.11×10^{-8}

rs10842953	12	27,771,749	*PPFIBP1*	0.82	3.11×10^{-8}
rs147524932	12	28,098,494	Intergenic	0.86	1.82×10^{-8}
rs16919514	12	32,386,414	*BICD1*	0.94	7.33×10^{-9}
rs7969641	12	44,310,268	*TMEM117*	0.87	4.22×10^{-9}
rs11182326	12	44,310,333	*TMEM117*	0.87	4.22×10^{-9}
rs11182327	12	44,310,418	*TMEM117*	0.79	1.42×10^{-8}
rs10880586	12	44,310,604	*TMEM117*	0.79	1.42×10^{-8}
rs11182328	12	44,311,277	*TMEM117*	0.87	4.22×10^{-9}
rs10880588	12	44,311,787	*TMEM117*	0.79	1.42×10^{-8}
rs10785470	12	44,311,983	*TMEM117*	0.79	1.42×10^{-8}
rs10785471	12	44,311,998	*TMEM117*	0.79	1.42×10^{-8}
rs7964465	12	44,312,649	*TMEM117*	0.79	1.42×10^{-8}
rs10785472	12	44,312,774	*TMEM117*	0.79	1.42×10^{-8}
rs12369569	12	44,313,000	*TMEM117*	0.79	1.42×10^{-8}
rs10880589	12	44,313,193	*TMEM117*	0.79	1.42×10^{-8}
rs7956825	12	44,313,718	*TMEM117*	0.79	1.42×10^{-8}
rs7974501	12	44,313,794	*TMEM117*	0.79	1.42×10^{-8}
rs7971487	12	44,313,830	*TMEM117*	0.79	1.42×10^{-8}
rs7957074	12	44,313,906	*TMEM117*	0.79	1.42×10^{-8}
rs73100931	12	44,314,388	*TMEM117*	0.79	1.42×10^{-8}
rs11182329	12	44,314,564	*TMEM117*	0.79	1.42×10^{-8}
rs10880591	12	44,314,630	*TMEM117*	0.79	1.42×10^{-8}
rs2031056	12	44,315,008	*TMEM117*	0.79	1.42×10^{-8}
rs10748379	12	44,315,397	*TMEM117*	0.79	1.42×10^{-8}
rs10748380	12	44,315,492	*TMEM117*	0.79	1.42×10^{-8}
rs4238092	12	44,315,762	*TMEM117*	0.87	4.22×10^{-9}
rs4238093	12	44,315,925	*TMEM117*	0.79	1.42×10^{-8}
rs10506232	12	44,316,485	*TMEM117*	0.79	1.42×10^{-8}
rs10785473	12	44,316,584	*TMEM117*	0.79	1.42×10^{-8}
rs11182332	12	44,317,599	*TMEM117*	1.01	1.83×10^{-10}
rs10785474	12	44,318,428	*TMEM117*	0.79	1.42×10^{-8}
rs10785477	12	44,318,723	*TMEM117*	0.79	1.42×10^{-8}
rs10785478	12	44,320,156	*TMEM117*	0.79	1.42×10^{-8}
rs10785479	12	44,320,184	*TMEM117*	0.79	1.42×10^{-8}
rs11182337	12	44,320,875	*TMEM117*	0.79	1.42×10^{-8}
rs12301015	12	44,321,160	*TMEM117*	0.87	4.22×10^{-9}
rs11829881	12	44,322,395	*TMEM117*	0.87	4.22×10^{-9}
rs11830410	12	44,322,978	*TMEM117*	0.97	8.34×10^{-10}
rs4417371	12	44,327,407	*TMEM117*	0.79	1.42×10^{-8}
rs3925072	12	44,327,565	*TMEM117*	0.79	1.42×10^{-8}
rs3925073	12	44,327,736	*TMEM117*	0.79	1.42×10^{-8}
rs11182339	12	44,331,841	*TMEM117*	0.77	2.16×10^{-8}
rs12305959	12	44,332,618	*TMEM117*	0.79	1.42×10^{-8}
rs7312373	12	44,332,843	*TMEM117*	0.79	1.42×10^{-8}
rs10880597	12	44,336,172	*TMEM117*	0.79	1.42×10^{-8}
rs10880598	12	44,336,375	*TMEM117*	0.79	1.42×10^{-8}
rs10880599	12	44,337,662	*TMEM117*	0.77	1.97×10^{-8}
rs73100975	12	44,339,016	*TMEM117*	0.79	1.42×10^{-8}
rs11182341	12	44,339,216	*TMEM117*	0.79	1.42×10^{-8}

rs12303804	12	44,340,018	*TMEM117*	0.87	4.22×10^{-9}
rs10785482	12	44,342,910	*TMEM117*	0.79	1.42×10^{-8}
rs12309681	12	44,343,202	*TMEM117*	0.87	4.22×10^{-9}
rs10880600	12	44,343,641	*TMEM117*	0.80	9.50×10^{-9}
rs10880601	12	44,343,768	*TMEM117*	0.79	1.42×10^{-8}
rs73085705	12	44,345,490	*TMEM117*	1.01	1.83×10^{-10}
rs12315094	12	44,345,773	*TMEM117*	0.87	4.22×10^{-9}
rs11182345	12	44,347,202	*TMEM117*	0.87	4.22×10^{-9}
rs10880604	12	44,349,398	*TMEM117*	0.79	1.42×10^{-8}
rs10880605	12	44,349,669	*TMEM117*	0.79	1.42×10^{-8}
rs11182346	12	44,350,097	*TMEM117*	0.79	1.42×10^{-8}
rs141826304	12	49,711,077	*LOC101927267*	0.85	2.17×10^{-8}
rs1270901	12	49,835,245	*SPATS2*	0.83	3.51×10^{-8}
rs73112192	12	50,043,760	*FMNL3*	0.85	2.70×10^{-8}
rs6581474	12	63,607,898	Intergenic	0.87	1.17×10^{-8}
rs116945474	12	100,161,401	*ANKS1B*	0.85	1.82×10^{-8}
rs117095921	12	100,161,834	*ANKS1B*	0.85	1.82×10^{-8}
rs34998821	12	107,010,970	*LOC100287944; RFX4*	0.91	1.15×10^{-8}
rs71452919	12	107,021,528	*LOC100287944; RFX4*	0.91	1.15×10^{-8}
rs34956441	12	107,034,597	*LOC100287944; RFX4*	0.91	1.15×10^{-8}
rs71452921	12	107,053,762	*LOC100287944; RFX4*	0.91	1.15×10^{-8}
rs12827304	12	107,101,769	*LOC100287944; RFX4*	0.91	1.15×10^{-8}
rs12833344	12	107,142,307	*LOC100287944; RFX4*	0.92	9.08×10^{-9}
rs78694549	12	107,205,456	*RIC8B*	0.95	5.92×10^{-9}
rs71452928	12	107,259,529	*RIC8B*	0.91	1.15×10^{-8}
rs118045072	12	107,279,615	*RIC8B*	0.93	8.53×10^{-9}
rs79486147	12	121,746,542	*ANAPC5*	0.89	3.24×10^{-8}
rs11838915	13	22,631,900	Intergenic	0.85	2.30×10^{-8}
rs2146681	13	22,635,412	Intergenic	0.85	2.30×10^{-8}
rs12859446	13	22,646,974	Intergenic	0.90	6.70×10^{-9}
rs3927455	13	43,285,214	Intergenic	0.93	1.62×10^{-9}
rs34760901	13	46,014,253	Intergenic	0.73	4.44×10^{-8}
rs112539271	13	46,026,944	Intergenic	0.75	3.69×10^{-8}
rs71431371	13	46,026,985	Intergenic	0.76	3.21×10^{-8}
rs12877658	13	46,027,084	Intergenic	0.76	2.93×10^{-8}
rs74873051	13	46,035,031	Intergenic	0.94	1.37×10^{-9}
rs71431373	13	46,041,497	*COG3*	0.77	3.17×10^{-8}
rs71431375	13	46,042,206	*COG3*	0.80	1.67×10^{-8}
rs35391963	13	46,042,998	*COG3*	0.77	2.87×10^{-8}
rs12585422	13	46,045,087	*COG3*	0.77	2.87×10^{-8}
rs12877431	13	46,048,613	*COG3*	0.77	2.87×10^{-8}
rs3751352	13	46,050,658	*COG3*	0.77	2.87×10^{-8}
rs3829342	13	46,050,745	*COG3*	0.77	2.87×10^{-8}
rs3794331	13	46,053,544	*COG3*	0.77	2.87×10^{-8}
rs34436430	13	46,055,795	*COG3*	0.77	2.87×10^{-8}
rs12583589	13	46,056,913	*COG3*	0.77	2.87×10^{-8}
rs12583793	13	46,057,286	*COG3*	0.77	2.87×10^{-8}
rs12585290	13	46,062,159	*COG3*	0.77	2.87×10^{-8}
rs35772078	13	46,062,563	*COG3*	0.75	4.99×10^{-8}

rs12877513	13	46,062,869	*COG3*	0.75	4.99×10^{-8}
rs12869186	13	46,064,675	*COG3*	0.75	4.99×10^{-8}
rs76888824	13	46,068,416	*COG3*	0.75	4.99×10^{-8}
rs12871388	13	46,068,483	*COG3*	0.75	4.99×10^{-8}
rs71431379	13	46,069,203	*COG3*	0.75	4.99×10^{-8}
rs71431380	13	46,072,566	*COG3*	0.75	4.99×10^{-8}
rs35441087	13	46,075,524	*COG3*	0.75	4.99×10^{-8}
rs34973279	13	46,080,863	*COG3*	0.75	4.99×10^{-8}
rs71431381	13	46,085,369	*COG3*	0.75	4.99×10^{-8}
rs34843311	13	46,088,121	*COG3*	0.75	4.99×10^{-8}
rs35485445	13	46,098,728	*COG3*	0.75	4.99×10^{-8}
rs9652191	13	46,108,679	*COG3*	0.75	4.99×10^{-8}
rs9595339	13	46,108,691	*COG3*	0.75	4.99×10^{-8}
rs75847672	13	46,116,436	*ERICH6B*	0.82	9.12×10^{-9}
rs12430521	13	71,574,642	Intergenic	0.94	1.04×10^{-9}
rs77621781	13	100,498,719	*CLYBL; LOC101927437*	0.86	2.88×10^{-8}
rs76756924	13	100,511,798	*CLYBL; LOC101927437*	0.92	6.60×10^{-9}
rs12587813	14	25,170,852	Intergenic	0.85	2.19×10^{-8}
rs72638491	14	25,179,546	Intergenic	0.83	4.05×10^{-8}
rs10135160	14	25,187,007	Intergenic	0.83	4.05×10^{-8}
rs61976784	14	25,187,939	Intergenic	0.83	4.05×10^{-8}
rs7159732	14	25,189,706	Intergenic	0.83	4.05×10^{-8}
rs61976787	14	25,195,408	Intergenic	0.83	4.05×10^{-8}
rs72638492	14	25,196,787	Intergenic	0.83	4.05×10^{-8}
rs12323449	14	25,196,793	Intergenic	0.83	4.05×10^{-8}
rs61976788	14	25,197,424	Intergenic	0.83	4.05×10^{-8}
rs147369410	14	25,201,284	Intergenic	0.83	4.05×10^{-8}
rs61976791	14	25,206,801	Intergenic	0.93	6.70×10^{-9}
rs73288879	14	51,502,593	*TRIM9*	0.83	2.69×10^{-8}
rs113785288	14	51,506,524	*TRIM9*	0.83	2.69×10^{-8}
rs73288888	14	51,508,525	*TRIM9*	0.80	4.66×10^{-8}
rs73288894	14	51,514,642	*TRIM9*	0.83	2.69×10^{-8}
rs73288895	14	51,515,228	*TRIM9*	0.83	2.69×10^{-8}
rs76481199	14	51,515,424	*TRIM9*	0.83	2.69×10^{-8}
rs59978383	14	51,516,134	*TRIM9*	0.83	2.69×10^{-8}
rs41525444	14	55,136,767	*SAMD4A*	0.85	3.62×10^{-8}
rs17185050	14	68,046,913	*PLEKHH1*	0.81	3.98×10^{-8}
rs7148140	14	88,587,741	Intergenic	0.87	9.99×10^{-9}
rs111686083	14	94,565,997	*IFI27L1*	0.95	1.23×10^{-9}
rs1243163	14	94,844,706	*SERPINA1*	0.94	2.79×10^{-9}
rs113040969	14	94,852,812	*SERPINA1*	0.94	2.79×10^{-9}
rs112209366	14	94,874,633	Intergenic	0.79	4.47×10^{-8}
rs148563267	15	22,823,001	Intergenic	0.81	3.58×10^{-8}
rs79461254	15	22,829,399	Intergenic	0.81	3.24×10^{-8}
rs148862869	15	22,831,198	Intergenic	0.91	8.84×10^{-9}
rs188997843	15	22,831,822	Intergenic	0.94	4.06×10^{-9}
rs75194988	15	64,186,359	Intergenic	0.96	3.97×10^{-9}
rs72741031	15	67,198,566	Intergenic	0.83	2.04×10^{-8}
rs80279633	15	97,561,675	Intergenic	0.91	3.31×10^{-9}

rs77316226	15	97,566,542	Intergenic	0.91	3.31×10^{-9}
rs74949876	15	97,570,531	Intergenic	0.91	3.31×10^{-9}
rs78192416	15	97,572,117	Intergenic	0.89	4.42×10^{-9}
rs75081365	15	97,576,106	Intergenic	0.89	4.42×10^{-9}
rs75027914	15	97,580,025	Intergenic	0.91	3.31×10^{-9}
rs1399070	15	97,581,901	Intergenic	0.91	3.31×10^{-9}
rs112346031	15	97,584,663	Intergenic	0.91	3.31×10^{-9}
rs75470401	15	97,586,599	Intergenic	0.91	3.31×10^{-9}
rs12934777	16	13,808,752	Intergenic	0.85	3.11×10^{-8}
rs11074571	16	23,713,065	*ERN2*	0.87	2.81×10^{-8}
rs75025260	16	78,825,170	*WWOX*	0.86	2.32×10^{-8}
rs117900066	16	78,825,428	*WWOX*	0.84	3.24×10^{-8}
rs118022621	16	78,826,034	*WWOX*	0.86	2.20×10^{-8}
rs73575631	16	78,826,299	*WWOX*	0.86	2.20×10^{-8}
rs7189879	16	78,826,367	*WWOX*	0.84	2.96×10^{-8}
rs117855422	16	78,826,747	*WWOX*	0.86	2.20×10^{-8}
rs117276031	16	78,827,076	*WWOX*	0.86	2.20×10^{-8}
rs117052407	16	78,827,213	*WWOX*	0.86	2.20×10^{-8}
rs62038631	16	78,827,528	*WWOX*	0.83	3.41×10^{-8}
rs62038632	16	78,828,557	*WWOX*	0.83	3.41×10^{-8}
rs62038633	16	78,828,838	*WWOX*	0.83	3.41×10^{-8}
rs62038634	16	78,832,101	*WWOX*	0.81	3.89×10^{-8}
rs62038635	16	78,834,303	*WWOX*	0.83	2.65×10^{-8}
rs62038638	16	78,834,449	*WWOX*	0.85	1.99×10^{-8}
rs112463288	16	78,835,122	*WWOX*	0.85	1.99×10^{-8}
rs62038640	16	78,836,182	*WWOX*	0.85	1.99×10^{-8}
rs62038641	16	78,836,195	*WWOX*	0.85	1.99×10^{-8}
rs62038642	16	78,836,219	*WWOX*	0.85	1.99×10^{-8}
rs62038643	16	78,836,412	*WWOX*	0.83	2.89×10^{-8}
rs62038644	16	78,836,498	*WWOX*	0.83	2.89×10^{-8}
rs79974905	16	78,878,405	*WWOX*	0.84	3.93×10^{-8}
rs76362068	16	78,878,979	*WWOX*	0.86	2.12×10^{-8}
rs112045810	16	78,879,773	*WWOX*	0.86	2.12×10^{-8}
rs80051429	16	78,881,302	*WWOX*	0.86	2.12×10^{-8}
rs78788905	16	78,881,730	*WWOX*	0.86	2.12×10^{-8}
rs13335885	16	78,882,650	*WWOX*	0.85	3.66×10^{-8}
rs75244456	16	78,882,894	*WWOX*	0.86	2.12×10^{-8}
rs79676606	16	78,883,576	*WWOX*	0.83	4.68×10^{-8}
rs76751158	16	78,883,628	*WWOX*	0.89	1.31×10^{-8}
rs112168623	16	78,885,583	*WWOX*	0.89	1.31×10^{-8}
rs74035122	16	78,886,189	*WWOX*	0.83	3.80×10^{-8}
rs148305710	16	78,888,650	*WWOX*	0.86	1.74×10^{-8}
rs78882345	16	78,888,872	*WWOX*	0.89	1.31×10^{-8}
rs74576447	16	78,889,221	*WWOX*	0.89	1.31×10^{-8}
rs79695127	16	78,894,691	*WWOX*	0.87	1.79×10^{-8}
rs112746092	16	78,895,750	*WWOX*	0.89	1.58×10^{-8}
rs184871667	16	78,896,131	*WWOX*	0.91	1.27×10^{-8}
rs76873778	16	78,896,449	*WWOX*	0.92	8.72×10^{-9}
rs111261059	16	78,896,852	*WWOX*	0.92	8.72×10^{-9}

	rs78346572	16	78,897,618	*WWOX*	0.92	8.72×10^{-9}
	rs190475654	16	89,268,380	Intergenic	0.80	1.78×10^{-8}
	rs137863414	17	144,412	*RPH3AL*	0.89	2.61×10^{-8}
	rs78304829	17	21,203,820	*MAP2K3*	-1.73	4.58×10^{-9}
	rs75854955	17	21,203,825	*MAP2K3*	-1.73	4.58×10^{-9}
	rs34105301	17	21,203,893	*MAP2K3*	-1.73	4.58×10^{-9}
	rs62057672	17	21,203,934	*MAP2K3*	-1.73	4.58×10^{-9}
	rs62057673	17	21,203,949	*MAP2K3*	-1.57	2.56×10^{-8}
	rs62057674	17	21,203,998	*MAP2K3*	1.88	1.12×10^{-9}
	rs56216806	17	21,204,192	*MAP2K3*	1.71	1.41×10^{-9}
	rs55796947	17	21,204,210	*MAP2K3*	1.71	1.41×10^{-9}
	rs72831582	17	21,205,406	*MAP2K3*	1.61	4.82×10^{-8}
	rs1657695	17	21,208,413	*MAP2K3*	-1.76	2.35×10^{-9}
	rs1657684	17	21,217,411	*MAP2K3*	-1.55	4.26×10^{-8}
	rs117260204	17	29,754,079	*RAB11FIP4*	0.95	5.98×10^{-9}
	rs143420844	17	48,503,419	Intergenic	0.94	6.21×10^{-9}
	rs77582510	17	71,141,917	Intergenic	0.78	4.75×10^{-8}
	rs79487192	17	71,147,296	Intergenic	0.78	4.79×10^{-8}
	rs138848149	18	350,797	*COLEC12*	0.82	3.37×10^{-8}
	rs117949098	18	9,668,569	Intergenic	0.84	4.90×10^{-8}
	rs117333808	18	31,634,885	*NOL4*	0.89	1.53×10^{-8}
	rs117181130	18	31,643,521	*NOL4*	0.89	1.53×10^{-8}
	rs181600518	18	53,032,497	*TCF4*	0.93	9.42×10^{-9}
	rs75654082	18	67,280,948	*DOK6*	0.83	3.28×10^{-8}
	rs72618590	19	1,037,941	*CNN2*	0.91	5.98×10^{-9}
	rs117187726	19	1,077,674	*HMHA1*	0.91	8.90×10^{-9}
	rs7253257	19	4,228,337	Intergenic	0.98	1.88×10^{-9}
	rs11671081	19	20,212,026	*ZNF90*	1.00	1.05×10^{-9}
	rs35138704	19	35,897,981	*LOC100128682*	0.75	4.37×10^{-8}
	rs117878840	19	40,238,379	Intergenic	0.88	1.40×10^{-8}
	rs10408005	19	44,437,489	*ZNF45*	0.82	1.39×10^{-8}
	rs73065065	19	52,417,100	Intergenic	0.87	1.18×10^{-8}
	rs73057650	19	54,198,788	Intergenic	0.85	2.68×10^{-8}
	rs79854277	20	29,811,379	Intergenic	1.00	1.64×10^{-10}
	rs79950712	20	29,844,516	Intergenic	0.94	6.31×10^{-10}
	rs76073627	20	29,844,621	Intergenic	0.94	6.31×10^{-10}
	rs28448705	20	61,178,845	Intergenic	0.83	3.82×10^{-8}
	rs188482663	21	44,449,827	*PKNOX1*	0.80	4.15×10^{-8}
	rs17402286	22	26,894,879	*TFIP11*	0.93	6.03×10^{-9}
	rs17404166	22	26,969,802	*TPST2*	0.89	1.36×10^{-8}
	rs73163286	22	27,059,724	*MIAT*	0.92	8.73×10^{-9}
	rs138027893	22	32,335,786	*C22orf24*	0.82	3.75×10^{-8}
PHLPP2	rs56262219	8	6,142,344	Intergenic	0.68	1.88×10^{-8}
PIK3CA	rs77458083	2	11,002,868	Intergenic	4.41	4.67×10^{-8}
	rs9862771	3	143,828,430	Intergenic	4.64	6.70×10^{-9}
	rs9841242	3	143,949,314	Intergenic	6.71	1.67×10^{-12}
	rs11766153	7	35,353,014	Intergenic	3.42	1.29×10^{-8}
	rs2075123	7	35,354,260	*LOC401324*	3.24	4.57×10^{-8}
	rs190184664	11	401,756	*PKP3*	4.10	4.27×10^{-8}

	rs4768117	12	39,665,227	Intergenic	4.96	3.15×10^{-10}
	rs4768736	12	39,685,533	Intergenic	4.41	3.97×10^{-9}
	rs1013202	12	39,697,638	*KIF21A*	4.30	5.64×10^{-9}
	rs78090720	12	39,744,381	*KIF21A*	4.30	5.64×10^{-9}
	rs80329996	12	39,766,618	*KIF21A*	4.30	5.64×10^{-9}
	rs76126034	12	39,948,455	*ABCD2*	4.34	3.16×10^{-9}
	rs12423225	12	39,977,900	*ABCD2*	4.28	5.04×10^{-9}
	rs12582802	12	39,985,206	*ABCD2*	4.40	2.43×10^{-9}
	rs16950399	15	27,997,638	Intergenic	4.62	1.67×10^{-8}
	rs16950402	15	28,001,858	*OCA2*	4.70	1.38×10^{-8}
	rs73370353	15	28,002,262	*OCA2*	4.55	2.80×10^{-8}
	rs17668418	18	31,068,387	Intergenic	4.52	4.02×10^{-8}
PIK3R3	rs111253804	7	71,006,385	*WBSCR17*	3.92	2.99×10^{-9}
PPP2CB	rs7615616	3	177,219,559	*LINC00578*	1.09	3.33×10^{-8}
	rs2133623	3	177,250,426	*LINC00578*	1.12	4.64×10^{-8}
PPP2R2C	rs17488560	10	24,055,881	*KIAA1217*	0.08	1.77×10^{-8}
PPP2R5A	rs10863953	1	212,365,800	Intergenic	0.65	2.23×10^{-8}
	rs7530375	1	212,371,305	Intergenic	0.64	2.84×10^{-8}
	rs11119879	1	212,372,348	Intergenic	0.64	2.84×10^{-8}
	rs10863954	1	212,373,286	Intergenic	0.64	2.84×10^{-8}
	rs1967114	1	212,375,336	Intergenic	0.65	2.09×10^{-8}
	rs11119883	1	212,387,260	Intergenic	0.66	1.37×10^{-8}
	rs4558048	1	212,388,789	Intergenic	0.64	3.12×10^{-8}
	rs12028026	1	212,390,135	Intergenic	0.66	1.76×10^{-8}
PPP2R5D	rs71500632	1	71,533,033	*ZRANB2*	2.48	1.16×10^{-8}
	rs12748327	1	71,597,419	*ZRANB2-AS2*	2.49	1.52×10^{-8}
	rs11578076	1	71,633,719	*ZRANB2-AS2*	2.42	2.06×10^{-8}
	rs12085188	1	106,644,224	Intergenic	2.46	3.14×10^{-8}
	rs1334484	1	106,644,303	Intergenic	2.46	3.14×10^{-8}
	rs12998270	2	118,995,694	Intergenic	2.62	2.80×10^{-8}
	rs34766043	2	119,001,719	Intergenic	2.62	2.80×10^{-8}
	rs34870009	2	119,010,835	Intergenic	2.91	9.22×10^{-9}
	rs67061610	2	119,059,751	Intergenic	2.80	1.89×10^{-8}
	rs180985129	2	129,205,502	Intergenic	2.50	7.64×10^{-9}
	rs7694095	4	88,701,214	Intergenic	2.72	2.96×10^{-8}
	rs55950365	5	72,601,127	Intergenic	2.23	1.36×10^{-8}
	rs74863536	6	136,778,260	*MAP7*	2.22	4.11×10^{-8}
	rs76061187	7	13,293,857	Intergenic	3.07	3.34×10^{-9}
	rs112100380	9	98,202,016	Intergenic	2.90	1.46×10^{-8}
	rs78309973	9	111,941,810	*EPB41L4B*	2.90	4.55×10^{-8}
	rs12376648	9	111,950,394	*EPB41L4B*	2.90	4.55×10^{-8}
	rs73517300	9	111,952,926	*EPB41L4B*	2.90	4.55×10^{-8}
	rs73519210	9	111,956,157	*EPB41L4B*	2.90	4.55×10^{-8}
	rs10979741	9	111,956,494	*EPB41L4B*	2.90	4.55×10^{-8}
	rs10979743	9	111,957,173	*EPB41L4B*	2.90	4.55×10^{-8}
	rs10816783	9	111,958,426	*EPB41L4B*	2.90	4.55×10^{-8}
	rs79727364	9	111,964,694	*EPB41L4B*	2.90	4.55×10^{-8}
	rs7026902	9	111,965,202	*EPB41L4B*	2.90	4.55×10^{-8}
	rs10979744	9	111,966,928	*EPB41L4B*	2.90	4.55×10^{-8}

	rs10979745	9	111,968,861	*EPB41L4B*	2.90	4.55×10^{-8}
	rs10979746	9	111,968,934	*EPB41L4B*	2.90	4.55×10^{-8}
	rs10979747	9	111,969,135	*EPB41L4B*	2.90	4.55×10^{-8}
	rs112867677	9	111,977,792	*EPB41L4B*	2.86	3.89×10^{-8}
	rs149938415	10	87,346,847	*GRID1-AS1*	2.92	1.07×10^{-8}
	rs77976920	10	87,356,377	*GRID1-AS1*	2.92	1.07×10^{-8}
	rs76088362	10	87,629,180	*GRID1*	3.10	6.65×10^{-9}
	rs182533869	11	23,007,867	Intergenic	3.03	2.48×10^{-9}
	rs76663324	13	62,062,128	Intergenic	2.36	3.10×10^{-8}
	rs77825385	14	59,324,562	*LOC102723742*	3.01	1.53×10^{-8}
	rs11637534	15	41,208,139	Intergenic	3.31	2.63×10^{-10}
PRKCA	rs10753542	1	23,065,902	*EPHB2*	0.62	1.95×10^{-10}
	rs75877521	2	306,489	Intergenic	0.45	3.93×10^{-8}
	rs11678872	2	45,568,888	Intergenic	0.61	1.24×10^{-8}
	rs74491823	3	143,593,286	Intergenic	0.72	1.75×10^{-11}
	rs35190805	3	146,587,878	Intergenic	0.50	1.20×10^{-8}
	rs13133632	4	70,197,624	Intergenic	0.51	1.50×10^{-8}
	rs73818525	5	158,863,387	Intergenic	0.57	8.27×10^{-9}
	rs112629596	6	48,904,398	Intergenic	0.60	8.19×10^{-10}
	rs9457557	6	159,545,322	Intergenic	0.67	3.30×10^{-10}
	rs62431955	6	159,545,755	Intergenic	0.67	6.06×10^{-10}
	rs56383199	6	159,551,255	Intergenic	0.67	6.06×10^{-10}
	rs61197787	6	159,551,624	Intergenic	0.67	3.30×10^{-10}
	rs76683707	7	10,135,723	Intergenic	0.57	2.00×10^{-8}
	rs10963435	9	18,076,712	Intergenic	0.73	8.95×10^{-12}
	rs10963444	9	18,094,734	Intergenic	0.63	5.56×10^{-9}
	rs117450265	9	128,823,268	Intergenic	0.45	1.87×10^{-8}
	rs11010962	10	37,249,196	Intergenic	0.54	4.59×10^{-8}
	rs699575	12	63,294,839	*PPM1H*	0.55	1.04×10^{-8}
	rs182946220	14	53,313,177	Intergenic	0.64	2.68×10^{-9}
	rs186910020	14	53,313,181	Intergenic	0.57	3.35×10^{-8}
	rs113386802	17	768,645	*NXN*	0.60	2.12×10^{-8}
	rs75396458	17	769,249	*NXN*	0.60	2.12×10^{-8}
	rs77993536	20	62,296,349	*RTEL1; RTEL1-TNFRSF6B*	0.54	2.78×10^{-9}
PTK2	rs9729600	1	2,024,507	*PRKCZ*	4.31	4.02×10^{-10}
	rs3128329	1	2,029,392	*PRKCZ*	3.44	4.88×10^{-8}
	rs113156794	3	22,078,400	Intergenic	4.41	4.23×10^{-8}
RPS6KB1	rs185916002	8	119,564,946	*SAMD12*	1.61	6.85×10^{-9}
	rs190838060	8	119,564,948	*SAMD12*	1.66	4.05×10^{-9}
RPTOR	rs7443309	5	175,476,562	Intergenic	0.79	1.88×10^{-8}
	rs77741484	11	97,370,026	Intergenic	1.14	3.90×10^{-8}
	rs117396230	11	97,370,648	Intergenic	1.14	3.90×10^{-8}
	rs117709668	11	97,370,709	Intergenic	1.14	3.90×10^{-8}
	rs76791180	11	97,372,432	Intergenic	1.14	3.90×10^{-8}
	rs77459416	11	97,373,747	Intergenic	1.14	3.90×10^{-8}
	rs79673239	18	1,027,510	Intergenic	1.03	1.94×10^{-8}
SGK1	rs117934587	11	68,729,371	Intergenic	2.73	4.48×10^{-9}
SGK2	rs74450793	2	204,862,815	Intergenic	0.05	1.78×10^{-8}
SGK3	rs1420890	4	161,745,664	Intergenic	0.80	2.93×10^{-11}

SPP1	rs6824554	4	176,589,648	*GPM6A*	0.12	4.62×10^{-8}
	rs3776990	5	153,784,806	*GALNT10; SAP30L-AS1*	0.13	2.65×10^{-8}
	rs4958734	5	153,789,505	*GALNT10; SAP30L-AS1*	0.15	3.70×10^{-9}
	rs7702161	5	153,790,949	*GALNT10; SAP30L-AS1*	0.15	1.93×10^{-9}
	rs192934414	12	96,494,950	Intergenic	0.10	4.54×10^{-8}
	rs76978746	13	84,207,570	Intergenic	0.15	4.34×10^{-8}
	rs79234512	13	84,211,622	Intergenic	0.15	4.34×10^{-8}
	rs76515201	13	84,216,551	Intergenic	0.15	3.49×10^{-8}
SYK	rs94626	9	93,543,203	Intergenic	2.86	2.35×10^{-10}
	rs290276	9	93,545,268	Intergenic	-3.45	5.92×10^{-14}
	rs290274	9	93,546,913	Intergenic	-3.43	7.39×10^{-14}
	rs188313	9	93,548,302	Intergenic	-3.43	7.39×10^{-14}
	rs290271	9	93,551,619	Intergenic	-3.43	7.39×10^{-14}
	rs290268	9	93,552,450	Intergenic	-3.47	5.53×10^{-14}
	rs10448310	9	93,556,174	Intergenic	2.59	2.09×10^{-8}
	rs10821504	9	93,556,995	Intergenic	2.73	2.09×10^{-8}
	rs1333637	9	93,557,066	Intergenic	2.58	2.44×10^{-8}
	rs10993693	9	93,557,698	Intergenic	2.59	2.09×10^{-8}
	rs290997	9	93,559,351	Intergenic	-3.50	2.00×10^{-14}
	rs290995	9	93,559,477	Intergenic	-3.51	1.35×10^{-14}
	rs290994	9	93,559,515	Intergenic	-3.50	2.00×10^{-14}
	rs290993	9	93,559,572	Intergenic	-3.47	3.28×10^{-14}
	rs772407	9	93,560,850	Intergenic	-3.50	2.00×10^{-14}
	rs290992	9	93,561,309	Intergenic	3.48	4.02×10^{-14}
	rs290991	9	93,561,450	Intergenic	-3.50	2.00×10^{-14}
	rs290990	9	93,561,695	Intergenic	-3.50	2.00×10^{-14}
	rs290989	9	93,561,836	Intergenic	-3.50	2.00×10^{-14}
	rs290988	9	93,562,955	Intergenic	-3.50	2.00×10^{-14}
	rs290987	9	93,563,267	Intergenic	-3.50	2.00×10^{-14}
	rs2562397	9	93,563,992	*SYK*	-3.50	2.00×10^{-14}
	rs1319677	9	93,565,999	*SYK*	-3.38	9.99×10^{-14}
	rs1333635	9	93,566,406	*SYK*	-3.36	1.21×10^{-13}
	rs2780701	9	93,567,281	*SYK*	-3.29	3.54×10^{-13}
	rs2562396	9	93,567,425	*SYK*	-3.29	3.54×10^{-13}
	rs2774260	9	93,567,472	*SYK*	-3.29	3.54×10^{-13}
	rs1333634	9	93,567,503	*SYK*	-3.29	3.54×10^{-13}
	rs1333633	9	93,567,572	*SYK*	3.29	6.61×10^{-13}
	rs919386	9	93,567,607	*SYK*	3.24	1.19×10^{-12}
	rs1333632	9	93,567,732	*SYK*	3.31	3.68×10^{-13}
	rs2613308	9	93,567,773	*SYK*	-3.33	3.27×10^{-13}
	rs2780702	9	93,567,783	*SYK*	-3.33	3.27×10^{-13}
	rs17488335	9	93,567,958	*SYK*	-3.30	3.63×10^{-9}
	rs62554850	9	93,568,122	*SYK*	-3.44	7.49×10^{-10}
	rs4501705	9	93,568,534	*SYK*	-3.26	2.54×10^{-9}
	rs2126051	9	93,569,001	*SYK*	-3.45	6.20×10^{-10}
	rs2115485	9	93,569,254	*SYK*	-3.45	6.20×10^{-10}
	rs2115484	9	93,569,328	*SYK*	-3.46	6.12×10^{-10}
	rs10821508	9	93,569,500	*SYK*	-3.45	6.20×10^{-10}
	rs12553524	9	93,569,646	*SYK*	-3.45	6.20×10^{-10}

	rs4002565	9	93,569,810	*SYK*	-3.49	4.32×10^{-10}
	rs4002564	9	93,569,818	*SYK*	-3.49	4.47×10^{-10}
	rs1460834	9	93,570,060	*SYK*	-3.45	6.59×10^{-10}
	rs1460835	9	93,570,190	*SYK*	-3.45	6.20×10^{-10}
	rs58668584	9	93,570,432	*SYK*	-3.44	7.02×10^{-10}
	rs7046316	9	93,570,602	*SYK*	-3.45	6.20×10^{-10}
	rs7046173	9	93,571,124	*SYK*	-3.45	6.20×10^{-10}
	rs7046193	9	93,571,160	*SYK*	-3.45	6.20×10^{-10}
	rs3789890	9	93,571,427	*SYK*	-3.45	6.20×10^{-10}
	rs3789891	9	93,571,514	*SYK*	-3.45	6.20×10^{-10}
	rs3789892	9	93,571,559	*SYK*	-3.45	6.20×10^{-10}
	rs12551275	9	93,572,123	*SYK*	-3.45	6.20×10^{-10}
	rs7032733	9	93,572,345	*SYK*	-3.45	6.20×10^{-10}
	rs7020331	9	93,572,543	*SYK*	-3.40	1.04×10^{-9}
	rs11788207	9	93,572,856	*SYK*	-3.45	6.20×10^{-10}
	rs2042984	9	93,573,095	*SYK*	-3.45	6.20×10^{-10}
	rs2042983	9	93,573,162	*SYK*	-3.45	6.20×10^{-10}
	rs2162977	9	93,573,489	*SYK*	-3.51	5.03×10^{-10}
THBS1	rs77596211	1	185,393,593	Intergenic	0.22	3.04×10^{-8}
	rs142050687	2	80,113,679	*CTNNA2*	0.24	1.73×10^{-8}
	rs35674887	2	103,482,305	Intergenic	0.24	1.84×10^{-10}
	rs73039349	3	18,857,954	Intergenic	0.30	9.77×10^{-10}
	rs73469723	6	47,739,014	Intergenic	0.26	2.64×10^{-8}
	rs73469733	6	47,745,062	Intergenic	0.26	2.64×10^{-8}
	rs73469739	6	47,746,798	Intergenic	0.25	3.20×10^{-8}
	rs4098287	6	47,757,598	*OPN5*	0.24	4.15×10^{-8}
	rs6458594	6	47,767,452	*OPN5*	0.27	1.56×10^{-8}
	rs7766520	6	47,768,443	*OPN5*	0.27	1.56×10^{-8}
	rs142854523	6	47,786,461	*OPN5*	0.26	4.71×10^{-8}
	rs140354855	6	47,788,697	*OPN5*	0.26	4.71×10^{-8}
	rs140905188	6	47,790,787	*OPN5*	0.26	4.71×10^{-8}
	rs55683950	6	47,793,688	*OPN5*	0.26	4.71×10^{-8}
	rs116523017	6	47,794,640	Intergenic	0.26	4.71×10^{-8}
	rs111415861	6	47,868,831	*PTCHD4*	0.25	2.96×10^{-8}
	rs77803641	6	47,876,110	*PTCHD4*	0.27	3.57×10^{-9}
	rs79486983	6	47,876,566	*PTCHD4*	0.27	3.57×10^{-9}
	rs77139175	6	47,877,032	*PTCHD4*	0.27	3.57×10^{-9}
	rs78764095	6	47,880,331	*PTCHD4*	0.28	7.98×10^{-10}
	rs78630456	6	47,880,383	*PTCHD4*	0.28	7.98×10^{-10}
	rs74939207	6	47,881,542	*PTCHD4*	0.28	7.98×10^{-10}
	rs77910861	6	47,881,866	*PTCHD4*	0.28	7.98×10^{-10}
	rs28436057	11	67,450,926	Intergenic	0.26	1.16×10^{-8}
	rs12272021	11	67,451,636	Intergenic	0.26	1.16×10^{-8}
	rs4435009	11	67,452,345	Intergenic	0.26	2.46×10^{-8}
	rs12273945	11	67,452,860	Intergenic	0.26	1.16×10^{-8}
	rs12277666	11	67,454,567	Intergenic	0.26	1.30×10^{-8}
	rs12279483	11	67,455,738	Intergenic	0.26	1.16×10^{-8}
	rs61450467	11	67,458,462	Intergenic	0.26	1.16×10^{-8}
	rs2514046	11	67,459,163	Intergenic	0.26	2.46×10^{-8}

	rs2447574	11	67,459,179	Intergenic	0.26	2.46×10^{-8}
	rs4930482	11	67,460,529	Intergenic	0.26	2.46×10^{-8}
	rs73159087	13	23,467,408	Intergenic	0.26	1.41×10^{-8}
	rs112624055	15	61,494,341	*RORA*	0.26	2.95×10^{-8}
	rs113955400	15	61,494,354	*RORA*	0.26	2.95×10^{-8}
	rs79142868	18	73,695,242	Intergenic	0.21	3.08×10^{-9}
	rs75850871	18	73,695,643	Intergenic	0.21	6.41×10^{-9}
	rs74259264	18	73,702,385	Intergenic	0.22	1.53×10^{-9}
	rs17058680	18	73,702,786	Intergenic	0.21	1.36×10^{-8}
	rs11872245	18	73,705,389	Intergenic	0.21	1.36×10^{-8}
	rs11872247	18	73,705,421	Intergenic	0.21	1.36×10^{-8}
	rs17058694	18	73,706,015	Intergenic	0.21	1.36×10^{-8}
	rs79467161	18	73,709,292	Intergenic	0.21	9.79×10^{-9}
	rs75649221	18	73,710,923	Intergenic	0.21	9.79×10^{-9}
	rs117222991	18	73,711,197	Intergenic	0.21	9.79×10^{-9}
	rs74524122	18	73,715,052	Intergenic	0.25	9.35×10^{-12}
	rs59615307	18	73,715,519	Intergenic	0.22	7.16×10^{-9}
	rs77891592	18	73,721,195	Intergenic	0.20	3.67×10^{-8}
	rs1021997	18	73,721,453	Intergenic	0.21	2.08×10^{-8}
	rs17252748	18	73,724,463	Intergenic	0.21	2.08×10^{-8}
	rs76232710	18	73,729,959	Intergenic	0.21	2.08×10^{-8}
	rs17058711	18	73,730,213	Intergenic	0.21	2.08×10^{-8}
	rs61263430	18	73,738,823	Intergenic	0.20	3.67×10^{-8}
	rs17058719	18	73,740,261	Intergenic	0.20	3.67×10^{-8}
	rs74680368	18	73,740,708	Intergenic	0.20	3.67×10^{-8}
	rs17338384	18	73,741,610	Intergenic	0.20	3.67×10^{-8}
	rs75377303	18	73,742,541	Intergenic	0.20	3.30×10^{-8}
	rs61691938	21	37,603,942	*DOPEY2*	0.22	1.22×10^{-9}
	rs2284633	21	37,604,665	*DOPEY2*	0.22	1.22×10^{-9}
	rs147393817	21	37,607,934	*DOPEY2*	0.22	1.22×10^{-9}
	rs13054662	22	29,642,012	*EMID1*	0.26	3.17×10^{-8}
	rs13056227	22	29,645,556	*EMID1*	0.25	4.12×10^{-8}
	rs8136401	22	29,650,993	*EMID1*	0.26	2.37×10^{-8}
	rs34662558	22	29,653,410	*EMID1*	0.26	1.94×10^{-8}
	rs13056534	22	29,807,863	Intergenic	0.25	2.72×10^{-8}
THBS2	rs6593746	1	146,686,649	*FMO5*	0.05	4.57×10^{-8}
	rs141715081	1	146,779,089	Intergenic	0.05	4.32×10^{-8}
	rs75310765	1	146,779,915	Intergenic	0.06	2.06×10^{-8}
TNN	rs75004160	4	62,250,512	Intergenic	0.21	4.16×10^{-8}
TSC2	rs150412385	1	17,771,845	Intergenic	6.20	3.70×10^{-8}
	rs189438727	4	138,211,649	Intergenic	7.01	1.63×10^{-8}
	rs73196291	7	108,960,971	Intergenic	4.33	4.90×10^{-8}
	rs10236328	7	108,965,422	Intergenic	4.44	3.88×10^{-8}
	rs10241388	7	108,967,169	Intergenic	4.52	2.32×10^{-8}
	rs7784033	7	108,968,289	Intergenic	4.76	1.47×10^{-8}
	rs10257229	7	108,969,055	Intergenic	4.51	3.39×10^{-8}
	rs56695851	7	108,970,712	Intergenic	4.55	2.83×10^{-8}
	rs848845	7	109,000,649	Intergenic	4.40	1.48×10^{-8}
	rs848844	7	109,000,669	Intergenic	4.40	1.48×10^{-8}

rs10277209	7	109,003,574	Intergenic	4.41	2.43×10^{-8}
rs12669457	7	109,003,780	Intergenic	4.40	1.48×10^{-8}
rs1881445	7	109,005,041	Intergenic	4.27	2.41×10^{-8}
rs2204697	7	109,007,156	Intergenic	4.27	2.41×10^{-8}
rs66766955	7	109,007,642	Intergenic	4.38	2.81×10^{-8}
rs1406296	7	109,012,340	Intergenic	4.38	2.81×10^{-8}
rs1406297	7	109,012,375	Intergenic	4.27	2.41×10^{-8}
rs11763389	7	109,013,378	Intergenic	4.27	2.41×10^{-8}
rs7778753	7	109,013,749	Intergenic	4.27	2.41×10^{-8}
rs7798705	7	109,014,214	Intergenic	4.27	2.41×10^{-8}
rs56240857	7	109,019,236	Intergenic	4.38	2.81×10^{-8}
rs1581661	7	109,019,420	Intergenic	4.27	2.41×10^{-8}
rs6960556	7	109,019,960	Intergenic	4.27	2.41×10^{-8}
rs66775503	7	109,020,429	Intergenic	4.38	2.81×10^{-8}
rs7798394	7	109,020,695	Intergenic	4.27	2.41×10^{-8}
rs4730379	7	109,021,661	Intergenic	4.27	2.41×10^{-8}
rs149650943	7	109,022,630	Intergenic	4.41	2.44×10^{-8}
rs147324976	7	109,022,702	Intergenic	4.50	1.70×10^{-8}
rs7794651	7	109,028,297	Intergenic	4.27	2.41×10^{-8}
rs16885699	7	109,034,609	Intergenic	4.40	1.48×10^{-8}
rs13309729	7	109,034,692	Intergenic	4.40	1.48×10^{-8}
rs4730384	7	109,039,061	Intergenic	4.32	1.20×10^{-8}
rs1528536	7	109,039,712	Intergenic	4.32	1.84×10^{-8}
rs4454216	7	109,040,135	Intergenic	4.32	1.84×10^{-8}
rs12531719	7	109,047,058	Intergenic	4.44	1.12×10^{-8}
rs12705585	7	109,049,072	Intergenic	4.44	1.12×10^{-8}
rs4730385	7	109,049,758	Intergenic	4.35	1.82×10^{-8}
rs1406301	7	109,052,753	Intergenic	4.44	1.12×10^{-8}
rs9791486	7	109,053,208	Intergenic	4.44	1.12×10^{-8}
rs9791889	7	109,053,491	Intergenic	4.44	1.12×10^{-8}
rs2396099	7	109,053,967	Intergenic	4.44	1.12×10^{-8}
rs1590077	7	109,060,671	Intergenic	4.44	1.12×10^{-8}
rs1590078	7	109,060,758	Intergenic	4.44	1.12×10^{-8}
rs12705586	7	109,062,226	Intergenic	4.44	1.12×10^{-8}
rs73198437	7	109,069,417	Intergenic	4.39	1.53×10^{-8}
rs73198438	7	109,069,564	Intergenic	4.39	1.53×10^{-8}
rs11983814	7	109,071,166	Intergenic	4.39	1.53×10^{-8}
rs11982074	7	109,072,929	Intergenic	4.39	1.53×10^{-8}
rs11763160	7	109,074,474	Intergenic	4.39	1.53×10^{-8}
rs12532946	7	109,075,053	Intergenic	4.39	1.53×10^{-8}
rs10278820	7	109,079,428	Intergenic	4.39	1.53×10^{-8}
rs10256936	7	109,085,010	Intergenic	4.41	6.27×10^{-9}
rs190031944	10	87,277,598	Intergenic	5.51	2.11×10^{-8}
rs72875663	11	25,356,126	Intergenic	6.81	2.55×10^{-8}
rs35182105	12	24,572,337	*SOX5*	6.16	4.93×10^{-8}
rs4926276	19	13,459,988	*CACNA1A*	5.54	4.08×10^{-9}
rs4926155	19	13,460,244	*CACNA1A*	5.37	9.73×10^{-9}
rs4926277	19	13,460,986	*CACNA1A*	5.54	4.08×10^{-9}
rs9676969	19	13,462,117	*CACNA1A*	5.54	4.08×10^{-9}

	rs8111026	19	13,462,471	*CACNA1A*	5.54	4.08×10^{-9}
	rs8101350	19	13,462,547	*CACNA1A*	5.54	4.08×10^{-9}
	rs993464	19	13,462,969	*CACNA1A*	5.47	5.25×10^{-9}
	rs62109120	19	13,463,093	*CACNA1A*	5.54	4.08×10^{-9}
	rs62109121	19	13,463,606	*CACNA1A*	5.54	4.08×10^{-9}
	rs60160458	19	13,464,814	*CACNA1A*	5.54	4.08×10^{-9}
VTN	rs76410874	21	46,378,941	*FAM207A*	0.03	7.01×10^{-9}
YWHAB	rs11225671	11	103,172,268	*DYNC2H1*	12.38	4.97×10^{-9}
	rs12362743	11	103,174,035	*DYNC2H1*	11.70	4.16×10^{-8}
	rs72975625	11	103,175,850	*DYNC2H1*	11.70	4.16×10^{-8}
	rs11225676	11	103,177,706	*DYNC2H1*	11.70	4.16×10^{-8}
	rs72975630	11	103,178,161	*DYNC2H1*	11.70	4.16×10^{-8}
	rs11225677	11	103,179,752	*DYNC2H1*	12.46	1.28×10^{-8}
	rs7937201	11	103,180,450	*DYNC2H1*	11.70	4.16×10^{-8}
	rs11225687	11	103,182,929	*DYNC2H1*	11.70	4.16×10^{-8}
	rs12366065	11	103,184,396	*DYNC2H1*	12.46	1.28×10^{-8}
	rs11225702	11	103,185,595	*DYNC2H1*	12.46	1.28×10^{-8}
	rs11225705	11	103,186,455	*DYNC2H1*	11.70	4.16×10^{-8}
	rs72975657	11	103,187,169	*DYNC2H1*	11.70	4.16×10^{-8}
	rs11225710	11	103,188,934	*DYNC2H1*	11.70	4.16×10^{-8}
	rs7936583	11	103,193,338	*DYNC2H1*	11.26	2.54×10^{-8}
YWHAH	rs7290696	22	32,341,684	*YWHAH*	4.51	4.51×10^{-8}
	rs7291050	22	32,341,957	*YWHAH*	4.49	4.88×10^{-8}
	rs9606887	22	32,347,771	*YWHAH*	4.50	4.80×10^{-8}
	rs9606888	22	32,348,513	*YWHAH*	4.50	4.80×10^{-8}
	rs9621371	22	32,356,150	Intergenic	4.50	4.80×10^{-8}
YWHAZ	rs3134376	8	101,934,140	*YWHAZ*	-12.84	6.49×10^{-9}
	rs3105460	8	101,937,374	*YWHAZ*	13.11	4.49×10^{-9}
	rs964917	8	101,937,455	*YWHAZ*	12.85	9.44×10^{-9}
	rs1376672	8	101,939,479	*YWHAZ*	-12.84	6.49×10^{-9}
	rs3134380	8	101,943,319	*YWHAZ*	-12.84	6.49×10^{-9}
	rs3105452	8	101,943,618	*YWHAZ*	-12.84	6.49×10^{-9}
	rs3100053	8	101,944,559	*YWHAZ*	-12.84	6.49×10^{-9}
	rs4734498	8	101,946,548	*YWHAZ*	-12.68	1.09×10^{-8}
	rs3134353	8	101,947,453	*YWHAZ*	13.11	4.49×10^{-9}
	rs1470764	8	101,950,038	*YWHAZ*	13.11	4.49×10^{-9}
	rs3134356	8	101,952,506	*YWHAZ*	-12.81	7.88×10^{-9}
	rs3134358	8	101,958,433	*YWHAZ*	13.22	3.17×10^{-9}
	rs1901362	8	101,959,609	*YWHAZ*	-12.89	6.26×10^{-9}
	rs983583	8	101,961,910	*YWHAZ*	13.22	3.17×10^{-9}
	rs71506349	8	101,962,897	*YWHAZ*	-12.63	1.16×10^{-8}
	rs3100052	8	101,967,139	Intergenic	13.74	7.89×10^{-10}
	rs7817485	8	101,969,528	Intergenic	13.74	7.89×10^{-10}
	rs4734500	8	101,970,119	Intergenic	13.80	5.76×10^{-10}
	rs3019275	8	101,974,450	Intergenic	-12.63	1.05×10^{-8}
	rs2386921	8	101,975,378	Intergenic	12.26	1.90×10^{-8}
	rs2386922	8	101,975,479	Intergenic	12.26	1.90×10^{-8}
	rs2923781	8	101,976,510	Intergenic	12.26	1.90×10^{-8}
	rs3019261	8	101,978,163	Intergenic	12.34	1.48×10^{-8}

rs3019263	8	101,983,081	Intergenic	-12.76	5.52×10^{-9}

Abbreviations: SNV, single-nucleotide variant.
[a]bp from GRCh37.

Table S2 Expression quantitative trait loci for two or more of the genes involved in the PI3K/AKT pathway by a genome-wide association study ($P<5\times10^{-8}$).

eQTL				Expressed Genes [b]			
SNV	***Chr***	***Position*** [a]	***Gene***				
rs116248210	2	103,945,833	Intergenic	*COL4A1*	*COL4A2*		
rs116506968	2	183,551,471	Intergenic	*COL4A1*	*COL4A2*		
rs34845474	4	169,751,339	*PALLD*	*COL4A1*	*COL4A2*		
rs33951557	6	81,393,482	Intergenic	*COL4A1*	*COL4A2*		
rs55869773	7	4,498,947	Intergenic	*COL4A1*	*COL4A2*		
rs76598348	9	7,568,139	Intergenic	*COL4A1*	*COL4A2*		
rs1987249	9	108,683,134	Intergenic	*COL4A1*	*COL4A2*		
rs4276755	9	124,377,411	*DAB2IP*	*COL4A1*	*COL4A2*		
rs116674084	12	61,420,772	Intergenic	*COL4A1*	*COL4A2*		
rs182060879	12	85,650,851	Intergenic	*COL4A1*	*COL4A2*		
rs11867392	17	1,921,816	*RTN4RL1*	*COL4A1*	*COL4A2*		
rs6002762	22	22,910,191	Intergenic	*COL4A1*	*COL4A2*		
rs74617949	22	27,681,626	Intergenic	*COL4A1*	*COL4A2*		
rs12759318	1	22,308,435	*CELA3B*	*COL4A5*	*COL4A6*		
rs2506992	1	48,097,805	Intergenic	*COL4A5*	*COL4A6*		
rs17019582	2	4,702,434	*LINC01249*	*COL4A5*	*COL4A6*		
rs72992248	2	231,197,652	*SP140L*	*COL4A5*	*COL4A6*		
rs72766225	5	63,042,657	Intergenic	*COL4A5*	*COL4A6*		
rs17545761	5	146,647,488	*STK32A*	*COL4A5*	*COL4A6*		
rs78851618	6	147,047,296	*ADGB*	*COL4A5*	*COL4A6*		
rs16871986	7	105,399,650	*ATXN7L1*	*COL4A5*	*COL4A6*	*KITLG*	
rs117766049	7	129,684,199	*ZC3HC1*	*COL4A5*	*COL4A6*		
rs117417471	10	56,973,794	Intergenic	*COL4A5*	*COL4A6*	*F2R*	
rs7121626	11	5,379,516	*OR51B5*	*COL4A5*	*COL4A6*		
rs7964807[c]	12	74,484,387	Intergenic	*COL4A5*	*COL4A6*		
rs189351960[c]	12	74,560,964	*LOC100507377*	*COL4A5*	*COL4A6*		
rs117219682	14	59,558,654	Intergenic	*COL4A5*	*COL4A6*		
rs72721031	14	68,012,909	*PLEKHH1*	*COL4A5*	*COL4A6*		
rs7165046	15	97,778,882	Intergenic	*COL4A5*	*COL4A6*		
rs3784843	16	62,003,873	*CDH8*	*COL4A5*	*COL4A6*		
rs7226068	17	17,231,431	*NT5M*	*COL4A5*	*COL4A6*		
rs6474703	9	12,336,801	Intergenic		*COL4A6*	*KITLG*	
rs75927735	15	86,169,103	*AKAP13*	*CDKN1A*			*F2R*
rs2226155	1	113,865,778	Intergenic	*COL1A1*		*GHR*	
rs73024503	6	162,326,020	*PARK2*	*COL1A1*		*GHR*	
rs142713725	13	88,877,310	Intergenic	*COL1A1*		*GHR*	
rs75782643	14	90,785,989	*NRDE2*	*COL1A1*		*GHR*	
rs181029676	17	64,558,223	*PRKCA*	*COL1A1* [cis]		*GHR*	
rs7532303	1	44,864,049	Intergenic		*ITGA11*	*GHR*	
rs142534849	1	181,041,664	Intergenic		*ITGA11*	*GHR*	
rs62152138	2	103,324,335	*SLC9A2*		*ITGA11*	*GHR*	
rs73246227	5	117,457,849	*LOC102467224*		*ITGA11*	*GHR* [cis]	
rs111934886	6	14,339,896	Intergenic		*ITGA11*	*GHR*	
rs62446904	7	30,754,538	Intergenic		*ITGA11*	*GHR*	
rs74657816	7	46,670,682	Intergenic		*ITGA11*	*GHR*	
rs7917505	10	70,974,481	Intergenic		*ITGA11*	*GHR*	

rs72798056	16	50,184,574	Intergenic		*ITGA11*	*GHR*	
rs112716132	22	27,463,211	Intergenic		*ITGA11*	*GHR*	
rs79442714	1	113,833,382	Intergenic	*COL1A1*	*ITGA11*	*GHR*	
rs55932807	4	90,753,339	*SNCA*	*COL1A1*	*ITGA11*		
rs115973927	4	181,655,290	Intergenic		*ITGA11*	*GHR*	
rs76821212	6	165,921,617	*PDE10A*		*ITGA11*	*GHR*	
rs112981873	8	74,798,939	Intergenic	*COL1A1*		*GHR*	
rs6006621	22	44,419,407	*PARVB*		*ITGA11*	*GHR*	
rs13147782	4	131,034,260	Intergenic	*COL1A1*	*ITGA11*	*GHR*	*PDGFA*
rs150483042	3	90,431,083	Intergenic	*COL1A1*	*ITGA11*		
rs13128743	4	7,301,089	*SORCS2*	*COL1A1*	*ITGA11*		
rs189724000	4	129,628,727	Intergenic	*COL1A1*	*ITGA11*		
rs116135844	4	168,179,343	Intergenic	*COL1A1*	*ITGA11*		
rs190065357	10	134,951,797	Intergenic	*COL1A1*	*ITGA11*		
rs72658018	13	113,230,049	*TUBGCP3*	*COL1A1*	*ITGA11*		
rs192073405	17	69,639,501	Intergenic	*COL1A1*[cis]	*ITGA11*		
rs34419428	20	61,288,015	*SLCO4A1*	*COL1A1*	*ITGA11*		
rs67897587	12	13,293,153	Intergenic	*COL1A1*	*ITGA11*		*LAMB3*
rs10823526	10	72,115,475	*LRRC20*			*GHR*	*LAMC2*
rs45450199	10	72,182,110	*EIF4EBP2*			*GHR*	*LAMC2*
rs74769936	6	47,322,096	Intergenic	*FN1*	*LAMA1*		
rs17339635	10	14,487,154	Intergenic	*FN1*	*MET*		

Abbreviations: eQTL, expression quantitative locus; SNV, single-nucleotide variant.

[a]bp from GRCh37.

[b]The superscript 'cis' indicates cis-regulation. No superscript shows trans-regulation.

[c]The rs7964807 and rs189351960 were identified at one association signal. At the signal, rs7964807 was the most significant variant associated with expression of *COL4A5*, and rs189351960 was the most significant variant associated with expression of *COL4A6*.

Table S3 Functional regulation annotation for nucleotide variants associated with gene expression of *COL4A1* and *COL4A2* using RegulomeDB.

SNV	*Chr*	*Position* [a]	*Score* [b]	*Regulatory annotation* [c]
rs145692667	22	22,900,983	2b	Transcription factor binding+any motif+DNase footprint+DNase peak
rs2073725	22	22,891,307	3a	Transcription factor binding+any motif+DNase peak
rs12628694	22	22,905,692	3a	Transcription factor binding+any motif+DNase peak
rs4820469	22	22,911,059	3a	Transcription factor binding+any motif+DNase peak
rs33951557	6	81,393,481	3a	Transcription factor binding+any motif+DNase peak
rs11867392	17	1,921,815	4	Transcription factor binding+DNase peak
rs8405	22	22,890,269	4	Transcription factor binding+DNase peak
rs5996169	22	22,907,729	4	Transcription factor binding+DNase peak
rs78256678	7	140,156,722	4	Transcription factor binding+DNase peak
rs3747098	22	22,890,065	5	Transcription factor binding or DNase peak
rs35317486	22	22,902,727	5	Transcription factor binding or DNase peak
rs57306165	22	22,902,758	5	Transcription factor binding or DNase peak
rs62225994	22	22,902,782	5	Transcription factor binding or DNase peak
rs34575077	22	22,902,828	5	Transcription factor binding or DNase peak
rs2266993	22	22,903,464	5	Transcription factor binding or DNase peak
rs12628336	22	22,904,505	5	Transcription factor binding or DNase peak
rs12628961	22	22,905,573	5	Transcription factor binding or DNase peak
rs12628728	22	22,905,591	5	Transcription factor binding or DNase peak
rs12628708	22	22,905,785	5	Transcription factor binding or DNase peak
rs35340611	22	22,906,059	5	Transcription factor binding or DNase peak
rs4822132	22	22,906,900	5	Transcription factor binding or DNase peak
rs12484127	22	22,907,617	5	Transcription factor binding or DNase peak
rs6002751	22	22,909,311	5	Transcription factor binding or DNase peak
rs11705416	22	22,909,403	5	Transcription factor binding or DNase peak
rs11704554	22	22,909,423	5	Transcription factor binding or DNase peak
rs57882020	22	22,909,635	5	Transcription factor binding or DNase peak
rs6002757	22	22,909,962	5	Transcription factor binding or DNase peak
rs6002758	22	22,909,980	5	Transcription factor binding or DNase peak
rs6002759	22	22,909,982	5	Transcription factor binding or DNase peak
rs13218634	6	81,392,644	5	Transcription factor binding or DNase peak
rs34570868	6	81,399,463	5	Transcription factor binding or DNase peak
rs55869773	7	4,498,946	5	Transcription factor binding or DNase peak
rs1987249	9	108,683,133	5	Transcription factor binding or DNase peak
rs13293907	9	108,702,985	5	Transcription factor binding or DNase peak
rs4276755	9	124,377,410	5	Transcription factor binding or DNase peak
rs182060879	12	85,650,850	6	Motif hit
rs28692543	17	79,904,306	6	Motif hit
rs5996147	22	22,898,674	6	Motif hit
rs2266994	22	22,903,479	6	Motif hit
rs3827308	22	22,904,029	6	Motif hit
rs11090146	22	22,904,801	6	Motif hit
rs12484407	22	22,905,029	6	Motif hit

rs34159908	22	22,905,978	6	Motif hit
rs4822130	22	22,906,390	6	Motif hit
rs4822131	22	22,906,528	6	Motif hit
rs4822137	22	22,910,272	6	Motif hit
rs4822138	22	22,910,415	6	Motif hit
rs6002779	22	22,912,889	6	Motif hit
rs34845474	4	169,751,338	6	Motif hit
rs141374855	6	81,394,817	6	Motif hit
rs76886693	6	81,396,606	6	Motif hit
rs12003625	9	108,702,801	6	Motif hit
rs116674084	12	61,420,771	7	No Data
rs2266991	22	22,902,951	7	No Data
rs3827312	22	22,904,142	7	No Data
rs3827313	22	22,904,149	7	No Data
rs12484878	22	22,905,094	7	No Data
rs12628637	22	22,905,882	7	No Data
rs6002761	22	22,910,144	7	No Data
rs6002762	22	22,910,190	7	No Data
rs4822139	22	22,910,522	7	No Data
rs4820467	22	22,910,530	7	No Data
rs4820468	22	22,910,673	7	No Data
rs6002772	22	22,911,548	7	No Data
rs6002778	22	22,912,167	7	No Data
rs4822154	22	22,913,628	7	No Data
rs62226018	22	22,914,299	7	No Data
rs74617949	22	27,681,625	7	No Data
rs116248210	2	103,945,832	7	No Data
rs113126958	2	134,065,881	7	No Data
rs116506968	2	183,551,470	7	No Data
rs76024614	4	169,745,499	7	No Data
rs12001361	9	108,684,544	7	No Data
rs13292657	9	108,703,074	7	No Data
rs76598348	9	7,568,138	7	No Data
rs76060026	9	7,568,567	7	No Data

Abbreviations: SNV, single-nucleotide variant

[a]bp from GRCh37.

[b]Lower score indicates more evidence for a variant to be functional.

[c]Regulation was annotated based on the Encyclopedia of DNA Elements (ENCODE) data.

Table S4 Functional regulation annotation for nucleotide variants associated with gene expression of *COL4A5* and *COL4A6* using RegulomeDB.

SNV	*Chr*	*Position* [a]	*Score* [b]	*Regulatory annotation* [c]
rs7165046	15	97,778,881	2b	Transcription factor binding+any motif+DNase Footprint+DNase peak
rs75617695	12	74,460,438	3a	Transcription factor binding+any motif+DNase peak
rs112931164	9	140,294,495	3a	Transcription factor binding+any motif+DNase peak
rs78649901	5	146,675,509	4	Transcription factor binding+DNase peak
rs75819971	11	123,347,707	5	Transcription factor binding or DNase peak
rs80351845	12	74,476,821	5	Transcription factor binding or DNase peak
rs61196665	16	62,004,511	5	Transcription factor binding or DNase peak
rs56391958	16	62,005,708	5	Transcription factor binding or DNase peak
rs55973238	16	62,009,664	5	Transcription factor binding or DNase peak
rs2506992	1	48,097,804	5	Transcription factor binding or DNase peak
rs73154681	22	31,236,542	5	Transcription factor binding or DNase peak
rs72992248	2	231,197,651	5	Transcription factor binding or DNase peak
rs17019595	2	4,704,140	5	Transcription factor binding or DNase peak
rs57543595	5	146,650,913	5	Transcription factor binding or DNase peak
rs17468085	5	146,655,510	5	Transcription factor binding or DNase peak
rs75926181	5	146,657,498	5	Transcription factor binding or DNase peak
rs115884442	5	146,660,023	5	Transcription factor binding or DNase peak
rs80343345	5	146,660,757	5	Transcription factor binding or DNase peak
rs74374168	5	146,675,453	5	Transcription factor binding or DNase peak
rs78851618	6	147,047,295	5	Transcription factor binding or DNase peak
rs16871986	7	105,399,649	5	Transcription factor binding or DNase peak
rs11764066	7	129,694,737	5	Transcription factor binding or DNase peak
rs117417471	10	56,973,793	6	Motif hit
rs7121626	11	5,379,515	6	Motif hit
rs117888950	12	74,131,739	6	Motif hit
rs77097711	12	74,348,618	6	Motif hit
rs7954064	12	74,460,018	6	Motif hit
rs117898983	12	74,467,395	6	Motif hit
rs117866772	12	74,481,377	6	Motif hit
rs7964807	12	74,484,386	6	Motif hit
rs151196400	12	74,486,749	6	Motif hit
rs117957068	12	74,491,626	6	Motif hit
rs2365426	12	74,492,567	6	Motif hit
rs7959349	12	74,495,387	6	Motif hit
rs12320777	12	74,510,144	6	Motif hit
rs77573113	12	74,512,139	6	Motif hit
rs2049205	12	74,514,938	6	Motif hit
rs78966406	12	74,532,288	6	Motif hit
rs78321472	12	74,533,994	6	Motif hit
rs116882385	12	74,543,794	6	Motif hit
rs7972328	12	74,570,626	6	Motif hit
rs117679796	12	74,583,322	6	Motif hit

rs139746544	12	74,583,645	6	Motif hit
rs192929004	12	80,701,465	6	Motif hit
rs117219682	14	59,558,653	6	Motif hit
rs1510164	16	62,011,416	6	Motif hit
rs7226068	17	17,231,430	6	Motif hit
rs56070607	5	146,651,083	6	Motif hit
rs55778516	5	146,651,129	6	Motif hit
rs77652165	5	146,652,470	6	Motif hit
rs73794372	5	146,652,848	6	Motif hit
rs76535160	5	146,654,317	6	Motif hit
rs114514147	5	146,665,168	6	Motif hit
rs79220773	5	146,677,173	6	Motif hit
rs16930808	11	5,451,093	7	No Data
rs7297672	12	74,130,230	7	No Data
rs74479492	12	74,442,419	7	No Data
rs79308023	12	74,449,370	7	No Data
rs74690482	12	74,466,909	7	No Data
rs139034977	12	74,467,904	7	No Data
rs10506704	12	74,472,785	7	No Data
rs7965039	12	74,484,535	7	No Data
rs2365428	12	74,488,718	7	No Data
rs116975431	12	74,489,763	7	No Data
rs75478957	12	74,493,554	7	No Data
rs76640917	12	74,495,005	7	No Data
rs80090951	12	74,510,975	7	No Data
rs7960444	12	74,513,150	7	No Data
rs7306803	12	74,521,218	7	No Data
rs116887184	12	74,523,218	7	No Data
rs75674568	12	74,525,778	7	No Data
rs17768585	12	74,545,303	7	No Data
rs116929977	12	74,555,635	7	No Data
rs1522125	12	74,556,764	7	No Data
rs189351960	12	74,560,963	7	No Data
rs78736553	12	74,574,219	7	No Data
rs78088641	12	74,580,925	7	No Data
rs77620106	12	74,592,297	7	No Data
rs76881855	12	74,600,041	7	No Data
rs77504227	12	74,624,664	7	No Data
rs72721031	14	68,012,908	7	No Data
rs66704776	16	62,002,462	7	No Data
rs16964108	16	62,003,447	7	No Data
rs3784843	16	62,003,872	7	No Data
rs16964118	16	62,009,482	7	No Data
rs1397133	16	62,009,910	7	No Data
rs12759318	1	22,308,434	7	No Data
rs17019582	2	4,702,433	7	No Data

rs17545761	5	146,647,487	7	No Data
rs73794346	5	146,647,972	7	No Data
rs141016113	5	146,662,214	7	No Data
rs116480535	5	146,664,188	7	No Data
rs114316116	5	146,673,484	7	No Data
rs78772082	5	146,674,761	7	No Data
rs74991998	5	146,676,066	7	No Data
rs79541136	5	146,677,739	7	No Data
rs72766225	5	63,042,656	7	No Data
rs117766049	7	129,684,198	7	No Data

Abbreviations: SNV, single-nucleotide variant

[a]bp from GRCh37.

[b]Lower score indicates more evidence for a variant to be functional.

[c]Regulation was annotated based on the Encyclopedia of DNA Elements (ENCODE) data.

Table S5 Epistasis between nucleotide variants associated with gene expression of *COL4A1* and *COL4A2* ($P<2.16\times10^{-4}$).

SNV 1		***SNV 2***		***COL4A1***		***COL4A2***	
ID	***Chr***	***ID***	***Chr***	**β**	**P**	**β**	**P**
rs116248210	2	rs116506968	2	0.39	7.36×10^{-83}	1.20	4.76×10^{-162}
rs116248210	2	rs141398405	3	0.31	1.39×10^{-34}	1.07	2.49×10^{-76}
rs116248210	2	rs150938622	4	0.40	3.94×10^{-62}	1.36	7.62×10^{-213}
rs116248210	2	rs34845474	4	0.38	7.85×10^{-54}	1.31	3.41×10^{-168}
rs116248210	2	rs55926606	5	0.38	1.52×10^{-59}	1.23	9.46×10^{-136}
rs116248210	2	rs185142701	6	0.38	9.51×10^{-55}	1.20	9.81×10^{-114}
rs116248210	2	rs33951557	6	0.33	4.08×10^{-37}	1.19	1.41×10^{-114}
rs116248210	2	rs190578649	7	0.37	1.58×10^{-52}	1.22	4.59×10^{-128}
rs116248210	2	rs55869773	7	0.37	4.15×10^{-53}	1.29	4.84×10^{-167}
rs116248210	2	rs188696513	8	0.41	1.73×10^{-89}	1.27	1.86×10^{-214}
rs116248210	2	rs185444096	9	0.38	7.49×10^{-63}	1.23	6.79×10^{-148}
rs116248210	2	rs76598348	9	0.38	2.00×10^{-64}	1.22	5.84×10^{-155}
rs116506968	2	rs34845474	4	0.30	5.95×10^{-37}	0.93	1.67×10^{-59}
rs116506968	2	rs55926606	5	0.31	2.50×10^{-41}	1.03	2.67×10^{-86}
rs116506968	2	rs185142701	6	0.32	5.62×10^{-44}	1.06	3.61×10^{-92}
rs116506968	2	rs33951557	6	0.34	1.17×10^{-58}	1.04	5.73×10^{-92}
rs116506968	2	rs190578649	7	0.37	5.52×10^{-67}	1.14	1.14×10^{-121}
rs116506968	2	rs55869773	7	0.33	3.46×10^{-49}	1.06	7.62×10^{-100}
rs116506968	2	rs188696513	8	0.33	2.92×10^{-54}	1.02	1.71×10^{-88}
rs116506968	2	rs185444096	9	0.28	1.17×10^{-32}	0.90	1.09×10^{-58}
rs116506968	2	rs76598348	9	0.29	3.59×10^{-61}	0.82	7.89×10^{-71}
rs116506968	2	rs1987249	9	0.33	3.63×10^{-81}	0.92	2.26×10^{-90}
rs116506968	2	rs4276755	9	0.32	4.06×10^{-76}	0.89	1.42×10^{-87}
rs34845474	4	rs33951557	6	0.31	8.40×10^{-30}	1.14	8.61×10^{-85}
rs34845474	4	rs190578649	7	0.32	1.86×10^{-33}	1.18	1.32×10^{-97}
rs34845474	4	rs55869773	7	0.31	5.33×10^{-35}	1.06	4.82×10^{-72}
rs34845474	4	rs188696513	8	0.41	1.05×10^{-67}	1.33	1.07×10^{-181}
rs34845474	4	rs185444096	9	0.37	9.36×10^{-48}	1.30	4.44×10^{-167}
rs34845474	4	rs76598348	9	0.40	3.41×10^{-64}	1.31	8.49×10^{-171}
rs34845474	4	rs1987249	9	0.36	3.32×10^{-46}	1.30	4.65×10^{-163}
rs34845474	4	rs4276755	9	0.34	1.09×10^{-48}	1.09	1.09×10^{-105}
rs34845474	4	rs187110906	10	0.40	1.19×10^{-68}	1.26	1.75×10^{-160}
rs34845474	4	rs77848728	11	0.37	4.57×10^{-59}	1.19	3.78×10^{-132}
rs33951557	6	rs55869773	7	0.35	4.11×10^{-41}	1.24	6.52×10^{-119}
rs33951557	6	rs188696513	8	0.34	5.91×10^{-41}	1.17	8.80×10^{-104}

rs33951557	6	rs185444096	9	0.36	4.49×10^{-44}	1.29	5.20×10^{-151}
rs33951557	6	rs76598348	9	0.34	9.53×10^{-40}	1.21	7.31×10^{-114}
rs33951557	6	rs1987249	9	0.33	4.06×10^{-35}	1.19	1.21×10^{-105}
rs33951557	6	rs4276755	9	0.36	1.24×10^{-44}	1.22	9.13×10^{-120}
rs33951557	6	rs187110906	10	0.36	1.12×10^{-54}	1.14	8.50×10^{-110}
rs33951557	6	rs77848728	11	0.38	5.02×10^{-60}	1.23	7.82×10^{-137}
rs33951557	6	rs145907000	12	0.38	2.86×10^{-62}	1.21	1.73×10^{-141}
rs55869773	7	rs76598348	9	0.33	9.53×10^{-39}	1.23	1.13×10^{-129}
rs55869773	7	rs1987249	9	0.33	1.34×10^{-36}	1.19	6.15×10^{-105}
rs55869773	7	rs4276755	9	0.40	2.79×10^{-59}	1.39	1.86×10^{-237}
rs55869773	7	rs187110906	10	0.36	1.03×10^{-47}	1.27	1.45×10^{-144}
rs55869773	7	rs77848728	11	0.39	7.53×10^{-58}	1.35	8.95×10^{-204}
rs55869773	7	rs145907000	12	0.34	1.19×10^{-50}	1.06	1.04×10^{-90}
rs55869773	7	rs116674084	12	0.40	3.93×10^{-70}	1.28	8.88×10^{-173}
rs55869773	7	rs182060879	12	0.39	3.54×10^{-70}	1.21	1.68×10^{-143}
rs76598348	9	rs1987249	9	0.36	1.49×10^{-52}	1.23	$3.58\times10\text{-}^{133}$
rs76598348	9	rs4276755	9	0.29	8.27×10^{-32}	1.04	2.37×10^{-81}
rs76598348	9	rs187110906	10	0.36	2.94×10^{-51}	1.21	1.12×10^{-128}
rs76598348	9	rs77848728	11	0.34	1.06×10^{-42}	1.18	7.01×10^{-115}
rs76598348	9	rs145907000	12	0.26	1.42×10^{-28}	0.89	3.30×10^{-55}
rs76598348	9	rs116674084	12	0.38	1.02×10^{-65}	1.24	1.80×10^{-161}
rs76598348	9	rs182060879	12	0.35	7.09×10^{-54}	1.15	3.51×10^{-122}
rs1987249	9	rs4276755	9	0.34	7.08×10^{-39}	1.22	3.77×10^{-123}
rs1987249	9	rs187110906	10	0.34	1.31×10^{-43}	1.09	9.76×10^{-88}
rs1987249	9	rs77848728	11	0.34	4.79×10^{-39}	1.21	1.92×10^{-118}
rs1987249	9	rs145907000	12	0.33	2.98×10^{-43}	1.09	7.94×10^{-95}
rs1987249	9	rs116674084	12	0.39	6.01×10^{-67}	1.25	1.95×10^{-155}
rs1987249	9	rs182060879	12	0.39	8.20×10^{-70}	1.23	4.97×10^{-158}
rs4276755	9	rs116674084	12	0.33	1.22×10^{-38}	1.18	5.96×10^{-112}
rs4276755	9	rs182060879	12	0.32	2.98×10^{-36}	1.12	5.45×10^{-97}
rs4276755	9	rs181615907	13	0.29	2.73×10^{-35}	0.94	1.22×10^{-64}
rs4276755	9	rs141219139	14	0.41	3.84×10^{-79}	1.28	6.23×10^{-194}
rs4276755	9	rs185218318	15	0.36	1.05×10^{-53}	1.15	5.40×10^{-117}
rs116674084	12	rs182060879	12	0.30	1.17×10^{-31}	1.07	1.09×10^{-83}
rs116674084	12	rs181615907	13	0.39	6.38×10^{-77}	1.26	5.66×10^{-198}
rs116674084	12	rs141219139	14	0.36	1.72×10^{-54}	1.19	6.69×10^{-128}
rs116674084	12	rs185218318	15	0.36	1.23×10^{-56}	1.13	5.37×10^{-111}
rs182060879	12	rs11867392	17	0.33	5.85×10^{-48}	1.07	1.03×10^{-98}
rs182060879	12	rs112067749	18	0.38	8.68×10^{-62}	1.22	3.69×10^{-144}

rs182060879	12	rs56182540	19	0.32	5.39×10^{-40}	1.01	2.68×10^{-73}
rs11867392	17	rs6002762	22	0.34	2.05×10^{-78}	0.91	2.08×10^{-86}
rs11867392	17	rs74617949	22	0.34	3.90×10^{-87}	0.92	2.21×10^{-95}
rs6002762	22	rs74617949	22	0.35	1.03×10^{-83}	0.94	9.36×10^{-90}

Table S6 Epistasis between nucleotide variants associated with gene expression of *COL4A5* and *COL4A6* (P<2.16×10^{-4}).

SNV 1		***SNV 2***		***COL4A5***		***COL4A6***	
ID	***Chr***	***ID***	***Chr***	**β**	**P**	**β**	**P**
rs12759318	1	rs2506992	1	1.21	1.33×10^{-86}	0.32	1.06×10^{-74}
rs12759318	1	rs149483862	2	1.35	1.25×10^{-178}	0.33	6.88×10^{-90}
rs12759318	1	rs17019582	2	1.36	5.50×10^{-132}	0.35	1.85×10^{-95}
rs12759318	1	rs72992248	2	1.25	4.00×10^{-101}	0.33	1.04×10^{-81}
rs12759318	1	rs141398405	3	1.04	1.46×10^{-62}	0.30	1.08×10^{-82}
rs12759318	1	rs150938622	4	0.93	2.27×10^{-39}	0.26	2.99×10^{-40}
rs12759318	1	rs55926606	5	1.16	1.65×10^{-85}	0.30	7.55×10^{-74}
rs12759318	1	rs72766225	5	1.12	1.88×10^{-76}	0.33	6.35×10^{-105}
rs12759318	1	rs17545761	5	1.07	3.20×10^{-52}	0.31	6.26×10^{-70}
rs12759318	1	rs185142701	6	1.15	1.77×10^{-69}	0.32	2.17×10^{-75}
rs12759318	1	rs78851618	6	1.14	1.82×10^{-67}	0.32	2.84×10^{-70}
rs12759318	1	rs190578649	7	1.11	2.24×10^{-60}	0.30	2.61×10^{-62}
rs12759318	1	rs16871986	7	1.31	1.12×10^{-118}	0.36	7.63×10^{-123}
rs12759318	1	rs117766049	7	1.24	1.98×10^{-103}	0.35	7.25×10^{-111}
rs12759318	1	rs188696513	8	1.19	4.26×10^{-81}	0.31	1.36×10^{-70}
rs12759318	1	rs185444096	9	1.16	4.09×10^{-84}	0.31	9.78×10^{-76}
rs12759318	1	rs187110906	10	1.14	5.28×10^{-72}	0.29	6.01×10^{-58}
rs2506992	1	rs17019582	2	1.23	9.77×10^{-97}	0.32	3.56×10^{-62}
rs2506992	1	rs72992248	2	1.23	4.11×10^{-82}	0.34	1.18×10^{-72}
rs2506992	1	rs141398405	3	1.21	2.09×10^{-80}	0.34	1.47×10^{-74}
rs2506992	1	rs150938622	4	1.19	7.48×10^{-84}	0.32	1.86×10^{-73}
rs2506992	1	rs55926606	5	1.16	2.26×10^{-63}	0.35	1.15×10^{-77}
rs2506992	1	rs72766225	5	1.10	1.09×10^{-62}	0.29	1.05×10^{-51}
rs2506992	1	rs17545761	5	1.06	1.75×10^{-58}	0.27	4.16×10^{-45}
rs2506992	1	rs185142701	6	1.01	6.93×10^{-42}	0.30	6.12×10^{-51}
rs2506992	1	rs78851618	6	1.27	1.46×10^{-93}	0.34	2.03×10^{-74}
rs2506992	1	rs190578649	7	1.01	1.41×10^{-43}	0.29	3.37×10^{-47}
rs2506992	1	rs16871986	7	1.01	3.87×10^{-42}	0.29	9.65×10^{-48}
rs2506992	1	rs117766049	7	1.34	1.28×10^{-117}	0.35	1.68×10^{-87}
rs2506992	1	rs188696513	8	1.30	1.45×10^{-110}	0.34	8.22×10^{-83}
rs2506992	1	rs185444096	9	1.18	2.06×10^{-71}	0.33	6.97×10^{-66}
rs2506992	1	rs187110906	10	1.07	1.79×10^{-62}	0.29	3.04×10^{-54}
rs2506992	1	rs117417471	10	1.26	1.27×10^{-94}	0.33	1.59×10^{-74}
rs17019582	2	rs72992248	2	1.20	4.54×10^{-83}	0.30	1.08×10^{-54}
rs17019582	2	rs141398405	3	1.33	3.27×10^{-149}	0.35	1.24×10^{-89}

rs17019582	2	rs150938622	4	1.19	4.12×10^{-99}	0.29	4.79×10^{-59}
rs17019582	2	rs55926606	5	1.18	1.50×10^{-69}	0.36	1.02×10^{-88}
rs17019582	2	rs72766225	5	1.21	3.06×10^{-101}	0.31	5.90×10^{-68}
rs17019582	2	rs17545761	5	1.26	2.98×10^{-123}	0.31	5.20×10^{-74}
rs17019582	2	rs185142701	6	1.07	2.77×10^{-51}	0.31	2.92×10^{-58}
rs17019582	2	rs78851618	6	1.27	2.72×10^{-111}	0.32	1.46×10^{-65}
rs17019582	2	rs190578649	7	1.05	1.38×10^{-50}	0.32	3.54×10^{-64}
rs17019582	2	rs16871986	7	1.12	3.41×10^{-59}	0.34	4.96×10^{-72}
rs17019582	2	rs117766049	7	1.33	1.85×10^{-145}	0.34	2.27×10^{-89}
rs17019582	2	rs188696513	8	1.42	1.29×10^{-233}	0.36	5.20×10^{-111}
rs17019582	2	rs185444096	9	1.35	6.47×10^{-153}	0.34	3.75×10^{-85}
rs17019582	2	rs187110906	10	1.20	5.05×10^{-96}	0.31	2.93×10^{-67}
rs17019582	2	rs117417471	10	1.29	2.14×10^{-129}	0.33	1.08×10^{-76}
rs72992248	2	rs72766225	5	1.28	2.91×10^{-96}	0.34	1.98×10^{-80}
rs72992248	2	rs17545761	5	1.27	1.02×10^{-107}	0.33	5.76×10^{-79}
rs72992248	2	rs185142701	6	1.10	1.18×10^{-54}	0.34	1.05×10^{-71}
rs72992248	2	rs78851618	6	1.13	9.22×10^{-69}	0.29	3.08×10^{-52}
rs72992248	2	rs190578649	7	1.18	4.40×10^{-78}	0.30	4.62×10^{-62}
rs72992248	2	rs16871986	7	1.06	2.85×10^{-47}	0.31	7.62×10^{-59}
rs72992248	2	rs117766049	7	1.34	1.69×10^{-115}	0.35	3.87×10^{-88}
rs72992248	2	rs188696513	8	0.88	1.20×10^{-31}	0.27	1.05×10^{-41}
rs72992248	2	rs185444096	9	1.05	2.38×10^{-47}	0.31	7.41×10^{-61}
rs72992248	2	rs187110906	10	1.27	1.17×10^{-92}	0.34	8.78×10^{-81}
rs72992248	2	rs117417471	10	1.03	1.33×10^{-53}	0.28	8.27×10^{-49}
rs72992248	2	rs77848728	11	1.25	1.73×10^{-87}	0.33	2.79×10^{-68}
rs72992248	2	rs7121626	11	1.01	1.68×10^{-51}	0.27	6.51×10^{-44}
rs72992248	2	rs145907000	12	1.21	6.99×10^{-80}	0.34	3.29×10^{-83}
rs72766225	5	rs17545761	5	1.21	2.54×10^{-90}	0.32	2.20×10^{-76}
rs72766225	5	rs185142701	6	1.04	2.55×10^{-47}	0.32	1.90×10^{-62}
rs72766225	5	rs78851618	6	1.04	3.92×10^{-56}	0.28	8.27×10^{-49}
rs72766225	5	rs190578649	7	1.28	6.58×10^{-112}	0.34	2.37×10^{-90}
rs72766225	5	rs16871986	7	1.18	3.58×10^{-67}	0.34	3.53×10^{-78}
rs72766225	5	rs117766049	7	1.18	5.28×10^{-72}	0.32	4.79×10^{-62}
rs72766225	5	rs188696513	8	1.00	5.19×10^{-47}	0.30	7.88×10^{-57}
rs72766225	5	rs185444096	9	1.02	1.85×10^{-47}	0.30	1.09×10^{-54}
rs72766225	5	rs187110906	10	1.37	1.18×10^{-131}	0.36	6.58×10^{-98}
rs72766225	5	rs117417471	10	1.37	2.99×10^{-137}	0.36	1.36×10^{-104}
rs72766225	5	rs77848728	11	1.26	8.49×10^{-92}	0.33	6.11×10^{-71}
rs72766225	5	rs7121626	11	0.85	4.24×10^{-34}	0.22	1.99×10^{-29}

rs72766225	5	rs145907000	12	1.25	3.86×10^{-95}	0.32	1.22×10^{-67}
rs17545761	5	rs78851618	6	1.01	2.69×10^{-46}	0.29	6.43×10^{-53}
rs17545761	5	rs190578649	7	1.11	3.38×10^{-82}	0.32	7.39×10^{-103}
rs17545761	5	rs16871986	7	0.77	2.32×10^{-40}	0.26	4.75×10^{-83}
rs17545761	5	rs117766049	7	0.98	8.37×10^{-46}	0.32	1.29×10^{-84}
rs17545761	5	rs188696513	8	1.14	1.06×10^{-80}	0.34	1.14×10^{-108}
rs17545761	5	rs185444096	9	1.20	3.26×10^{-81}	0.34	2.32×10^{-88}
rs17545761	5	rs187110906	10	1.15	1.11×10^{-69}	0.32	3.34×10^{-76}
rs17545761	5	rs117417471	10	1.17	2.62×10^{-87}	0.35	5.93×10^{-128}
rs17545761	5	rs77848728	11	1.13	5.14×10^{-83}	0.33	1.14×10^{-111}
rs17545761	5	rs7121626	11	1.08	5.91×10^{-59}	0.28	7.07×10^{-52}
rs17545761	5	rs145907000	12	1.20	2.45×10^{-110}	0.35	2.51×10^{-152}
rs17545761	5	rs7964807	12	1.14	5.90×10^{-77}	0.29	1.19×10^{-60}
rs78851618	6	rs16871986	7	0.95	4.76×10^{-39}	0.28	6.52×10^{-46}
rs78851618	6	rs117766049	7	0.96	3.41×10^{-42}	0.27	4.15×10^{-45}
rs78851618	6	rs188696513	8	1.19	3.21×10^{-68}	0.35	2.41×10^{-83}
rs78851618	6	rs185444096	9	1.05	5.47×10^{-47}	0.31	1.39×10^{-54}
rs78851618	6	rs187110906	10	1.12	2.53×10^{-58}	0.33	2.48×10^{-68}
rs78851618	6	rs117417471	10	1.10	2.49×10^{-53}	0.33	1.09×10^{-70}
rs78851618	6	rs77848728	11	1.15	2.53×10^{-62}	0.32	5.67×10^{-65}
rs78851618	6	rs7121626	11	0.97	1.59×10^{-42}	0.29	4.44×10^{-50}
rs78851618	6	rs145907000	12	1.07	3.91×10^{-49}	0.32	2.22×10^{-58}
rs78851618	6	rs7964807	12	1.04	5.13×10^{-49}	0.30	5.60×10^{-56}
rs78851618	6	rs189351960	12	1.18	1.14×10^{-68}	0.36	1.10×10^{-91}
rs16871986	7	rs117766049	7	1.06	7.19×10^{-66}	0.31	1.30×10^{-84}
rs16871986	7	rs188696513	8	1.14	4.43×10^{-64}	0.34	1.58×10^{-93}
rs16871986	7	rs185444096	9	1.24	1.33×10^{-97}	0.35	4.48×10^{-109}
rs16871986	7	rs187110906	10	1.02	6.34×10^{-50}	0.29	4.02×10^{-54}
rs16871986	7	rs117417471	10	1.06	5.58×10^{-54}	0.30	7.02×10^{-58}
rs16871986	7	rs77848728	11	1.23	1.69×10^{-93}	0.35	1.89×10^{-108}
rs16871986	7	rs7121626	11	1.20	2.28×10^{-96}	0.33	3.19×10^{-94}
rs16871986	7	rs145907000	12	1.23	1.56×10^{-92}	0.32	6.87×10^{-74}
rs16871986	7	rs7964807	12	0.94	1.86×10^{-47}	0.26	1.54×10^{-50}
rs16871986	7	rs189351960	12	1.14	3.59×10^{-76}	0.29	1.86×10^{-57}
rs117766049	7	rs117417471	10	1.02	5.44×10^{-48}	0.33	1.86×10^{-89}
rs117766049	7	rs77848728	11	1.12	1.66×10^{-70}	0.34	3.50×10^{-100}
rs117766049	7	rs7121626	11	1.29	1.02×10^{-103}	0.37	9.06×10^{-124}
rs117766049	7	rs145907000	12	1.30	8.74×10^{-104}	0.36	9.94×10^{-121}
rs117766049	7	rs7964807	12	1.08	9.54×10^{-66}	0.31	2.01×10^{-84}

rs117766049	7	rs189351960	12	1.23	1.42×10^{-111}	0.37	4.70×10^{-182}
rs117766049	7	rs181615907	13	1.07	1.04×10^{-55}	0.27	5.21×10^{-42}
rs117766049	7	rs141219139	14	1.06	6.51×10^{-64}	0.31	5.21×10^{-81}
rs117766049	7	rs117219682	14	1.05	1.16×10^{-59}	0.27	8.49×10^{-47}
rs117417471	10	rs7121626	11	1.12	2.20×10^{-55}	0.34	4.51×10^{-80}
rs117417471	10	rs145907000	12	1.27	3.77×10^{-88}	0.36	1.33×10^{-107}
rs117417471	10	rs7964807	12	1.22	1.74×10^{-73}	0.33	3.23×10^{-76}
rs117417471	10	rs189351960	12	1.15	5.21×10^{-62}	0.35	2.58×10^{-88}
rs117417471	10	rs181615907	13	1.02	2.03×10^{-48}	0.31	6.78×10^{-69}
rs117417471	10	rs141219139	14	1.01	3.42×10^{-41}	0.29	2.76×10^{-45}
rs117417471	10	rs117219682	14	0.96	2.13×10^{-42}	0.30	1.32×10^{-63}
rs117417471	10	rs72721031	14	1.09	4.40×10^{-53}	0.32	8.65×10^{-65}
rs7121626	11	rs7964807	12	1.09	9.66×10^{-54}	0.32	4.17×10^{-64}
rs7121626	11	rs189351960	12	1.16	2.81×10^{-62}	0.33	1.99×10^{-72}
rs7121626	11	rs181615907	13	1.24	1.92×10^{-83}	0.35	6.39×10^{-89}
rs7121626	11	rs141219139	14	1.21	4.46×10^{-85}	0.35	3.14×10^{-92}
rs7121626	11	rs117219682	14	1.40	4.44×10^{-137}	0.37	6.53×10^{-104}
rs7121626	11	rs72721031	14	1.25	3.37×10^{-97}	0.35	1.55×10^{-103}
rs7121626	11	rs185218318	15	1.37	1.94×10^{-133}	0.36	3.28×10^{-99}
rs7964807	12	rs189351960	12	1.18	1.14×10^{-28}	0.37	2.53×10^{-39}
rs7964807	12	rs181615907	13	1.14	8.33×10^{-61}	0.33	3.54×10^{-72}
rs7964807	12	rs141219139	14	1.00	2.93×10^{-46}	0.29	3.20×10^{-49}
rs7964807	12	rs117219682	14	1.11	1.62×10^{-55}	0.32	5.60×10^{-65}
rs7964807	12	rs72721031	14	0.82	2.97×10^{-29}	0.25	9.45×10^{-37}
rs7964807	12	rs185218318	15	1.15	2.58×10^{-67}	0.35	1.60×10^{-92}
rs189351960	12	rs117219682	14	1.15	5.20×10^{-61}	0.33	6.59×10^{-70}
rs189351960	12	rs72721031	14	1.01	4.66×10^{-45}	0.29	6.50×10^{-50}
rs189351960	12	rs185218318	15	1.06	1.92×10^{-48}	0.31	7.55×10^{-57}
rs189351960	12	rs7165046	15	0.88	1.98×10^{-34}	0.26	3.82×10^{-43}
rs189351960	12	rs62028702	16	1.12	1.87×10^{-60}	0.34	1.32×10^{-82}
rs117219682	14	rs72721031	14	1.12	7.72×10^{-71}	0.32	3.44×10^{-81}
rs117219682	14	rs185218318	15	1.33	1.58×10^{-111}	0.35	4.36×10^{-87}
rs117219682	14	rs7165046	15	1.19	5.70×10^{-80}	0.34	4.88×10^{-98}
rs117219682	14	rs62028702	16	1.06	3.36×10^{-56}	0.28	1.15×10^{-47}
rs72721031	14	rs7165046	15	1.05	6.60×10^{-56}	0.28	2.63×10^{-47}
rs72721031	14	rs62028702	16	1.17	9.79×10^{-85}	0.33	4.90×10^{-88}
rs72721031	14	rs3784843	16	1.08	1.90×10^{-64}	0.29	1.40×10^{-56}
rs7165046	15	rs3784843	16	1.23	2.94×10^{-92}	0.32	1.06×10^{-73}
rs7165046	15	rs62053745	17	1.24	5.16×10^{-88}	0.33	1.05×10^{-74}

rs3784843	16	rs7226068	17	1.01	4.39×10^{-53}	0.26	4.73×10^{-43}
